# Tripartite Combination of Candidate Pandemic Mitigation Agents: Vitamin D, Quercetin, and Estradiol Manifest Properties of Medicinal Agents for Targeted Mitigation of the COVID-19 Pandemic Defined by Genomics-Guided Tracing of SARS-CoV-2 Targets in Human Cells

**DOI:** 10.3390/biomedicines8050129

**Published:** 2020-05-21

**Authors:** Gennadi V. Glinsky

**Affiliations:** Institute of Engineering in Medicine, University of California, San Diego, 9500 Gilman Dr. MC 0435, La Jolla, CA 92093-0435, USA; gglinskii@ucsd.edu; Tel.: +1-858-401-3470

**Keywords:** COVID-19, SARS-CoV-2 coronavirus, genomics, mitigation approaches, drugs and medicinal substance repurposing, vitamin D, quercetin, luteolin, eriodictyol, estradiol

## Abstract

Genes required for SARS-CoV-2 entry into human cells, ACE2 and FURIN, were employed as baits to build genomic-guided molecular maps of upstream regulatory elements, their expression and functions in the human body, and pathophysiologically relevant cell types. Repressors and activators of the ACE2 and FURIN genes were identified based on the analyses of gene silencing and overexpression experiments as well as relevant transgenic mouse models. Panels of repressors (*VDR; GATA5; SFTPC; HIF1a*) and activators (*HMGA2; INSIG1; RUNX1; HNF4a; JNK1/c-FOS*) were then employed to identify existing drugs manifesting in their effects on gene expression signatures of potential coronavirus infection mitigation agents. Using this strategy, vitamin D and quercetin have been identified as putative 2019 coronavirus disease (COVID-19) mitigation agents. Quercetin has been identified as one of top-scoring candidate therapeutics in the supercomputer SUMMIT drug-docking screen and Gene Set Enrichment Analyses (GSEA) of expression profiling experiments (EPEs), indicating that highly structurally similar quercetin, luteolin, and eriodictyol could serve as scaffolds for the development of efficient inhibitors of SARS-CoV-2 infection. In agreement with this notion, quercetin alters the expression of 98 of 332 (30%) of human genes encoding protein targets of SARS-CoV-2, thus potentially interfering with functions of 23 of 27 (85%) of the SARS-CoV-2 viral proteins in human cells. Similarly, Vitamin D may interfere with functions of 19 of 27 (70%) of the SARS-CoV-2 proteins by altering expression of 84 of 332 (25%) of human genes encoding protein targets of SARS-CoV-2. Considering the potential effects of both quercetin and vitamin D, the inference could be made that functions of 25 of 27 (93%) of SARS-CoV-2 proteins in human cells may be altered. GSEA and EPEs identify multiple drugs, smoking, and many disease conditions that appear to act as putative coronavirus infection-promoting agents. Discordant patterns of testosterone versus estradiol impacts on SARS-CoV-2 targets suggest a plausible molecular explanation of the apparently higher male mortality during the coronavirus pandemic. Estradiol, in contrast with testosterone, affects the expression of the majority of human genes (203 of 332; 61%) encoding SARS-CoV-2 targets, thus potentially interfering with functions of 26 of 27 SARS-CoV-2 viral proteins. A hypothetical tripartite combination consisting of quercetin/vitamin D/estradiol may affect expression of 244 of 332 (73%) human genes encoding SARS-CoV-2 targets. Of major concern is the ACE2 and FURIN expression in many human cells and tissues, including immune cells, suggesting that SARS-CoV-2 may infect a broad range of cellular targets in the human body. Infection of immune cells may cause immunosuppression, long-term persistence of the virus, and spread of the virus to secondary targets. Present analyses and numerous observational studies indicate that age-associated vitamin D deficiency may contribute to the high mortality of older adults and the elderly. Immediate availability for targeted experimental and clinical interrogations of potential COVID-19 pandemic mitigation agents, namely vitamin D and quercetin, as well as of the highly selective (Ki, 600 pm) intrinsically specific FURIN inhibitor (a1-antitrypsin Portland (a1-PDX), is considered an encouraging factor. Observations reported in this contribution are intended to facilitate follow-up targeted experimental studies and, if warranted, randomized clinical trials to identify and validate therapeutically viable interventions to combat the COVID-19 pandemic. Specifically, gene expression profiles of vitamin D and quercetin activities and their established safety records as over-the-counter medicinal substances strongly argue that they may represent viable candidates for further considerations of their potential utility as COVID-19 pandemic mitigation agents. In line with the results of present analyses, a randomized interventional clinical trial evaluating effects of estradiol on severity of the coronavirus infection in COVID19+ and presumptive COVID19+ patients and two interventional randomized clinical trials evaluating effects of vitamin D on prevention and treatment of COVID-19 were listed on the ClinicalTrials.gov website.

## 1. Introduction

The 2019 coronavirus disease pandemic (COVID-19), caused by the newly emerged SARS-CoV-2 virus is rapidly transitioning through the dangerous acute phase of its evolution in the United States. The absence of a vaccine and lack of efficient targeted therapeutic approaches emphasizes the urgent need for identification of candidate pandemic mitigation agents among existing drugs and medicinal substances.

SARS-CoV-2 virus was discovered in December 2019 and shortly thereafter it was isolated and sequenced [1,2]. Recent analyses of the structure, function, and antigenicity of the SARS-CoV-2 spike glycoprotein revealed the key role of the *ACE2* and *FURIN* genes in facilitating the high-affinity binding of viral particles and their entry into human cells [3]. The efficient invasion of host cells by SARS-CoV-2 is further enhanced by the presence of the unexpected FURIN cleavage site, which is cleaved during biosynthesis [3]. This novel feature distinguishes the previously known SARS-CoV and the newly emerged SARS-CoV-2 viruses and possibly contributes to the expansion of the cellular tropism of SARS-CoV-2 [3]. The crystal structure and high-resolution cryo-electron microscopy of the SARS-CoV-2 receptor-binding domain (RBD) in complex with human ACE2 revealed specific structural features of the SARS-CoV-2 RBD that appear to enhance its binding affinity to human ACE2 [4,5]. Collectively, these observations firmly established protein products of the human genes *ACE2* and *FURIN* as the principal mediators of the SARS-CoV-2 invasion into human cells, acting as the high-affinity receptor (ACE2) and invasion-promoting protease (FURIN), respectively.

In this contribution, genomic screens were performed employing the *ACE2* and *FURIN* genes as baits to build genomic-guided human tissues-tailored maps of upstream regulatory elements, their expression and functions. To identify the high-priority list of potential candidate mitigation agents, validation analyses were performed using gene silencing and overexpression experiments as well as relevant transgenic mouse models with the emphasis on pathophysiologically relevant cell types. Panels of repressors (*VDR; GATA5; SFTPC; HIF1a*) and activators (*HMGA2; INSIG1; RUNX1; HNF4a; JNK1/c-FOS*) of *ACE2* and *FURIN* expression were identified and then employed to identify existing drugs and medicinal substances that could be repurposed to ameliorate the outcomes of the coronavirus infection. Two of the most promising candidate mitigation agents, namely vitamin D and quercetin, manifest gene expression-altering activities and have established safety records as over-the-counter medicinal substances that seem sufficient for further assessment and considerations of their potential utility for amelioration of the clinical course of the coronavirus pandemic. Collectively, observations reported in this contribution indicate that highly structurally similar quercetin, luteolin, and eriodictyol could serve as scaffolds for the development of efficient inhibitors of SARS-CoV-2 infection. Unexpectedly, present analyses revealed discordant patterns of testosterone versus estradiol impacts on SARS-CoV-2 targets with the former manifesting potential coronavirus infection-promoting activities, which is consistent with the apparently higher male mortality across all age groups during the coronavirus pandemic. Significantly, in agreement with findings reported herein, numerous observational studies suggest that age-associated vitamin D insufficiency and/or deficiency may contribute to the high mortality of older adults and elderly individuals during the COVID-19 pandemic. Consequently, vitamin D supplementation may mitigate the severity of the disease.

## 2. Methods

### 2.1. Data Source and Analytical Protocols

All data analyzed in this study were obtained from publicly available sources. Gene set enrichment analyses (GSEA) were carried out using the Enrichr bioinformatics platform, which enables the interrogation of nearly 200,000 gene sets from more than 100 gene set libraries. The Enrichr API (January 2020 to March 2020 releases) [6,7] was used to test genes linked to the ACE2 and FURIN genes (or other genes of interest) for the significant enrichment of numerous functional categories. In all tables and plots (unless stated otherwise), in addition to the nominal *p* values and adjusted *p* values, the “combined score” calculated by Enrichr is reported, which is a product of the significance estimate and the magnitude of enrichment (combined score c = log(*p*) * z, where *p* is the Fisher’s exact test *p*-value and z is the z-score deviation from the expected rank). The validation of the GSEA findings was carried out by employing computational retrievals and manual curations of the gene expression profiles of the Gene Expression Omnibus (GEO) database. The final selection of the top-scoring candidates was made based on the consensus observations documented by the GSEA and expression profiling experiments (EPEs).

### 2.2. Statistical Analyses of the Publicly Available Datasets

All statistical analyses of the publicly available genomic datasets, including error rate estimates, background and technical noise measurements and filtering, feature peak calling, feature selection, assignments of genomic coordinates to the corresponding builds of the reference human genome, and data visualization, were performed exactly as reported in the original publications [8,9,10,11,12,13,14] and associated references linked to the corresponding data visualization tracks (http://genome.ucsc.edu/). Any modifications or new elements of statistical analyses are described in the corresponding sections of the results. The statistical significance of the Pearson correlation coefficients was determined using GraphPad Prism version 6.00 software. Both nominal and Bonferroni-adjusted *p* values were estimated. The statistical significance between the mean values was estimated using Student’s t-test. The significance of the differences in the numbers of events between the groups was calculated using the two-sided Fisher’s exact and chi square tests, and the significance of the overlap between the events was determined using the hypergeometric distribution test [15].

## 3. Results and Discussion

### 3.1. Enrichr-Guided Gene Set Enrichment Analyses (GSEA) of Genomic Features Associated with the ACE2 and FURIN Genes

**Figure 1:**
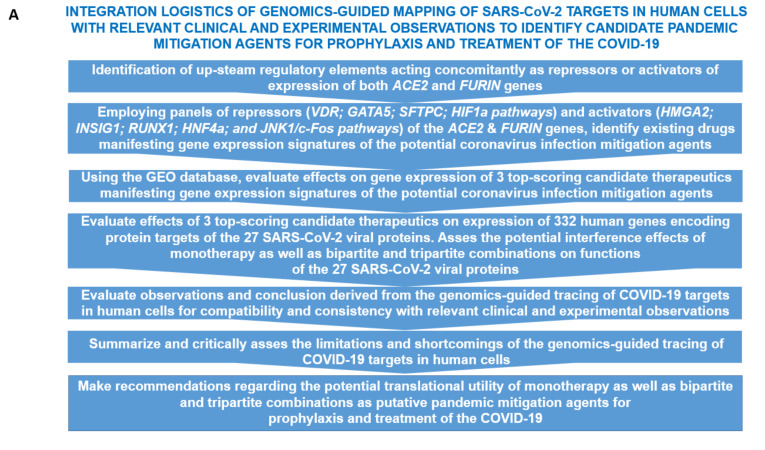

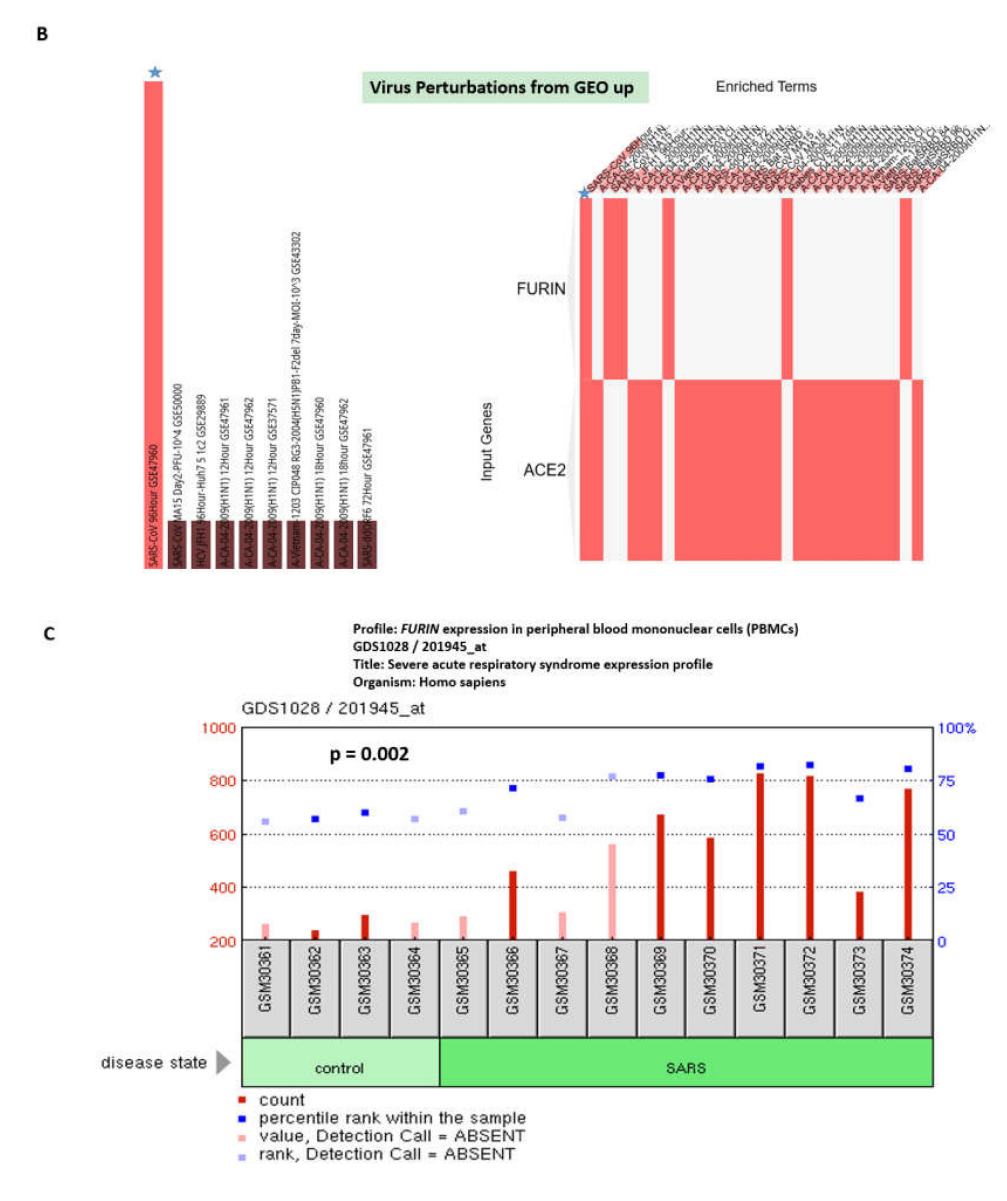
Genomic-guided mapping of regulatory networks affecting expression of human genes encoding protein targets of SARS-CoV-2 enables identification of putative 2019 coronavirus disease (COVID-19) mitigation agents. (**A**). Flow chart of a decision-making process during the identification of candidate pandemic mitigation agents employing genomic-guided tracing of genetic regulators and biological and chemical effectors of SARS-CoV-2 targets in human cells. (**B**) and (**C**). Effects of viral challenges on expression of the *ACE2* and *FURIN* genes. (**B**). Gene Set Enrichment Analyses (GSEA) of the Virus Perturbations from GEO focused on upregulated genes (Enrichr bioinformatics platform). SARS-CoV *p* value = 0.0002; *q* value = 0.072). Star denotes the SARS-CoV record at the 96 hrs. (**C**). Increased *FURIN* expression in peripheral blood mononuclear cells (PBMC) of patients with severe acute respiratory syndrome (SARS). *p* value = 0.002; *q* value = 0.126.

One of the goals of this work was to identify human genes implicated in regulatory cross talks affecting expression and functions of the *ACE2* and *FURIN* genes to build a model of genomic regulatory interactions potentially affecting SARS-CoV-2 infection. To this end, the Enrichr bioinformatics platform was utilized (see Methods; [6,7]) at the initial stage of the analyses and the identified records and top-scoring candidate features were further interrogated using targeted evaluation of the publicly available records of the Gene Expression Omnibus (GEO) database. GSEA were carried out using the *ACE2* and *FURIN* genes as baits applied to a broad spectrum of genomic databases reflecting the current state of knowledge regarding the structural, functional, regulatory, and pathophysiological features that could be statistically linked to these genes (Figure 1). Expression profiling experiments and GSEA revealed ubiquitous patterns of both *ACE2* and *FURIN* genes across human tissues (Supplementary Figure S1) with notable examples of high expression of the *FURIN* gene in the lung (the second-ranked tissue in the GTEX database) and testis being identified as the top-ranked *ACE2*-expressing tissue. In addition to the human lung, tagged by the *ACE2* expression in the ACRHS4 Human Tissues database search [16], other noteworthy significantly enriched records are the Peripheral Blood Mononuclear Cells (PBMC), Natural Killer Cells and Macrophages, tagged by *FURIN* expression (Supplementary Figure S1).

GSEA of the virus perturbations’ data sets among Gene Expression Omnibus (GEO) records of upregulated genes identified the SARS-CoV challenge at 96 h (GSE47960) as the most significantly enriched record (Supplementary Figure S2), tagged by the expression of both *ACE2* and *FURIN* in human airway epithelial cells. These observations suggest that coronavirus infection triggers the increased expression of both *ACE2* and *FURIN* genes 4 days after the initial encounter with host cells (Figure 1; Supplementary Figure S2). These findings were corroborated by the increased *FURIN* expression documented in the PBMC of patients with severe acute respiratory syndrome (Figure 1; Supplementary Figure S1; [17]). It would be of interest to investigate whether this potentially infection-promoting effect on the expression of the host genes in virus-targeted cells is mediated by the virus-induced release of biologically active molecules with the paracrine mode of actions such as interferons, interleukins and cytokines.

GSEA identified numerous significantly enriched records of common human disorders manifesting the upregulation of either *ACE* or *FURIN* genes (Supplementary Figure S3), which is consistent with the clinical observations that individuals with underlying health conditions are more likely to have clinically severe and lethal coronavirus infection.

Of note, both seasonal and pandemic H1N1 influenza virus infection significantly increases the *ACE2* expression in human bronchial epithelial cells in vitro (Supplementary Figure S3). Exploration of the DisGeNET database of human disorders highlighted multiple disease state records manifesting the altered expression of either *ACE2* or *FURIN* genes (Supplementary Figure S3). Cigarette smoking appears to significantly increase the *ACE2* expression in human large airway epithelial cells (Supplementary Figure S3), indicating that cigarette smoking should be considered as a potential coronavirus infection-promoting agent.

Gene Ontology (GO) analyses revealed that *ACE2* and *FURIN* genes are associated with the largely non-overlapping records of GO Biological Processes, GO Molecular Functions, and GO Cellular Components (Supplementary Figure S4). The common significantly enriched records are Viral Life Cycle (GO Biological Process 2018); Peptidase activity (acting on L-amino acid peptides) and Endopeptidase activity (GO Molecular Function 2018); Membrane raft (GO Cellular Component 2018); Meprin A complex and Retrotrasposon nucleocapsid (Jensen Compartments).

### 3.2. Identifications of the Enriched Records of Transcription Factor-Binding Sites Affecting the ACE2 and FURIN Expression

GSEA of the enriched records of transcription factor binding sites (TFBS) using ENCODE TF ChIP-seq 2015 and ChEA 2016 databases revealed predominantly distinct patterns of TFBS associated with the *ACE2* and *FURIN* genes (Supplementary Figure S5). Common TFBS shared by both *ACE2* and *FURIN* genes are *FOS*, *JUND*, *EP300* (ENCODE TF ChIP-seq 2015) and *GATA1, GATA2, RUNX1, FOXA1, HNF4A* (ChIP-seq 2015). Consistent with these findings, non-overlapping profiles of significantly enriched records associated with either *ACE2* or *FURIN* genes were observed of pathways (BioPlanet 2019 database), protein–protein interactions (PPI) hub proteins (PPI Hub Proteins database), and drugs affecting *ACE2* and *FURIN* expression (Drug Signatures Database, DSigDB), indicating that regulatory mechanisms governing the expression and activities of the *ACE2* and *FURIN* genes are predominantly non-overlapping and discordant (Supplementary Figure S5).

**Figure 2:**
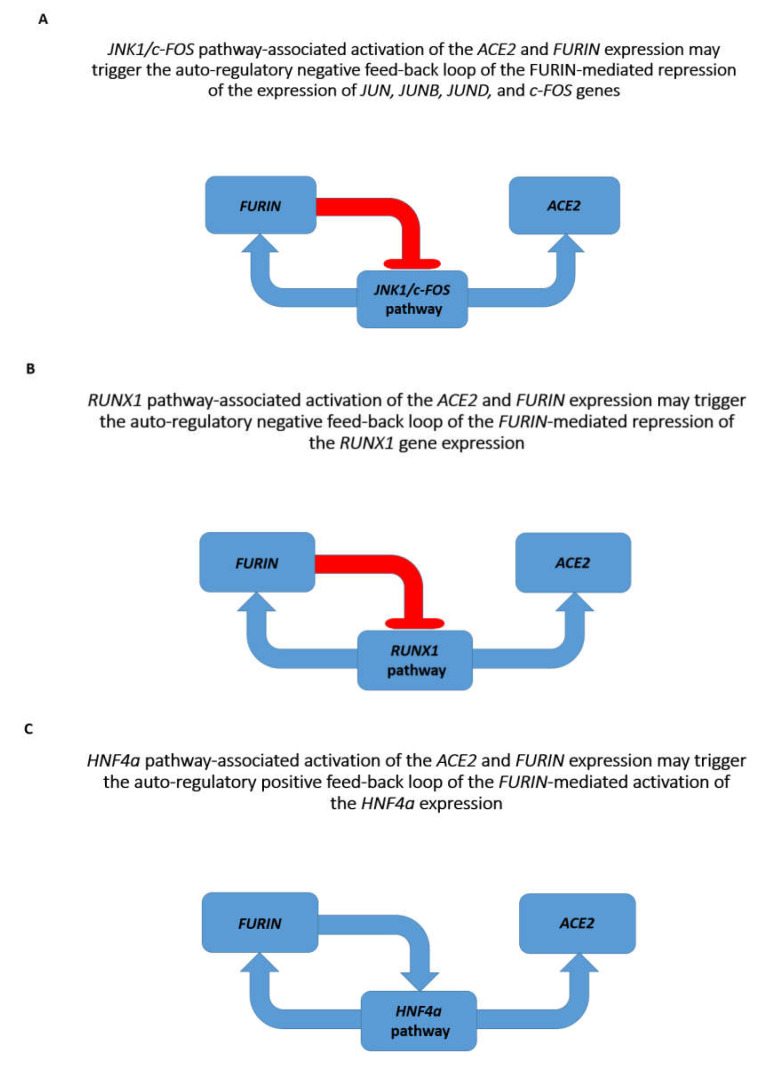
Pathways and genes affecting the newly emerged SARS-CoV-2 virus-related host targets. (**A**). *JNK1/c-FOS* pathway-associated activation of the *ACE2* and *FURIN* expression may trigger the auto-regulatory negative feed-back loop of the *FURIN*-mediated repression of the expression of *JUN, JUNB, JUND,* and *c-FOS* genes. (**B**). *RUNX1* pathway-associated activation of the *ACE2* and *FURIN* expression may trigger the auto-regulatory negative feed-back loop of the *FURIN*-mediated repression of the *RUNX1* gene expression (**C**). *HNF4a* pathway-associated activation of the *ACE2* and *FURIN* expression may trigger the auto-regulatory positive feed-back loop of the *FURIN*-mediated activation of the *HNF4a* expression.

Next, the Gene Expression Omnibus (GEO) database was interrogated to gauge the effects on *ACE2* and *FURIN* expression of transcription factors having TFBS associated with their promoters. There are multiple relevant GEO records reporting the activation effects of the *JNK1*/*c-FOS* pathway on *ACE2* and *FURIN* expression as well as the activation effects of *FURIN* depletion on expression of the *Fos*, *Jun*, *Jund*, and *Junb* genes (Supplementary Figure S5). Conversely, *c-Jun* inhibition (effect of the dominant negative *c-Jun*) or *c-Jun* depletion (*c-Jun* knockout) has resulted in decreased expression of the *FURIN* gene (Supplementary Figure S5). The summary of these observations is reported in Figure 2.

Similarly, there are several reports indicating that depletion of either *Hnf4a* or *Runx1* in mouse cells and *RUNX1* in human cells decreases the *ACE2* and *FURIN* expression (Supplementary Figure S6). Conversely, *FURIN* depletion enhances the expression of the *Runx1* and *Foxa1* genes in murine T cells (Supplementary Figure S6). In contrast, *FURIN* depletion decreases the expression of the *Hnf4a* gene, while *Hnf4a* depletion decreases the *FURIN* gene expression (Supplementary Figure S6). The summary of these observations is reported in Figure 2.

### 3.3. Identification of the VDR and HIF1a Genes as Putative Repressors of the ACE2 Expression

Next, GSEA of genomic databases were performed to identify the potential activators and repressors of the *ACE2* and *FURIN* genes. Analysis of the ARCHS4 transcription factor co-expression database identified the *VDR* gene that co-expressed with both *ACE2* and *FURIN* genes in human tissues (Supplementary Figure S7). Other significantly enriched records manifest non-overlapping patterns of co-expression with either *ACE2* or *FURIN* genes. The GTEX expression profile of the *VDR* gene in human tissues revealed the ubiquitous pattern of expression and placed the *VDR* expression in human lungs in the top quartile (Supplementary Figure S7). Analysis of gene expression profiling experiments of wild type and vitamin D receptor (*Vdr*) knockout primary bone marrow-derived macrophages reported in [18] demonstrated increased expression of the *ACE2* gene in the *Vdr* knockout cells (Supplementary Figure S7), implicating the product of the *VDR* gene as the putative repressor of the *ACE2* expression. Consistent with this hypothesis, vitamin D appears to inhibit the *ACE2* expression in human bronchial smooth muscle cells (Supplementary Figure S7).

**Figure 3:**
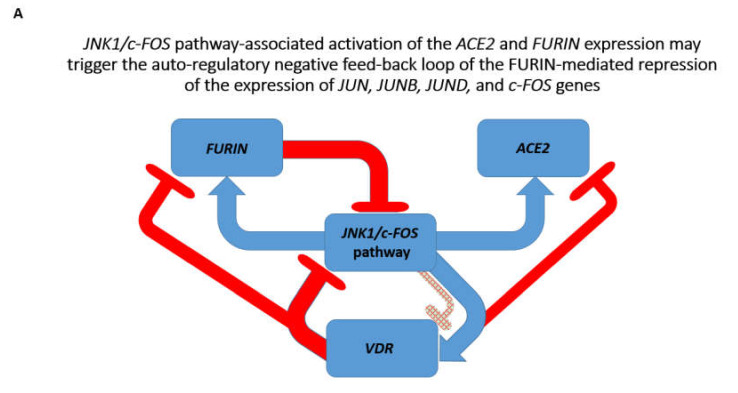

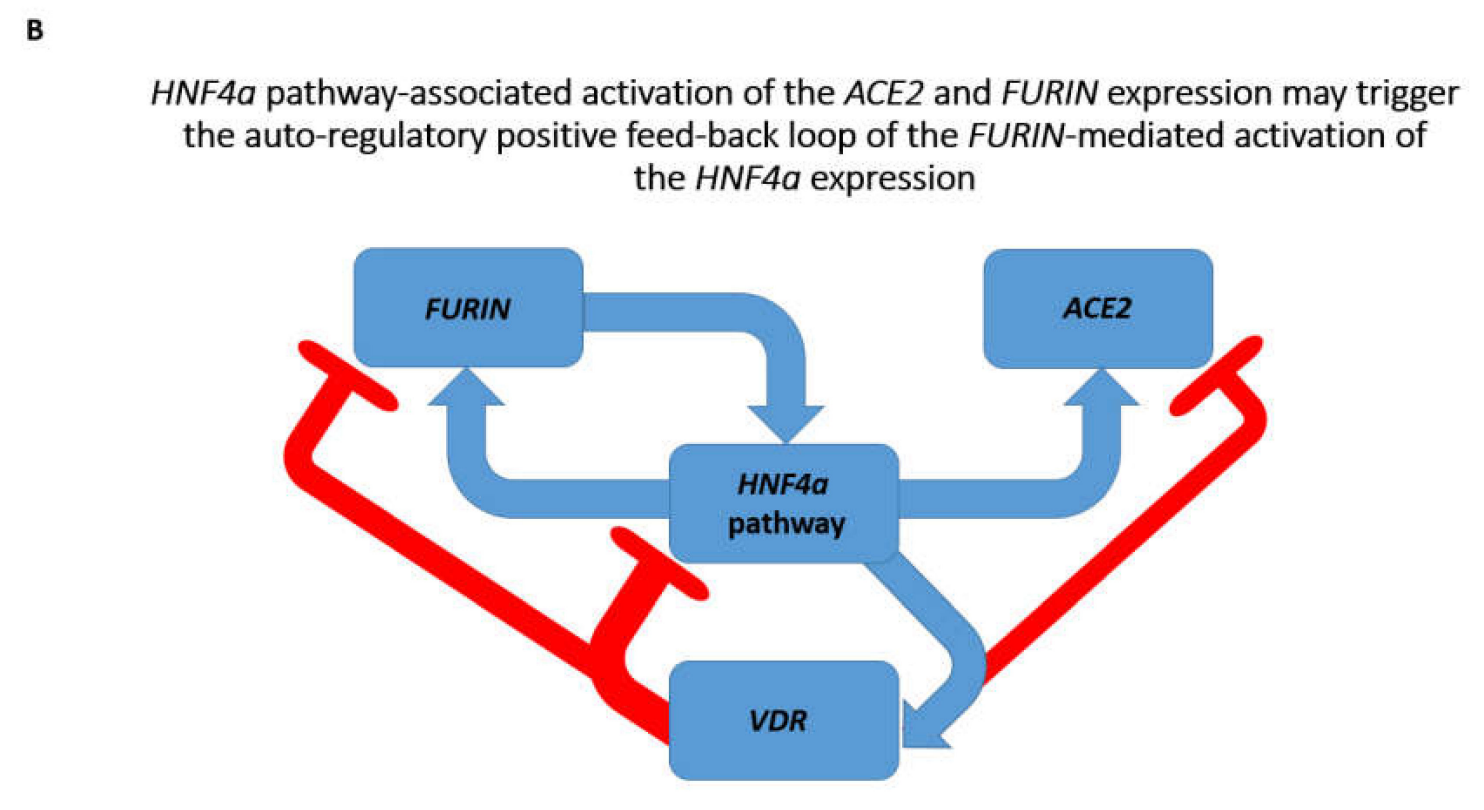
Effects of the *VDR* gene and vitamin D on pathways and genes affecting the newly emerged SARS-CoV-2 virus-related host targets. (**A**). *JNK1/c-FOS* pathway-associated activation of the *ACE2* and *FURIN* expression may trigger the auto-regulatory negative feed-back loop of the *FURIN*-mediated repression of the expression of *JUN, JUNB, JUND*, and *c-FOS* genes. (**B**). *HNF4a* pathway-associated activation of the *ACE2* and *FURIN* expression may trigger the auto-regulatory positive feed-back loop of the *FURIN*-mediated activation of the HNF4a expression. Activation of *VDR* gene would block *JNK1/c-FOS*- and *HNF4a*-mediated increased expression of both *ACE2* and *FURIN* genes.

Notably, examinations of direct and reciprocal effects of the *VDR* gene and vitamin D administration on expression of the *JNK1/c-FOS* pathway genes revealed expression profiles consistent with the potential therapeutic utility of the vitamin D administration and activation of *VDR* gene expression (Supplementary Figure S7). Analyses of direct and reciprocal effects of the *VDR* gene and vitamin D administration on *HNF4a* expression revealed that *HNF4a* depletion in human and murine cells inhibits *VDR* gene expression, while *Vdr* gene depletion increases *Hnf4a* expression (Supplementary Figure S7). These results are consistent with the hypothesis stating that vitamin D administration and activation of *VDR* gene expression may have mitigating effects on coronavirus infection. The summary of these findings is reported in Figure 3.

The GSEA of the transcription factor perturbations followed by the expression database and the GEO gene perturbations database focused on upregulated genes identified *HIF1a* and *POU5F1* gene products as putative repressors of the *ACE2* and *FURIN* expression (Supplementary Figure S8). These findings were corroborated by observations that *HIF1a* overexpression in human embryonic kidney cells significantly inhibits the *ACE2* expression (Supplementary Figure S8). Notably, vitamin D significantly increases expression of the *HIF1a* gene in human bronchial smooth muscle cells (Supplementary Figure S8), suggesting that *VDR* and *HIF1a* genes may cooperate as repressors of *ACE2* expression.

### 3.4. GSEA Identify Estradiol and Quercetin as Putative Candidate Coronavirus Infection Mitigation Agents

The GSEA of the drug perturbations from GEO database focused on downregulated genes identified estradiol and quercetin among the top significantly enriched records (Supplementary Figure S9). Estradiol appears to affect both *FURIN* and *ACE2* expression, while quercetin seems to target *ACE2* expression. Consistently, a GSEA of the ligand perturbations from GEO database focused on downregulated genes identified five estradiol administration records (50%) among the top ten significantly enriched ligand perturbations records (Supplementary Figure S9). The GSEA of the drug perturbations from the GEO database focused on upregulated genes indicated that doxorubicin, imatinib, and bleomycin may act as potential coronavirus infection-promoting agents (data not shown). Collectively, these observations provide the initial evidence supporting the hypothesis that both estradiol and quercetin may function as potential candidate coronavirus infection mitigation agents.

Consistent with this hypothesis, an interrogation of the GEO records revealed that quercetin appears to inhibit the expression of several potential coronavirus infection-promoting genes: *c-FOS* expression in human and rat cells (Supplementary Figure S9); *Runx1* expression in rat cells (Supplementary Figure S9); *HNF4a* expression in human cells (Supplementary Figure S9). However, quercetin administration appears to increase *c-Fos* expression in cultured rat cardiomyocytes (Supplementary Figure S9).

### 3.5. Confirmation of the Estradiol and Quercetin Activities as Potential Candidate Coronavirus Infection Mitigation Agents

The results of the GSEA suggest that both estradiol and quercetin appear to exhibit biological activities reflected in their gene expression signatures which are consistent with the activity of medicinal compounds expected to mitigate the coronavirus infection. Next, manual curation of the GEO data sets has been carried out to identify further experimental evidence supporting this hypothesis. The administration of estradiol appears to inhibit *ACE2* and/or *FURIN* expression in rat, mouse, and human cells (Supplementary Figure S10) and the effects of estradiol seem to be mediated by estrogen receptor beta. In agreement with the hypothesis on potential therapeutic utility of quercetin, the administration of quercetin has resulted in significantly decreased expression of the *ACE2* gene during the differentiation of human intestinal cells (Supplementary Figure S10).

### 3.6. Inference of Potential Interference of the Quercetin, Vitamin D, and Estradiol with Functions of SARS-CoV-2 Proteins in Human Cells

An excellent recent proteomics study of the SARS-CoV-2 interactome in human cells identified 332 high-confidence protein targets of the 27 SARS-CoV-2 viral proteins [19]. A GSEA of the 332 human genes encoding human prey proteins of the coronavirus SARS-CoV-2 revealed nuclear envelope disassembly, proteins targeting mitochondria, and tRNA and protein transports as major biological processes targeted by SARS-CoV-2 (Supplementary Figure S14). RNA and GDP-binding functions were identified as the main biological functions, while mitochondrion was identified as one of top-scoring cellular components targeted by SARS-CoV-2 infection (Supplementary Figure S14). It is reasonable to expect that candidate therapeutics that significantly alter the expression of human genes encoding prey proteins for SARS-CoV-2 may interfere with the viral replication by affecting the stoichiometry of viral protein/human prey protein interactions. Ultimately, the extent of therapeutics’ interference could be estimated by the numbers of affected human genes encoding prey proteins and the numbers of potentially affected viral proteins interacting with affected human prey proteins. Collectively, it may reflect the potential ability of candidate therapeutics to block functions of viral proteins essential for viral replication in human cells, thus blocking the propagation and spread of the infection in the human body.

**Figure 4:**
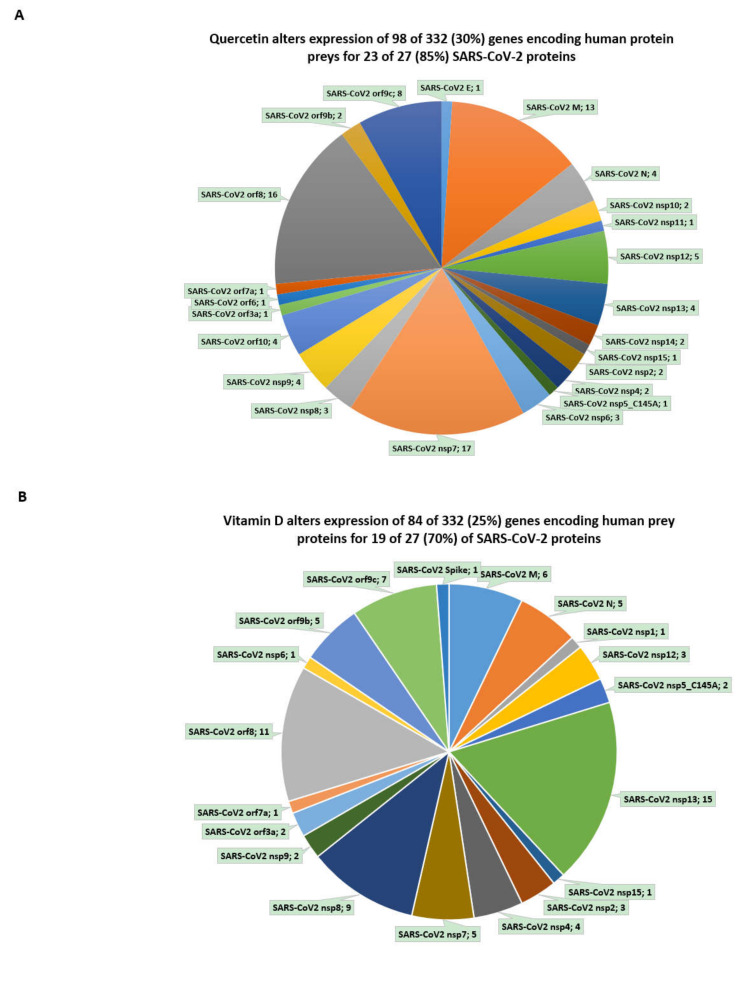

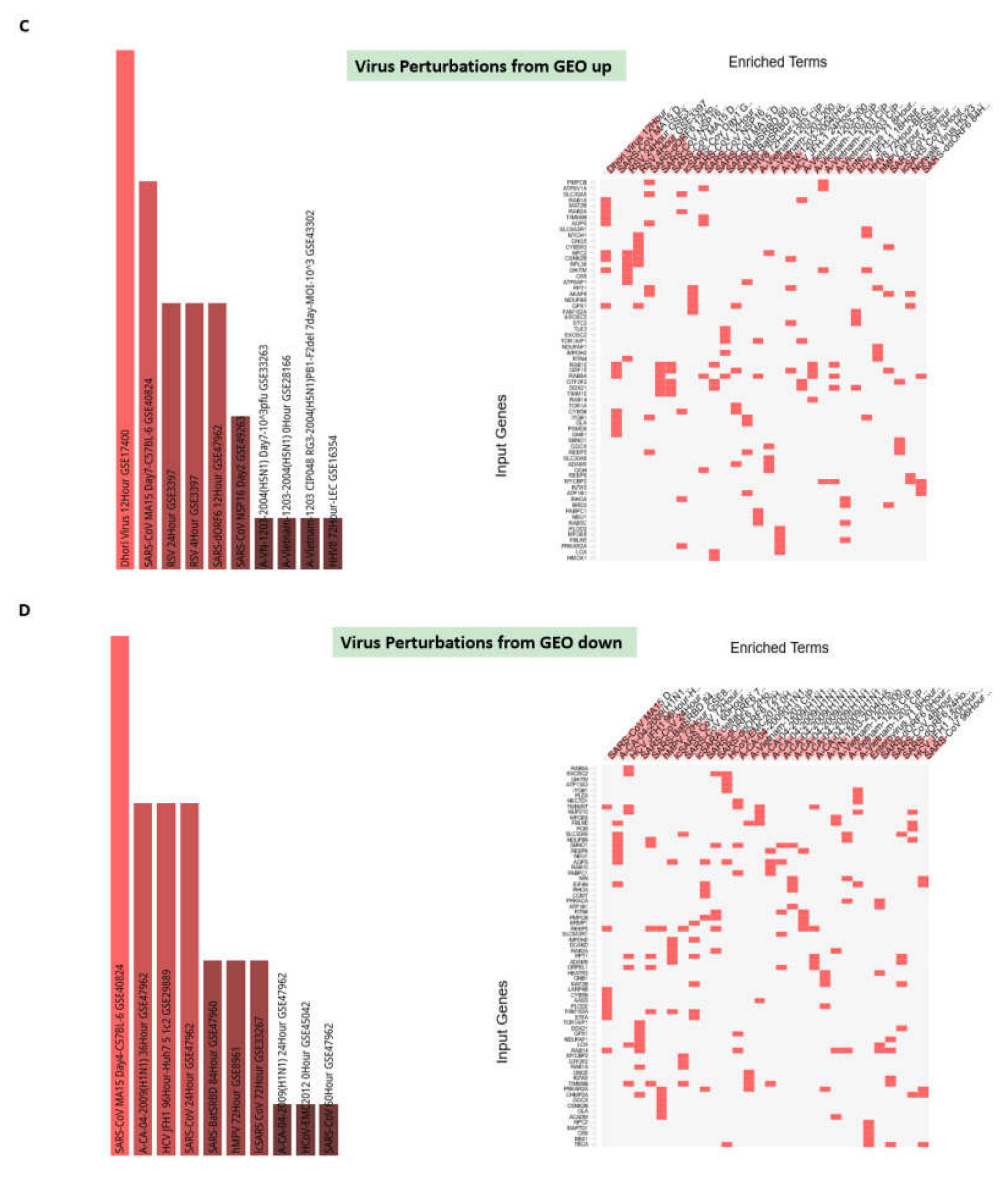
Effects of quercetin and vitamin D on expression of genes encoding human prey proteins of SARS-CoV-2. (**A**) Quercetin alters the expression of 98 of 332 (30%) of genes encoding human prey proteins for 23 of 27 (85%) of SARS-CoV-2 proteins. Percent values refer to the fractions of targets affected by quercetin based on gene expression profiling experiments. Both downregulated and upregulated genes were scored assuming that expression changes would alter the stoichiometry of viral protein/human prey protein interactions. Numerical values next to each viral protein names indicate the number of affected target protein-encoding genes. (**B**) Vitamin D alters the expression of 84 of 332 (25%) of genes encoding human prey proteins for 19 of 27 (70%) of SARS-CoV-2 proteins. Percent values refer to the fractions of targets affected by vitamin D based on gene expression profiling experiments. Both downregulated and upregulated genes were scored assuming that expression changes would alter the stoichiometry of viral protein/human prey protein interactions. Numerical values next to each viral protein names indicate the number of affected target protein-encoding genes. Panels C and D report the results of GSEA of the 332 genes in the Virus Perturbations from GEO database for upregulated (**C**) and downregulated (**D**) genes. See text and Supplementary Figure S14 for details.

Interestingly, quercetin alters the expression of 98 of 332 (30%) of genes encoding protein targets of SARS-CoV-2 in human cells, thus potentially interfering with the activities of 23 of 27 (85%) SARS-CoV-2 proteins (Figure 4; Supplementary Figure S14). Similarly, analyses of vitamin D-regulated genes identified in undifferentiated human THP-1 cells [20,21] revealed that vitamin D alters the expression of 84 of 332 (25%) human genes encoding prey proteins for SARS-CoV-2 (Figure 4; Supplementary Figure S14). These observations suggest that vitamin D, in addition to the inhibition of the expression of the *ACE2* and *FURIN* genes, may potentially interfere with the functions of 19 of 27 (70%) SARS-CoV-2 proteins (Figure 4; Supplementary Figure S14).

Gene expression-guided inference of the potential effects of a combination of both quercetin and vitamin D on functions of SARS-CoV-2 proteins suggests that together they may affect the activities of nearly all (25 of 27; 93%) SARS-CoV-2 viral proteins in human cells. It would be of interest and essential to test the validity of these predictions experimentally before definitive conclusions regarding the potential utility of a combination of quercetin and vitamin D as putative COVID-19 mitigation agents can be made.

**Figure 5:**
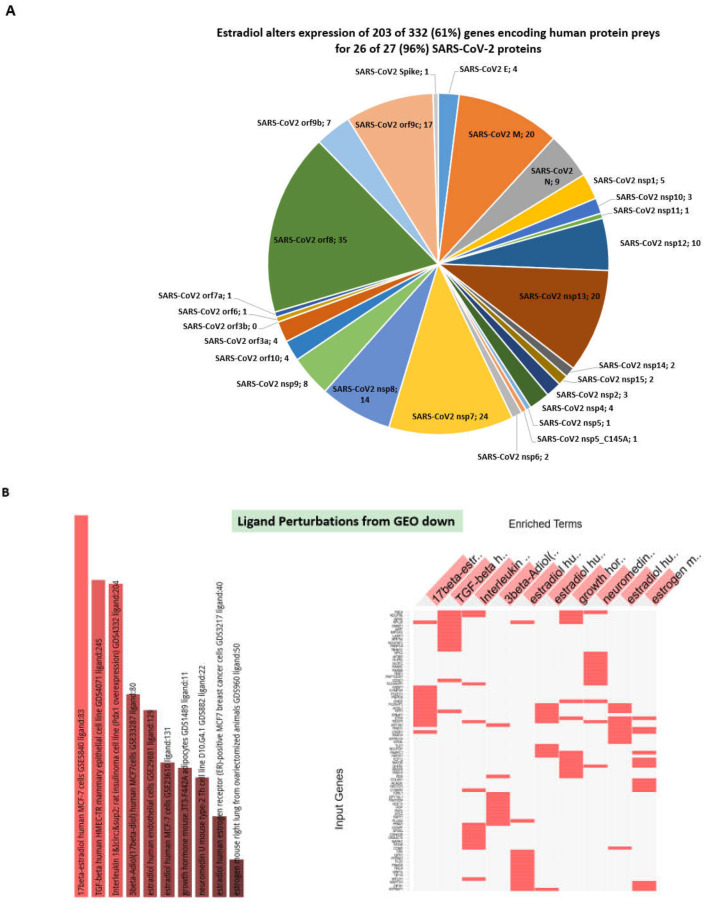

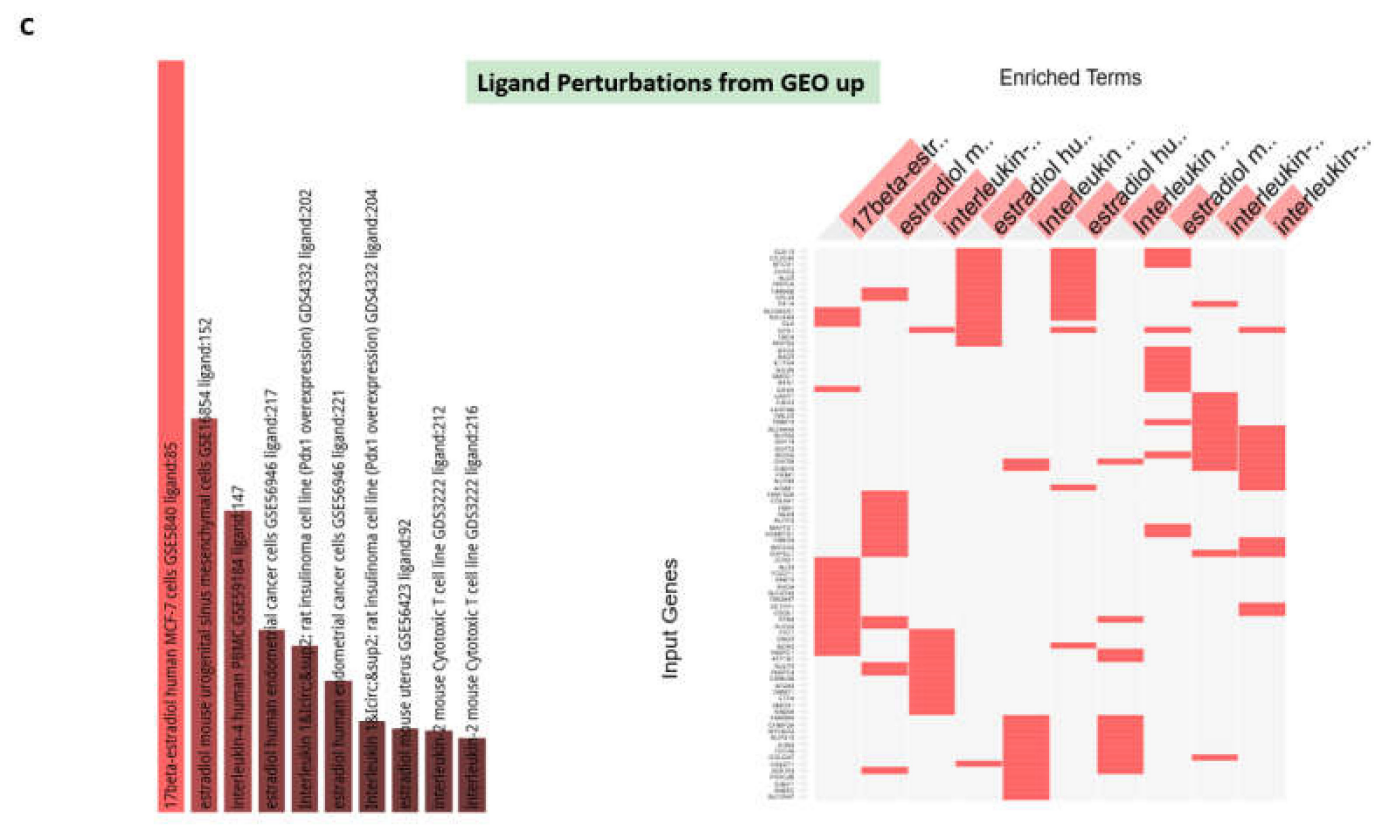
Effects of estradiol on expression of genes encoding human prey proteins of SARS-CoV-2. (**A**) Estradiol alters the expression of 203 of 332 (61%) of genes encoding human prey proteins for 26 of 27 (96%) of SARS-CoV-2 proteins. Percent values refer to the fractions of targets affected by the estradiol based on gene expression profiling experiments. Both downregulated and upregulated genes were scored assuming that expression changes would alter the stoichiometry of viral protein/human prey protein interactions. Numerical values next to each viral protein names indicate the number of affected target protein-encoding genes. Panels (**B**) and (**C**) reports the results of GSEA of the 332 genes in the Ligand Perturbations from GEO of downregulated (**B**) and upregulated (**C**) genes. Note marked representations of the estradiol records among top 10 significantly enriched entries of endogenous ligands.

Notably, estradiol manifests significant patterns of interference with the expression of genes encoding 332 prey proteins of SARS-CoV-2 in human cells (Figure 5; Supplementary Figure S14), while testosterone does not manifest significant associations (data not shown). Overall, estradiol affects the expression of 203 of 332 (61%) human genes encoding protein targets of SARS-CoV-2, thus potentially interfering with functions 26 of 27 (96%) SARS-CoV-2 viral proteins (Figure 5).

A hypothetical tripartite combination for the mitigation of the COVID-19 pandemic consisting of quercetin/vitamin D/estradiol may affect expression of 244 of 332 (73%) human genes encoding SARS-CoV-2 targets and interfere with the functions of all but one SARS-CoV-2 viral proteins (Table 1 and Table 2). Notably, the putative therapeutic benefits of the proposed bipartite (vitamin D and quercetin) and tripartite (quercetin/vitamin D/estradiol) combinations could be observed at different target selection thresholds ranging from 33% to 70% of genes potentially affected by corresponding treatment protocols, which were defined independently for each individual SARS-CoV-2 viral protein (Table 2).

However, estradiol administration appears to manifest cell type-specific effects on *c-FOS* expression (Supplementary Figure S10). For example, it decreases the *c-FOS* expression in the endometrium of Macaca mulatta, while it increases *c-FOS* expression in the mouse uterus (Supplementary Figure S10). The biological significance, if any, of these differences remains to be elucidated.

Collectively, these observations indicate that any definitive conclusions regarding the potential clinical utility of the herein identified potential coronavirus infection-mitigating agents should be made only after appropriately designed and carefully executed preclinical studies and randomized controlled clinical trials. In contrast to estradiol, which exhibits evidence of both putative coronavirus infection-mitigating actions and some coronavirus infection-promoting activities, the administration of testosterone appears to manifest more clearly defined patterns of altered gene expression consistent with testosterone being identified as the potential coronavirus infection-promoting agent (Supplementary Figure S11).

### 3.7. Potential Mechanisms Affecting Gene Expression Inferred from Transgenic Mouse Models and Observed in Pathophysiologically and Therapeutically Relevant Mouse and Human Cells

**Figure 6:**
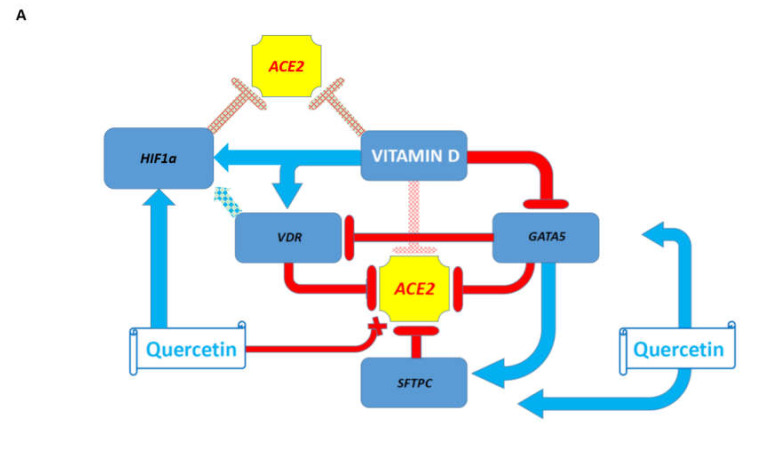

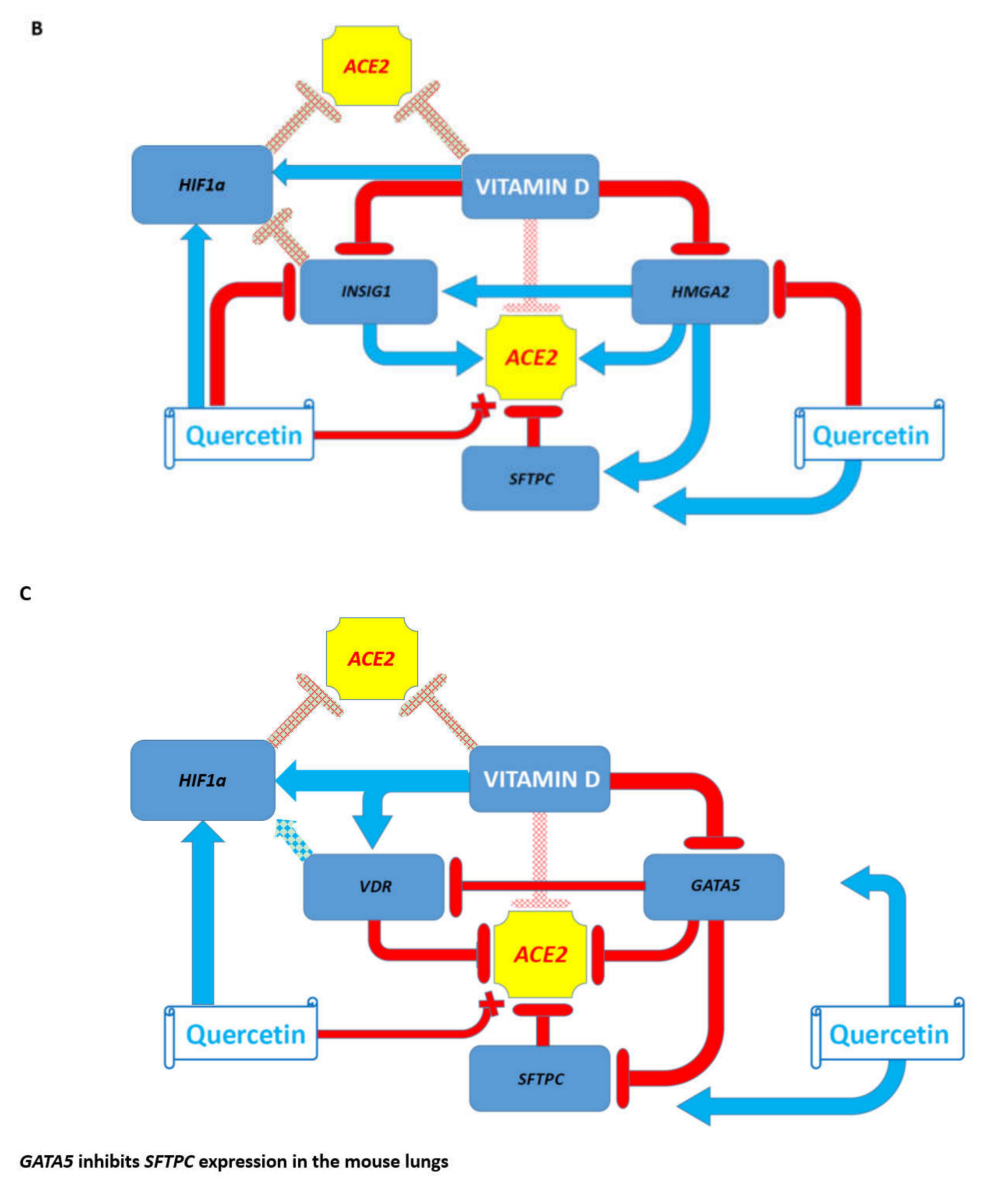

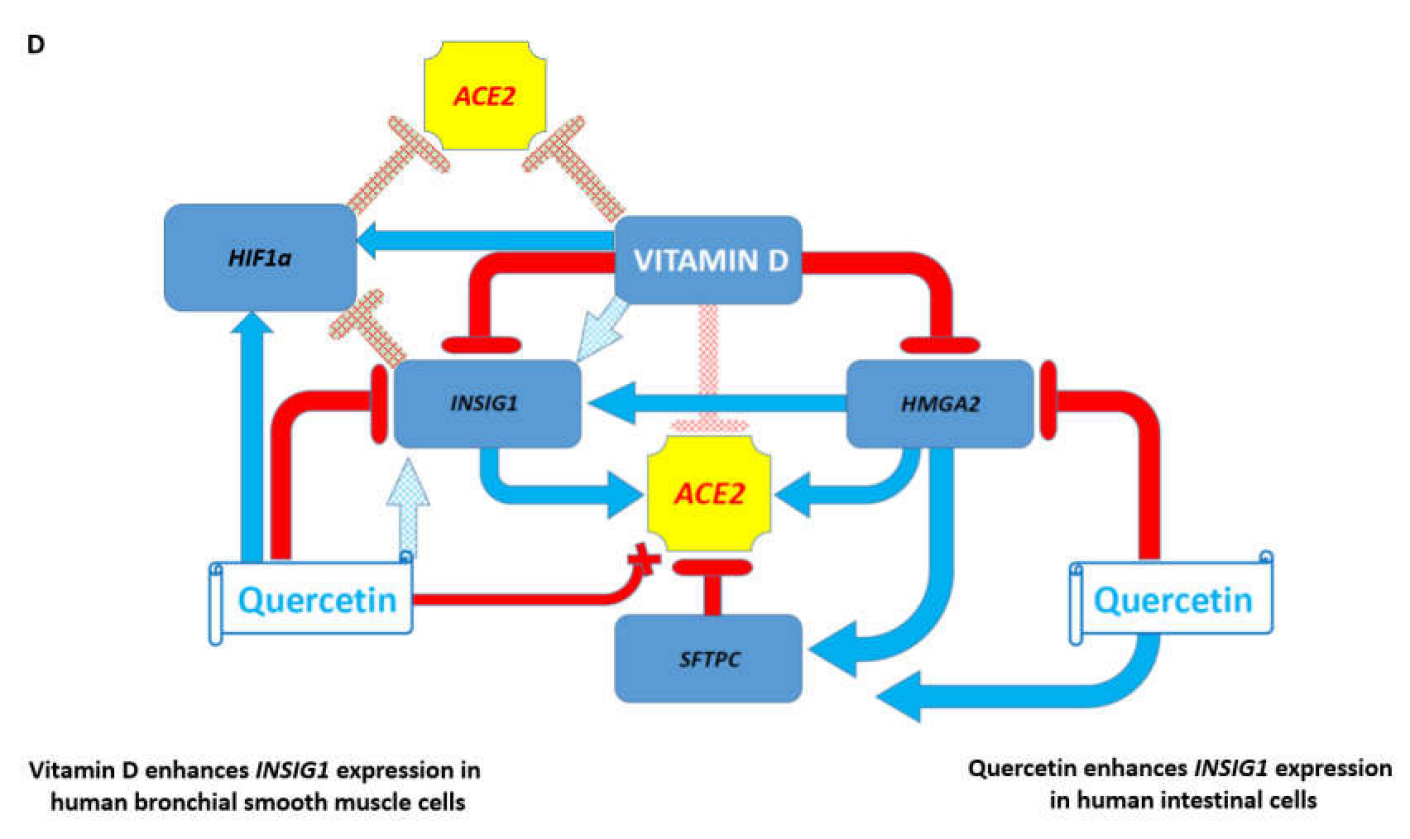
Effects of the *VDR* gene, vitamin D, and quercetin on pathways and genes affecting the newly emerged SARS-CoV-2 virus-related host targets. (**A**) Effects of the *VDR* gene, vitamin D, and quercetin on repressors of the *ACE* expression. (**B**). Effects of the *VDR* gene, vitamin D, and quercetin on activators of the *ACE* expression. (**C**). Effects of the *VDR* gene, vitamin D, and quercetin on repressors of the *ACE* expression reflecting *GATA5* inhibitory effects on *SFTPC* expression in the mouse lungs. (**D**). Effects of the *VDR* gene, vitamin D, and quercetin on activators of the *ACE* expression reflecting the cell type-specific effects of vitamin D and quercetin: vitamin D-induced activation of the *INSIG1* expression in human bronchial smooth muscle cells and quercetin-induced activation of the *INSIG1* expression in human intestinal cells.

Taking into considerations that the effects of potential coronavirus infection mitigation agents often manifest cell type-specific patterns of gene expression changes, next, the manual curation of GEO gene expression profiles was carried out to identify the relevant host genetic targets and putative mitigation agents. These analyses were focused on several candidate repressors (*VDR; GATA5; SFTC; HIF1a*) and activators (*INSIG1; HMGA2*) of the *ACE2* and *FURIN* expression (Supplementary Figure S12). Notably, the effects on gene expression of the administration of either vitamin D or quercetin appear consistent with their definition as putative coronavirus infection mitigation agents (Supplementary Figure S12). A summary of these observations is presented in Figure 6. The conclusion regarding the findings of cell type-specific effects on gene expression of putative coronavirus infection-mitigating agents remains valid and examples of the potential negative effects of drugs on *ACE2* expression are reported in Supplementary Figure S12. For example, *HIF1a* expression is significantly increased in murine alveolar type I cells deficient in sterol response element-binding protein inhibitor *INSIG1* (Supplementary Figure S12). These data indicate that the *INSIG1* gene product, which appears to function as an activator of *ACE2* expression, may function as an inhibitor of *HIF1a* expression, thus interfering with *HIF1a*-mediated *ACE2* repression in specific cell types. Additional examples of the potential positive and negative effects of gene expression changes inferred from transgenic mouse models are reported in Supplementary Figure S13.

### 3.8. Is Vitamin D Deficiency a Potential Risk Factor for Increased Disease Severity in Older Adults and Elderly Individuals?

Present analyses suggest that vitamin D and vitamin D receptor (VDR) are putative mitigation factors of the coronavirus infection. Conversely, vitamin D deficiency could be a potential aggravating factor for the clinical course of the pandemic. Multiple lines of evidence suggest that vitamin D deficiency, particularly in the elderly, might be a negative factor affecting the clinical course of the pandemic. In the United States, approximately 30% of whites and 5% of African Americans have sufficient vitamin D levels [22] and the significant increase in the prevalence of individuals with severe vitamin D deficiency has been reported [23]. The age-associated decline in the human skin’s ability to produce vitamin D in response to sunlight exposure is likely a contributing factor to the vitamin D deficiency in older individuals, since it has been reported that elderly people produce 75% less cutaneous vitamin D3 than young individuals [24]. A meta-analysis of randomized controlled clinical trials indicated that the intake of ordinary doses of vitamin D was associated with a significant decrease in total mortality rates [25]. A prospective study of 3408 older adults in the United States demonstrated that a group at high risk of all-cause mortality could be defined by the serum 25-hydroxyvitamin D [25(OH)D] level [26]. A significant, independent, inverse association was observed between the serum 25(OH)D level and all-cause and cardiovascular disease (CVD) mortality [26].

To date, the majority of observational studies reported inverse associations between the circulating 25(OH)D concentration and all-cause mortality in generally healthy populations [27]. In generally healthy adults over 50 years old, significant inverse associations were found between low 25(OH)D levels and all-cause mortality, respiratory and cardiovascular events, as well as markers relating to hip and non-vertebral fractures [28]. Therefore, it would be important to determine whether vitamin D deficiency may be one of the risk factors contributing to the increased disease severity in older adults and elderly individuals during the coronavirus pandemic. Significantly, recent studies have highlighted numerous beneficial clinical effects of vitamin D supplementations [29,30,31] and underscore the significant COVID-19 mitigation potential of vitamin D. Finally, an investigation into whether dramatic variations in the severity of clinical manifestations of the COVID-19 pandemic in various countries are linked to vitamin D deficiency has revealed that the risk of severe COVID-19 is significantly higher among patients with severe vitamin D deficiency [32].

### 3.9. Clinical and Experimental Observations Appear Highly Compatible with the Results of Genomic-Guided Mapping of COVID-19 Targets in Human Cells

Genomic-guided tracing of SARS-CoV-2 targets in human cells revealed very unusual, perhaps, unique, features of this virus. One of these features is highlighted by discordant patterns of testosterone versus estradiol impacts on SARS-CoV-2 targets, suggesting a plausible molecular explanation of the apparently higher male mortality during the coronavirus pandemic. These findings are in agreement with several observations regarding discordant effects of androgens and estrogens described previously for coronavirus infection. It has been noted [33] that Transmembrane Protease, Serine 2 (TMPRSS2), the protease contributing to coronavirus infectivity, is an androgen-expressed protein, indicating that SARS-CoV-2 infection is likely to be androgen-mediated. In mice, males appear more vulnerable to the coronavirus infection, while females manifested resistance and ovariectomy increased their susceptibility to infection [34]. Despite the fact that both *TMPRSS2* and the virus receptor *ACE2* are expressed in male and female lungs (this study; [35], males and females may benefit from different therapeutic strategies: the prescription of vitamin D and quercetin may be useful for both sexes, while additional estrogen supplementation could be beneficial for males. Overall, the results of the present analyses are consistent with the hypothesis that men and older women may benefit from short-term estrogen supplementation to alleviate the clinical course of the COVID-19 pandemic.

One major concern noted in this study is the *ACE2* and *FURIN* expression in many human cells and tissues, including immune and endothelial cells, suggesting that SARS-CoV-2 may infect a broad range of cellular targets in the human body. Consistent with this hypothesis, clinical observations indicate that patients hospitalized with COVID-19 in the King County (Washington), Wuhan (China), and the New York City area exhibit a very broad range of clinical manifestations of the disease and co-morbidities which are not limited to the upper respiratory tract infection and pneumonia [36,37,38]. Presence of SARS-CoV-2 virus was reported in neural and capillary endothelial cells in frontal lobe tissue obtained at postmortem examination from a COVID-19 patient who suffered from worsening neurologic symptoms [39], thus documenting the direct targeting of the human brain by SARS-CoV-2 infection.

Results of the present analyses are consistent with the hypothesis that infection of immune cells by SARS-CoV-2 may cause immunosuppression, long-term persistence of the virus, and spread of the virus to secondary targets. It has been reported that human endothelial cells express principal cellular targets bound by SARS-CoV-2 to gain entry into host cells, including ACE2, CD147, sialic acid receptor, and TMPRSS2 [40]. Therefore, a putative path to the disease’s spread and broad pathological impacts on human body is highlighted by the potential susceptibility to infection of endothelial cells denoted in the present study and underscored in the previous work implicating the endothelial dysfunction in the pathogenesis of COVID-19 [40,41], including cardiac injury [38] and thrombotic damage [36,41].

### 3.10. Limitations

Several shortcomings of present analyses must be considered as limitations of this study. The features, records, and traits reported in this study are high scoring in the relative ranks of hundreds of thousands of analyzed features, records, and traits included in specific databases. However, in several instances when the top-scoring candidate genomic features and/or traits were selected and reported, statistical significance was achieved only based on nominal *p* values. This was primarily due to the relatively small sample size available for the interrogation, which highlights another common limitation of the available records. Where possible, attempts to address these shortcomings were made by analyzing multiple independent data sets to arrive at the consensus. One of the important limitations is the small number of studies conducted on human cells, in particular, human cells and tissues pathophysiologically relevant to the COVID-19 pandemic. Collectively, these considerations underscore the critical need for follow-up targeted experimental studies and, if warranted, randomized clinical trials to identify and validate the utility of quercetin and vitamin D as therapeutically viable interventions to combat the COVID-19 pandemic.

## 4. Conclusions

The main motivation of this work was to identify human genes implicated in regulatory cross talks affecting expression and functions of the *ACE2* and *FURIN* genes to build a model of genomic regulatory interactions potentially affecting SARS-CoV-2 infection. A panel of genes acting as activators and/or repressors of the *ACE2* and/or *FURIN* expression then could be employed to search for existing drugs and medicinal substances that, based on their mechanisms of action, could be defined as candidate coronavirus infection mitigation agents. After experimental and clinical validation, these existing drugs and/or medicinal agents could be utilized to ameliorate the clinical severity of the pandemic. This knowledge could also be exploited in an ongoing effort to discover novel targeted therapeutics tailored to prevent SARS-CoV-2 infection and block the entry of the virus into human cells. Observations reported in this contribution are in agreement with recent studies describing numerous beneficial clinical effects of the vitamin D supplementations, emphasizing many detrimental effects of the vitamin D insufficiency and deficiency, and underscoring the significant COVID-19 mitigation potential of vitamin D [29,30]. Importantly, two recent interventional randomized clinical trials aiming to evaluate effects of vitamin D on the prevention and treatment of COVID-19 were listed on the ClinicalTrials.gov website (https://www.clinicaltrials.gov/ct2/show/NCT04334005 and https://clinicaltrials.gov/ct2/show/NCT04344041).

One of the important findings documented herein is that identified medicinal compounds with potential coronavirus infection-mitigating effects also appear to induce cell type-specific patterns of gene expression alterations. Therefore, based on all observations reported in this study, it has been concluded that any definitive recommendations regarding the potential clinical utility of the herein identified putative coronavirus infection-mitigating agents, namely vitamin D, quercetin, and estradiol, should be made only after preclinical studies and randomized controlled clinical trials have been appropriately designed, carefully executed, and the desired outcomes have been reached.

**Figure 7:**
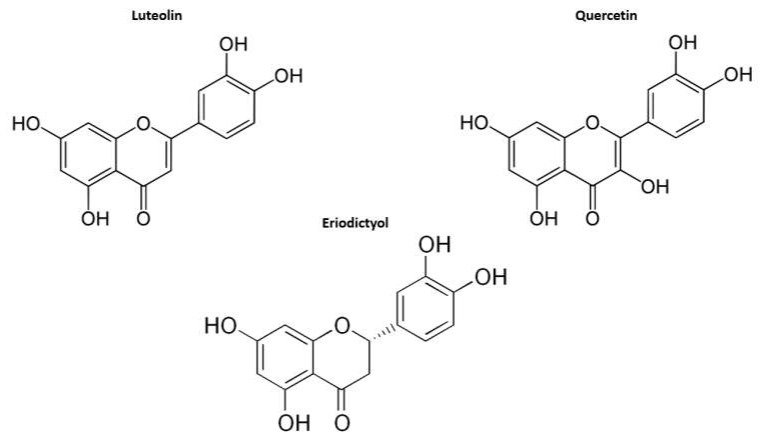
Similarity between chemical structures of the luteolin, quercetin, and eriodictyol. Luteolin, quercetin, and eriodictyol have been identified in the recent supercomputer SUMMIT drug-docking screen as top candidate inhibitors of the SARS-CoV-2 spike-protein–human ACE2 receptor interface–ligand binding complex [42], while Luteolin and quercetin have been identified as potent inhibitors of the SARS-CoV infection [43].

A supercomputer modeling study using the world’s most powerful supercomputer, SUMMIT, identified several candidate small molecule drugs which bind to either the isolated SARS-CoV-2 Viral S-protein at its host receptor region or to the S protein-human ACE2 interface [42]. Interestingly, in this study, quercetin was identified among the top five scoring ligands for viral S-protein-human ACE2 receptor interface. Thus, quercetin also appears to be a potentially promising therapeutic molecule that may directly interfere with the binding of SARS-CoV-2 to human cells. Previously reported experiments demonstrated that quercetin appears to inhibit SARS-CoV entry into host cells [43]. Since SARS-CoV-2 utilizes, for the entry into human cells, the same receptor (ACE2) and the accessory protease FURIN as the SARS-CoV coronavirus [3], these observations suggest that quercetin may, indeed, possess antiviral activity against SARS-CoV-2 as well. Significantly, both quercetin and luteolin have been identified among the top five ligands for the viral S-protein–human ACE2 receptor interface–ligand-binding complex [42], suggesting that these highly structurally similar compounds (Figure 7) could serve as efficient inhibitors of SARS-CoV-2 infection. Consistent with this hypothesis, it has been reported that both quercetin and luteolin significantly inhibit the SARS-CoV virus infection [43].

It has been observed that administration of testosterone appears to manifest clearly defined patterns of altered gene expression, consistent with testosterone being identified as the potential coronavirus infection-promoting agent, particularly in some cell types that may play a role in the virus entry into the human body and the respiratory system (Supplementary Figure S11). This is in contrast to estradiol, which seems to manifest complex cell type-specific effects on gene expression consistent with either infection-inhibiting or infection-promoting patterns of gene expression changes. It would be of interest to determine whether these discordant effects may contribute to the apparently higher mortality among men with coronavirus infection. In line with present analyses, a randomized interventional clinical trial entitled “Phase II Clinical Trial of estradiol to Reduce Severity of COVID19 Infection in COVID19+ and Presumptive COVID19+ Patients” has been posted on ClinicalTrials.gov with the start date April 20, 2020 and completion date November 20, 2020 (https://clinicaltrials.gov/ct2/show/NCT04359329). Interestingly, a hypothetical tripartite combination of pandemic mitigation agents consisting of vitamin D/quercetin/estradiol may affect the expression of 244 out of 332 (73%) human genes encoding SARS-CoV-2 targets (Table 1 and Table 2). It has been observed that both bipartite (vitamin D and quercetin) and tripartite (vitamin D/quercetin/estradiol) combinations of candidate pandemic mitigation agents manifest statistically more robust effects on expression of SARS-CoV-2 target genes compared to monotherapies (Table 1 and Table 2). These differences were documented for comparisons of either the numbers of affected human genes encoding SARS-CoV-2 targets (Table 1) or the numbers of SARS-CoV-2 viral proteins presumed to be functionally blocked by the prospective therapeutics (Table 2).

The present analyses highlight the major uncertainty regarding the outcomes of the current pandemic associated with the potential of SARS-CoV-2 in the expansion of the cellular tropism [3] based on its access to genetically vulnerable host cells due to the nearly ubiquitous expression of the *ACE2* and *FURIN* genes in the human body. A particular dangerous factor that must be noted in this contribution is the potential ability of SARS-CoV-2 to infect the immune cells, because the infection of immune cells may cause immunosuppression, the long-term persistence of the virus in the body, and the spread of the virus to secondary targets.

It has been reported that both SARS-CoV and SARS-CoV-2 utilize the ACE2 as the entry receptor to infect human cells [3]. The coronaviruses SARS-CoV and SARS-CoV-2 have comparable binding affinities, achieved by balancing the energetics and dynamics of interactions with the ACE2 receptor [44]. However, the SARS-CoV-2–ACE2 complex contains a higher number of contacts, a larger interface area, and decreased interface residue fluctuations compared to the SARS-CoV-ACE2 complex [44], suggesting a markedly distinct and more efficient strategy for SARS-CoV-2 interactions with the human ACE2 receptor. It seems remarkable to attribute this level of adaptation toward the highly efficient human receptor recognition of the natural evolutionary exploration of coronaviruses in non-human hosts.

Taken together with predominantly cell type-specific patterns of expression of genetic repressors and activators of the *ACE2* and *FURIN* expression, this may complicate the development of universally effective therapeutics. The availability of many genetically relevant transgenic mouse models—in particular, FURIN-null mice—should be regarded as a considerable advantage for the preclinical development of drug candidates tailored to target the coronavirus infection. Specifically, the potential therapeutic utility of the highly selective (Ki, 600 pm) and intrinsically specific FURIN inhibitor (a1-antitrypsin Portland (a1-PDX); [45]) should be tested in the immediate future.

**Figure 8:**
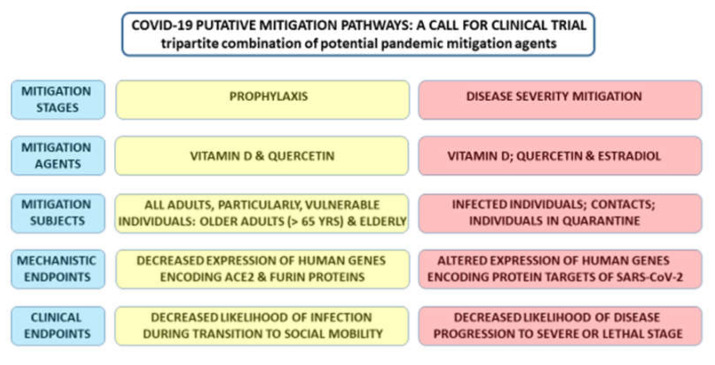
Proposed mitigation approaches for prophylaxis and treatment of the COVID-19 pandemic based on prescriptions of bipartite (vitamin D and quercetin) and tripartite (vitamin D/quercetin/estradiol) combinations of candidate therapeutics.

**Figure A1:**
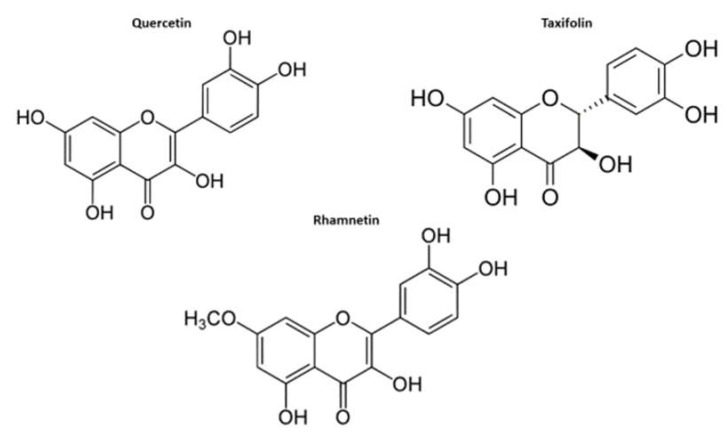
Similarity of chemical structures of the Quercetin, Taxifolin, and Rhemnetin. Taxifolin and rhemnetin have been identified in the recent a chemical library of over 687 million compounds for binding at the recently solved crystal structure of the main protease of SARS-CoV-2 coronavirus (Fischer et al., 2020. https://doi.org/10.26434/chemrxiv.11923239.v2 ) among the top-scoring candidate inhibitors of the SARS-CoV-2 main protease.

It has been noted that observations and conclusions derived from the genomic-guided tracing of SARS-CoV-2 targets in human cells appear logistically compatible with relevant experimental and clinical observations. Importantly, observations reported in this contribution seem to provide a mechanistic basis for a better understanding of SARS-CoV-2′s biology and pathophysiology, as well as a diverse spectrum of clinical manifestations of the COVID-19 pandemic, including the potential to inflict the extensive damage to the endothelium, brain, and the cardio-vascular system. Collectively, these findings and conclusions strongly support the feasibility of the proposed bipartite and tripartite combinations of candidate pandemic mitigation agents for the prophylaxis and treatment of COVID-19 (Figure 8). It is considered a highly encouraging factor that clinical trials have been announced for all three candidate pandemic mitigation agents identified in this study, albeit aiming to evaluate their effectiveness as a monotherapy for the COVID-19 pandemic. Additionally, see the note added in Appendix (Figure A1).

## Figures and Tables

**Table 1:** Number of drug-specific and common human genes encoding protein targets of SARS-CoV-2, whose expression is altered by vitamin D, quercetin, and estradiol.

Therapeutic Modality	Number of Affected Genes	Percent of All SARS-CoV-2 Targets *
Vitamin D (all affected genes)	84 **	25.30
Quercetin (all affected genes)	98 **	29.52
Estradiol (all affected genes)	203 **	61.14
Vitamin D and Estradiol	32	9.64
Vitamin D and Quercetin	6	1.81
Quercetin and Estradiol	57	17.17
Vitamin D/Quercetin/Estradiol	22	6.63
Estradiol only	90	27.11
Quercetin only	13	3.92
Vitamin D only	24	7.23
All SARS-CoV-2 targets affected by Vitamin D/Quercetin/Estradiol	244 **	73.49

Legend: * the percent of affected targets was estimated based on a total number of 332 human proteins identified as targets of 27 SARS-CoV-2 viral proteins. **, a tripartite combination (vitamin D; quercetin; estradiol) affected the expression of a significantly larger number of genes compared to monotherapies (*p* < 0.05; Fisher’s exact test).

**Table 2:** Number of drug-specific and common SARS-CoV-2 viral proteins with predicted interference by vitamin D, quercetin, and estradiol treatments.

Therapeutic Modality	Number of Affected SARS-CoV-2 Proteins (33% Targets’ Threshold)	Percent of All SARS-CoV-2 Proteins (*n* = 27)	Number of Affected SARS-CoV-2 Proteins (50% Targets’ Threshold)	Percent of All SARS-CoV-2 Proteins (*n* = 27)	Number of Affected SARS-CoV-2 Proteins (70% Targets’ Threshold)	Percent of All SARS-CoV-2 Proteins (*n* = 27)
Vitamin D (all affected targets)	10	37.04	4	14.81	1	3.70
Quercetin (all affected targets)	12	44.44	6	22.22	2	7.41
Vitamin D and Quercetin	23*	85.19	11	40.74	3	11.11
Estradiol (all affected targets)	26	96.30	23	85.19	5	18.52
Vitamin D/ Quercetin/Estradiol	26	96.30	24	88.89	14 *	51.85
Testosterone	8	29.63	4	14.81	1	3.70

Legend: * denotes statistically significant increase in targets affected by bipartite (vitamin D and quercetin) and tripartite (vitamin D; quercetin; estradiol) compared with the most effective monotherapy (*p* < 0.05; Fisher’s exact test). Thresholds of affected targets were defined based on numbers of genes, the expression of which was altered by specified agents and total numbers of human genes encoding protein targets for each of the 27 SARS-CoV-2 viral proteins. Note the markedly contrasting and significantly different effects of estradiol and testosterone.

## Supplementary Materials

Supplementary materials can be found at https://www.mdpi.com/2227-9059/8/5/129/s1.

## Appendix A

In the comprehensive exploration of the chemical space to date, a computational screening of a chemical library of over 687 million compounds for binding at the recently solved crystal structure of the main protease of SARS-CoV-2 coronavirus (Fischer A, Sellner M, Neranjan S, Lill MA, Smieško M. Potential Inhibitors for Novel Coronavirus Protease Identified by Virtual Screening of 606 Million Compounds. 2020. https://doi.org/10.26434/chemrxiv.11923239.v2) identified two highly similar to Quercetin molecules, namely taxifolin and rhamnetin, among the top-scoring candidate inhibitors of the SARS-CoV-2 main protease (Figure A1).
