# Supplementary material for: Tripartite Combination of Candidate Pandemic Mitigation Agents: Vitamin D, Quercetin, and Estradiol Manifest Properties of Medicinal Agents for Targeted Mitigation of the COVID-19 Pandemic Defined by Genomics-Guided Tracing of SARS-CoV-2 Targets in Human Cells
Source: Biomedicines. 2020 May 21;8(5):129. doi: 10.3390/biomedicines8050129 (PMC7277789; doi:10.3390/biomedicines8050129)
Supplement: Supplementary file 1 [file biomedicines-08-00129-s001.pdf]

ACE2 and FURIN

Supplemental Figure S1. Expression in human cells, tissues, and organs

RNA-Seq Expression Data from GTEx (53 Tissues, 570 Donors)

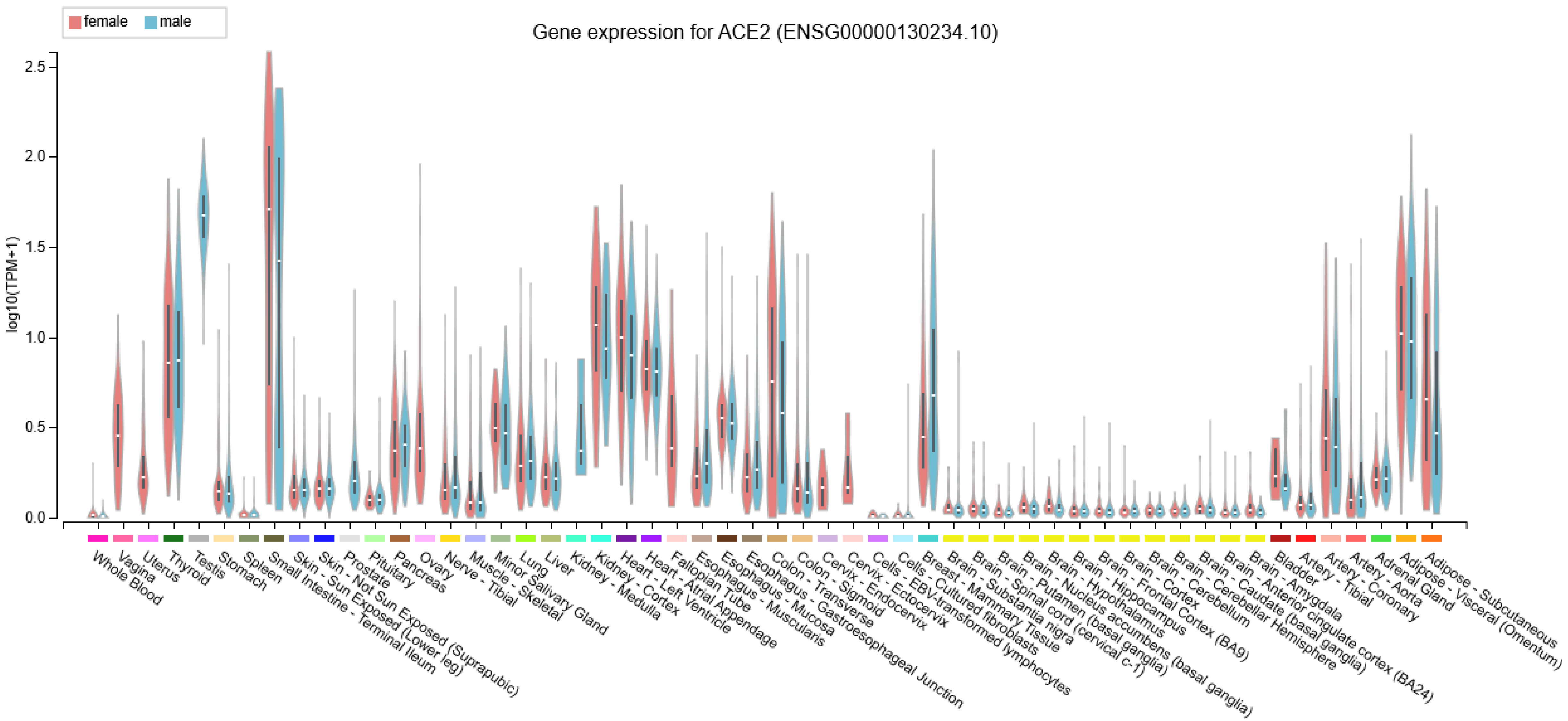

RNA-Seq Expression Data from GTEx (53 Tissues, 570 Donors)

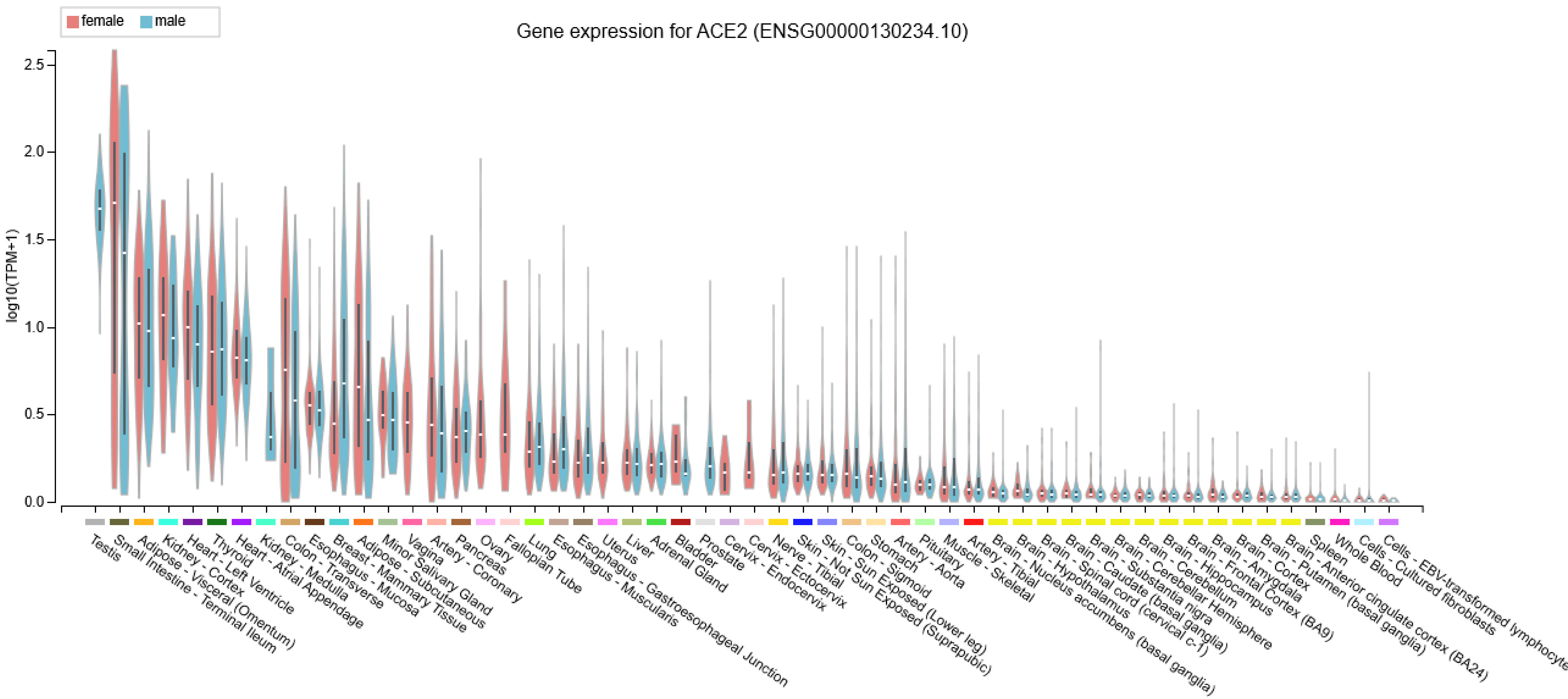

RNA-Seq Expression Data from GTEx (53 Tissues, 570 Donors)

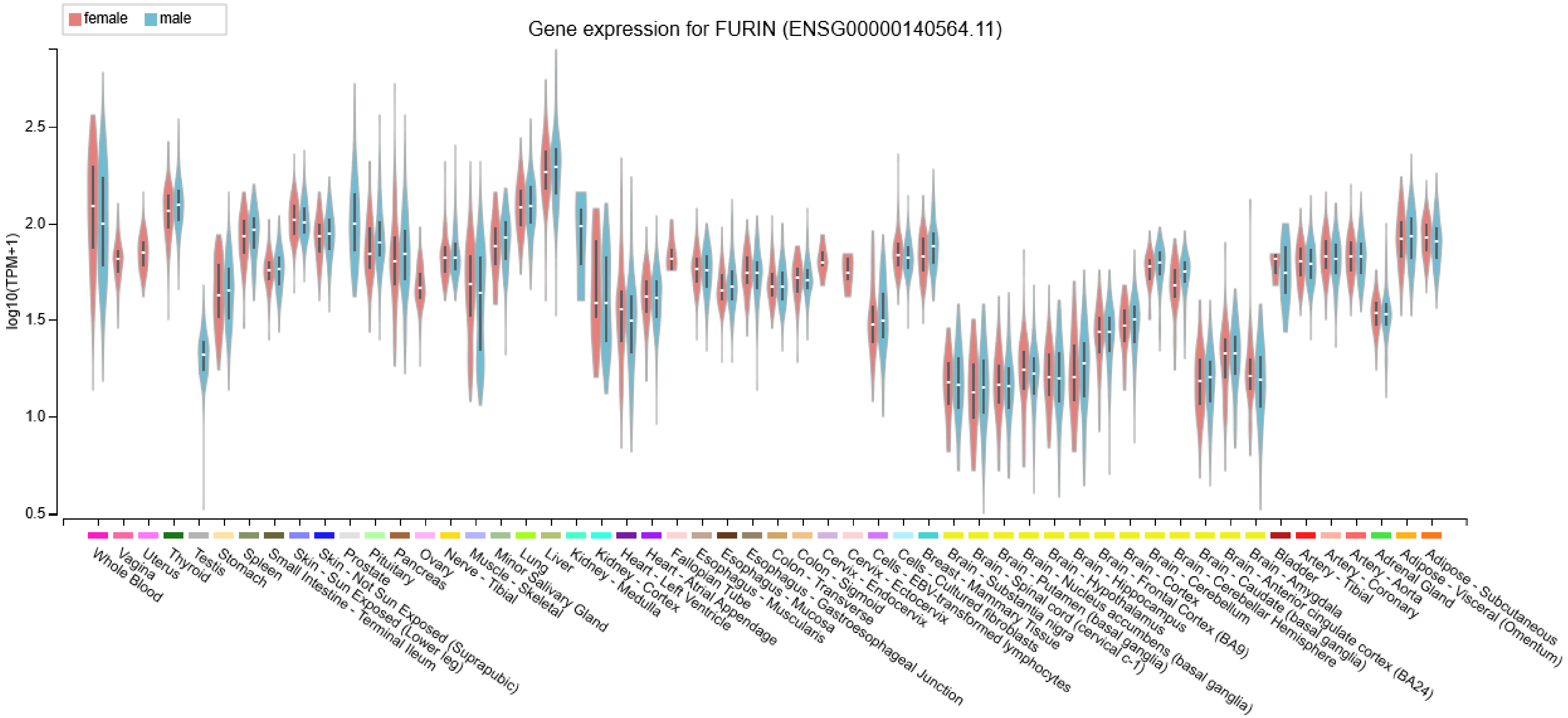

RNA-Seq Expression Data from GTEx (53 Tissues, 570 Donors)

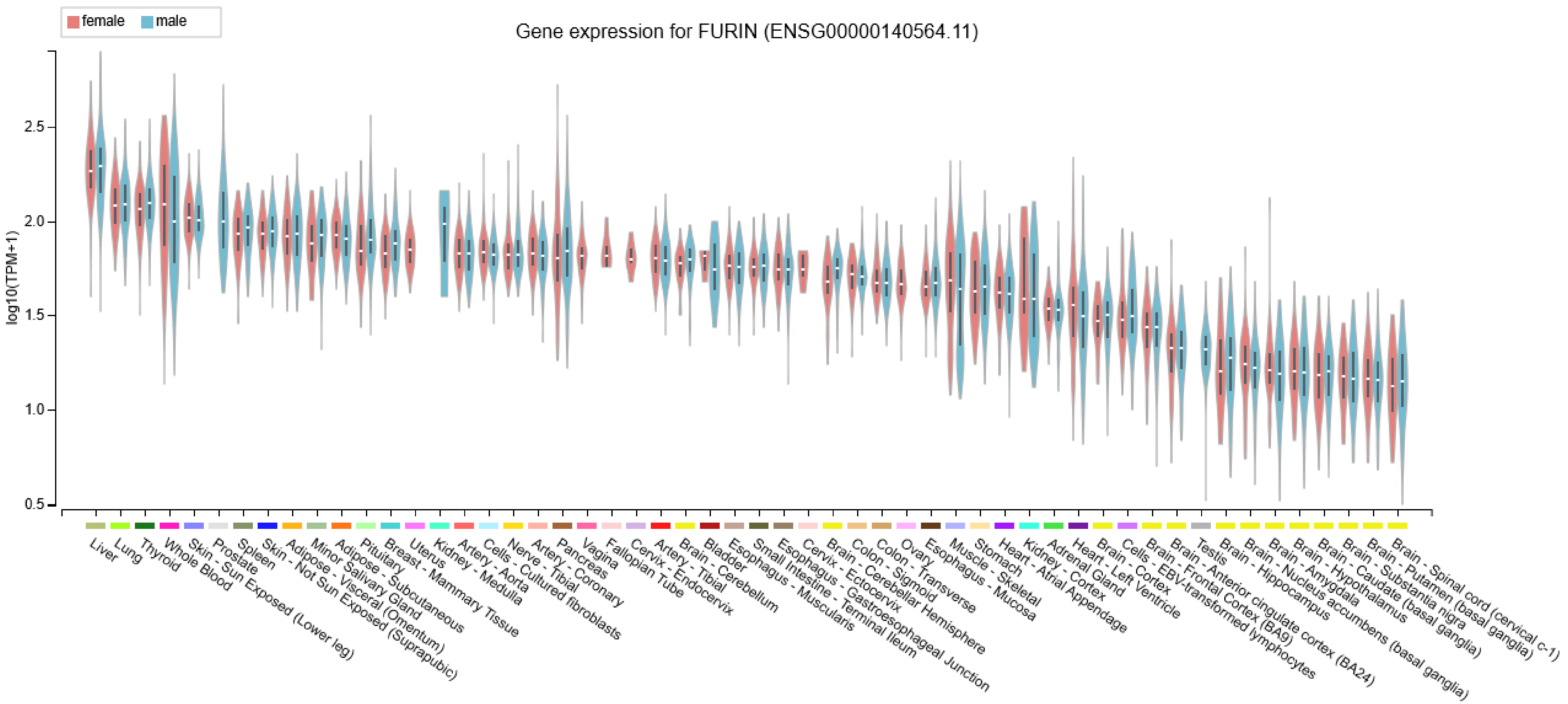

Jensen TISSUES

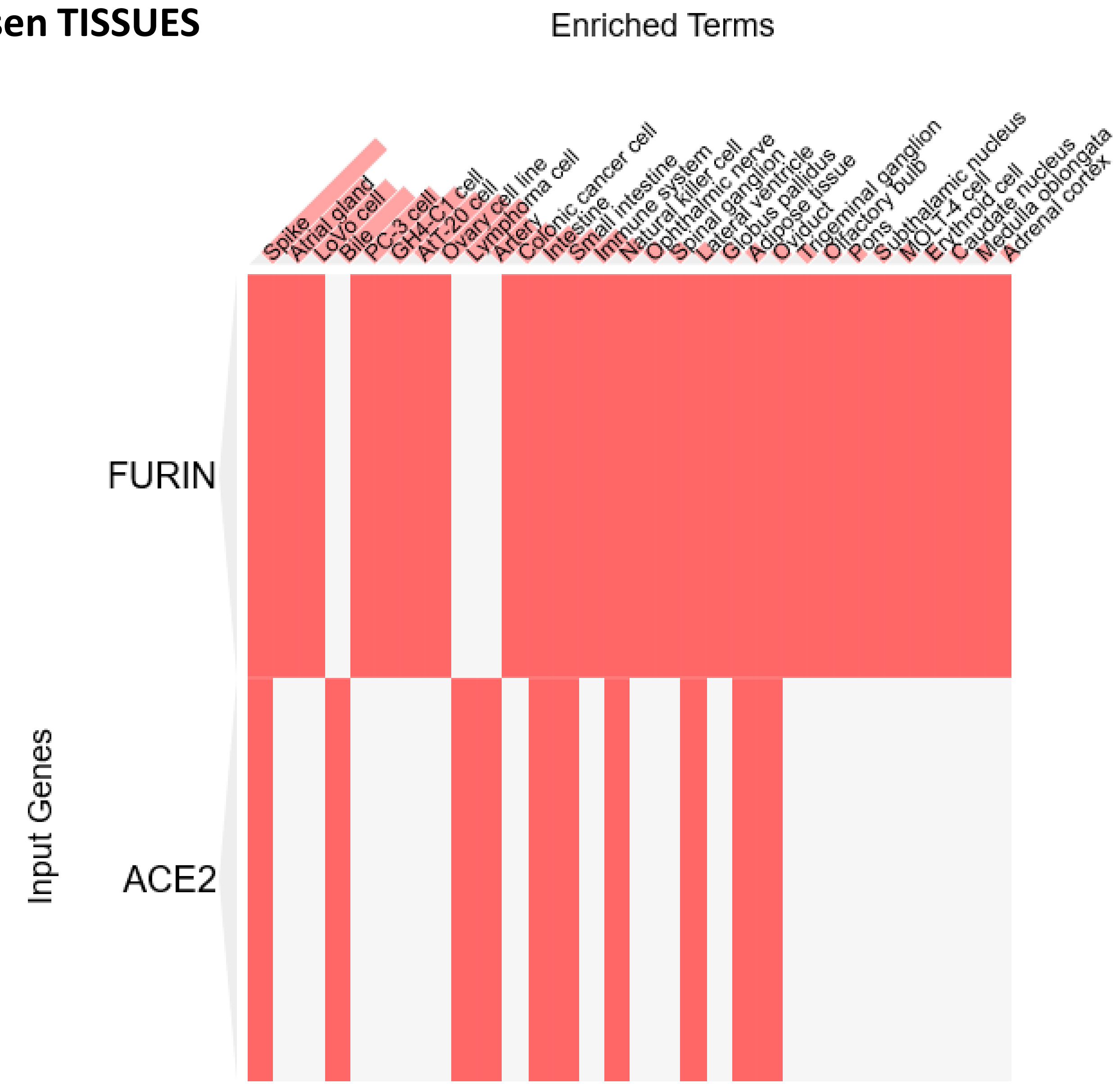

ARCHS4 Human Tissues

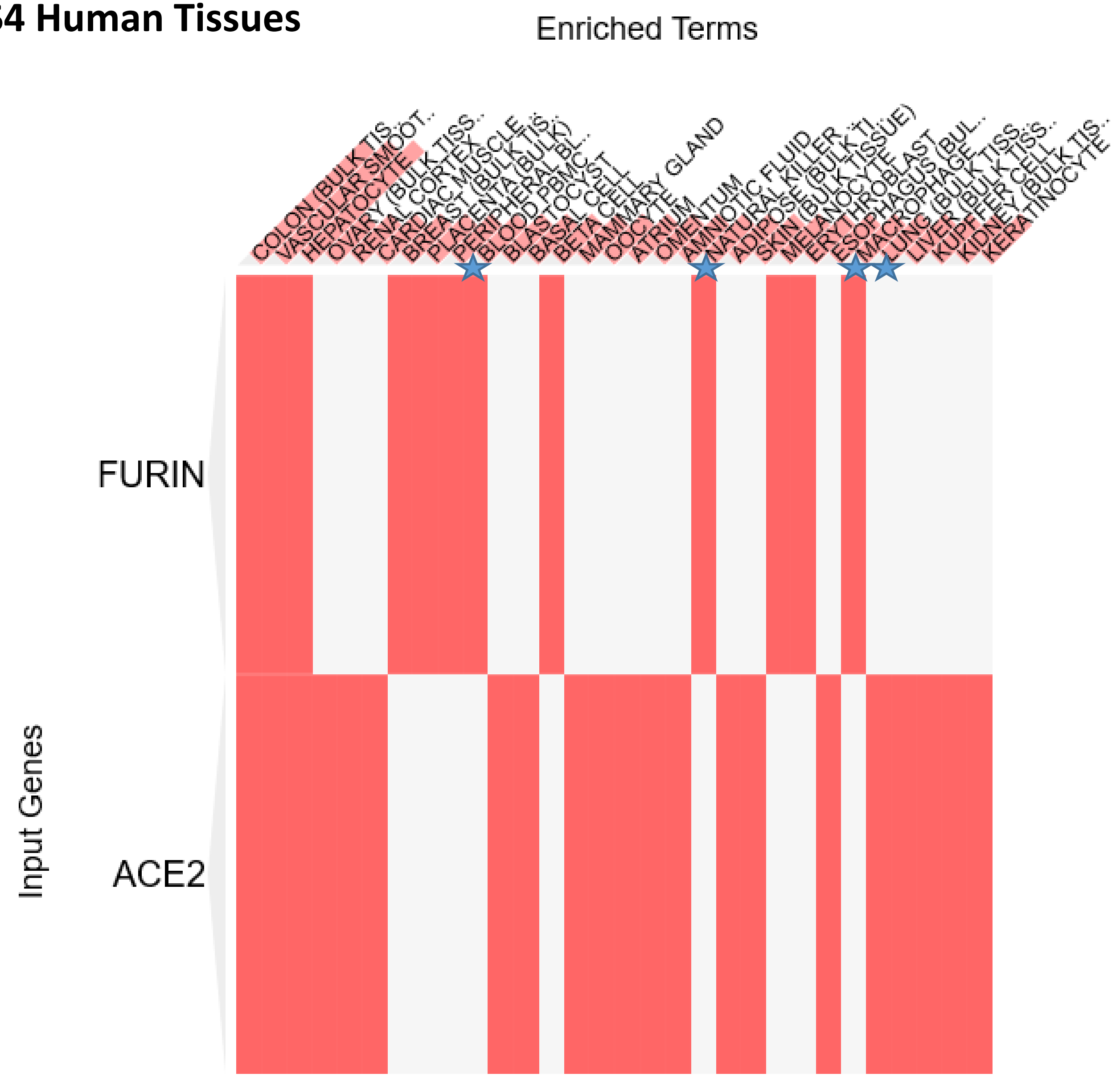

ACE2 and FURIN

Supplemental Figure S2. Effects of viral challenges on expression

Virus Perturbations from GEO up

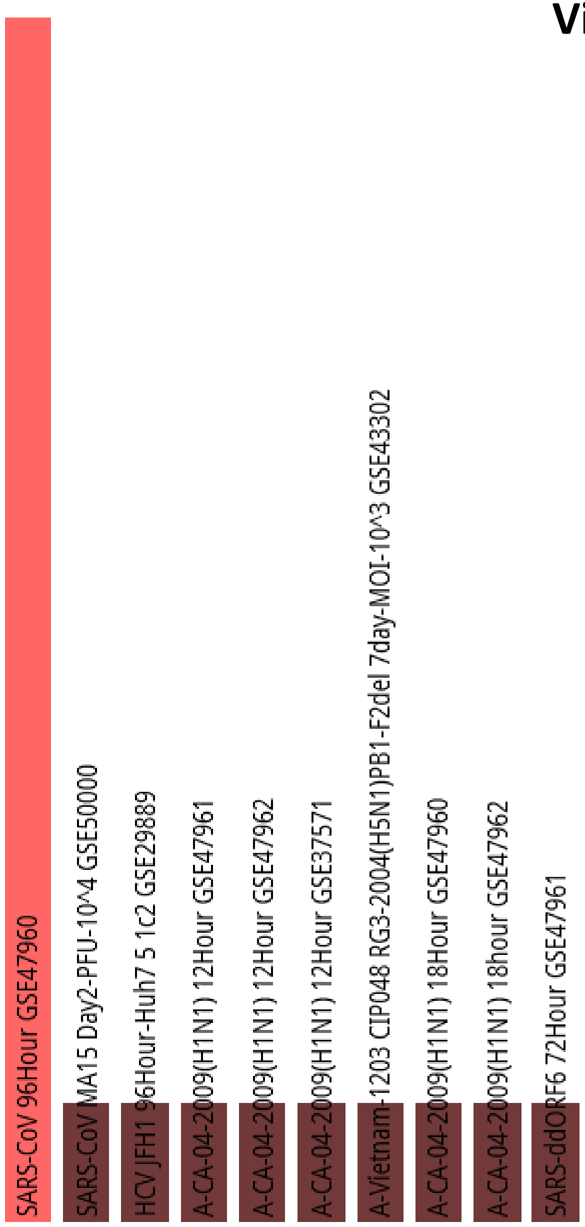

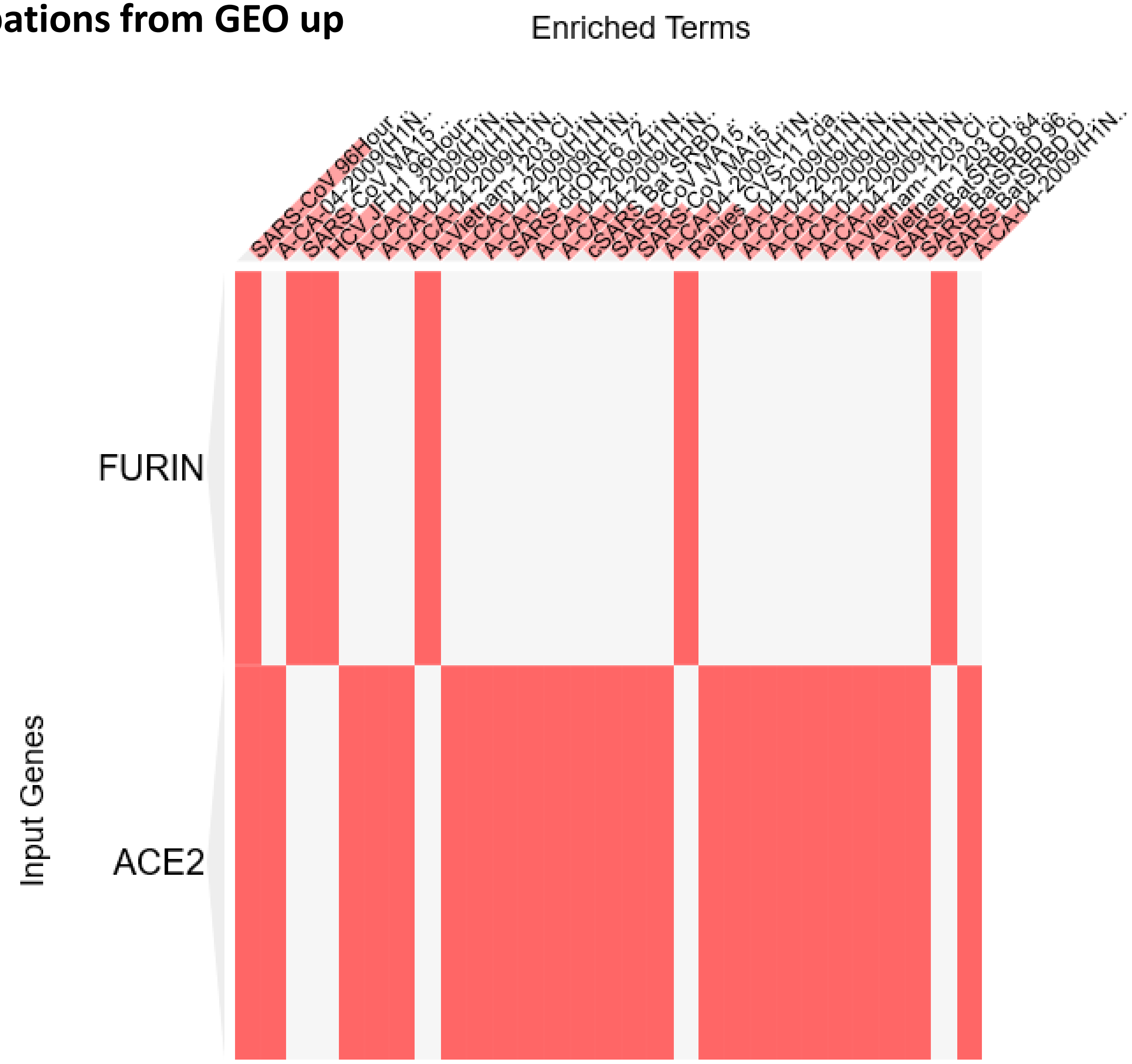

Profile: *FURIN* expression in peripheral blood mononuclear cells (PBMCs)
GDS1028 / 201945_at
Title
Severe acute respiratory syndrome expression profile
Organism
Homo sapiens

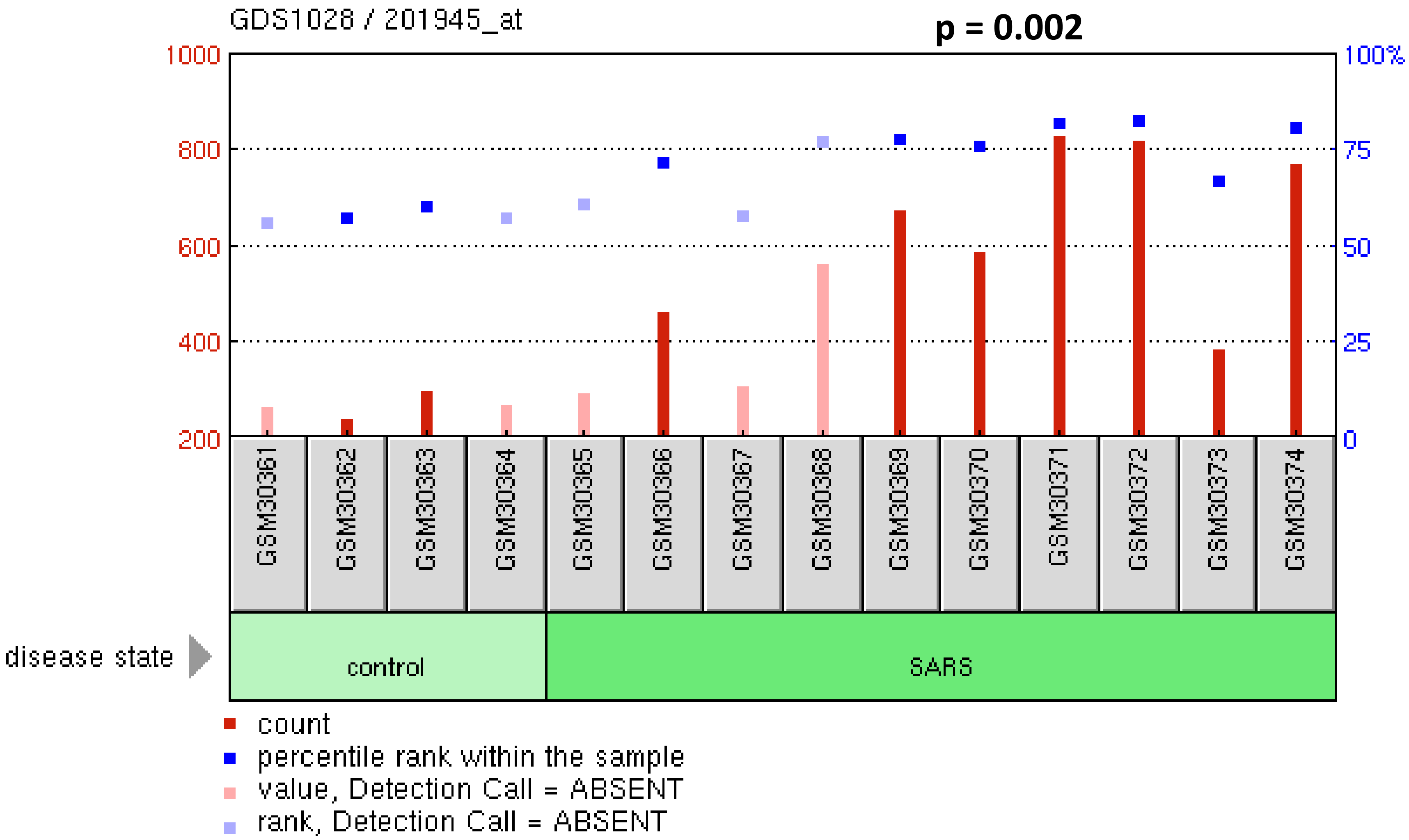

FURIN expression in peripheral blood mononuclear cells (PBMCs)

Sample	Title	Value
GSM30361	N1	264.2
GSM30362	N2	241.7
GSM30363	N3	298.1
GSM30364	N4	268.5
GSM30365	S1	295.5
GSM30366	S2	464
GSM30367	S3	309.7
GSM30368	S4	564.1
GSM30369	S5	674.4
GSM30370	S6	588
GSM30371	S7	830.2
GSM30372	S8	818.8
GSM30373	S9	385.1
GSM30374	S10	771.2

ACE2 and FURIN

Supplemental Figure S3. Effects of common human diseases on expression changes

Distinct patterns of diseases associated with increased expression of the *ACE2* and *FURIN* genes

Disease Perturbations from GEO up

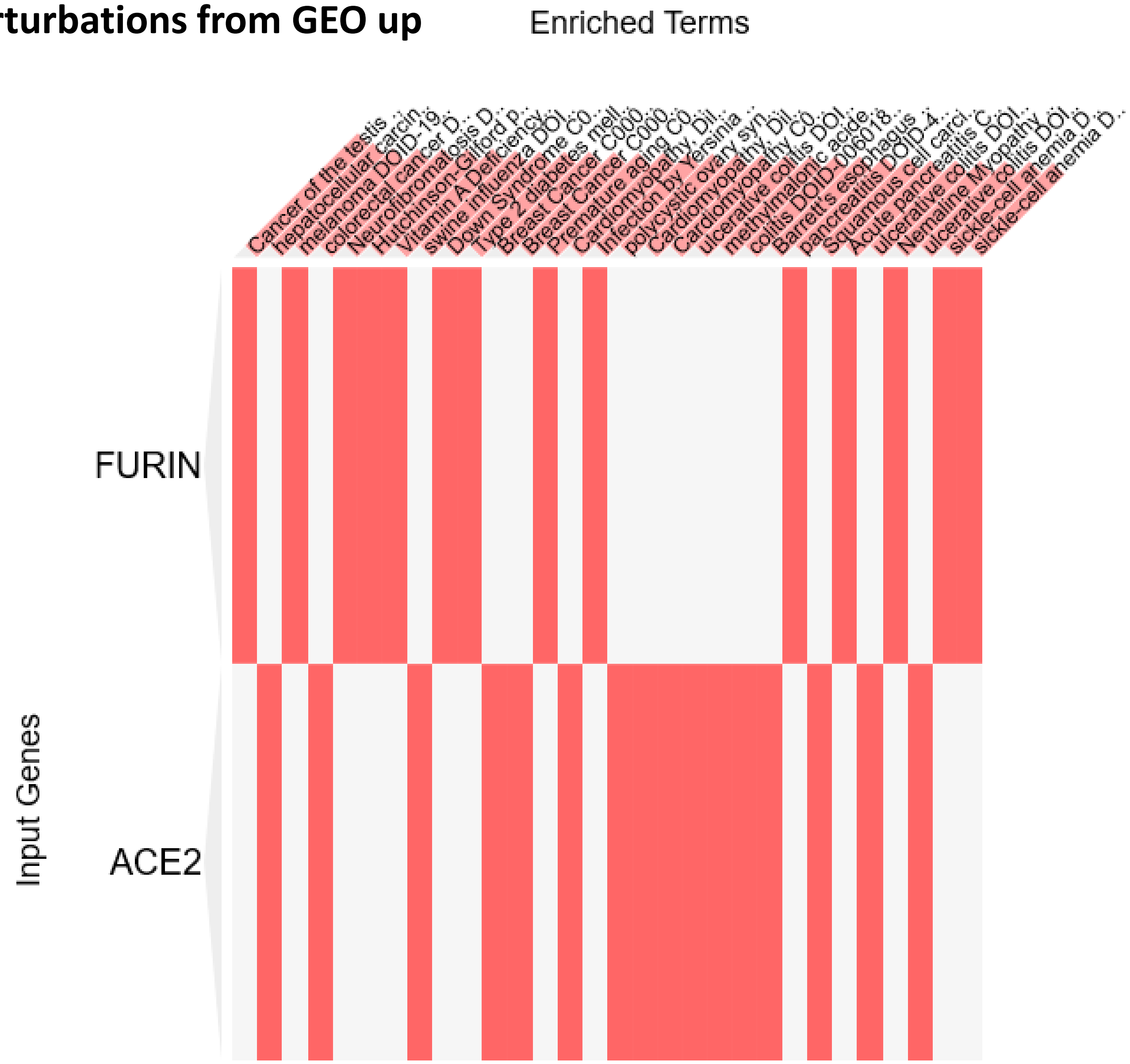

Distinct patterns of diseases associated with expression changes of the *ACE2* and *FURIN* genes

DisGeNET

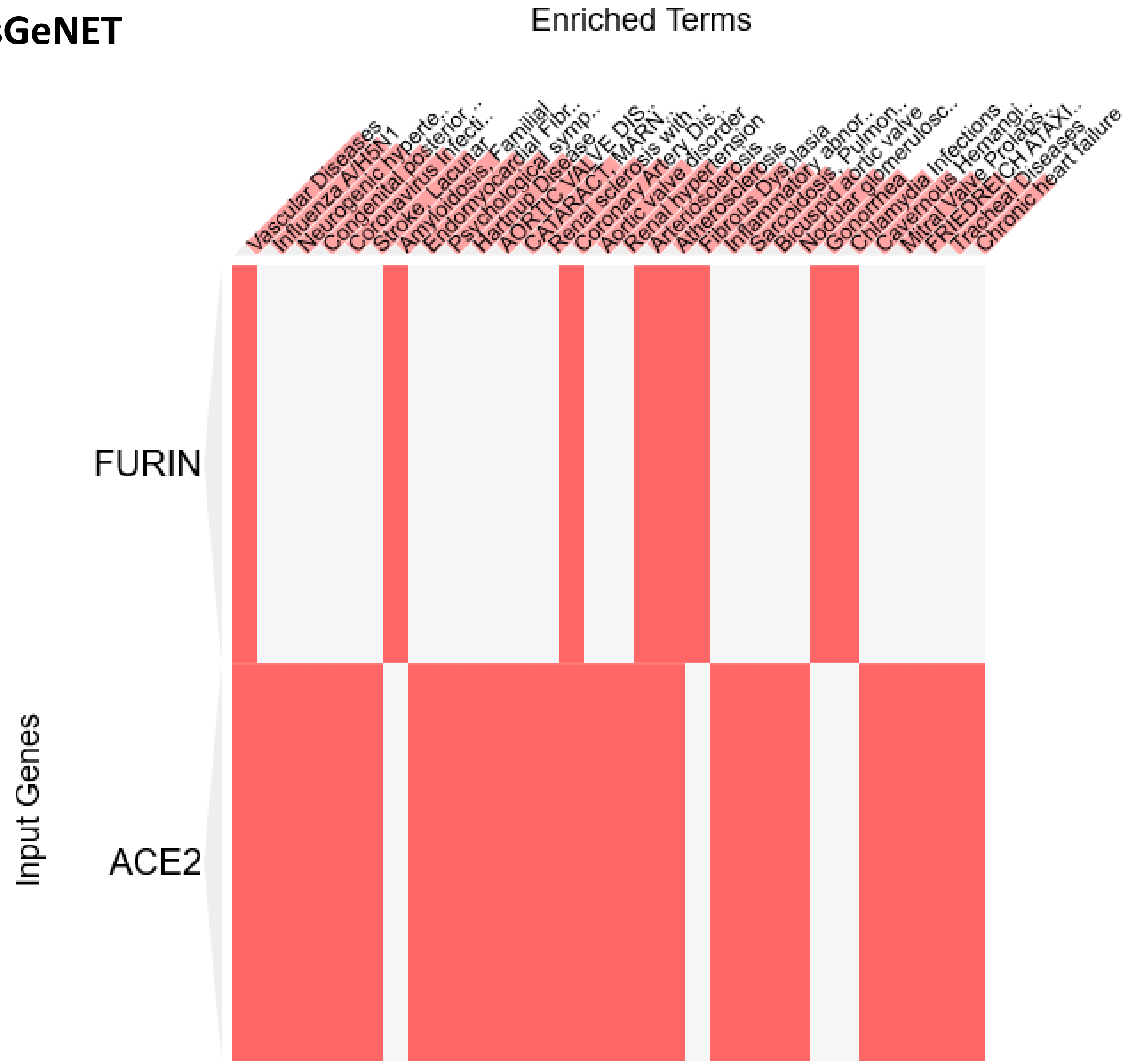

Profile: *ACE2* expression
GDS4855 / 222257_s_at
Title
Pandemic and seasonal H1N1 influenza virus infections of bronchial epithelial cells in vitro
Organism
Homo sapiens

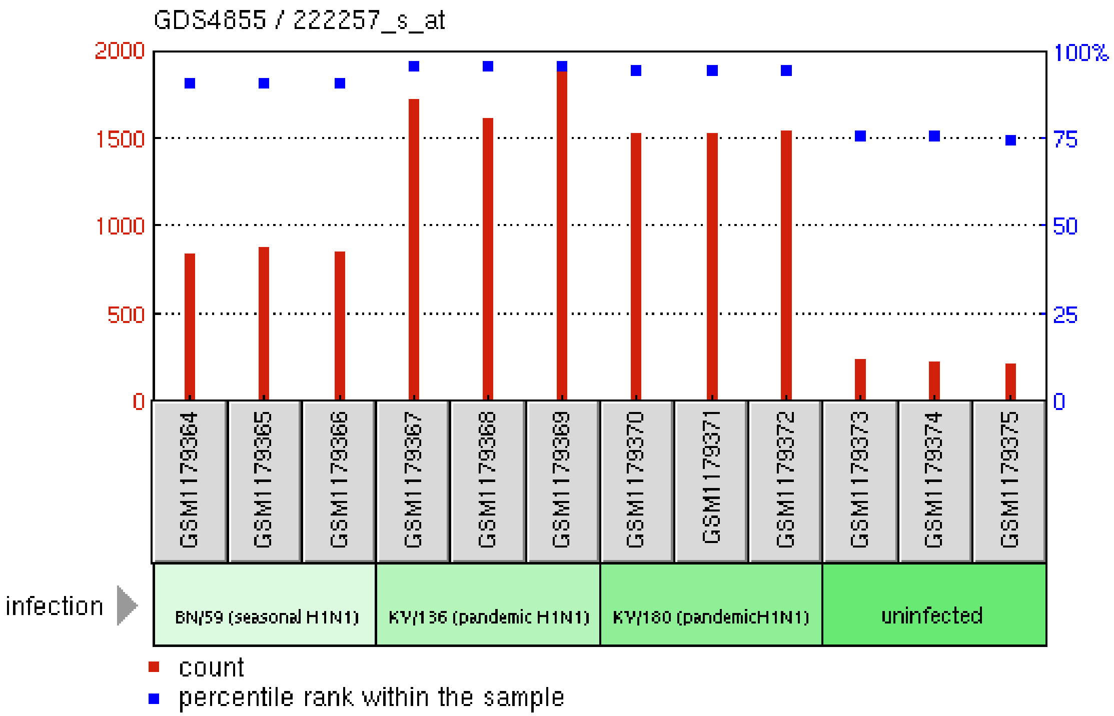

Effects of pandemic and seasonal H1N1 influenza virus infections on *ACE2* expression in human bronchial epithelial cells in vitro

Sample	Title	Value
GSM1179364	wdNHBE with BN/59 at 36hpi, biological rep1	**847.236**
GSM1179365	wdNHBE with BN/59 at 36hpi, biological rep2	**890.883** **$p < 0.05$**
GSM1179366	wdNHBE with BN/59 at 36hpi, biological rep3	**862.141**
GSM1179367	wdNHBE with KY/136 at 36hpi, biological rep1	1734.88
GSM1179368	wdNHBE with KY/136 at 36hpi, biological rep2	1614.9 **$p < 0.05$**
GSM1179369	wdNHBE with KY/136 at 36hpi, biological rep3	1883.21
GSM1179370	wdNHBE with KY/180 at 36hpi, biological rep1	**1533.9**
GSM1179371	wdNHBE with KY/180 at 36hpi, biological rep2	**1540.84** **$p < 0.05$**
GSM1179372	wdNHBE with KY/180 at 36hpi, biological rep3	**1552.49**
GSM1179373	wdNHBE uninfected at 36hpi, biological rep1	**242.199**
GSM1179374	wdNHBE uninfected at 36hpi, biological rep2	**238.781**
GSM1179375	wdNHBE uninfected at 36hpi, biological rep3	**224.471**

Profile: *ACE* expression
GDS4855 / 219962_at
Title
Pandemic and seasonal H1N1 influenza virus infections of bronchial epithelial cells in vitro
Organism
Homo sapiens

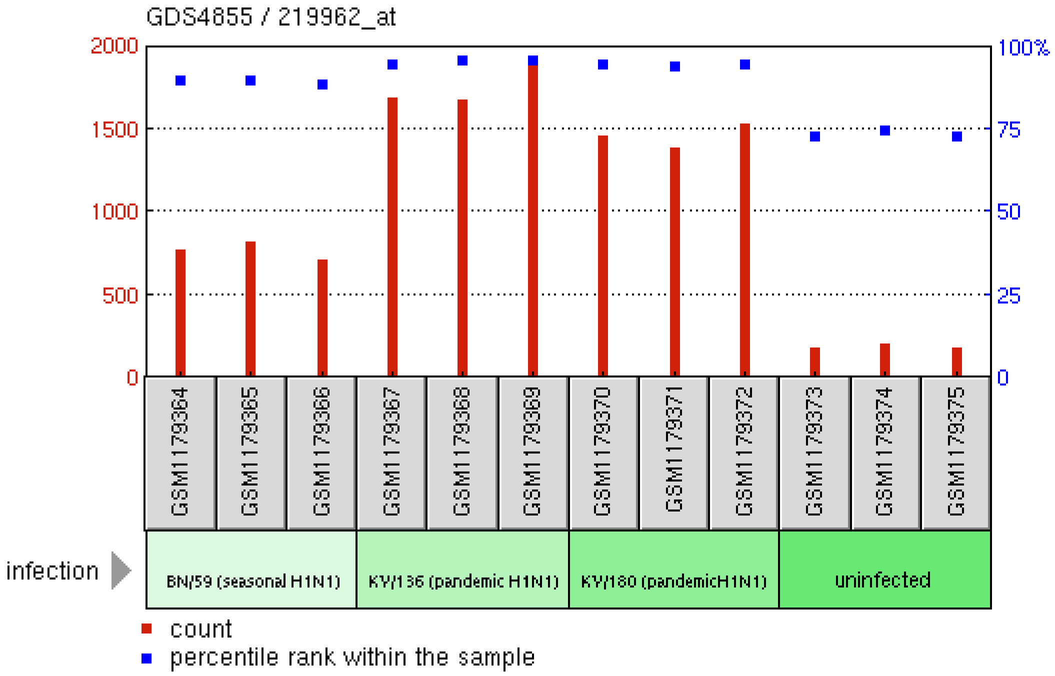

Effects of pandemic and seasonal H1N1 influenza virus infections on *ACE2* expression in human bronchial epithelial cells in vitro

Sample	Title	Value
GSM1179364	wdNHBE with BN/59 at 36hpi, biological rep1	**779.935**
GSM1179365	wdNHBE with BN/59 at 36hpi, biological rep2	**825.417** **$p < 0.05$**
GSM1179366	wdNHBE with BN/59 at 36hpi, biological rep3	**713.858**
GSM1179367	wdNHBE with KY/136 at 36hpi, biological rep1	1691.88
GSM1179368	wdNHBE with KY/136 at 36hpi, biological rep2	1675.14 **$p < 0.05$**
GSM1179369	wdNHBE with KY/136 at 36hpi, biological rep3	1923.87
GSM1179370	wdNHBE with KY/180 at 36hpi, biological rep1	**1464.32**
GSM1179371	wdNHBE with KY/180 at 36hpi, biological rep2	**1393.98** **$p < 0.05$**
GSM1179372	wdNHBE with KY/180 at 36hpi, biological rep3	**1536.46**
GSM1179373	wdNHBE uninfected at 36hpi, biological rep1	**190.363**
GSM1179374	wdNHBE uninfected at 36hpi, biological rep2	**214.431**
GSM1179375	wdNHBE uninfected at 36hpi, biological rep3	**185.11**

Profile: ACE2 expression
GDS2490 / 222257_s_at
Title
Large airway epithelium response to cigarette smoking (HG-133A)
Organism
Homo sapiens

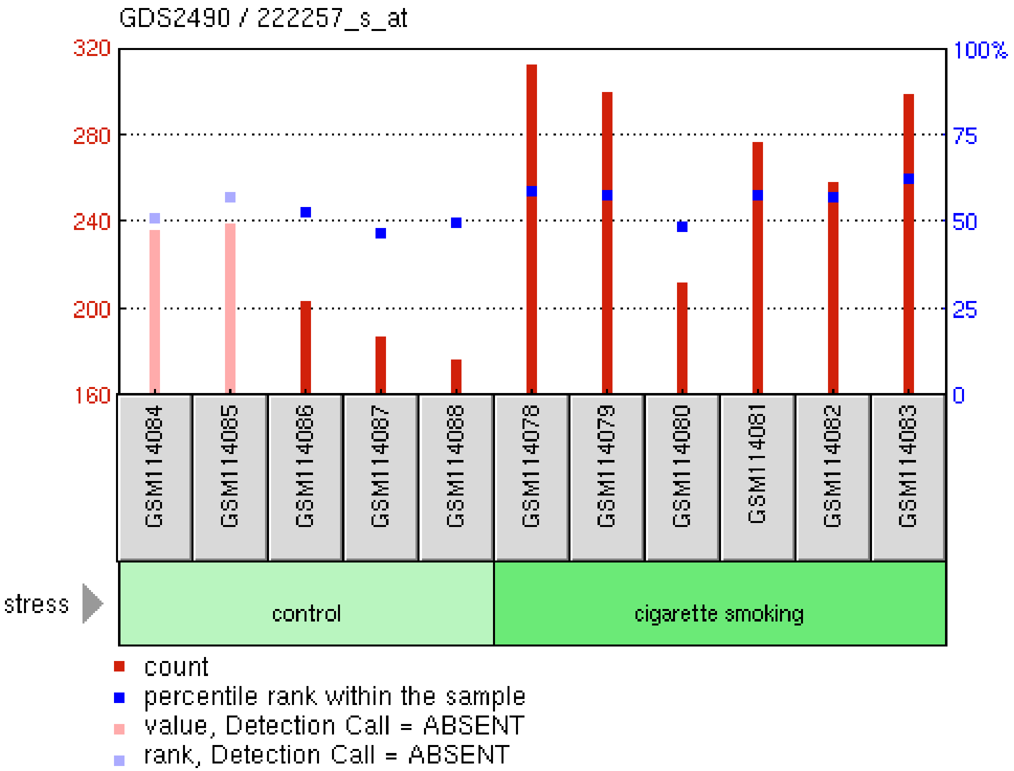

$p < 0.05$

Sample	Title	Value
GSM114084	large airways, non-smoker 1, MAS5	236.4
GSM114085	large airways, non-smoker 2, MAS5	239.9
GSM114086	large airways, non-smoker 3, MAS5	203.8
GSM114087	large airways, non-smoker 4, MAS5	187.6
GSM114088	large airways, non-smoker 5, MAS5	176.5
GSM114078	large airways, smoker 1, MAS5	**313.1**
GSM114079	large airways, smoker 2, MAS5	**300.5**
GSM114080	large airways, smoker 3, MAS5	**212.8**
GSM114081	large airways, smoker 4, MAS5	**277.3**
GSM114082	large airways, smoker 5, MAS5	**258.6**
GSM114083	large airways, smoker 6, MAS5	**299.2**

ACE2 and FURIN

Supplemental Figure S4. Gene ontology (GO) analyses

GO Biological Process 2018

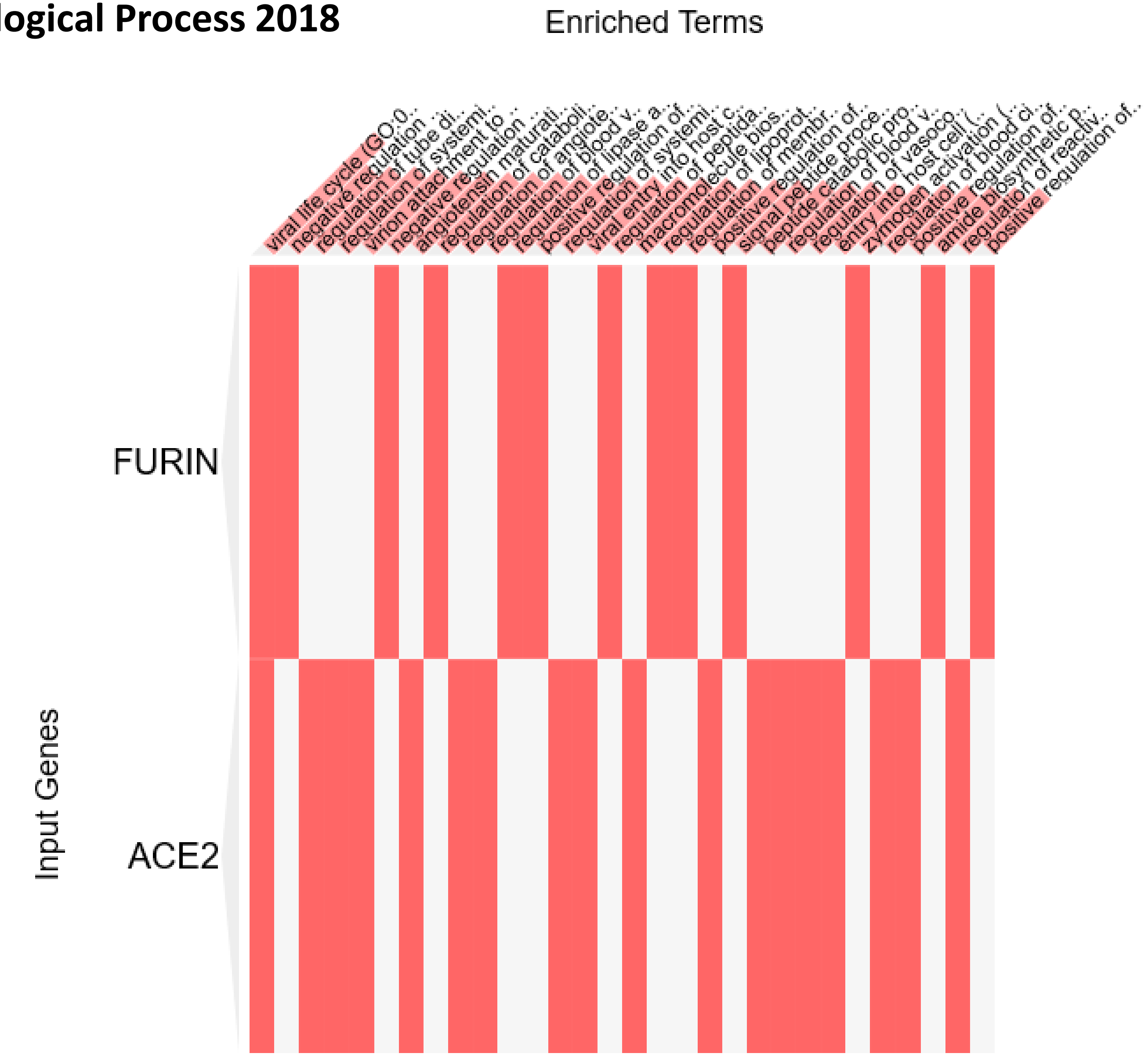

GO Molecular Function 2018

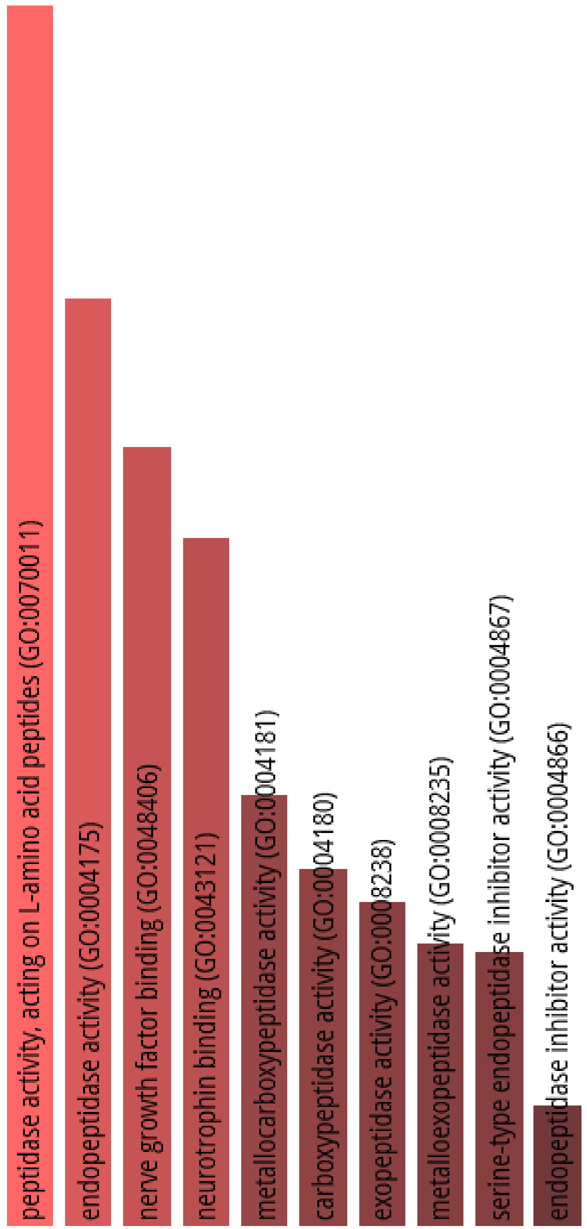

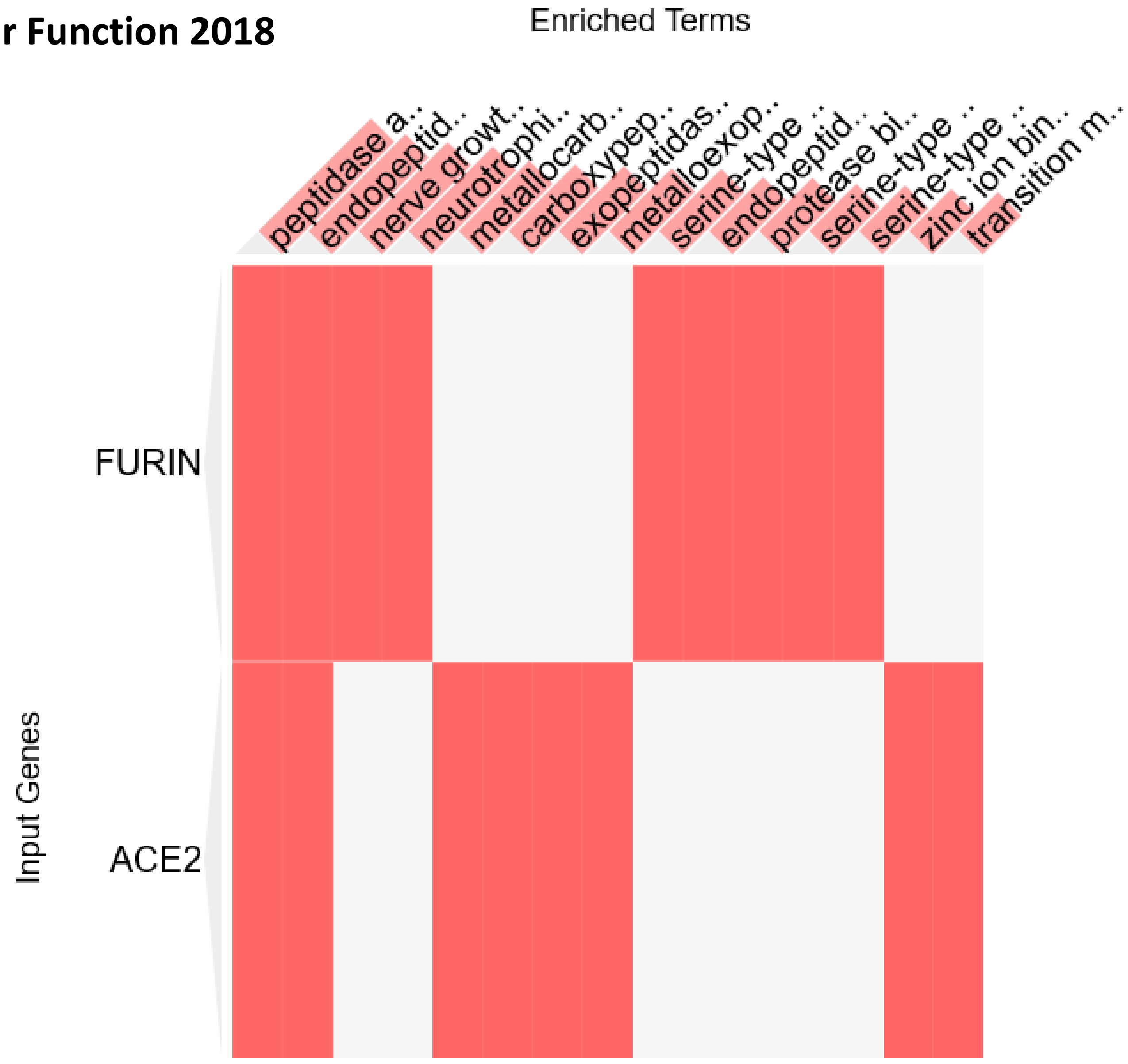

GO Cellular Component 2018

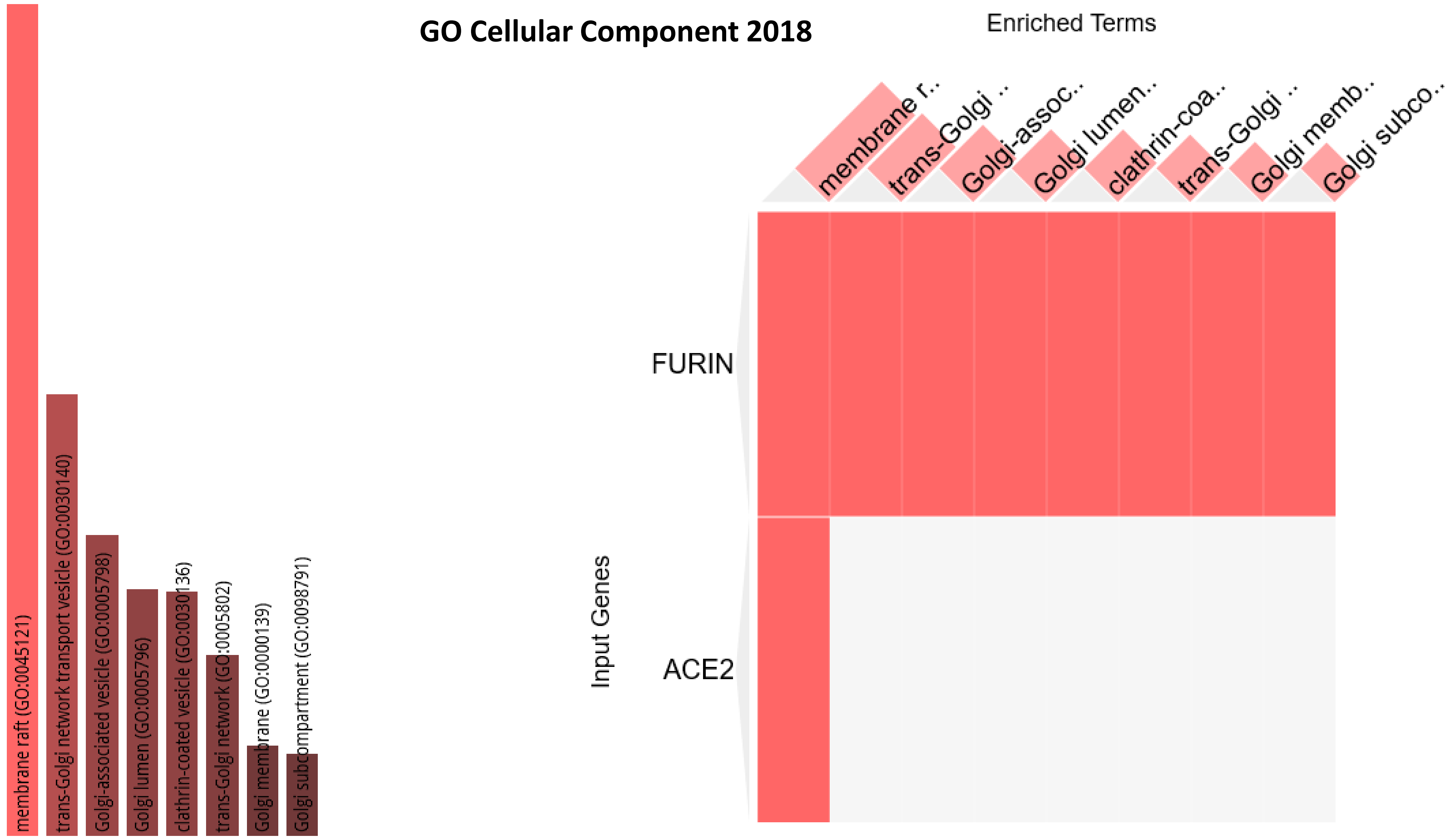

Jensen COMPARTMENTS

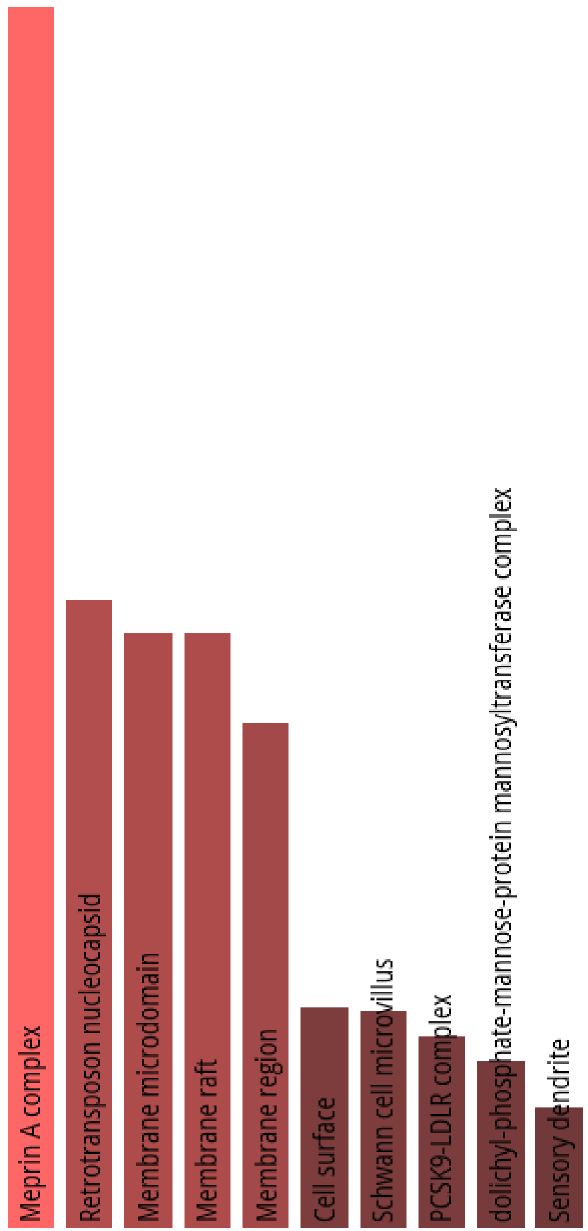

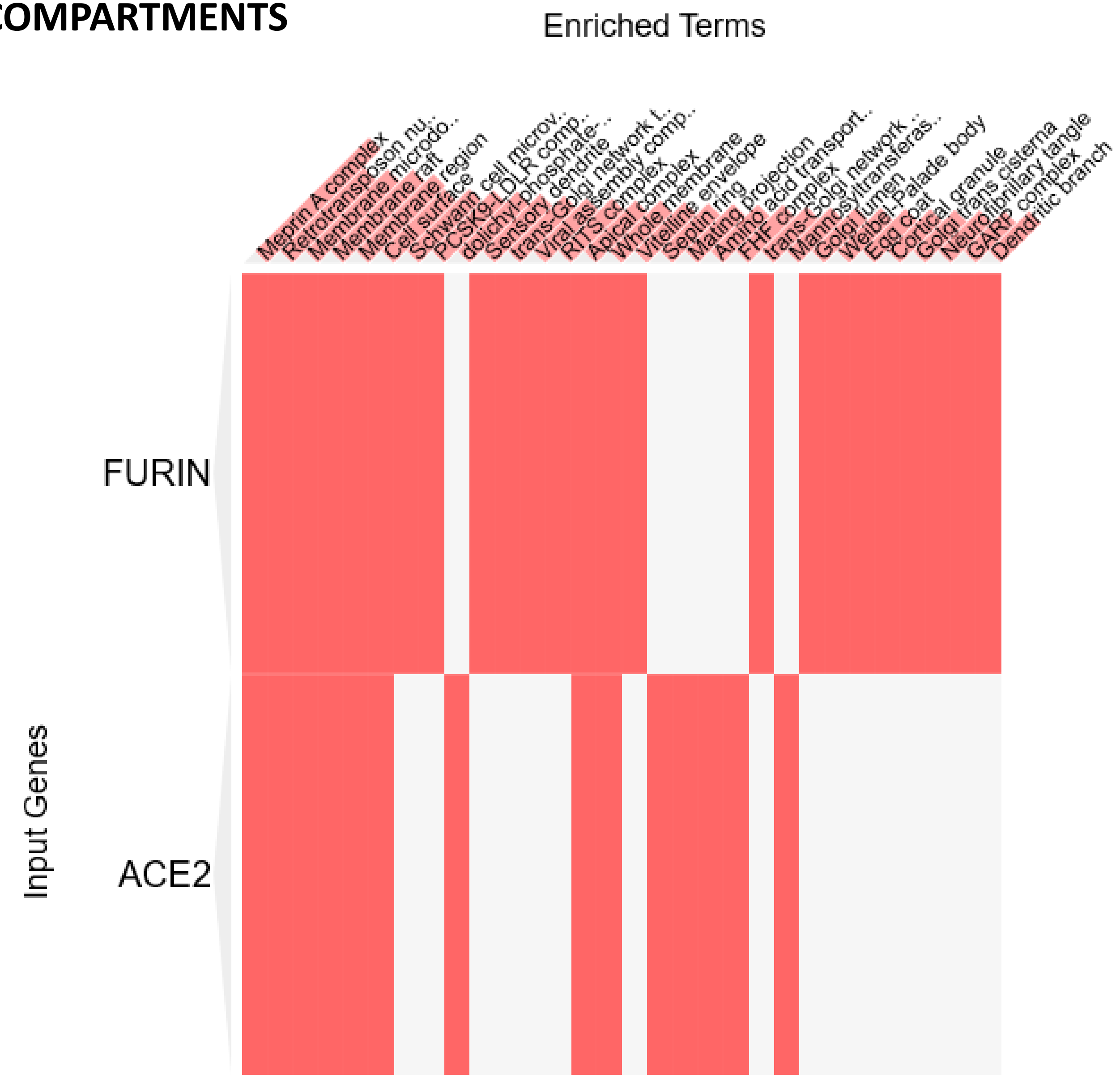

ACE2 and FURIN

Supplemental Figure S5. Potential mechanisms affecting gene expression: identifications of the enriched records of transcription factor-binding sites

Predominantly distinct transcription factors make-up chromatin of the ACE2 and FURIN genes

ENCODE TF ChIP-seq 2015

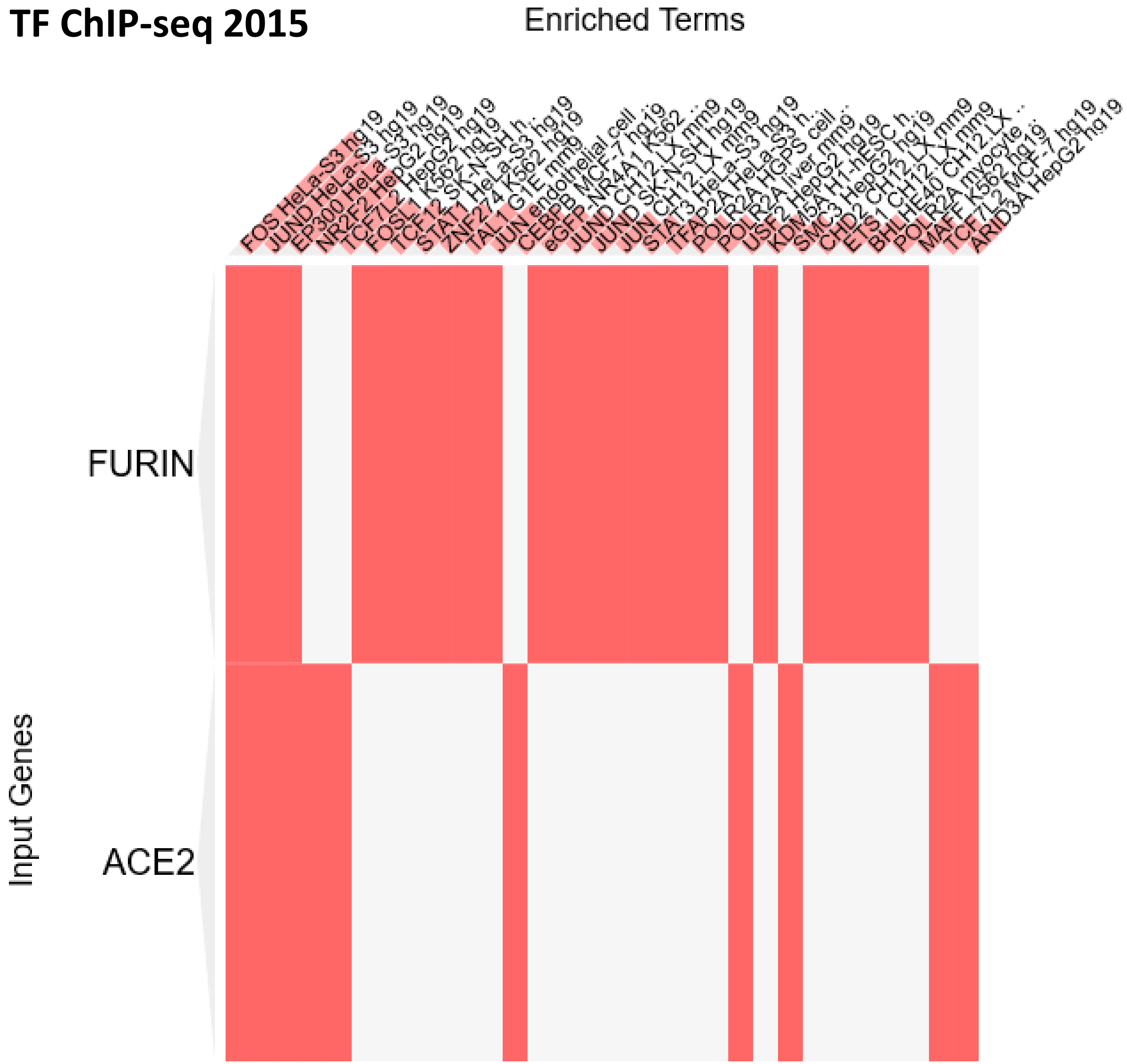

ACE2 and FURIN

Supplemental Figure S5-1. Potential mechanisms affecting gene expression: identifications of the enriched records of pathways, protein-protein interactions (PPI) hub proteins, and drugs affecting *ACE2* and *FURIN* expression

Distinct patterns of pathways associated with the *ACE2* and *FURIN* genes

BioPlanet 2019

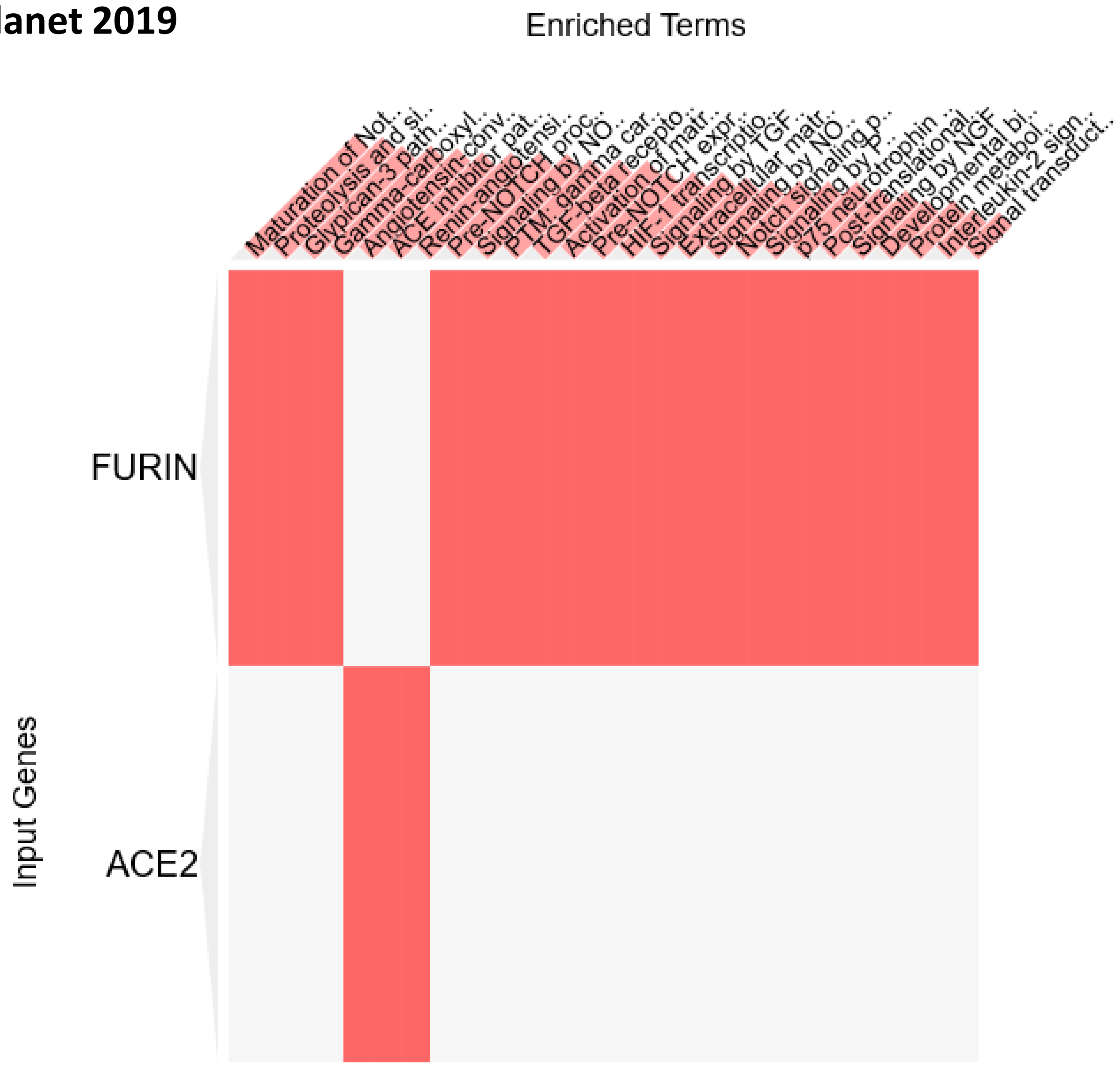

Distinct patterns of protein-protein interactions' (PPI) hub proteins associated with the *ACE2* and *FURIN* gene products

PPI Hub Proteins

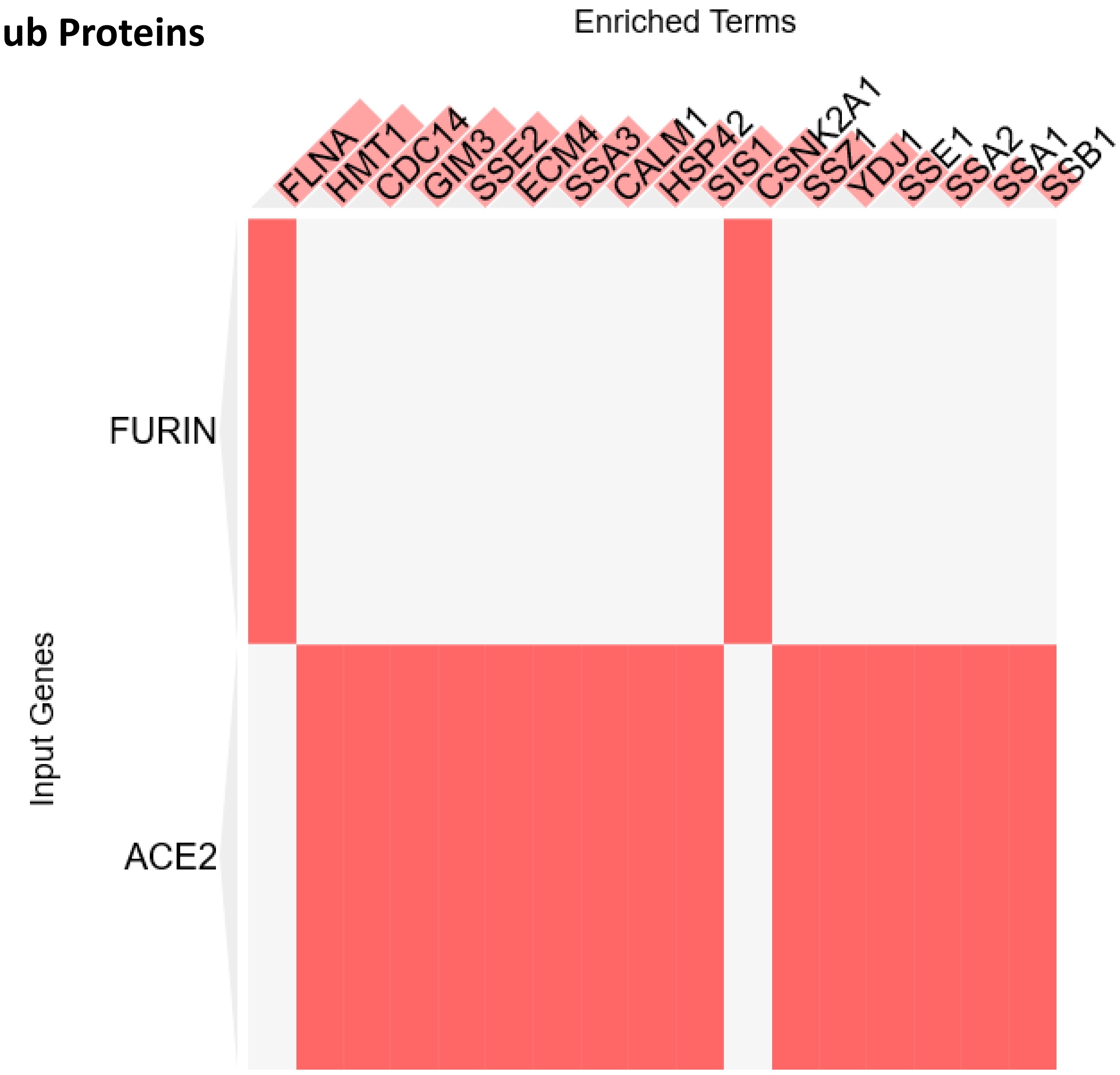

Distinct patterns of drugs affecting expression of the *ACE2* and *FURIN* genes

DSigDB

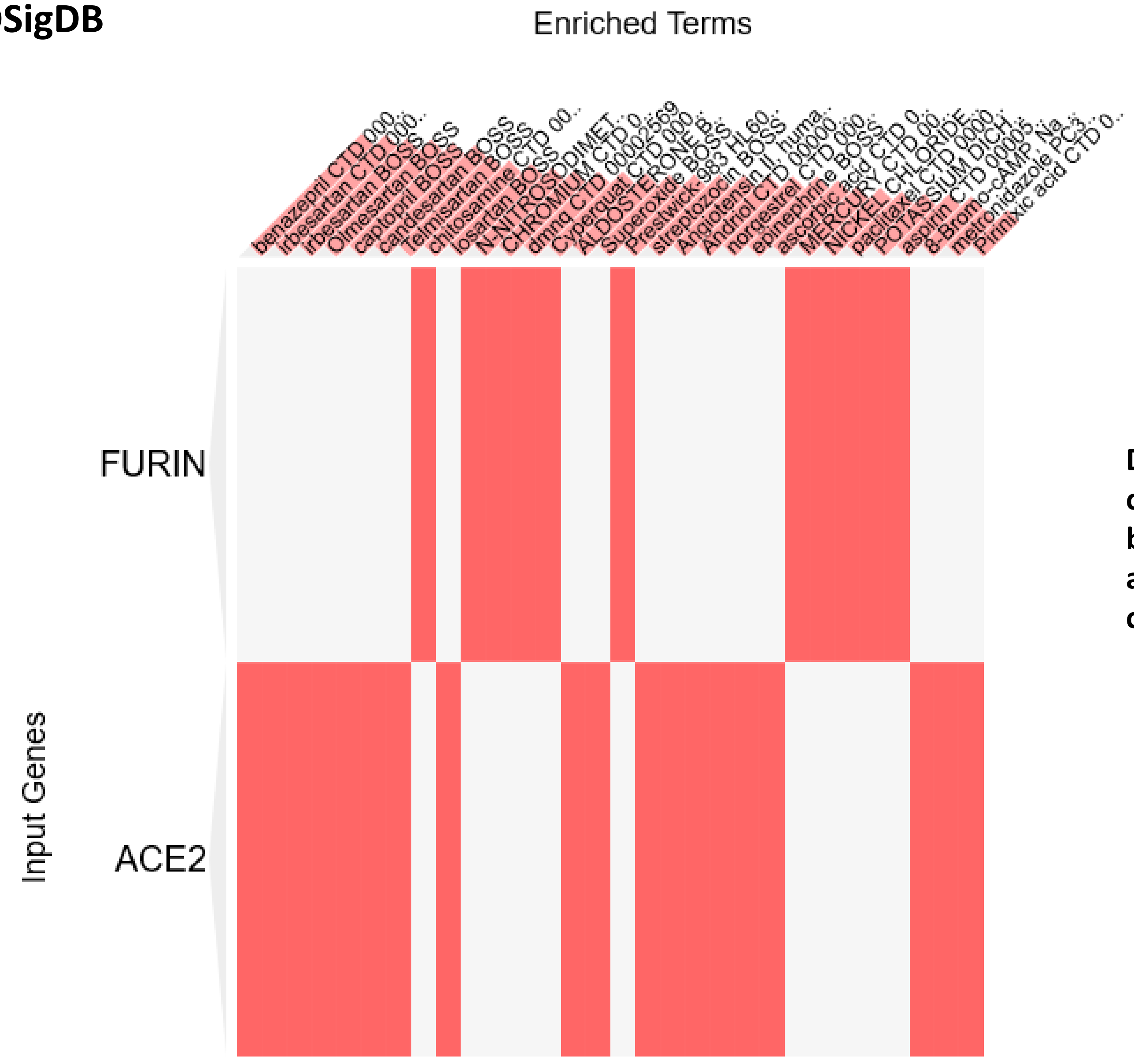

Drug Signatures Database contains drug/gene associations based on quantitative inhibition and drug-induced gene expression changes

ACE2 and FURIN

Supplemental Figure S5-2. Potential mechanisms affecting gene expression: identifications of the enriched records of transcription factor-binding sites that affect expression of target genes

Profile: *ACE2* expression
GDS3001 / 1425103_at
Title
c-Jun N-terminal kinase 1 depletion effect on livers of diet-induced obese animals
Organism
Mus musculus

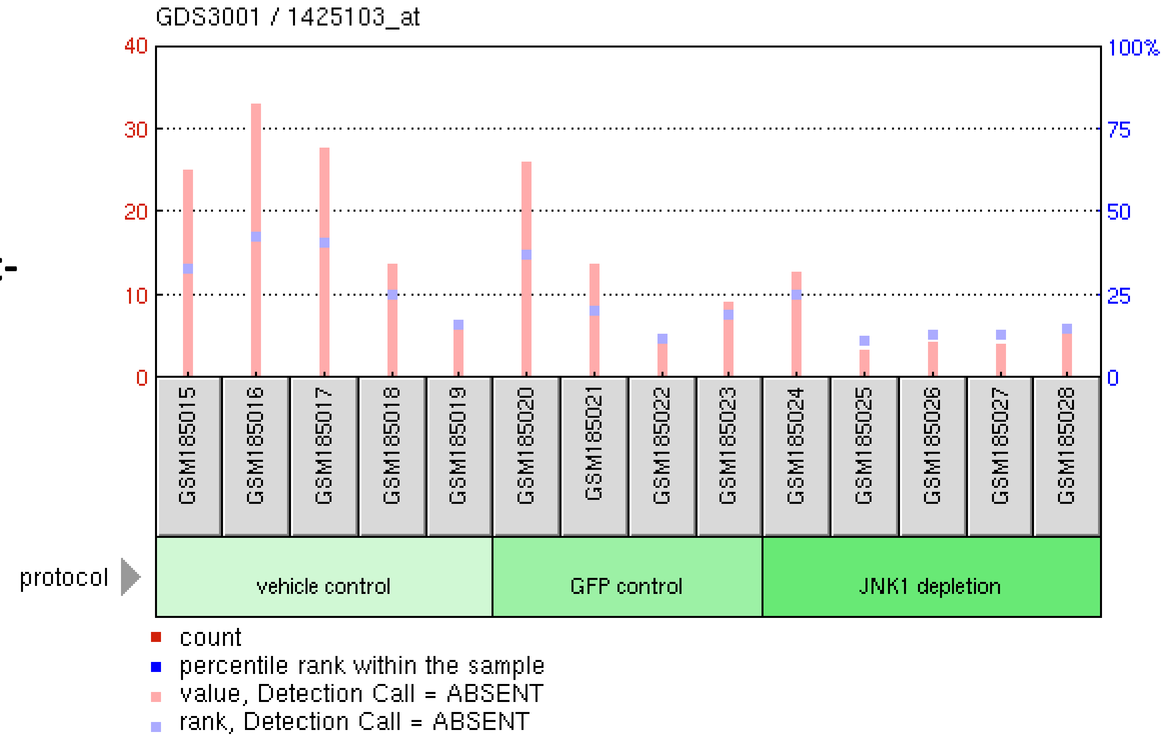

JNK1 depletion is associated with decreased expression of the *ACE2* gene

Sample	Title	Value
GSM185015	DIO Vehicle 1	**25.1364**
GSM185016	DIO Vehicle 2	**33.1885**
GSM185017	DIO Vehicle 3	**27.7494**
GSM185018	DIO Vehicle 4	**13.9459**
GSM185019	DIO Vehicle 5	**6.24653**
GSM185020	GFP Adv-shRNA 1	26.1209
GSM185021	GFP Adv-shRNA 2	13.8813
GSM185022	GFP Adv-shRNA 3	4.77496
GSM185023	GFP Adv-shRNA 5	9.28534
GSM185024	Jnk1 Adv-shRNA 1	**12.9219**
GSM185025	Jnk1 Adv-shRNA 2	**3.46532**
GSM185026	Jnk1 Adv-shRNA 3	**4.42602**
GSM185027	Jnk1 Adv-shRNA 4	**4.17173**
GSM185028	Jnk1 Adv-shRNA 5	**5.42079**

$p < 0.05$

Profile: *Fos* expression
GDS3512 / 1423100_at
Title
Furin deficiency effect on T-cells
Organism
Mus musculus

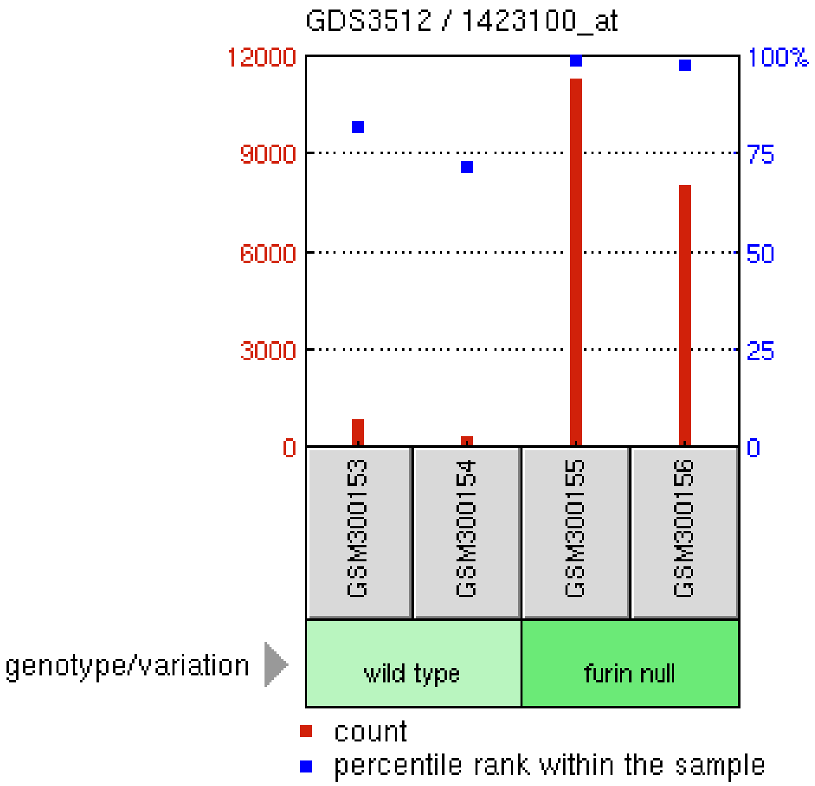

$p < 0.05$

***Furin* depletion is associated with increased *Fos* expression**

Sample	Title	Value
GSM300153	Naive_Furin_Wild-type_1	873.5
GSM300154	Naive_Furin_Wild-type_2	374.2
GSM300155	Naive_Furin_Knockout_1	11333.5
GSM300156	Naive_Furin_Knockout_2	8045.2

Profile: *Jun* expression
GDS3512 / 1417409_at
Title
Furin deficiency effect on T-cells
Organism
Mus musculus

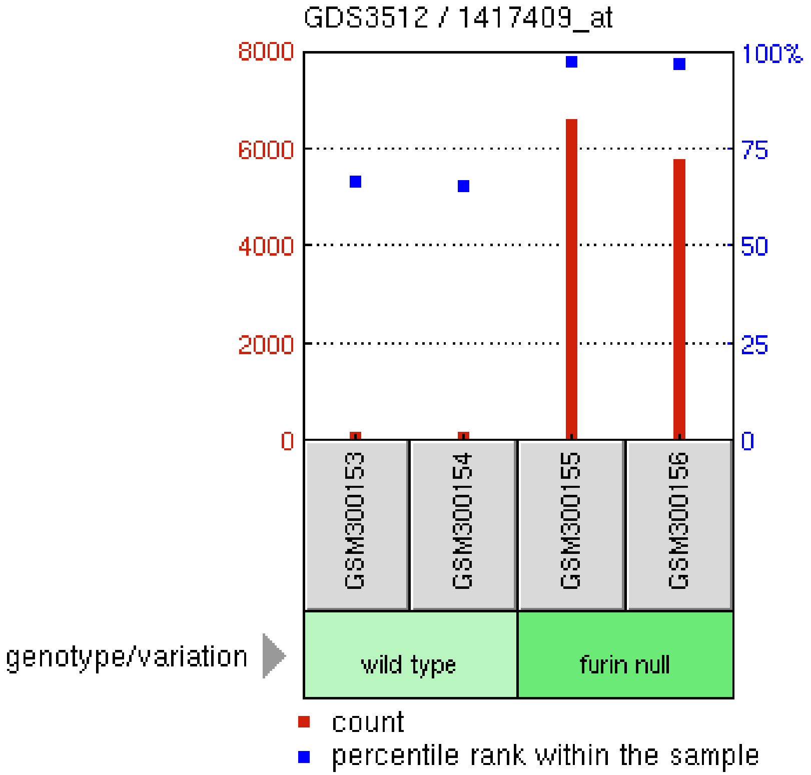

$p < 0.05$

***Furin* depletion is associated with increased *Jun* expression**

Sample	Title	Value
GSM300153	Naive_Furin_Wild-type_1	232.7
GSM300154	Naive_Furin_Wild-type_2	238.9
GSM300155	Naive_Furin_Knockout_1	6631.6
GSM300156	Naive_Furin_Knockout_2	5795

Profile: *Junb* expression
GDS3512 / 1415899_at
Title
Furin deficiency effect on T-cells
Organism
Mus musculus

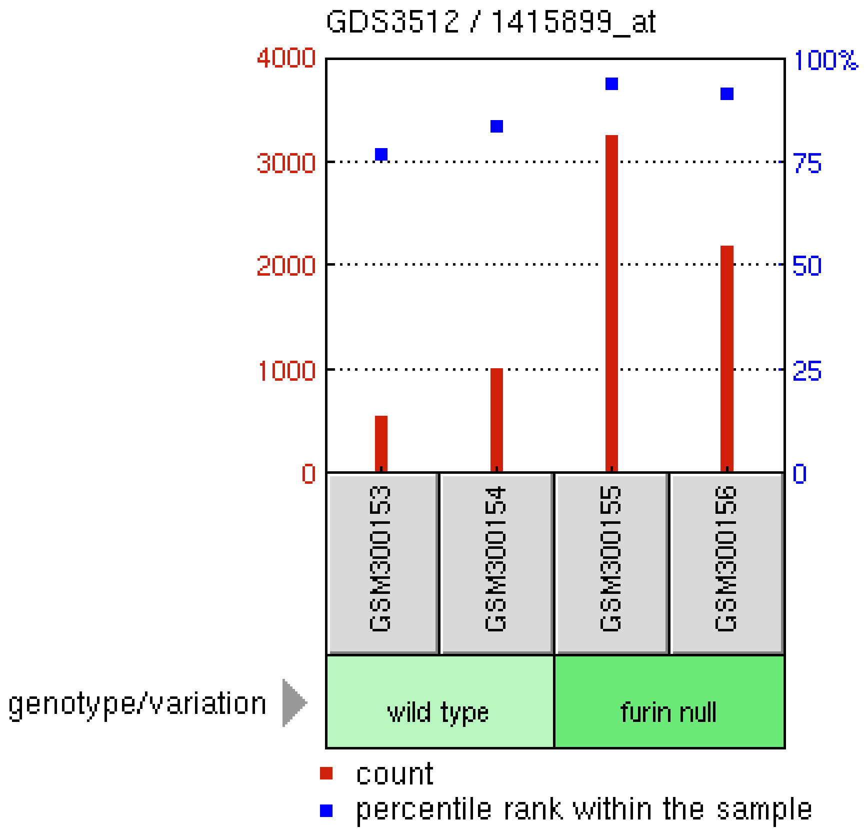

$p < 0.05$

***Furin* depletion is associated with increased *Junb* expression**

Sample	Title	Value
GSM300153	Naive_Furin_Wild-type_1	556
GSM300154	Naive_Furin_Wild-type_2	1032.5
GSM300155	Naive_Furin_Knockout_1	3257.8
GSM300156	Naive_Furin_Knockout_2	2203.1

Profile: *FURIN* expression
GDS2766 / 1418518_at
Title
Dominant negative cJun effect on apolipoprotein E deficient livers
Organism
Mus musculus

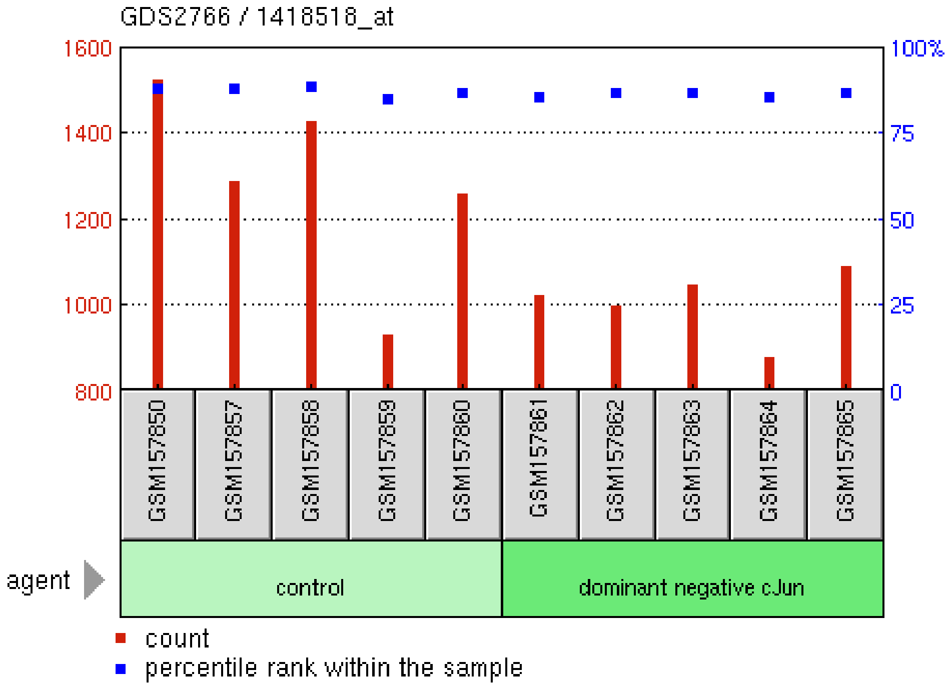

$p < 0.05$

***cJun* inhibition is associated with decreased *Furin* expression**

Sample	Title	Value
GSM157850	Control VZ11	1526.31
GSM157857	Control VZ12	1290.04
GSM157858	Control VZ13	1426.9
GSM157859	Control VZ14	932.365
GSM157860	Control VZ15	1259.69
GSM157861	Dominant negative cJun VZ16	1026.16
GSM157862	Dominant negative cJun VZ17	1002.07
GSM157863	Dominant negative cJun VZ18	1049.73
GSM157864	Dominant negative cJun VZ19	879.625
GSM157865	Dominant negative cJun VZ20	1093.33

Profile: *FURIN* expression
GDS4205 / 10564960
Title
Transcription factor c-JUN knockout effect on B lymphoid cells
Organism
Mus musculus

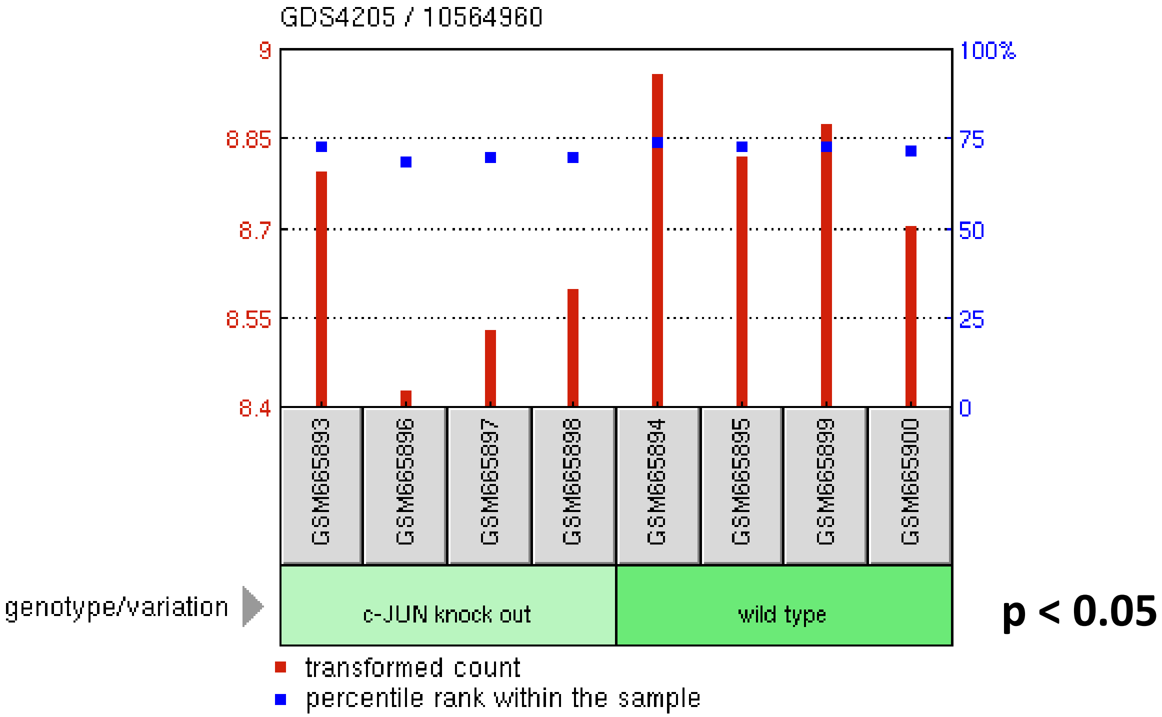

$p < 0.05$

cJun depletion is associated with decreased _Furin_ expression

Sample	Title	Value
GSM665893	RO35	8.79585
GSM665896	RO38	8.42939
GSM665897	D1	8.53367
GSM665898	D6	8.60035
GSM665894	RO36	8.96016
GSM665895	RO37	8.82158
GSM665899	F2	8.87409
GSM665900	F4	8.70635

Profile: *FURIN* expression
GDS1621 / X55660_at
Title
c-Jun N-terminal kinase pathway activation and suppression effect on aortic vascular smooth muscle cells
Organism
Rattus norvegicus

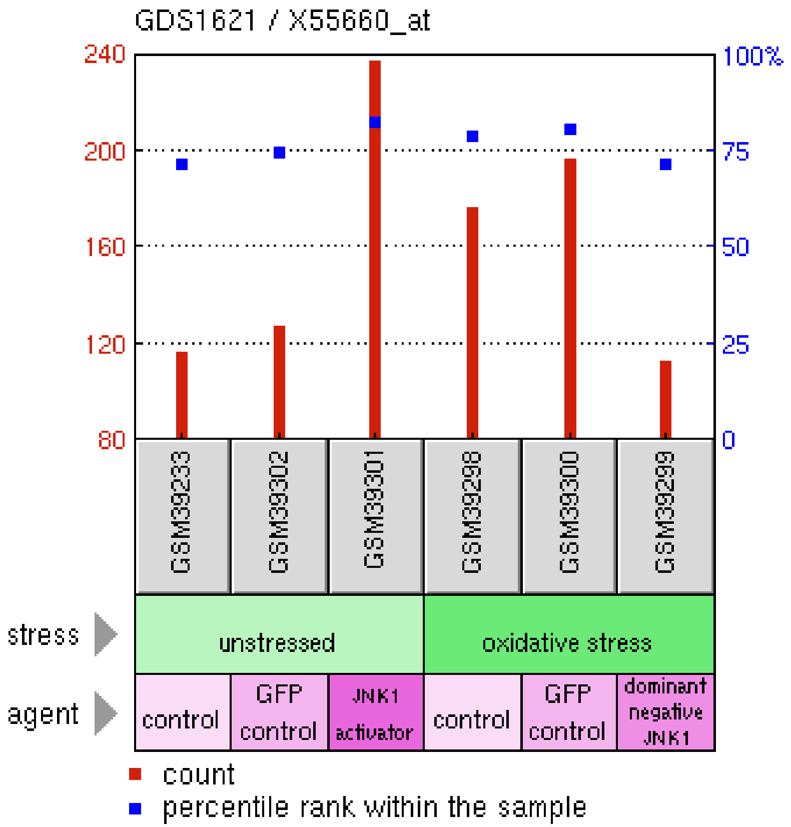

JNK1 pathway **inhibition is associated with decreased** ***Furin*** **expression, while** ***JNK1*** **activation is associated with increased** ***Furin*** **expression**

Sample	Title	Value
GSM39233	rVSMC_control	116.8
GSM39302	rVSMC_nEGFP	128.1
GSM39301	rVSMC_MKK7ED+JNK1WT	**237.3**
GSM39298	rVSMC_H2O2	177.2
GSM39300	rVSMC_H2O2+nEGFP	197.2
GSM39299	rVSMC_H2O2+APF	**113.2**

JNK1/c-FOS pathway-associated activation of the *ACE2* and *FURIN* expression may trigger the auto-regulatory negative feed-back loop of the FURIN-mediated repression of the expression of *JUN, JUNB, JUND,* and *c-FOS* genes

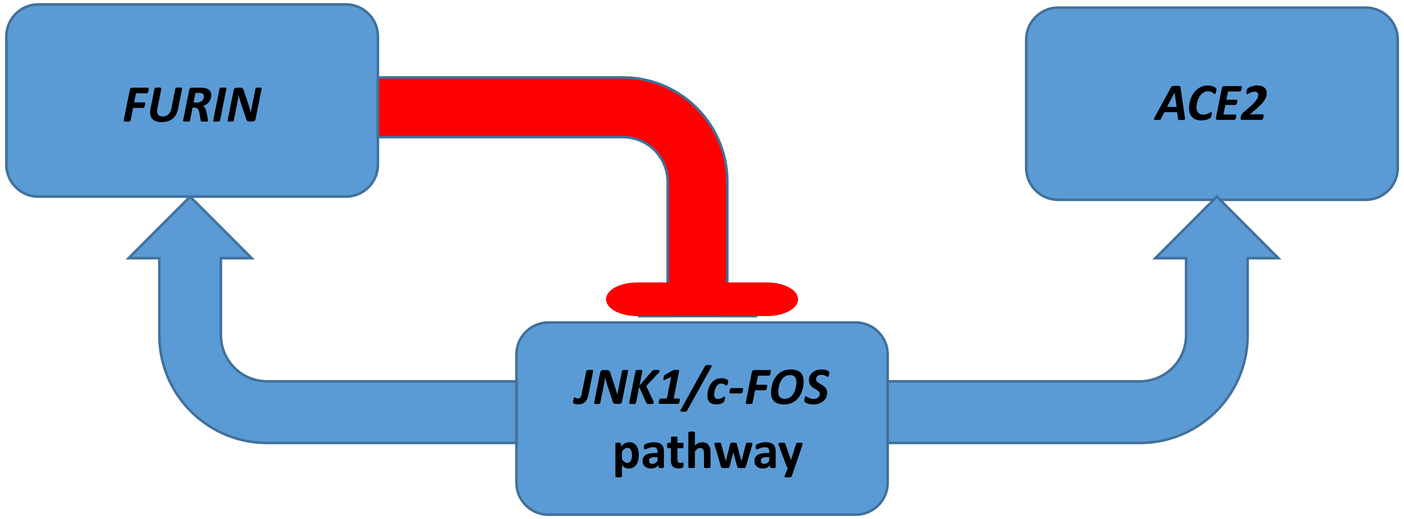

ACE2 and FURIN

Supplemental Figure S6. Potential mechanisms affecting gene expression: identifications of the enriched records of transcription factor-binding sites

Predominantly distinct transcription factors make-up chromatin of the ACE2 and FURIN genes

ChEA 2016

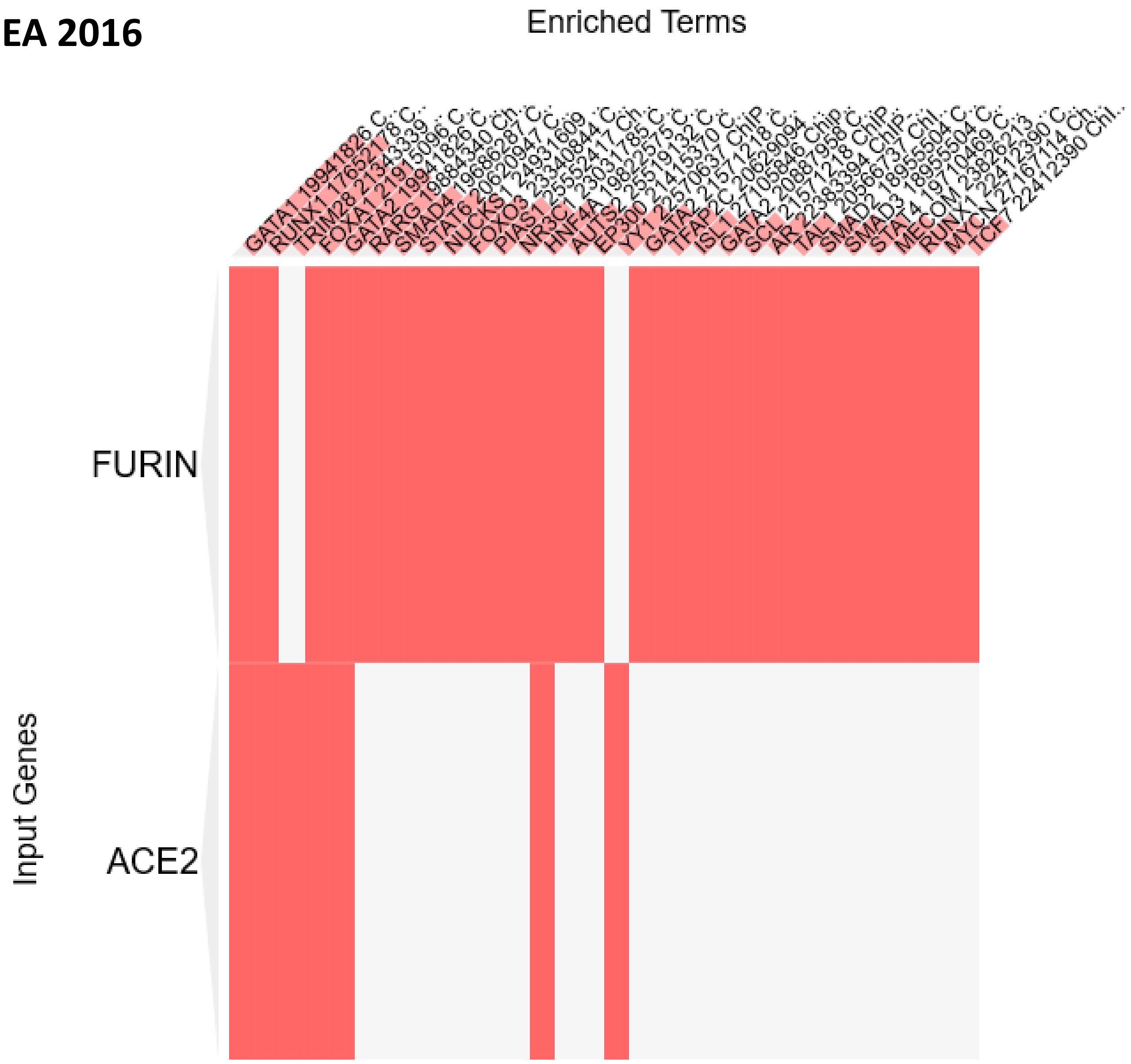

ACE2 and FURIN

Supplemental Figure S6-1. Potential mechanisms affecting gene expression: identifications of the enriched records of transcription factor-binding sites that affect expression of target genes

Profile: *ACE2* expression
GDS1916 / 1425103_at
Title
Hepatocyte nuclear factor 4 alpha knockout effect on the embryonic liver
Organism
Mus musculus

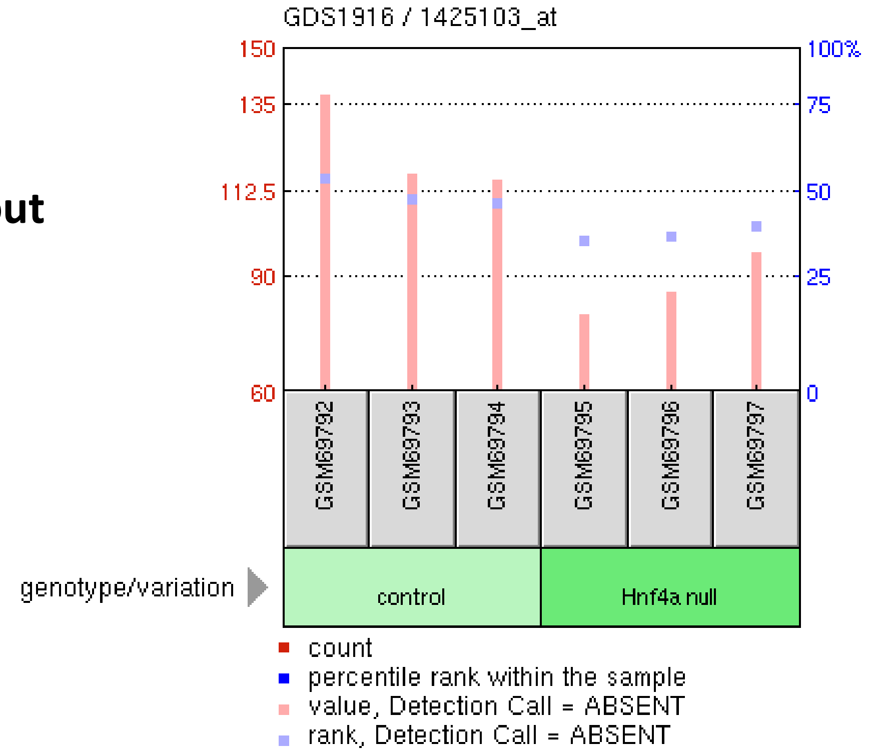

$p < 0.05$

***Hnf4a* deficiency decreases *ACE2* expression in mouse cells**

Sample	Title	Value
GSM69792	HNF4 Control_8	137.683
GSM69793	HNF4 Control_1192	117.056
GSM69794	HNF4 Control_1193	115.675
GSM69795	HNF4 Null_61	**80.4453**
GSM69796	HNF4 Null_1191	**86.1111**
GSM69797	HNF4 Null_1195	**96.6528**

Profile: *ACE2* expression
GDS5284 / 1425102_a_at
Title
Hepatocyte nuclear factor 4 alpha deficiency effect on the colon
Organism
Mus musculus

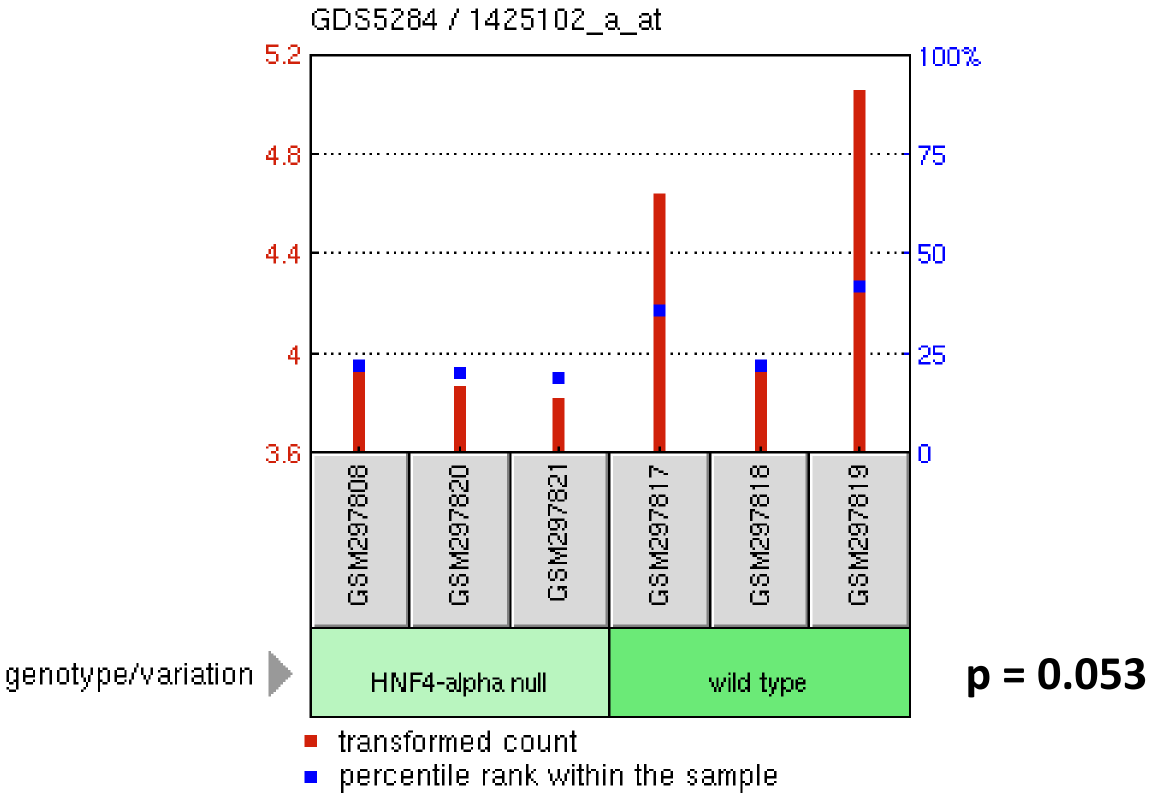

p = 0.053

***Hnf4a* deficiency decreases *ACE2* expression in mouse cells**

Sample	Title	Value
GSM297808	mouse colon_hnf4mutant_rep1	3.94799
GSM297820	mouse colon_hnf4mutant_rep2	3.87791
GSM297821	mouse colon_hnf4mutant_rep3	3.82229
GSM297817	mouse colon_hnf4control_rep1	4.64383
GSM297818	mouse colon_hnf4control_rep2	3.9605
GSM297819	mouse colon_hnf4control_rep3	5.05635

Profile: *Furin* expression
GDS1916 / 1418518_at
Title
Hepatocyte nuclear factor 4 alpha knockout effect on the embryonic liver
Organism
Mus musculus

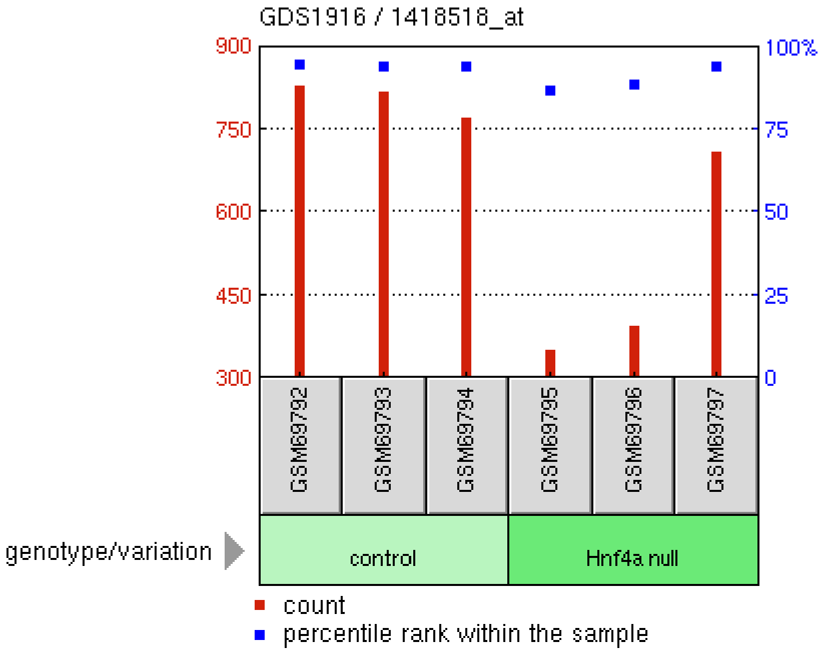

$p < 0.05$

***Hnf4a* deficiency decreases *Furin* expression in murine cells**

Sample	Title	Value
GSM69792	HNF4 Control_8	828.42
GSM69793	HNF4 Control_1192	820.313
GSM69794	HNF4 Control_1193	772.082
GSM69795	HNF4 Null_61	354.031
GSM69796	HNF4 Null_1191	396.723
GSM69797	HNF4 Null_1195	708.976

HNF4a pathway-associated activation of the *ACE2* and *FURIN* expression may trigger the auto-regulatory positive feed-back loop of the *FURIN*-mediated activation of the *HNF4a* expression

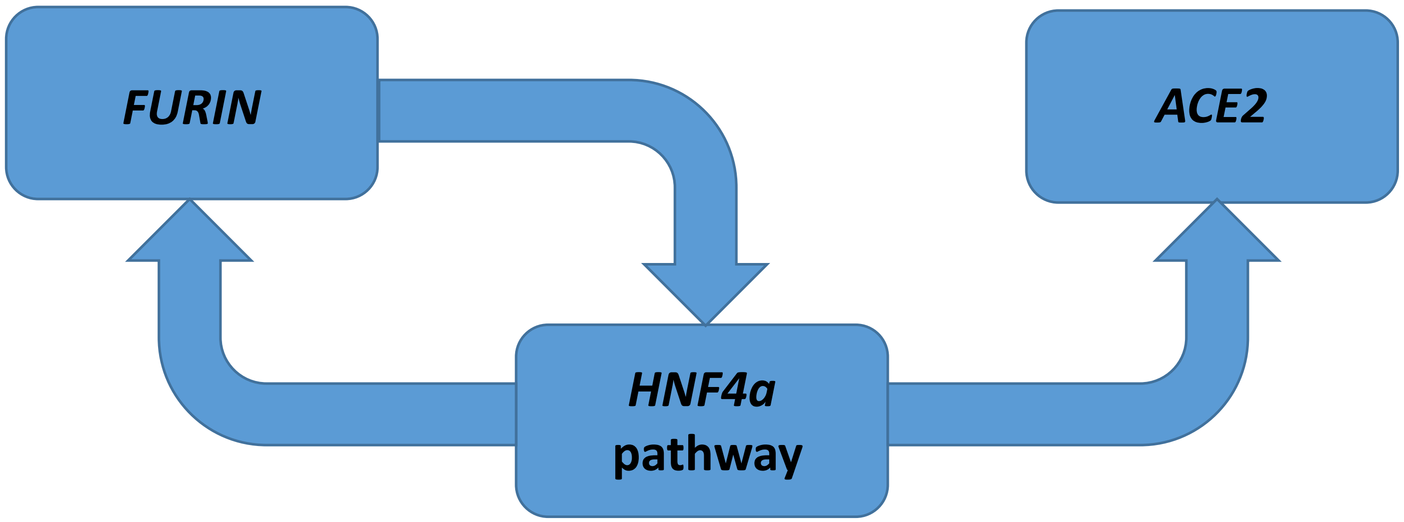

Profile: *ACE2* expression
GDS4441 / 1425103_at
Title
Runt homology domain expression effect on hematopoietic stem cells
Organism
Mus musculus

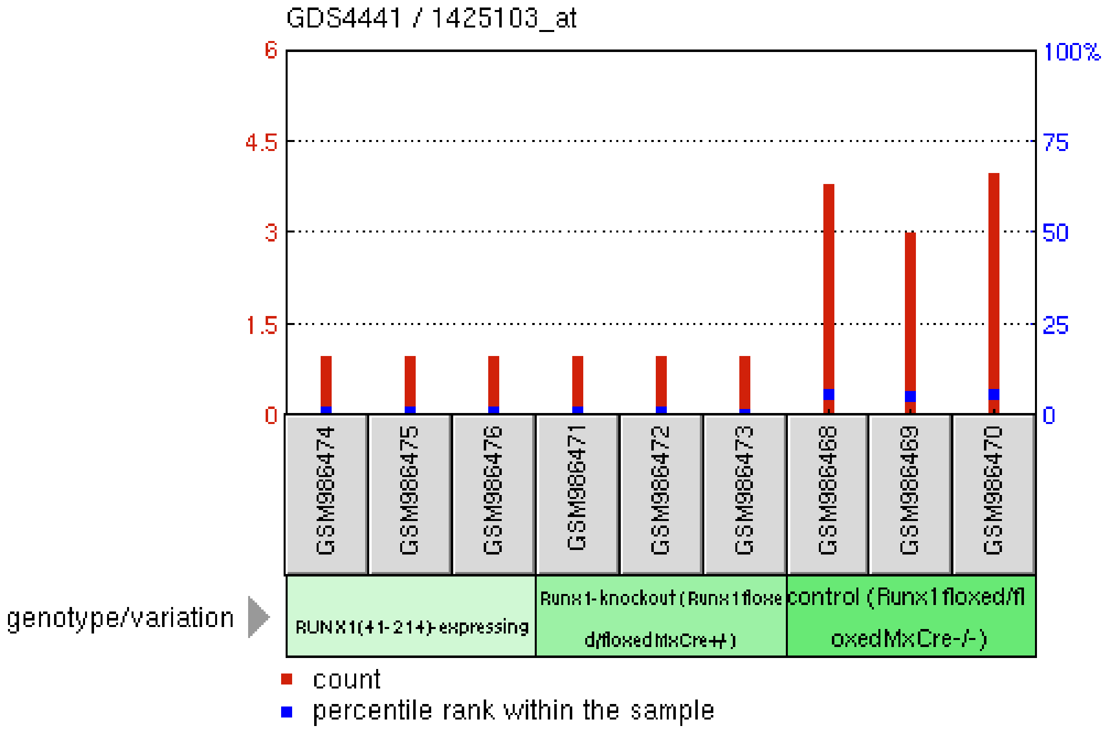

$p < 0.05$

Runx1 deficiency decreases *ACE2* expression in mouse cells

Sample	Title	Value
GSM986474	RUNX1(41-214), biological replicate 1	1
GSM986475	RUNX1(41-214), biological replicate 2	1
GSM986476	RUNX1(41-214), biological replicate 3	1
GSM986471	Runx1KO, biological replicate 1	1
GSM986472	Runx1KO, biological replicate 2	1
GSM986473	Runx1KO, biological replicate 3	1
GSM986468	Control, biological replicate 1	3.83
GSM986469	Control, biological replicate 2	3
GSM986470	Control, biological replicate 3	4

$p < 0.05$

Profile: *FURIN* expression
GDS5606 / 7986092
Title
Androgen effect on runt-related transcription factor 1-deficient prostate cancer cell line
Organism
Homo sapiens

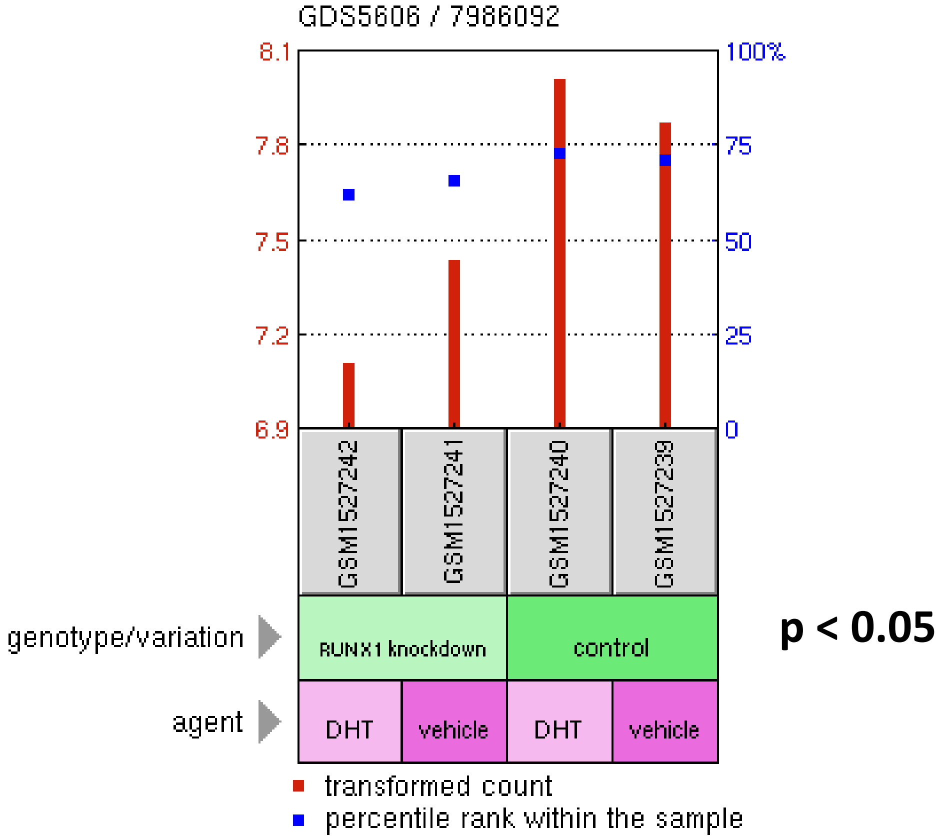

$p < 0.05$

***RUNX1* deficiency decreases *FURIN* expression in human cells**

Sample	Title	Value
GSM1527242	LNCaP_siRUNX1_DHT	**7.11175**
GSM1527241	LNCaP_siRUNX1_Veh	**7.43782**
GSM1527240	LNCaP_siControl_DHT	8.00649
GSM1527239	LNCaP_siControl_Veh	7.87368

Profile: *Runx1* expression
GDS3512 / 1427847_at
Title
Furin deficiency effect on T-cells
Organism
Mus musculus

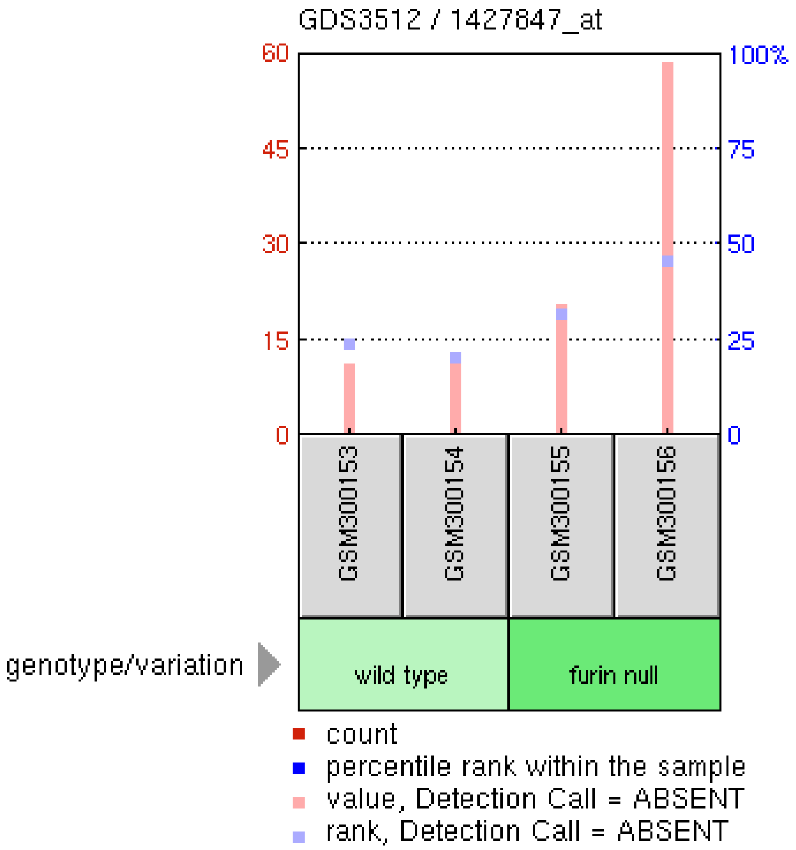

***Furin* deficiency enhances *Runx1* expression in murine T-cells**

Sample	Title	Value
GSM300153	Naive_Furin_Wild-type_1	11.5
GSM300154	Naive_Furin_Wild-type_2	12.5
GSM300155	Naive_Furin_Knockout_1	**20.9**
GSM300156	Naive_Furin_Knockout_2	**58.7**

Profile: *Foxa1* expression
GDS3512 / 1418496_at
Title
Furin deficiency effect on T-cells
Organism
Mus musculus

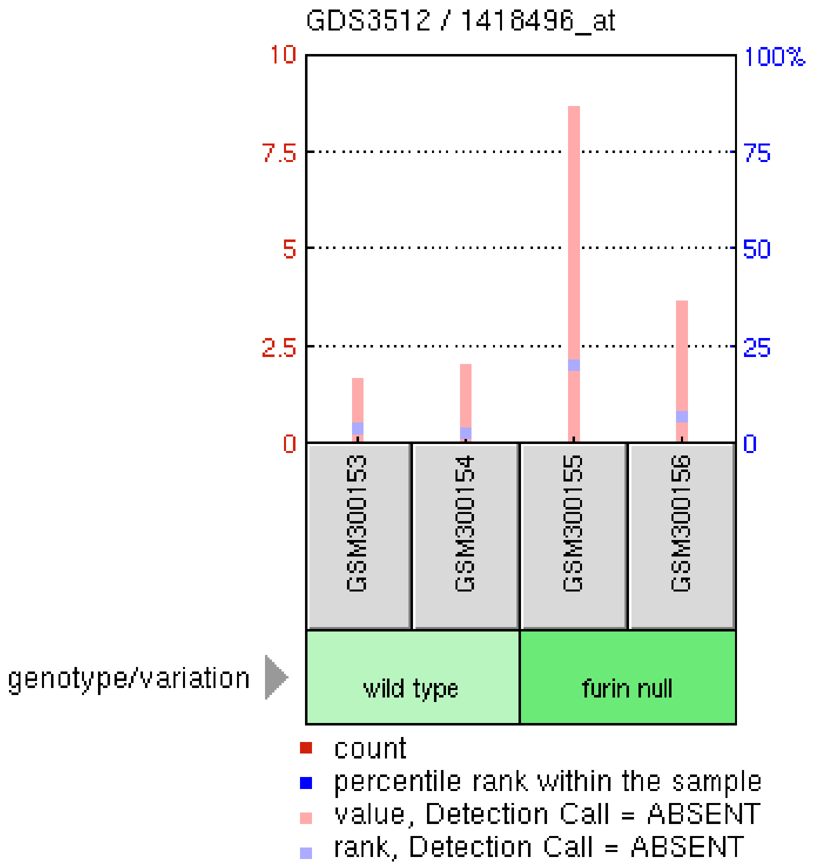

***Furin* deficiency enhances *Foxa1* expression in murine T-cells**

Sample	Title	Value
GSM300153	Naive_Furin_Wild-type_1	1.7
GSM300154	Naive_Furin_Wild-type_2	2.1
GSM300155	Naive_Furin_Knockout_1	**8.7**
GSM300156	Naive_Furin_Knockout_2	**3.7**

RUNX1 pathway-associated activation of the *ACE2* and *FURIN* expression may trigger the auto-regulatory negative feed-back loop of the *FURIN*-mediated repression of the *RUNX1* gene expression

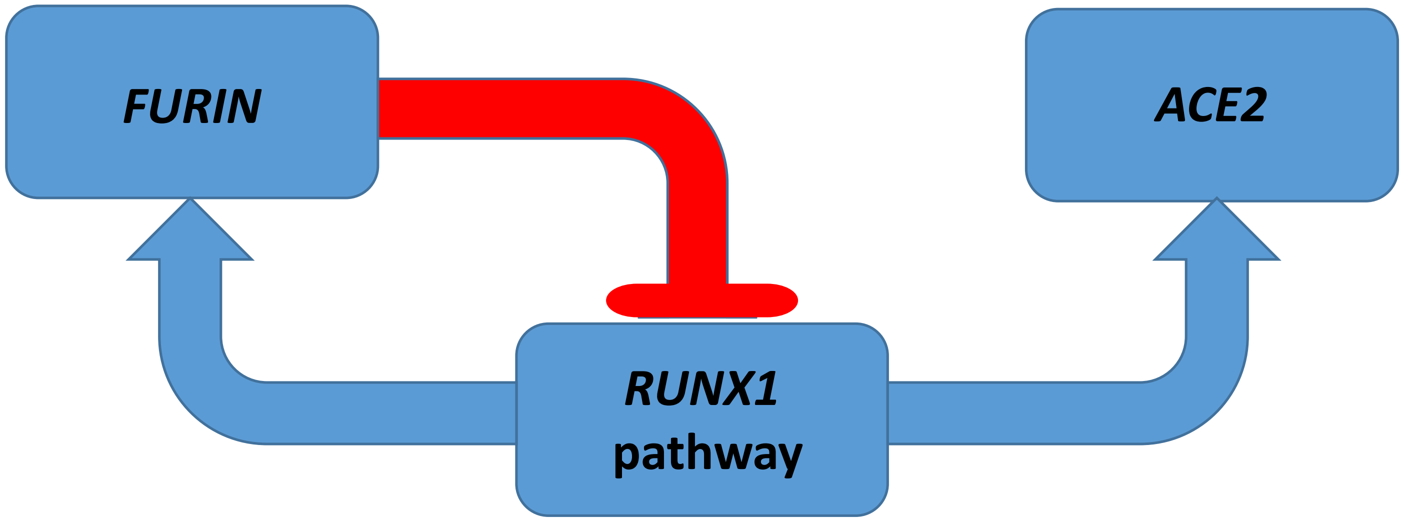

ACE2 and FURIN

Supplemental Figure S7. Potential mechanisms affecting gene expression: Putative inhibitory role of the *VDR* gene and Vitamin D

ARCHS4 TFs Co-expression

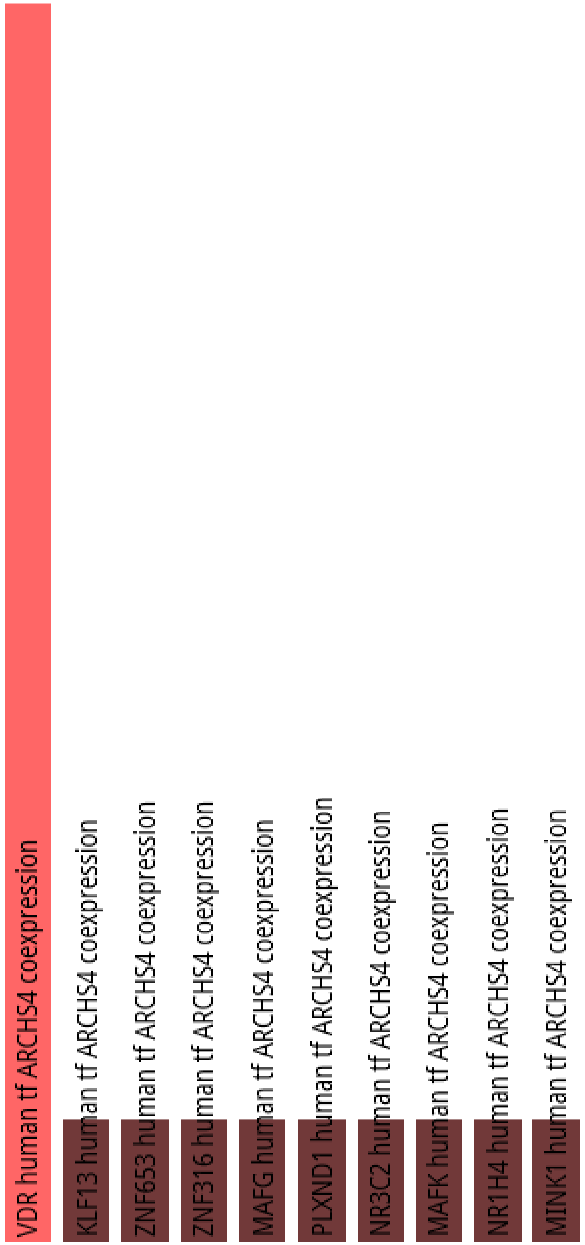

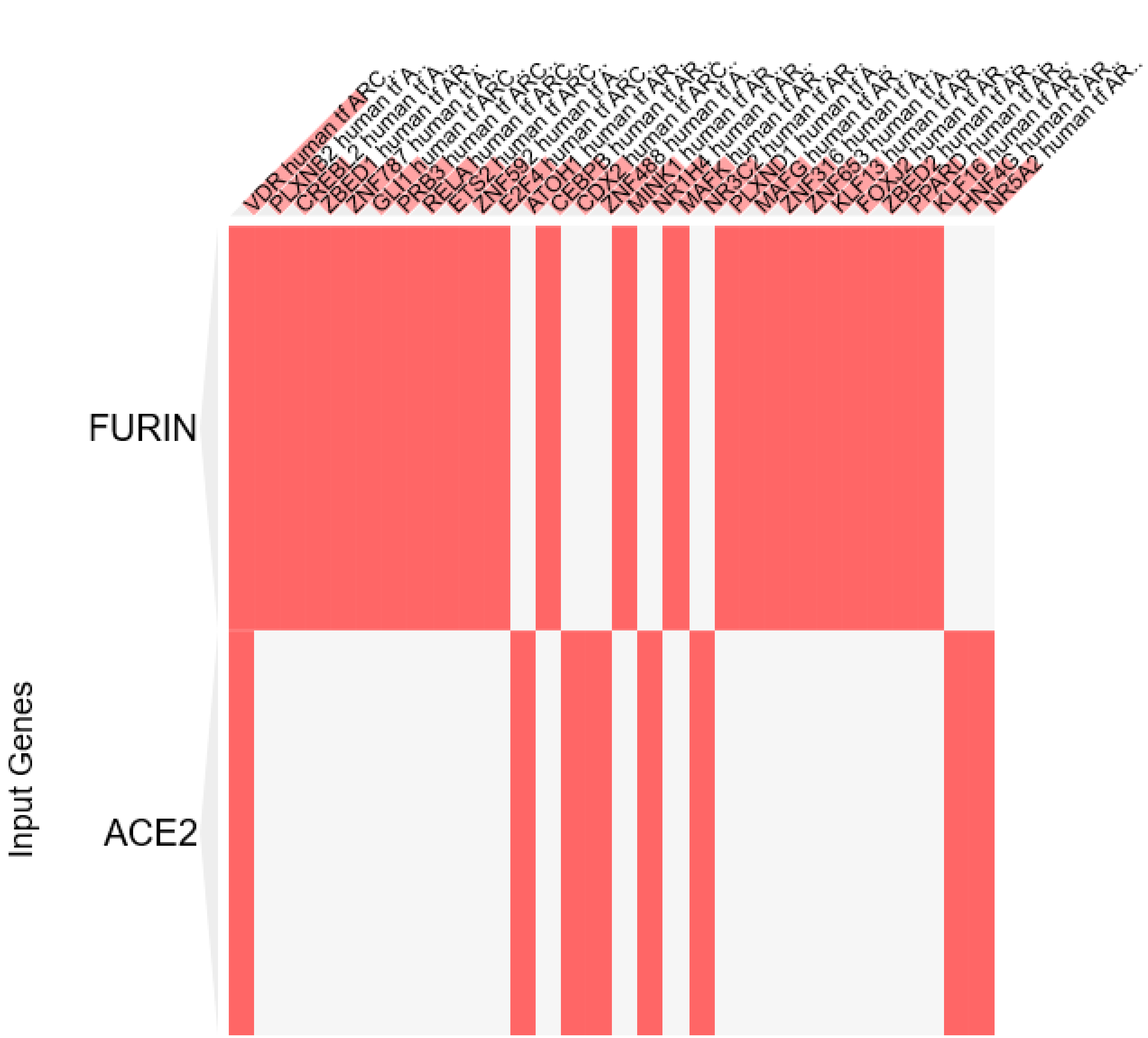

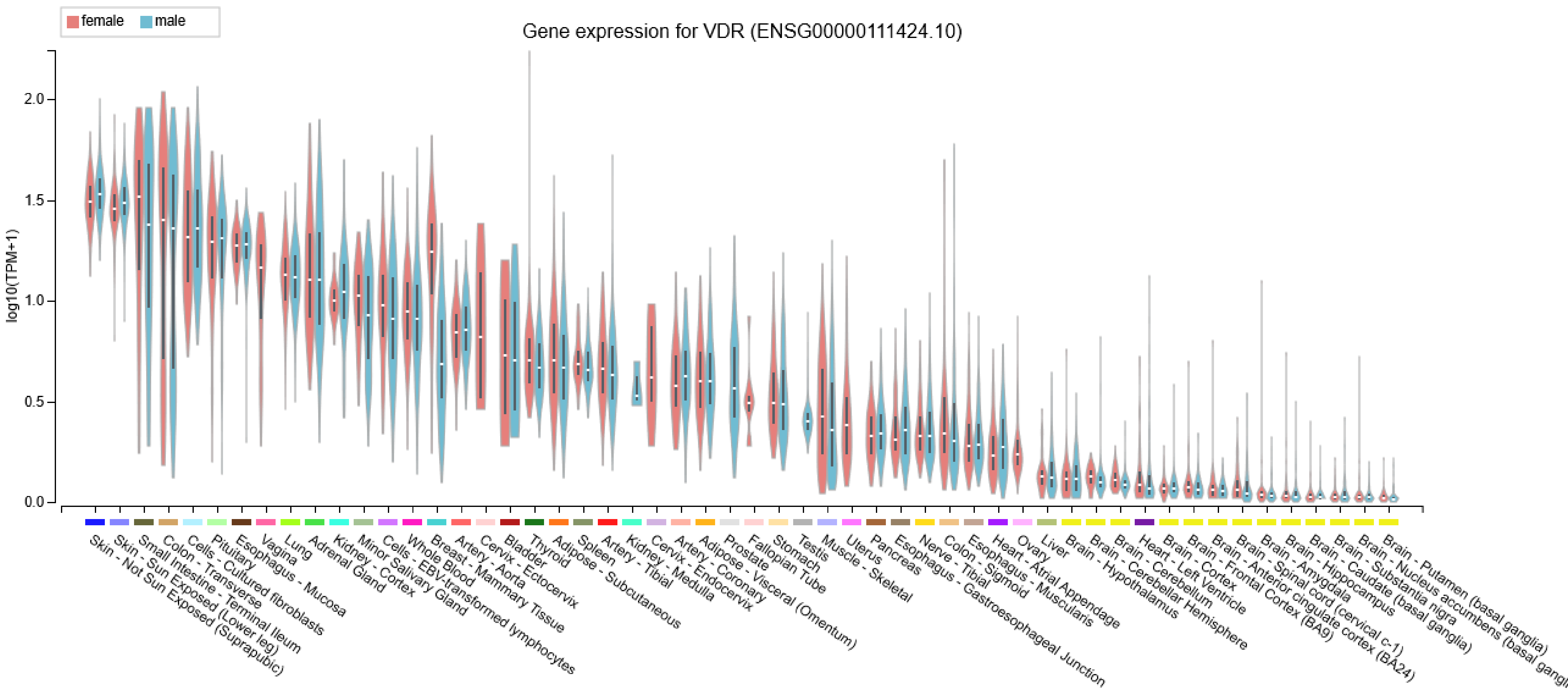

female
male
Gene expression for VDR (ENSG00000111424.10)
log10(TPM+1)
2.0
1.5
1.0
0.5
0.0
Skin - Not Sun Exposed (Suprapubic)
Skin - Sun Exposed (Lower leg)
Small Intestine - Terminal Ileum
Colon - Transverse
Cells - Cultured fibroblasts
Pituitary
Esophagus - Mucosa
Vagina
Lung
Adrenal Gland
Kidney - Cortex
Minor Salivary Gland
Cells - EBV-transformed lymphocytes
Whole Blood
Breast - Mammary Tissue
Artery - Aorta
Cervix - Ectocervix
Bladder
Thyroid
Adipose - Subcutaneous
Spleen
Artery - Tibial
Kidney - Medulla
Cervix - Endocervix
Artery - Coronary
Adipose - Visceral (Omentum)
Prostate
Fallopian Tube
Stomach
Testis
Muscle - Skeletal
Uterus
Pancreas
Esophagus - Gastroesophageal Junction
Nerve - Tibial
Colon - Sigmoid
Esophagus - Muscularis
Heart - Atrial Appendage
Ovary
Liver
Brain - Hypothalamus
Brain - Cerebellar Hemisphere
Brain - Cerebellum
Heart - Left Ventricle
Brain - Cortex
Brain - Frontal Cortex (BA9)
Brain - Anterior cingulate cortex (BA24)
Brain - Spinal cord (cervical c-1)
Brain - Amygdala
Brain - Hippocampus
Brain - Caudate (basal ganglia)
Brain - Substantia nigra
Brain - Nucleus accumbens (basal ganglia)
Brain - Putamen (basal ganglia)

ACE2 and FURIN

Supplemental Figure S7-1. Potential mechanisms affecting gene expression: Putative inhibitory role of the *VDR* gene and Vitamin D revealed by gene expression profiles in the GEO data sets

Profile: *ACE2* expression
GDS1389 / 1425103_at
Title
1alpha,25-dihydroxyvitamin D3 suppressive effect on IFN-gamma activated macrophages
Organism
Mus musculus

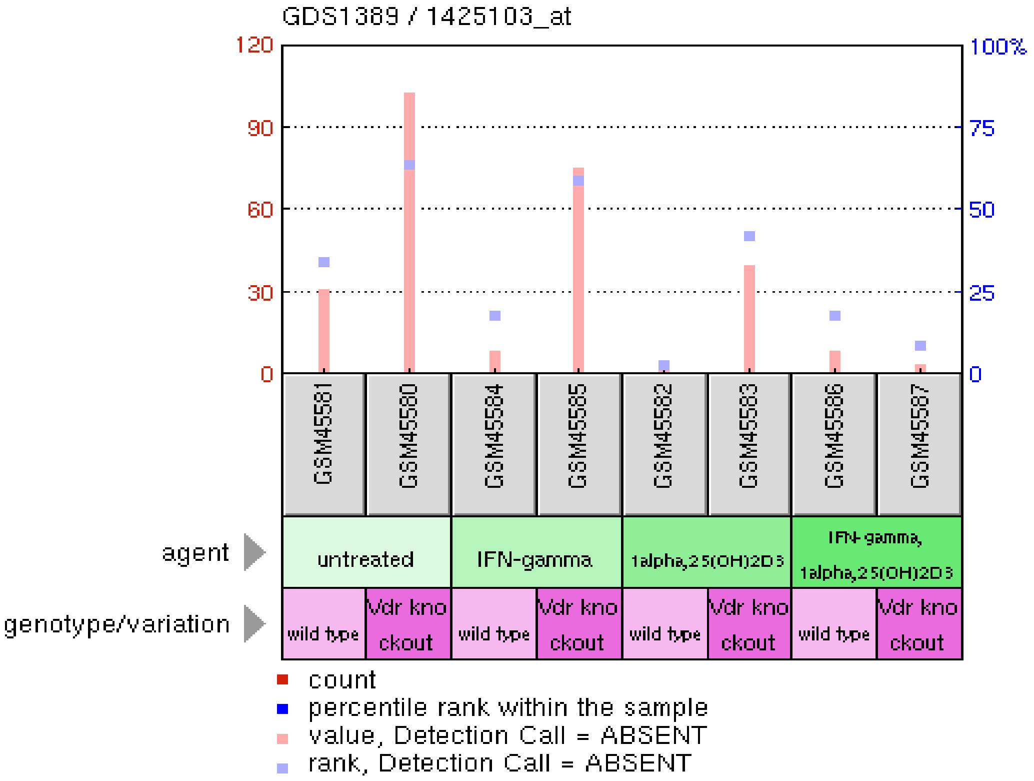

VDR depletion increases *ACE2* expression

Sample	Title	Value
GSM45581	0-WT	31.4
GSM45580	0-KO	**102.7**
GSM45584	IFN-WT	9.1
GSM45585	IFN-KO	**75.7**
GSM45582	Vit-WT	3.8
GSM45583	Vit-KO	**40.2**
GSM45586	IFN/Vit-WT	9
GSM45587	IFN/Vit-KO	4.1

VDR: a candidate inhibitor of the *ACE2* expression

GEO profile	GDS1389 / 1452138_a_at	GDS1389 / 1452138_a_at	GDS1389 / 1425103_at	GDS1389 / 1425103_at
Sample	GSM45581	GSM45580	GSM45581	GSM45580
Genotype	VDR-WT	VDR-KO	VDR-WT	VDR-KO
Expression value	27.4	59.8	31.4	102.7

GEO Profile: *ACE2* expression
GDS1389 / 1425103_at & 1452138_a_at
Title
1alpha,25-dihydroxyvitamin D3 suppressive effect on IFN-gamma activated macrophages
Organism
Mus musculus

Profile: *ACE2* expression
GDS2628 / 219962_at
Title
Vitamin D effect on bronchial smooth muscle cells
Organism
Homo sapiens

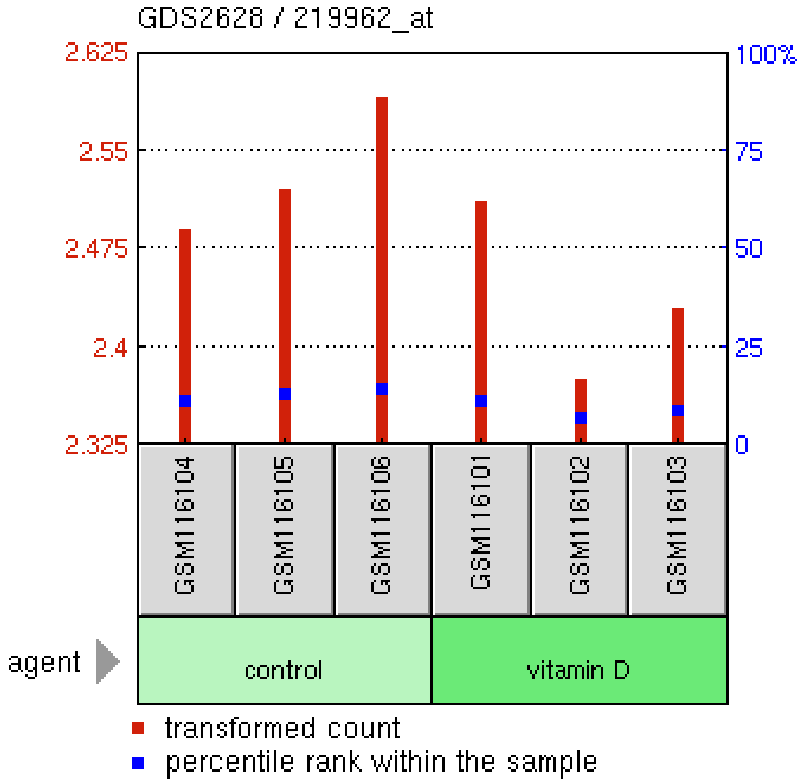

p = 0.064

Vitamin D effect on *ACE2* expression

Sample	Title	Value
GSM116104	hBSMC_control_rep1	2.49036
GSM116105	hBSMC_control_rep2	2.52156
GSM116106	hBSMC_control_rep3	2.59211
GSM116101	hBSMC_vitamin D_rep1	2.51261
GSM116102	hBSMC_vitamin D_rep2	2.37673
GSM116103	hBSMC_vitamin D_rep3	2.43005

Direct and reciprocal effects of the *VDR* gene and Vitamin D administration on expression of the *JNK1/c-FOS* pathway genes

Profile: *VDR* expression
GDS1621 / J04147_at
Title
c-Jun N-terminal kinase pathway activation and suppression effect on aortic vascular smooth muscle cells
Organism
Rattus norvegicus

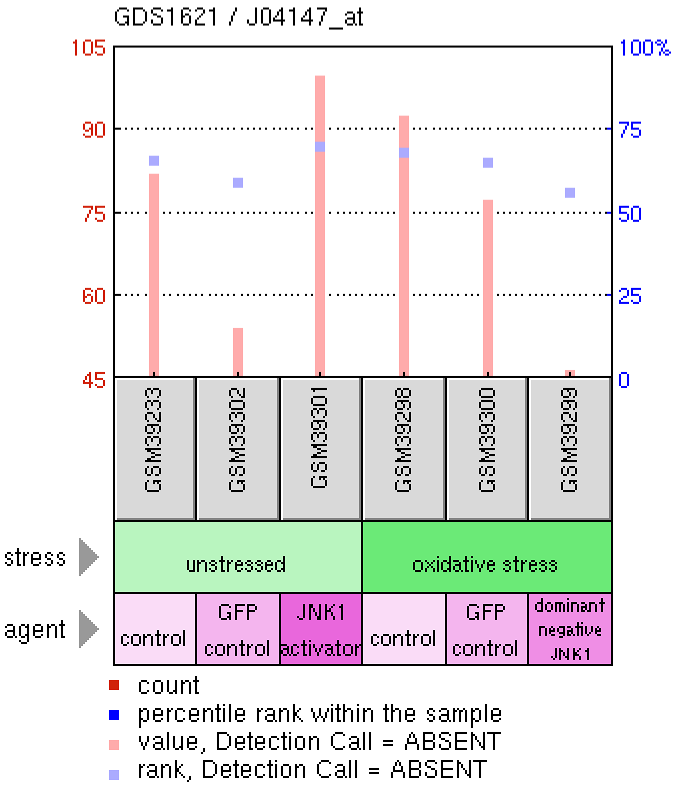

JNK1 pathway **inhibition is associated with decreased *VDR* expression, while *JNK1* activation is associated with increased *VDR* expression**

Sample	Title	Value
GSM39233	rVSMC_control	82
GSM39302	rVSMC_nEGFP	54.3
GSM39301	rVSMC_MKK7ED+JNK1WT	99.8
GSM39298	rVSMC_H2O2	92.7
GSM39300	rVSMC_H2O2+nEGFP	77.3
GSM39299	rVSMC_H2O2+APF	45.7

Profile: *c-JUN* expression
GDS2628 / 201464_x_at
Title
Vitamin D effect on bronchial smooth muscle cells
Organism
Homo sapiens

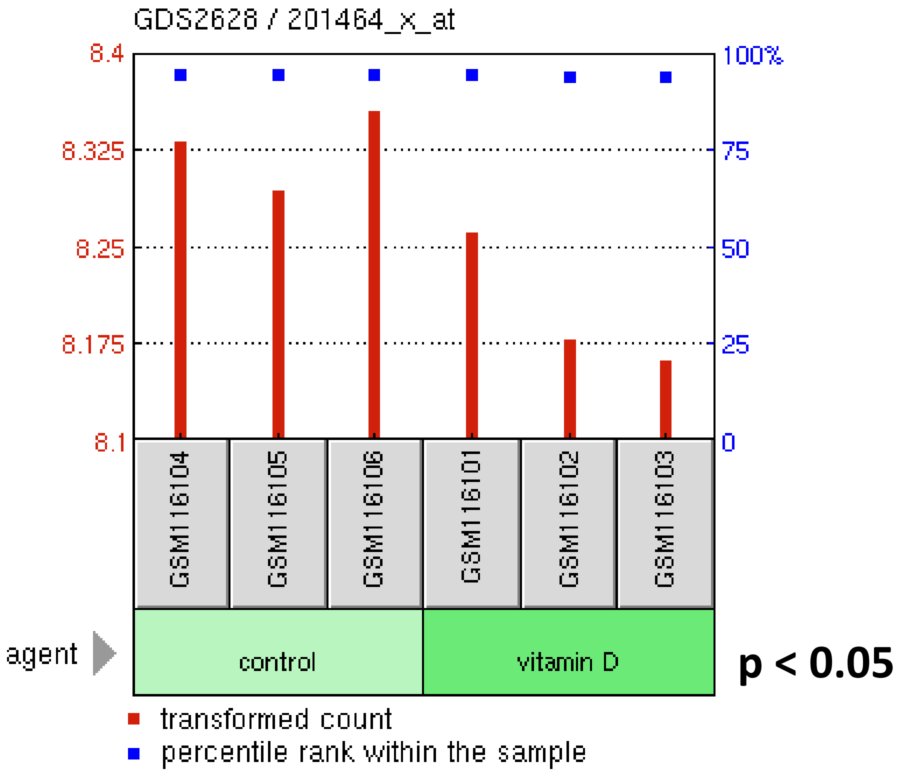

$p < 0.05$

Vitamin D inhibits *c-JUN* expression

Sample	Title	Value
GSM116104	hBSMC_control_rep1	8.33308
GSM116105	hBSMC_control_rep2	8.29401
GSM116106	hBSMC_control_rep3	8.35545
GSM116101	hBSMC_vitamin D_rep1	8.26197
GSM116102	hBSMC_vitamin D_rep2	8.17801
GSM116103	hBSMC_vitamin D_rep3	8.16188

Profile: *c-FOS* expression
GDS2628 / 209189_at
Title
Vitamin D effect on bronchial smooth muscle cells
Organism
Homo sapiens

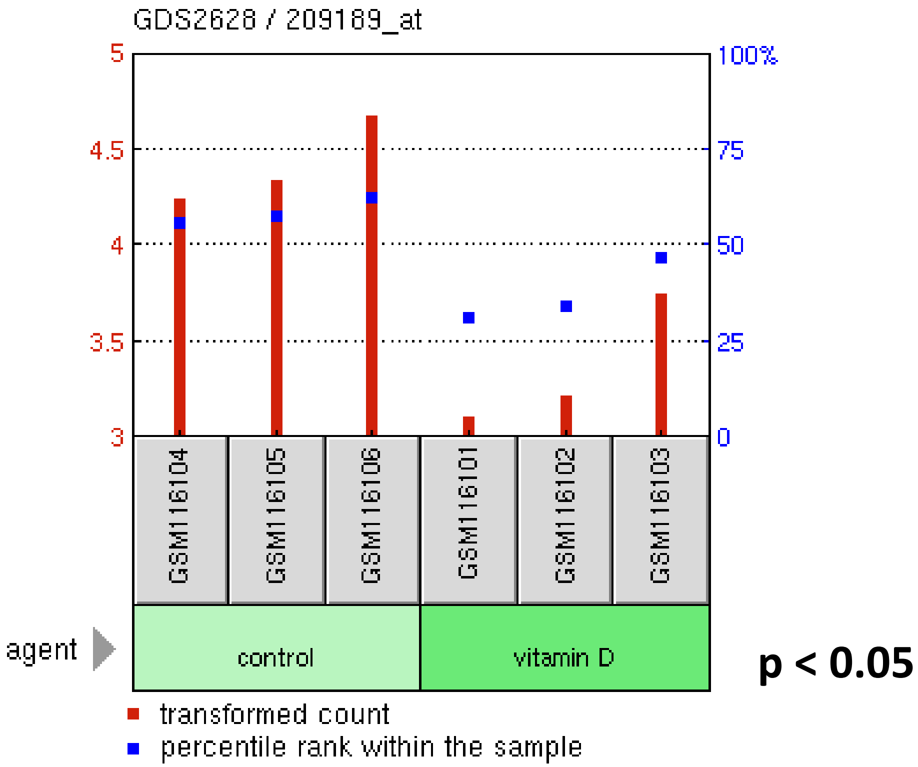

$p < 0.05$

Vitamin D inhibits *c-FOS* expression

Sample	Title	Value
GSM116104	hBSMC_control_rep1	4.24348
GSM116105	hBSMC_control_rep2	4.34194
GSM116106	hBSMC_control_rep3	4.67701
GSM116101	hBSMC_vitamin D_rep1	3.11013
GSM116102	hBSMC_vitamin D_rep2	3.21701
GSM116103	hBSMC_vitamin D_rep3	3.75394

Profile: *VDR* expression
GDS766 / 99964_at
Title
Osteoclastogenesis regulation by c-Fos and Nfat
Organism
Mus musculus

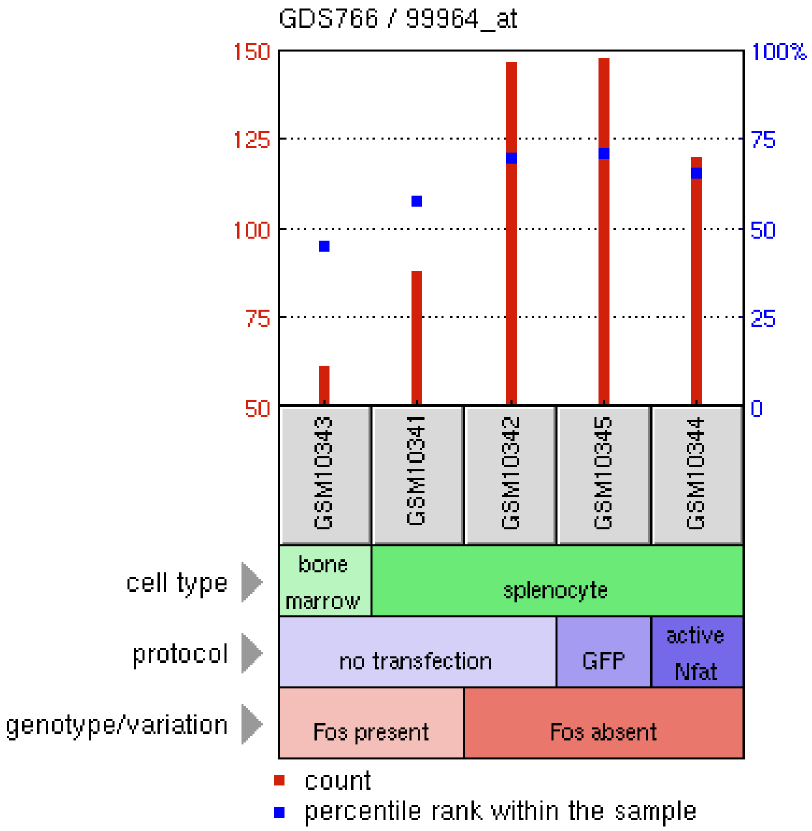

$p < 0.05$

***c-Fos* inhibits *Vdr* expression**

Sample	Title	Value
GSM10343	Fos+/+ bone-marrow	61.7
GSM10341	Fos+/+ splenocytes	88
GSM10342	Fos-/- splenocytes	146.8
GSM10345	Fos-/- splenocytes expressing GFP	147.6
GSM10344	Fos-/- splenocytes expressing deltaNFAT	120.3

JNK1/c-FOS pathway-associated activation of the *ACE2* and *FURIN* expression may trigger the auto-regulatory negative feed-back loop of the FURIN-mediated repression of the expression of *JUN, JUNB, JUND,* and *c-FOS* genes

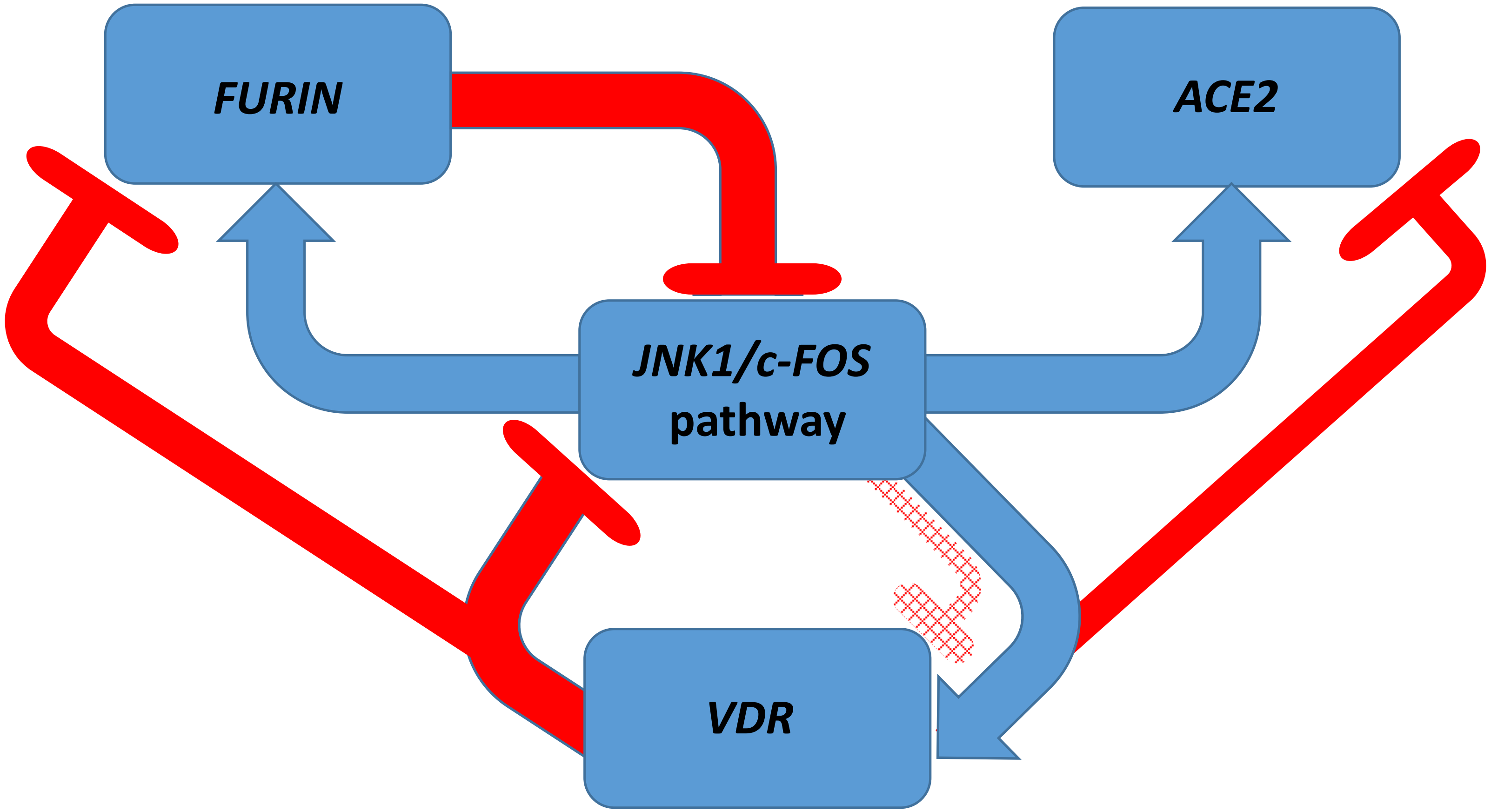

Direct and reciprocal effects of the *VDR* gene and Vitamin D administration on expression of the *HNF4a* gene

Profile: *VDR* expression
GDS5284 / 1418175_at
Title
Hepatocyte nuclear factor 4 alpha deficiency effect on the colon
Organism
Mus musculus

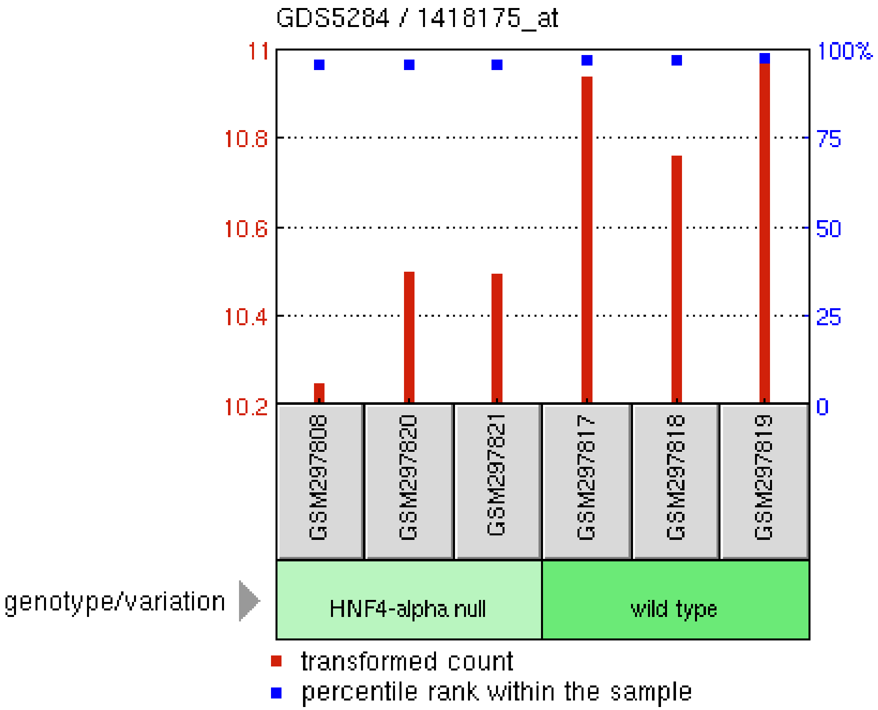

$p < 0.05$

***HNF4a* depletion inhibits *VDR* expression**

Sample	Title	Value
GSM297808	mouse colon_hnf4mutant_rep1	10.2483
GSM297820	mouse colon_hnf4mutant_rep2	10.4994
GSM297821	mouse colon_hnf4mutant_rep3	10.4987
GSM297817	mouse colon_hnf4control_rep1	10.9398
GSM297818	mouse colon_hnf4control_rep2	10.7599
GSM297819	mouse colon_hnf4control_rep3	10.9741

Profile: *VDR* expression
GDS4798 / 213692_s_at
Title
Hepatocyte nuclear factor 4 alpha depletion effect on hepatocellular carcinoma cell line
Organism
Homo sapiens

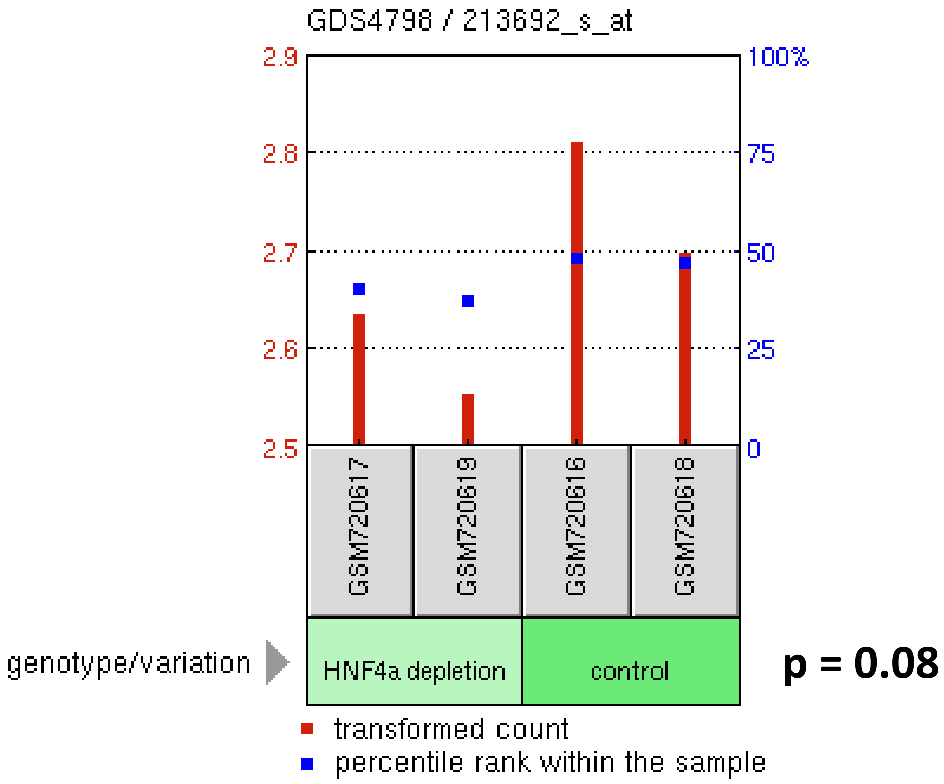

p = 0.08

***HNF4a* depletion inhibits *VDR* expression**

Sample	Title	Value
GSM720617	HepG2 cells, HNF4a RNAi treated, rep1	2.63638
GSM720619	HepG2 cells, HNF4a RNAi treated, rep2	2.5545
GSM720616	HepG2 cells, control, rep1	2.81268
GSM720618	HepG2 cells, control, rep2	2.69839

Profile: *HNF4a* expression
GDS1389 / 1450447_at
Title
1alpha,25-dihydroxyvitamin D3 suppressive effect on IFN-gamma activated macrophages
Organism
Mus musculus

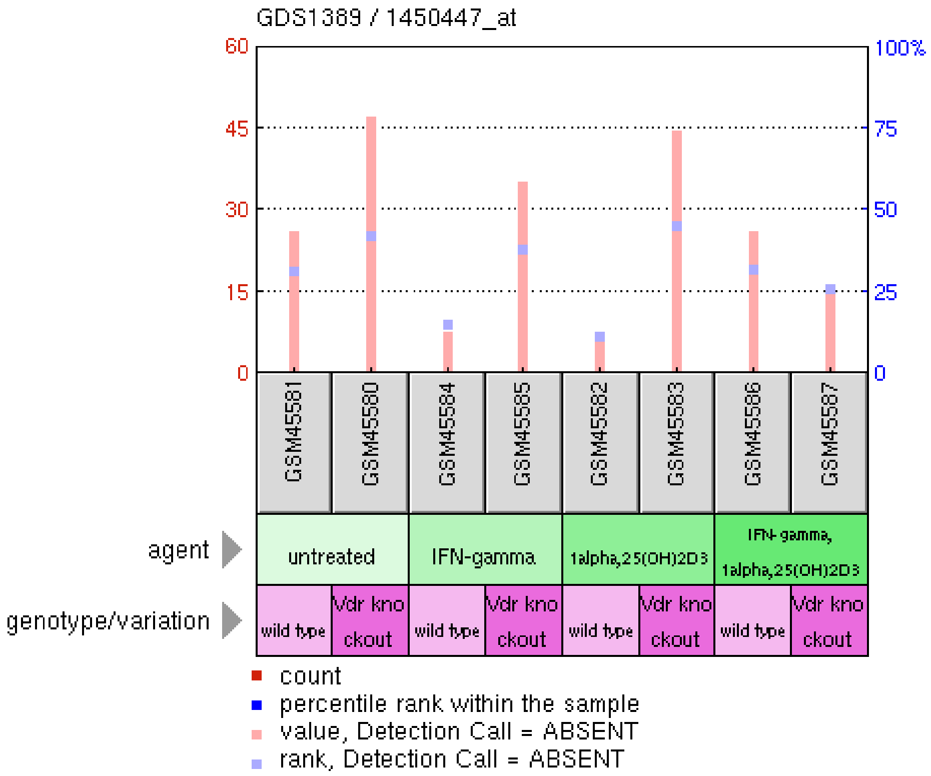

***VDR* depletion increases *HNF4a* expression**

Sample	Title	Value
GSM45581	0-WT	26.1
GSM45580	0-KO	**47.2**
GSM45584	IFN-WT	7.8
GSM45585	IFN-KO	**35.4**
GSM45582	Vit-WT	6.9
GSM45583	Vit-KO	**44.6**
GSM45586	IFN/Vit-WT	26.2
GSM45587	IFN/Vit-KO	15.1

HNF4a pathway-associated activation of the *ACE2* and *FURIN* expression may trigger the auto-regulatory positive feed-back loop of the *FURIN*-mediated activation of the *HNF4a* expression

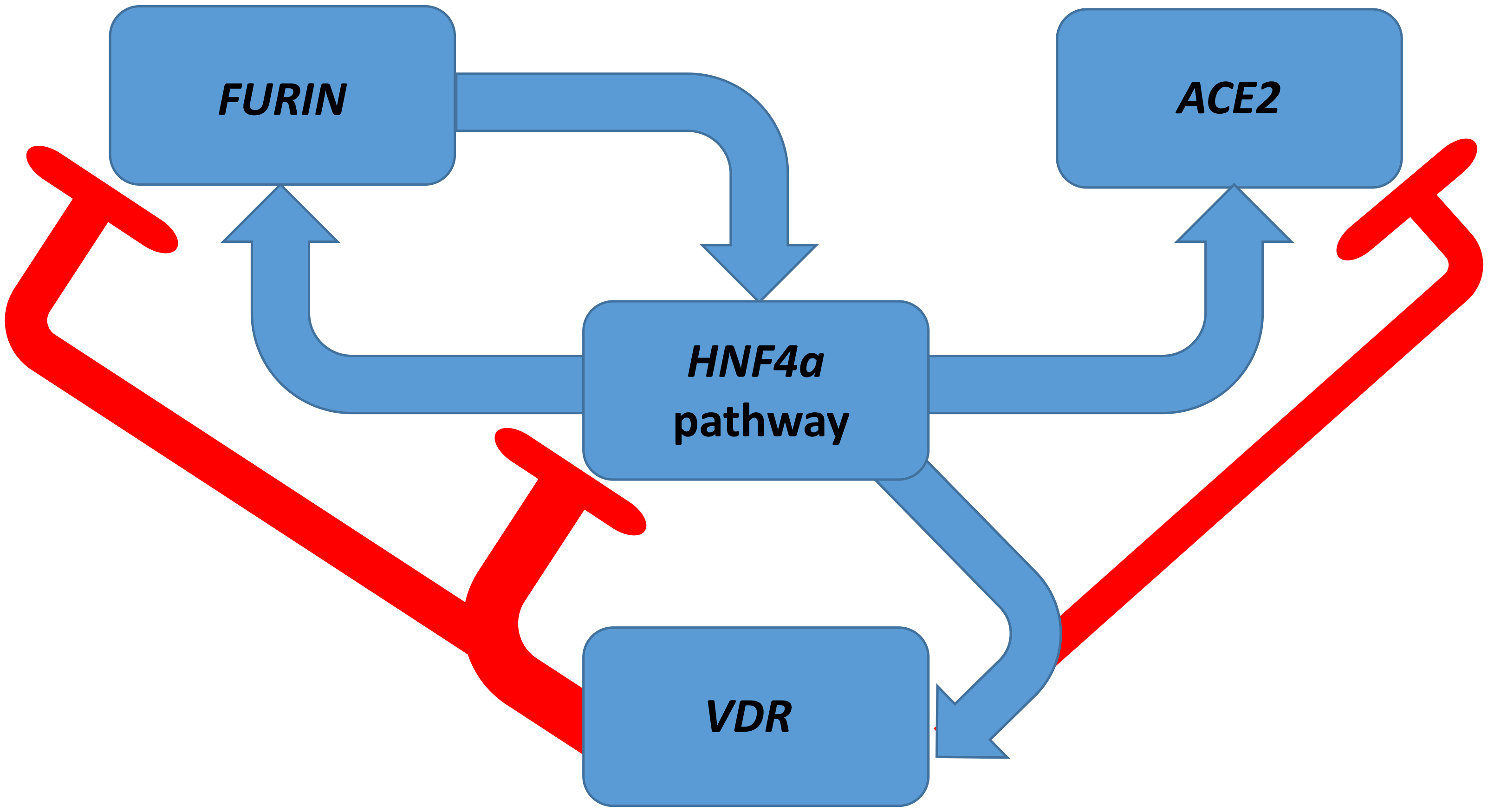

ACE2 and FURIN

Supplemental Figure S8. Potential mechanisms affecting gene expression: *HIF1A* gene product as potential repressor of the *ACE2* expression

Predominantly distinct transcription factors affect expression of the ACE2 and FURIN genes

TF Perturbations Followed by Expression

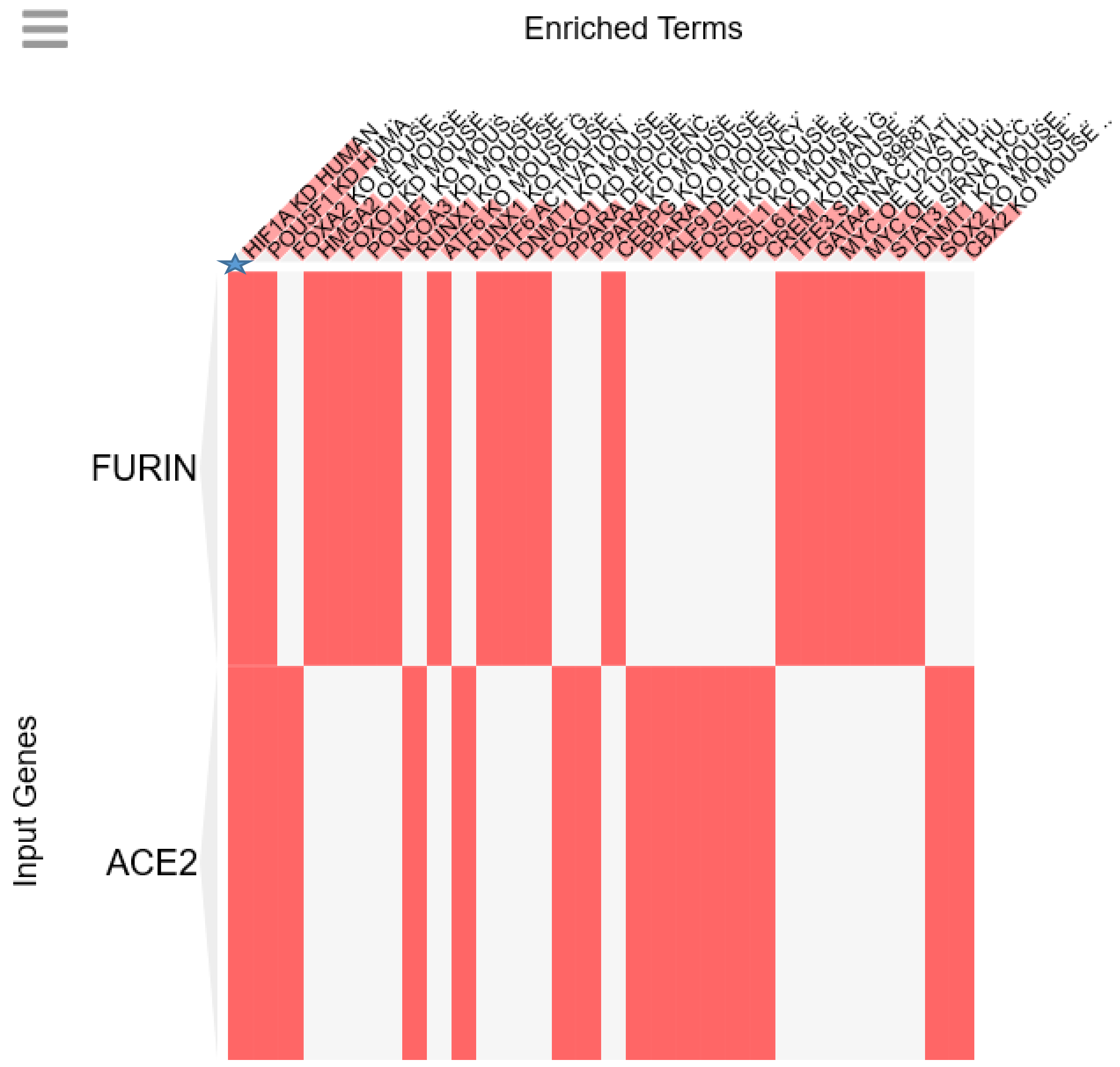

HIF1A and POU5F1: candidate inhibitors of the *ACE2* and *FURIN* expression

Gene Perturbations from GEO up

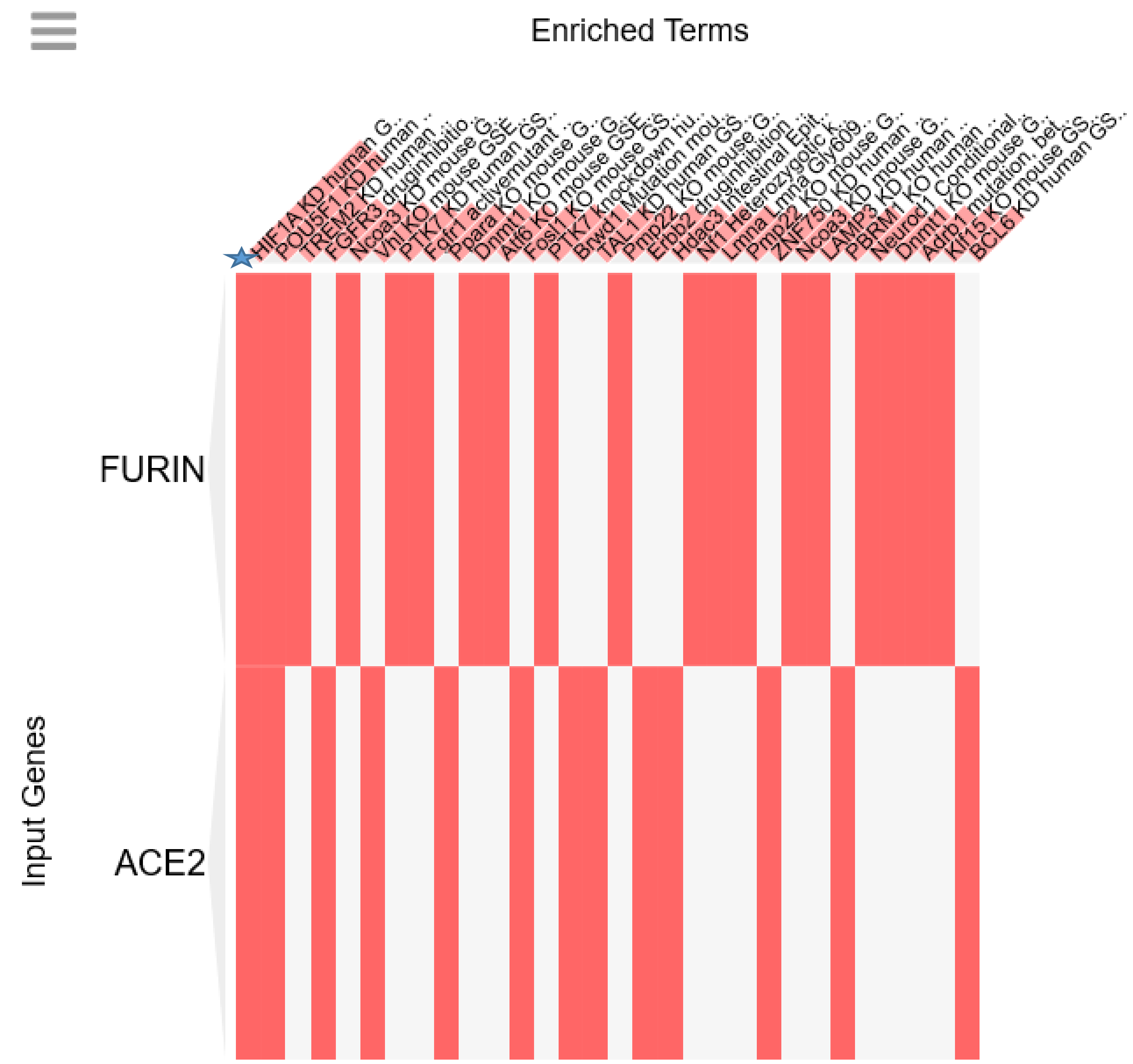

Profile: *ACE2* expression
GDS2018 / 1181882_1
Title
Hypoxia-inducible factors-1 and -2 overexpression effect on embryonic kidney cells
Organism
Homo sapiens

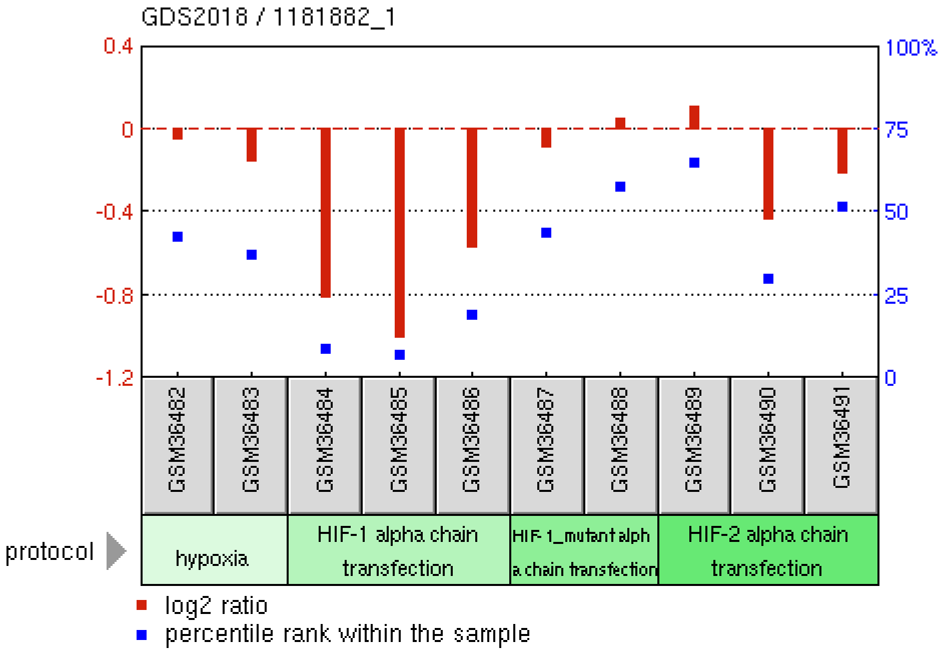

HIF1a inhibits the ACE2 expression

Sample	Title	Value
GSM36482	Nor:Hypo rep 1	-0.062
GSM36483	Nor:Hypo rep 2	-0.161
GSM36484	Nor:HIF1a rep 1	**-0.821**
GSM36485	Nor:HIF1a rep 2	**-1.013**
GSM36486	Nor:HIF1a rep 3	**-0.575**
GSM36487	Nor:HIF1amut rep 1	-0.098
GSM36488	Nor:HIF1amut rep 2	0.061
GSM36489	Nor:HIF2a rep 1	**0.115**
GSM36490	Nor:HIF2a rep 2	**-0.44**
GSM36491	Nor:HIF2a rep 3	**-0.222**

$p < 0.05$

$p < 0.05$

$p < 0.05$

Profile: *HIF1a* expression
GDS2628 / 200989_at
Title
Vitamin D effect on bronchial smooth muscle cells
Organism
Homo sapiens

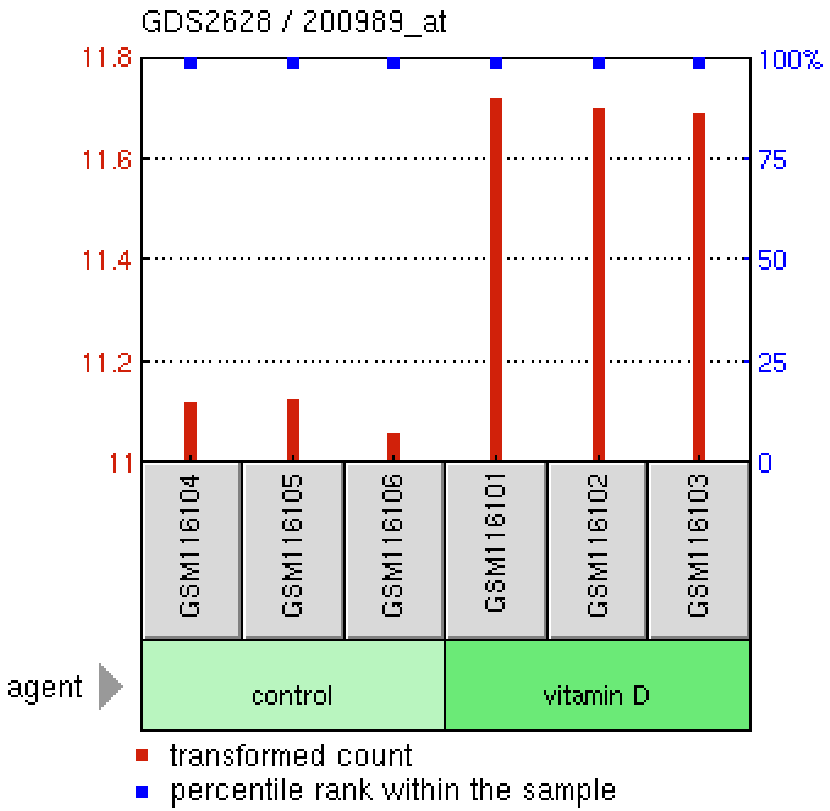

$p < 0.05$

Vitamin D enhances the *HIF1a* expression

Sample	Title	Value
GSM116104	hBSMC_control_rep1	11.1243
GSM116105	hBSMC_control_rep2	11.1271
GSM116106	hBSMC_control_rep3	11.0608
GSM116101	hBSMC_vitamin D_rep1	**11.7223**
GSM116102	hBSMC_vitamin D_rep2	**11.7034**
GSM116103	hBSMC_vitamin D_rep3	**11.6893**

Supplemental Figure S9. GSEA identify Estradiol and Quercetin as potential candidate coronavirus infection mitigation agents

Distinct patterns of drugs down-regulating expression of the *ACE2* and *FURIN* genes:

Estradiol and Quercetin emerged as promising candidate coronavirus infection mitigation agents

Drug Perturbations from GEO down

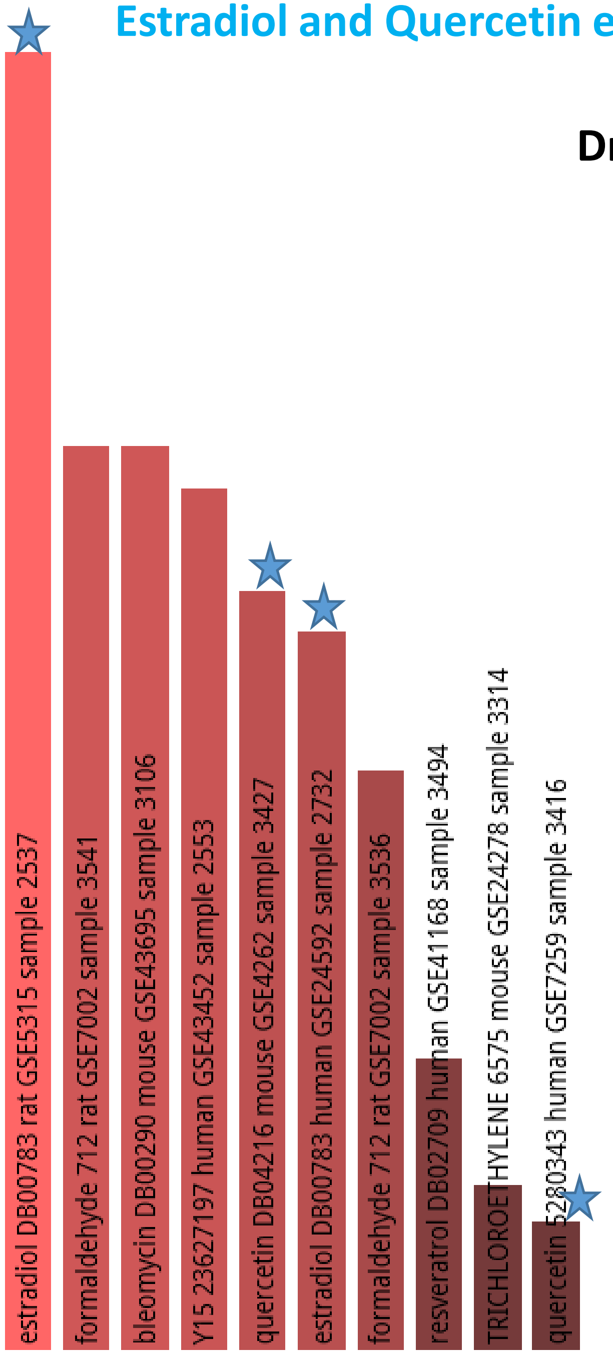

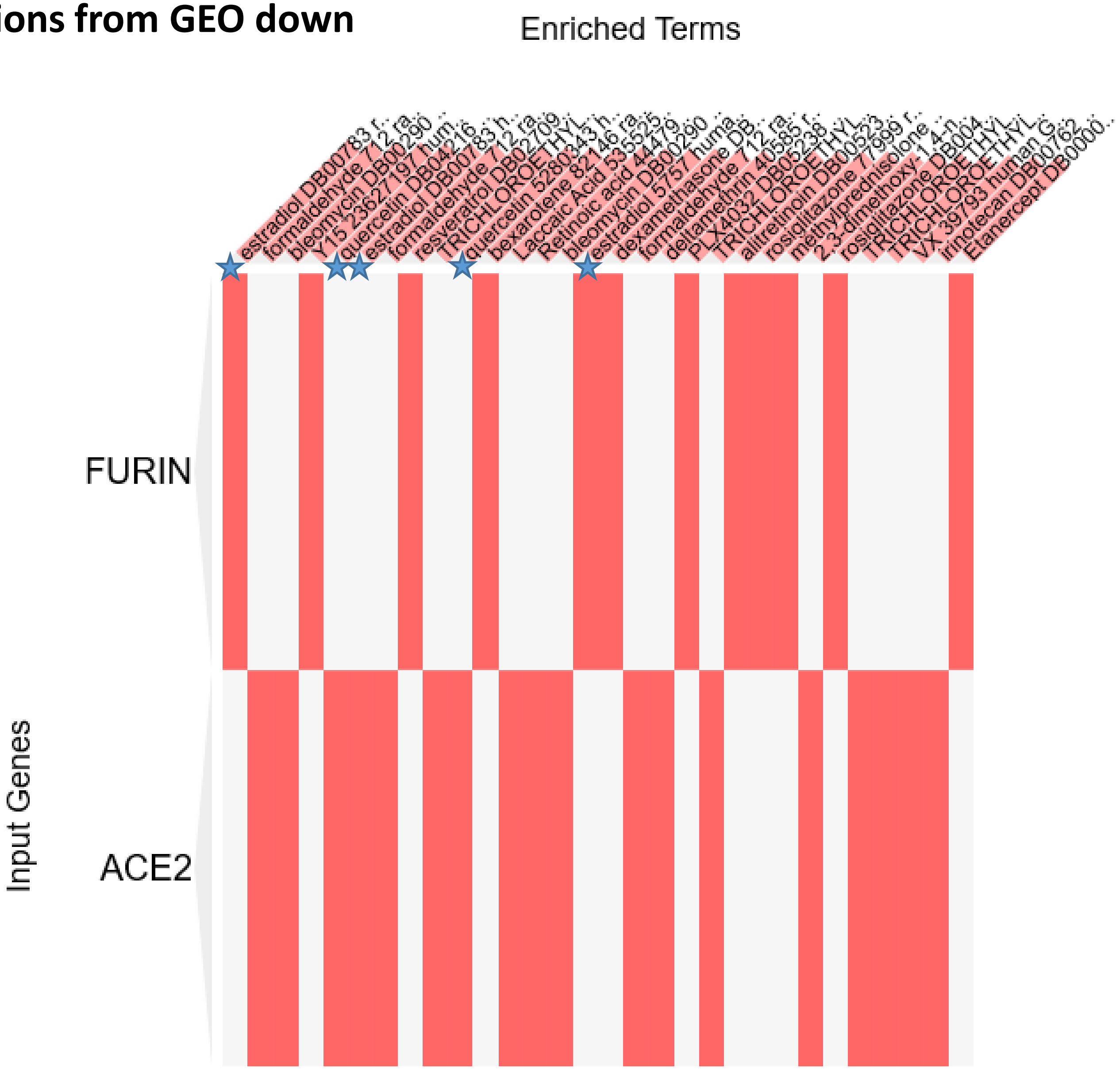

Distinct patterns of ligands down-regulating expression of the *ACE2* and *FURIN* genes

Ligand Perturbations from GEO down

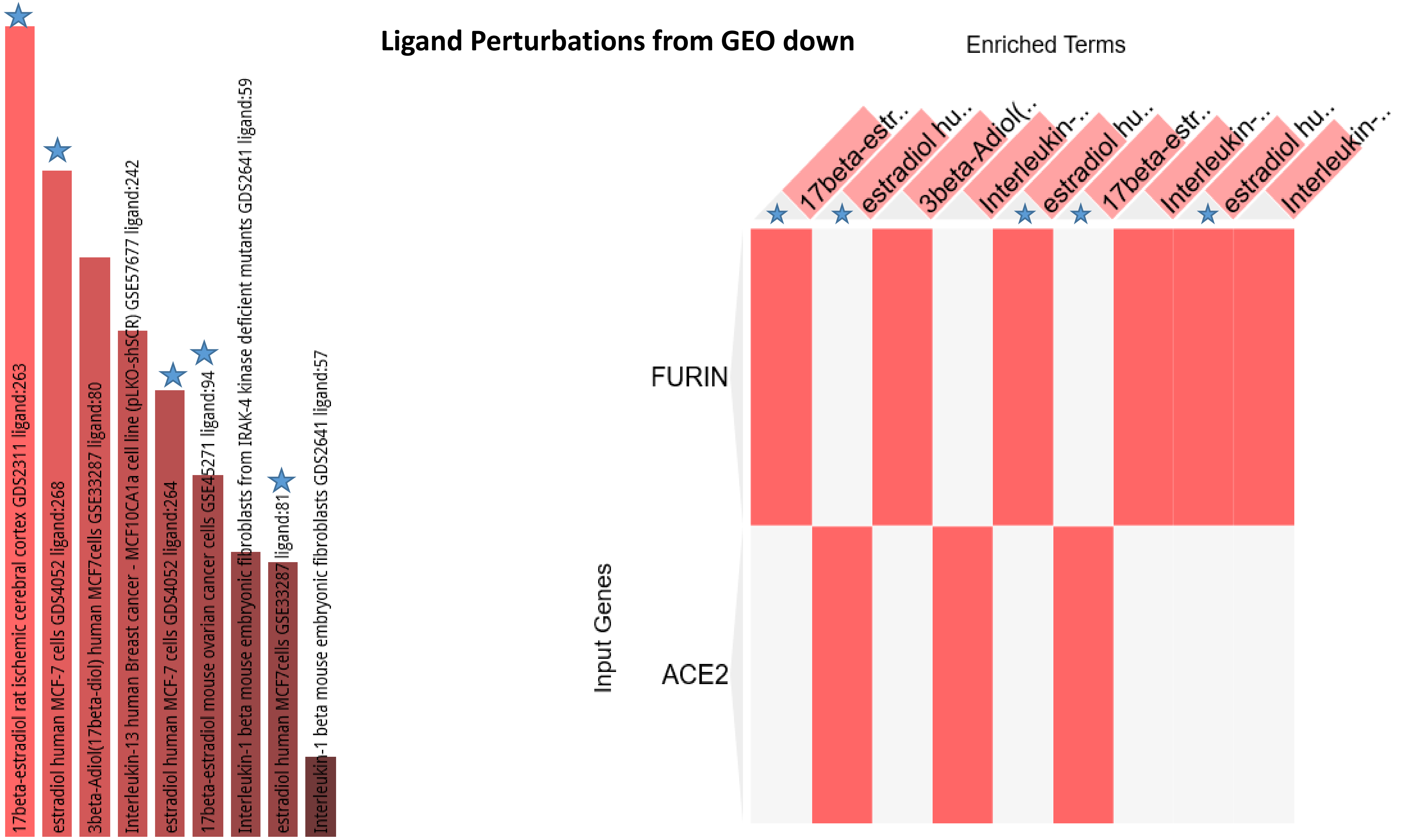

Profile: *c-FOS* expression
GDS3133 / 1375043_at
Title
Quercetin effect on the colonic mucosa
Organism
Rattus norvegicus

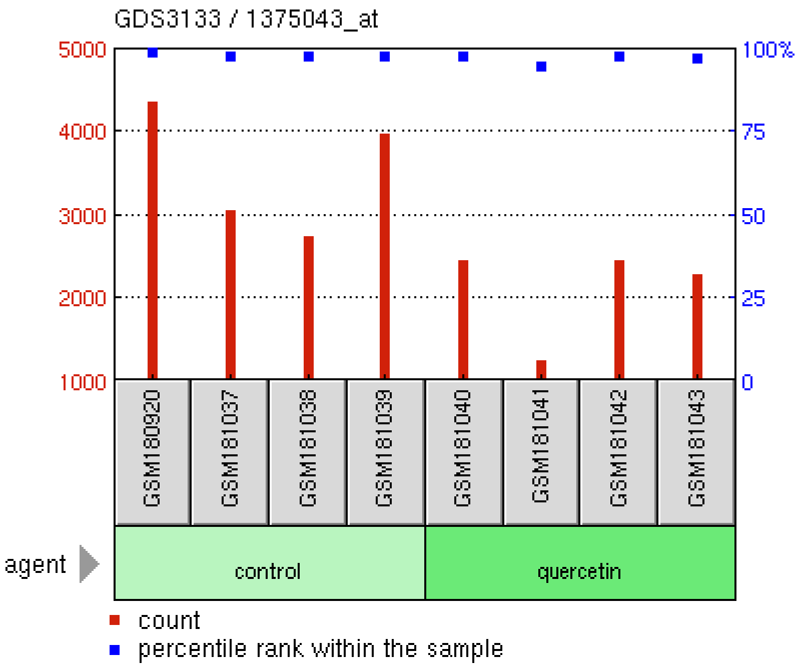

$p < 0.05$

Quercetin inhibits *c-Fos* expression

Sample	Title	Value
GSM180920	Control, sample 1	4352.72
GSM181037	Control, sample 2	3050.22
GSM181038	Control, sample 3	2743.92
GSM181039	Control, sample 4	3972.13
GSM181040	Quercetin, sample 1	2451.67
GSM181041	Quercetin, sample 2	1258.62
GSM181042	Quercetin, sample 3	2468.64
GSM181043	Quercetin, sample 4	2279.08

Profile: *c-FOS* expression
GDS3032 / 209189_at
Title
Quercetin effect on intestinal cell differentiation in vitro: time course
Organism
Homo sapiens

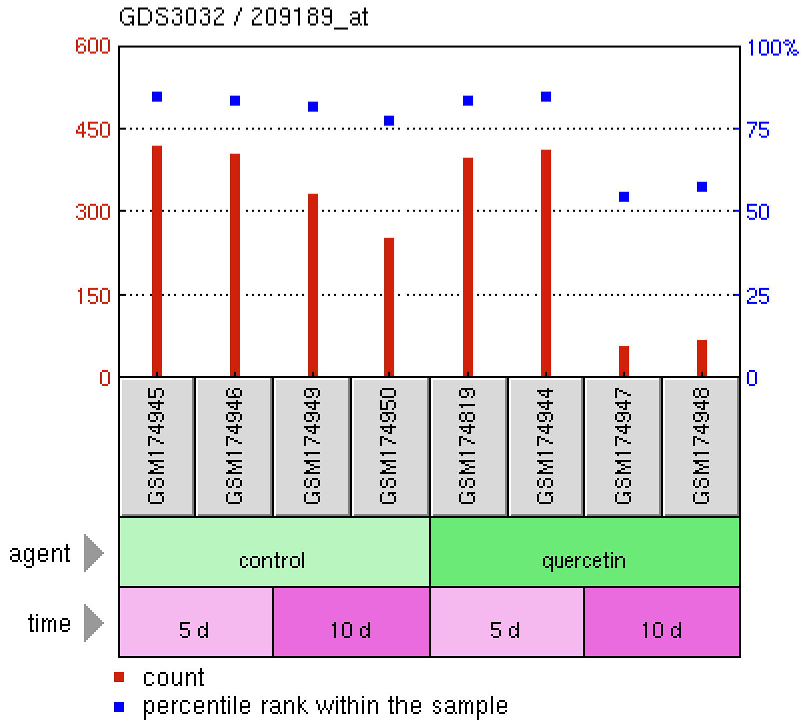

Day 10
$p < 0.05$

Quercetin inhibits *c-Fos* expression

Sample	Title	Value	
GSM174945	Control day 05 sample 1	420.956	
GSM174946	Control day 05 sample 2	404.99	
GSM174949	Control day 10 sample 1	332.662	
GSM174950	Control day 10 sample 2	256.542	
GSM174819	Quercetin day 05 sample 1	399.894	
GSM174944	Quercetin day 05 sample 2	413.261	
GSM174947	Quercetin day 10 sample 1	**60.5856**	**$p < 0.05$**
GSM174948	Quercetin day 10 sample 2	**71.7716**	

Profile: *c-FOS* expression
GDS3032 / 218881_s_at
Title
Quercetin effect on intestinal cell differentiation in vitro: time course
Organism
Homo sapiens

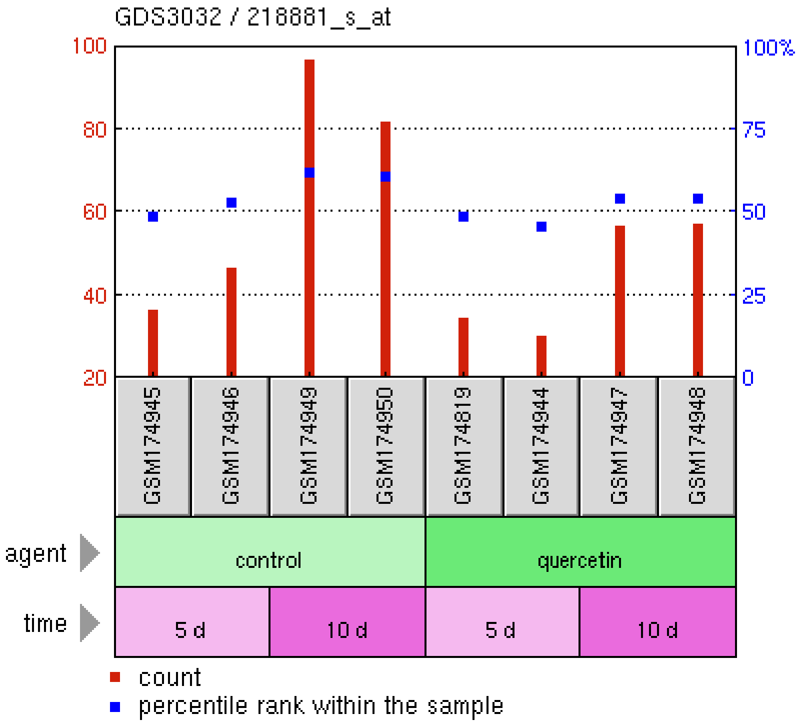

Day 10
$p < 0.05$

Quercetin inhibits *c-Fos* expression

Sample	Title	Value
GSM174945	Control day 05 sample 1	36.7758
GSM174946	Control day 05 sample 2	46.8416
GSM174949	Control day 10 sample 1	96.6905
GSM174950	Control day 10 sample 2	81.9045
GSM174819	Quercetin day 05 sample 1	34.7583
GSM174944	Quercetin day 05 sample 2	30.5511
GSM174947	Quercetin day 10 sample 1	**56.9884**
GSM174948	Quercetin day 10 sample 2	**57.2094**

Profile: *Runx1* expression
GDS3133 / 1368914_at
Title
Quercetin effect on the colonic mucosa
Organism
Rattus norvegicus

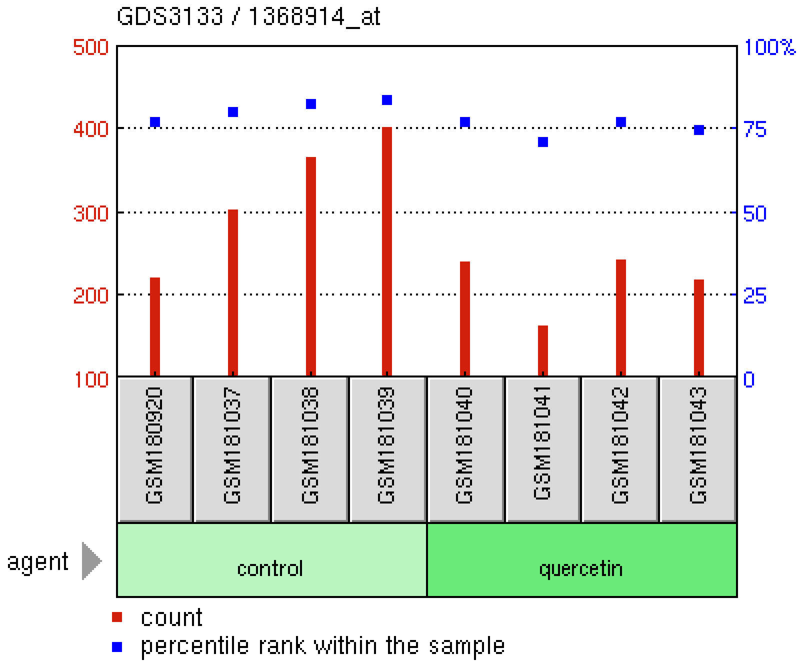

Day 10
$p < 0.05$

Quercetin inhibits *Runx1* expression

Sample	Title	Value
GSM180920	Control, sample 1	222.664
GSM181037	Control, sample 2	302.432
GSM181038	Control, sample 3	366.811
GSM181039	Control, sample 4	402.875
GSM181040	Quercetin, sample 1	241.637
GSM181041	Quercetin, sample 2	163.728
GSM181042	Quercetin, sample 3	**243.316**
GSM181043	Quercetin, sample 4	**218.646**

Profile: *Runxt1* expression
GDS3676 / 205528_s_at
Title
Quercetin effect on CD14+ monocyte
Organism
Homo sapiens

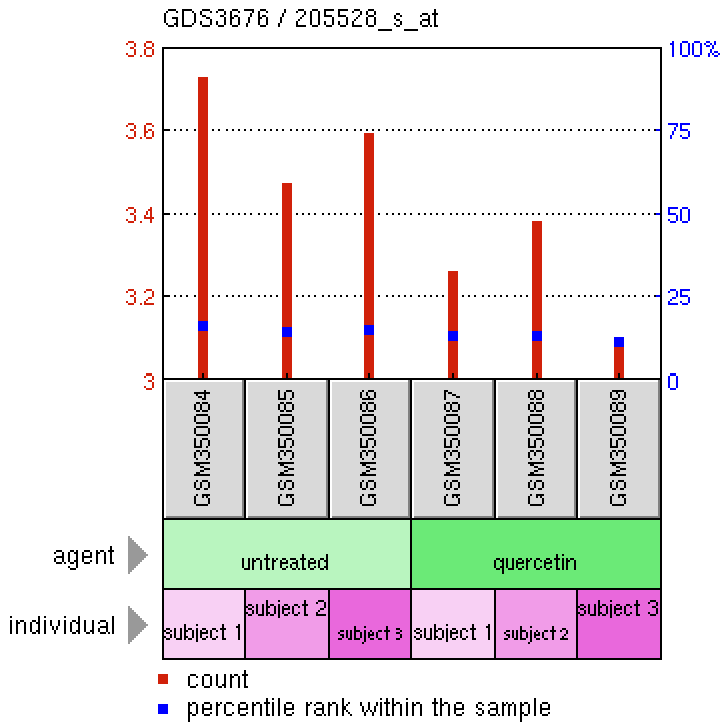

$p < 0.05$

Quercetin inhibits *Runxt1* expression

Sample	Title	Value
GSM350084	CD14+ at baseline, biological replicate 1	3.72993
GSM350085	CD14+ at baseline, biological replicate 2	3.47495
GSM350086	CD14+ at baseline, biological replicate 3	3.59744
GSM350087	CD14+ after 2 wk quercetin supplementation, biological replicate 1	3.2641
GSM350088	CD14+ after 2 wk quercetin supplementation, biological replicate 2	3.38173
GSM350089	CD14+ after 2 wk quercetin supplementation, biological replicate 3	3.10117

RUNXT1, RUNX1 translocation partner 1

Profile: *HNF4a* expression
GDS3032 / 216889_s_at
Title
Quercetin effect on intestinal cell differentiation in vitro: time course
Organism
Homo sapiens

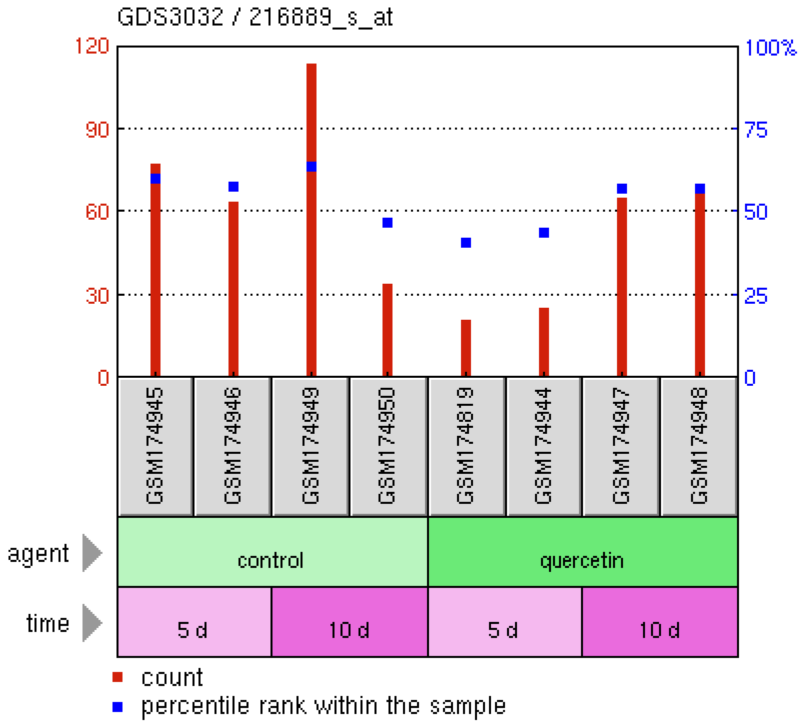

Day 5
$p < 0.05$

Quercetin inhibits *HNF4a* expression

Sample	Title	Value
GSM174945	Control day 05 sample 1	**77.6175**
GSM174946	Control day 05 sample 2	**63.792**
GSM174949	Control day 10 sample 1	113.821
GSM174950	Control day 10 sample 2	34.1332
GSM174819	Quercetin day 05 sample 1	**21.0188**
GSM174944	Quercetin day 05 sample 2	**25.7496**
GSM174947	Quercetin day 10 sample 1	65.2834
GSM174948	Quercetin day 10 sample 2	69.9663

Profile: *c-FOS* expression
GDS2708 / 8494
Title
Quercetin effect on cultured cardiomyocytes: time course
Organism
Rattus norvegicus

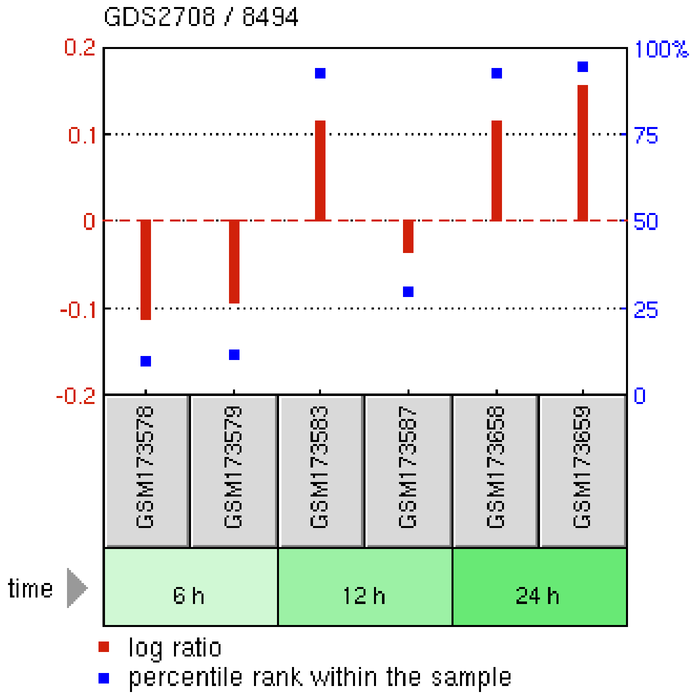

24 hrs
$p < 0.05$

Quercetin increases *c-Fos* expression

Sample	Title	Value
GSM173578	Quercetin treated cardiomyocytes for 6 hours (Q6-A).	-0.114041
GSM173579	Quercetin treated cardiomyocytes for 6 hours (Q6-B).	-0.0962766
GSM173583	Quercetin treated cardiomyocytes for 12 hours (Q12-A).	0.118043
GSM173587	Quercetin treated cardiomyocytes for 12 hours (Q12-B).	-0.0368638
GSM173658	Quercetin treated cardiomyocytes for 24 hours (Q24-A).	0.118043
GSM173659	Quercetin treated cardiomyocytes for 24 hours (Q24-B).	0.15779

Supplemental Figure S10. Estradiol and Quercetin manifest activities as potential candidate coronavirus infection mitigation agents

Estradiol effect on *FURIN* expression in the rat ischemic brain

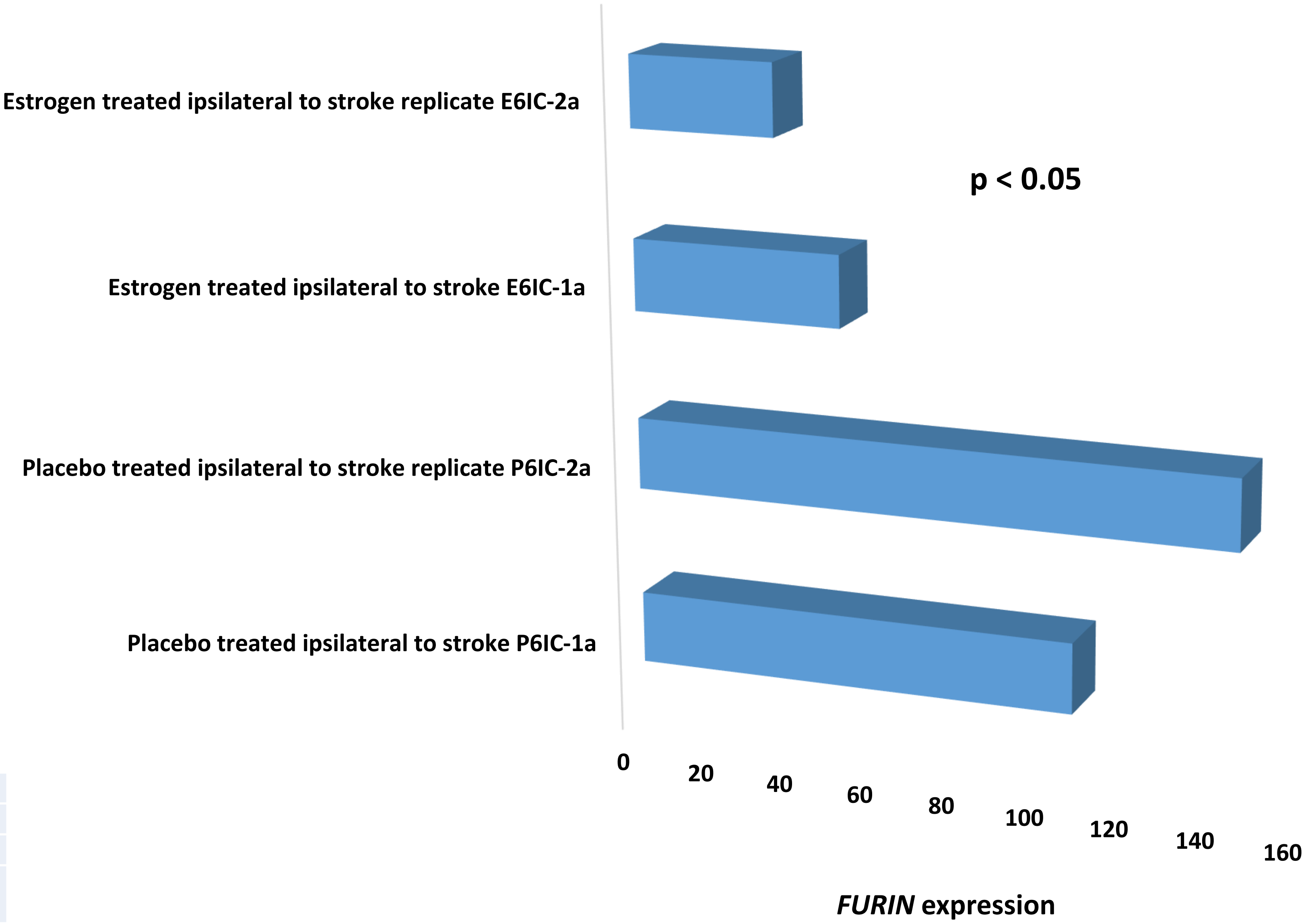

GEO Profile
GDS2311 / X55660_at
Title
Estradiol effect on the ischemic brain
Organism
Rattus norvegicus

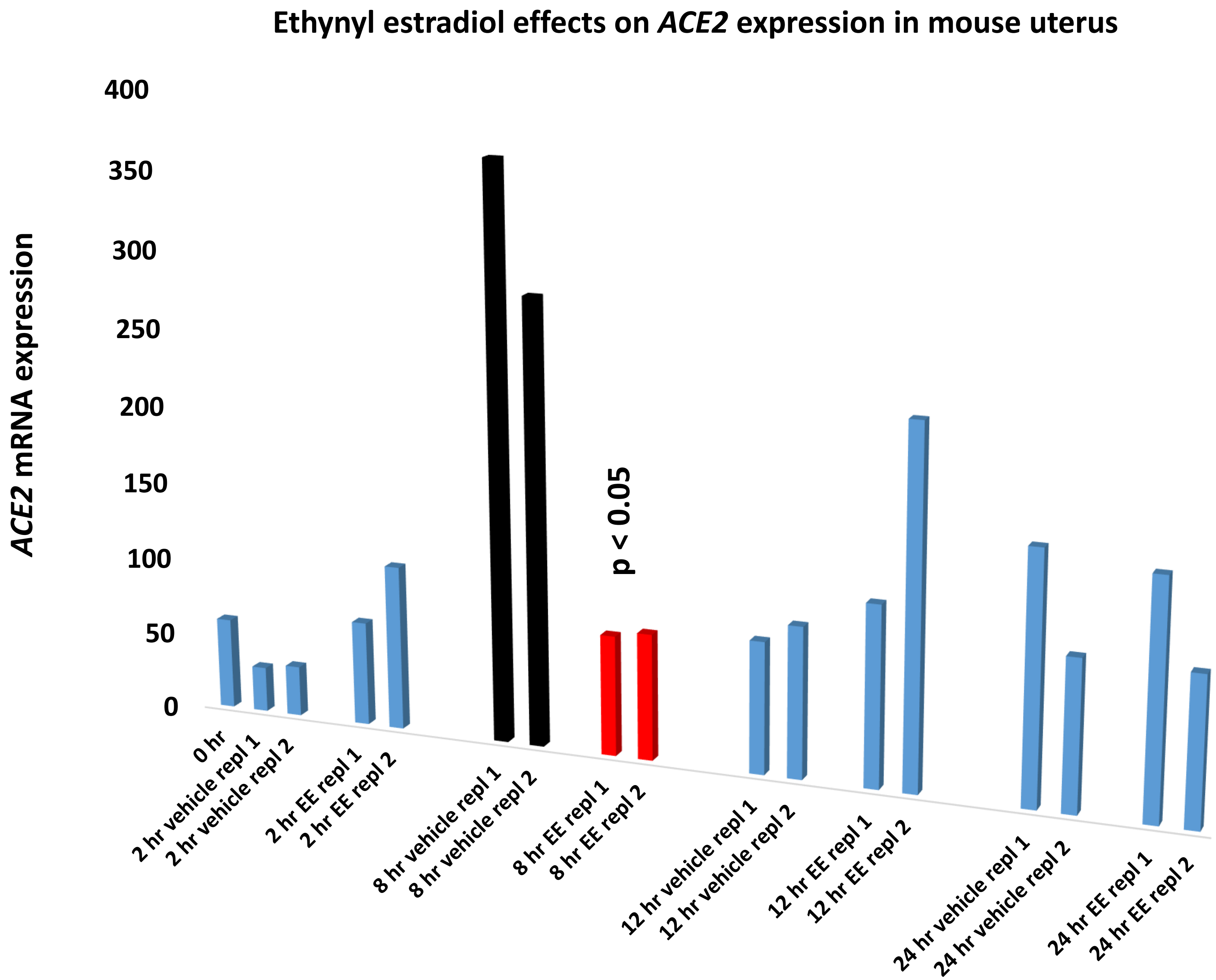

GEO Profile
GDS285 / AA690434_at
Title
Ethynyl estradiol estrogen effect in uterus
Organism
Mus musculus

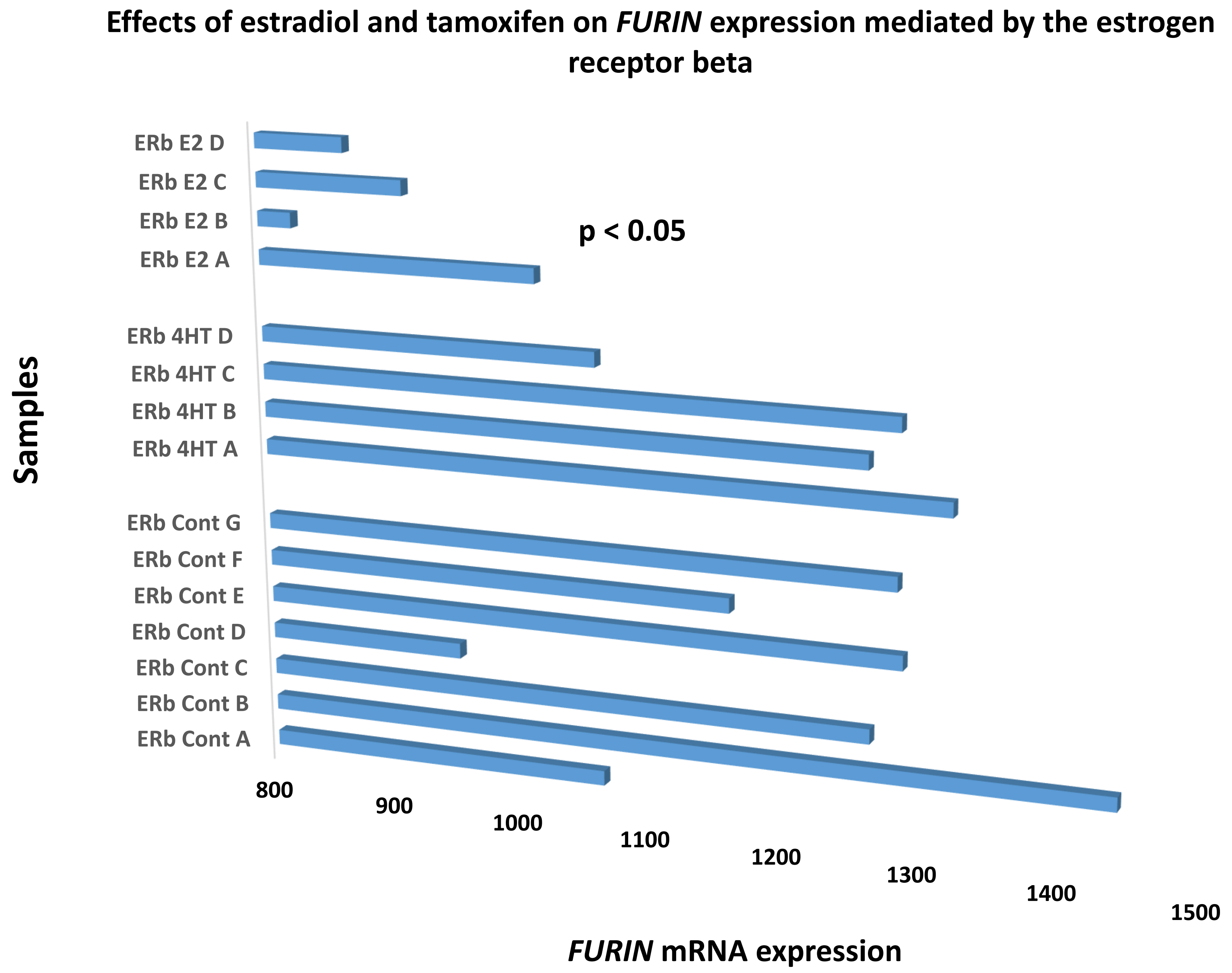

GEO Profile
GDS1094 / 201945_at
Title
Estrogen receptor alpha/beta heterodimer action in response to estrogen and tamoxifen
Organism
Homo sapiens

Effects of estradiol and tamoxifen on ACE2 expression mediated by the estrogen receptor beta

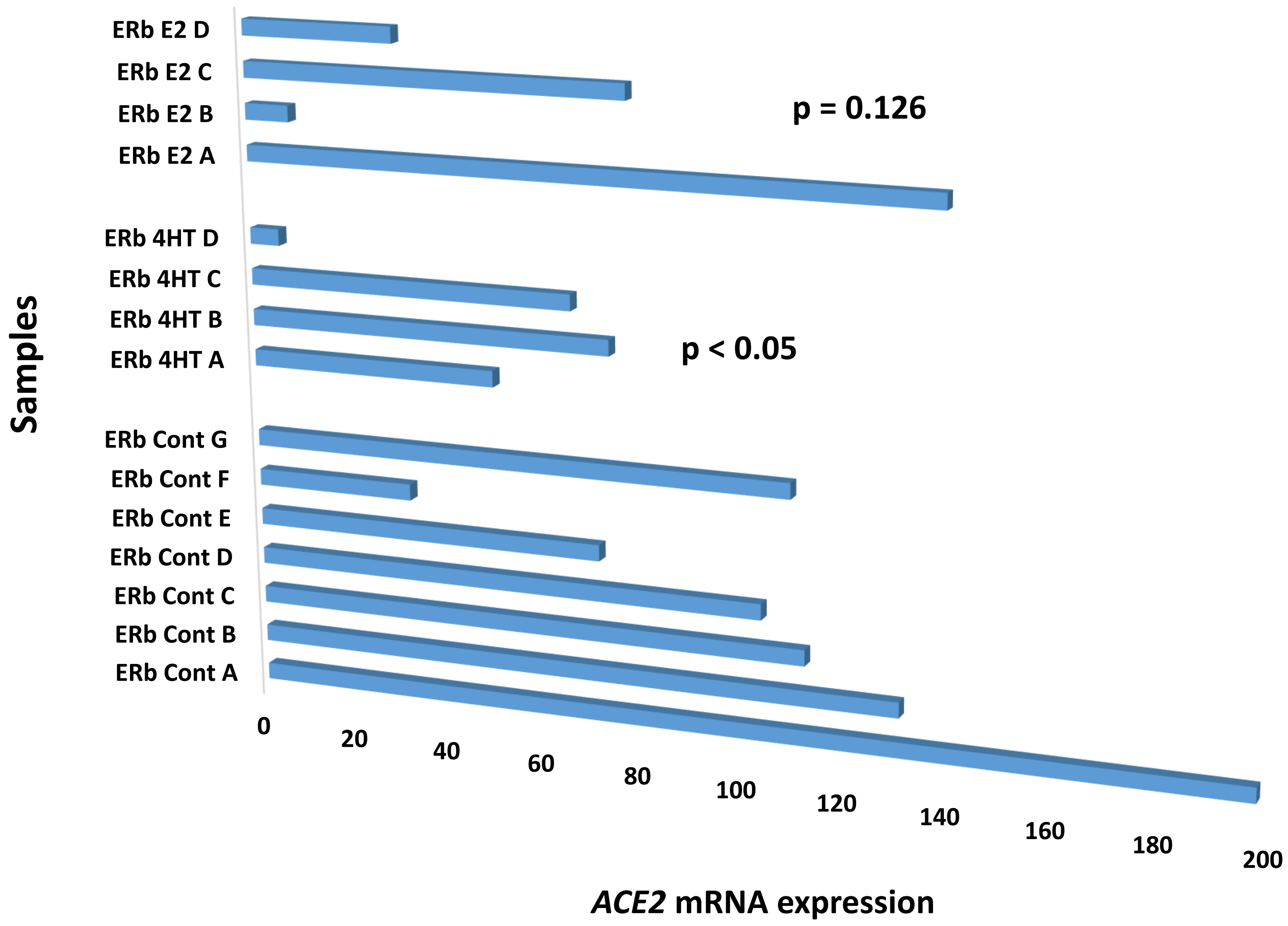

GEO Profile
GDS1094 / 219962_at
Title
Estrogen receptor alpha/beta heterodimer action in response to estrogen and tamoxifen
Organism
Homo sapiens

Profile: ACE2 expression
GDS3032 / 222257_s_at
Title
Quercetin effect on intestinal cell differentiation in vitro: time course
Organism
Homo sapiens

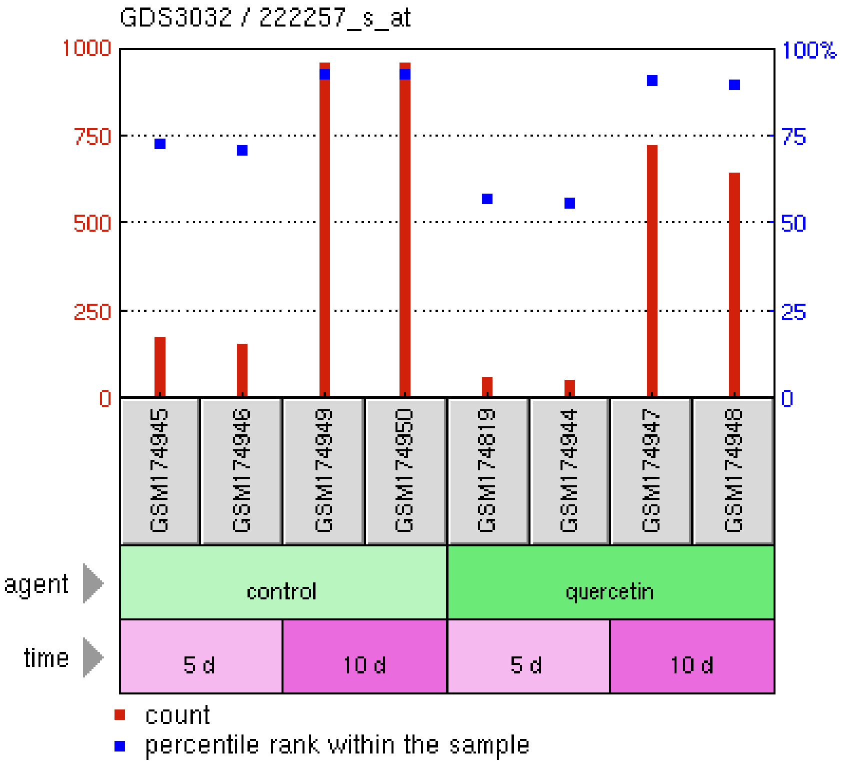

Day 5
$p < 0.05$

Day 10
$p < 0.05$

Quercetin inhibits the *ACE2* expression

Sample	Title	Value
GSM174945	Control day 05 sample 1	176.763
GSM174946	Control day 05 sample 2	157.459
GSM174949	Control day 10 sample 1	962.644
GSM174950	Control day 10 sample 2	959.364
GSM174819	Quercetin day 05 sample 1	60.6977
GSM174944	Quercetin day 05 sample 2	59.0821
GSM174947	Quercetin day 10 sample 1	725.488
GSM174948	Quercetin day 10 sample 2	649.304

Profile: ACE2 expression
GDS3032 / 219962_at
Title
Quercetin effect on intestinal cell differentiation in vitro: time course
Organism
Homo sapiens

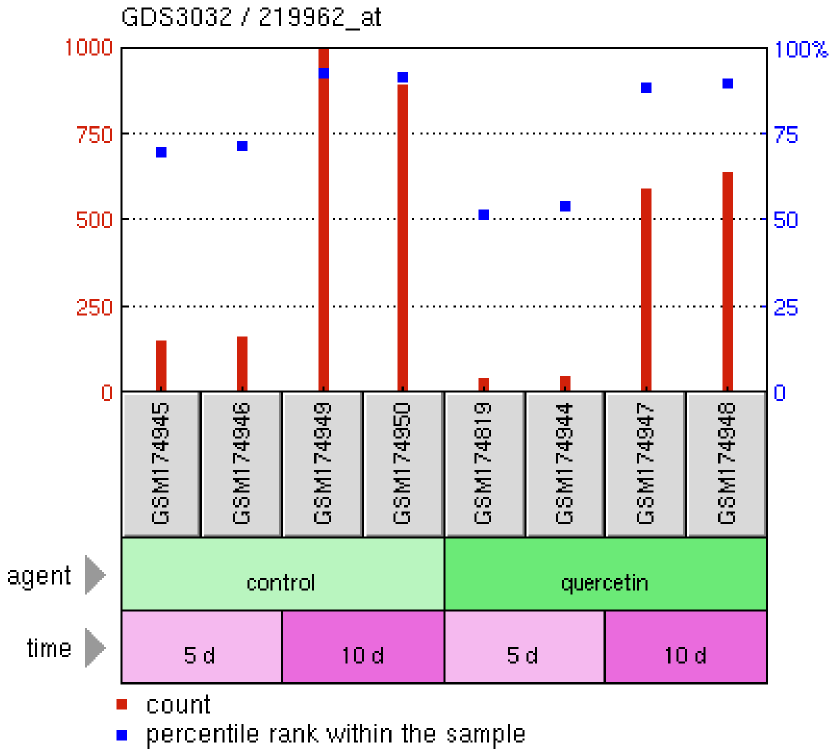

Day 5
$p < 0.05$

Day 10
$p < 0.05$

Quercetin inhibits the *ACE2* expression

Sample	Title	Value
GSM174945	Control day 05 sample 1	152.856
GSM174946	Control day 05 sample 2	163.228
GSM174949	Control day 10 sample 1	995.184
GSM174950	Control day 10 sample 2	895.468
GSM174819	Quercetin day 05 sample 1	43.9228
GSM174944	Quercetin day 05 sample 2	49.8735
GSM174947	Quercetin day 10 sample 1	595.486
GSM174948	Quercetin day 10 sample 2	642.974

Profile: *c-FOS* expression
GDS5278 / MmugDNA.38565.1.S1_at
Title
Endometrium response to tamoxifen and low dose estradiol combination therapy
Organism: Macaca mulatta

Estradiol inhibits the *c-FOS* expression

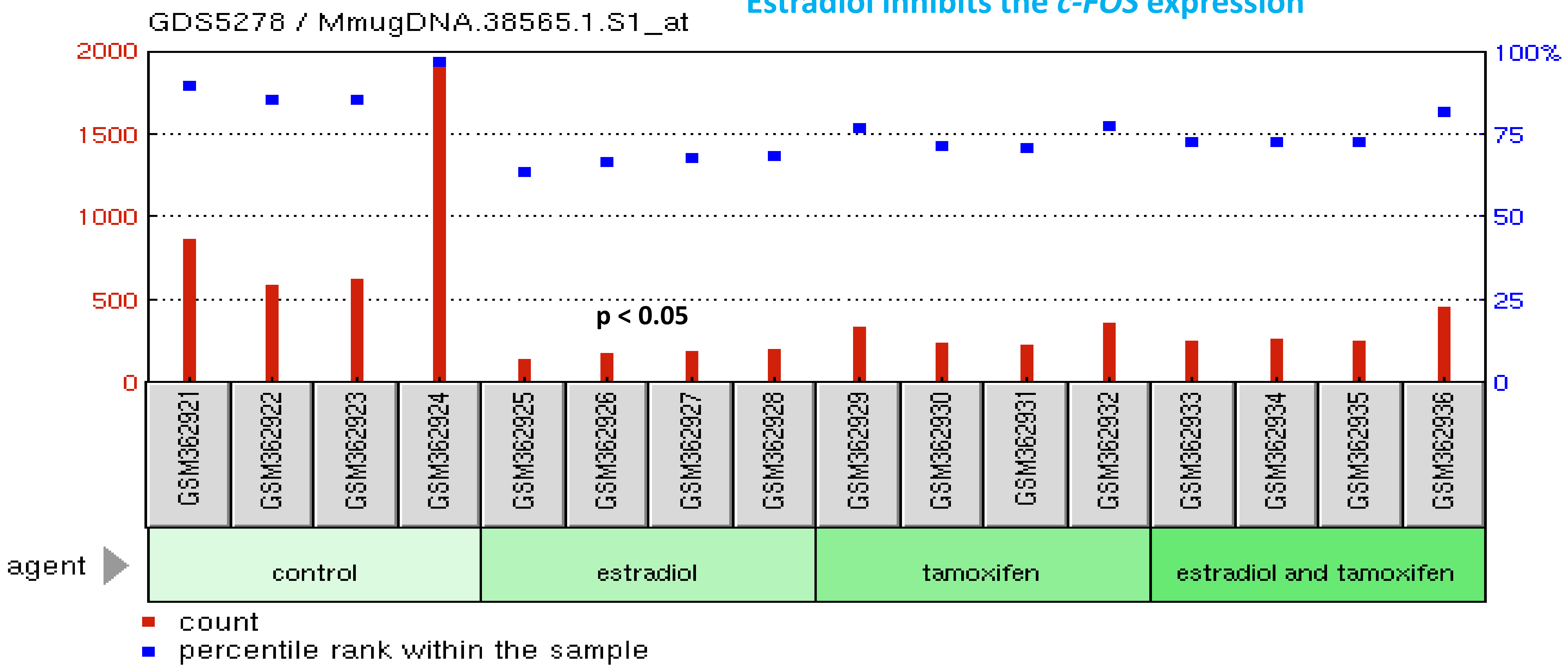

Profile: *c-FOS* expression
GDS5278 / MmugDNA.38565.1.S1_at
Title
Endometrium response to tamoxifen and low dose estradiol combination therapy
Organism: Macaca mulatta

Estradiol inhibits the *c-FOS* expression

Sample	Title	Value
GSM362921	Control 1	**868.444**
GSM362922	Control 2	**596.312**
GSM362923	Control 3	**633.478**
GSM362924	Control 4	**1956.61**
GSM362925	Estradiol 1	147.047 **$p < 0.05$**
GSM362926	Estradiol 2	183.666
GSM362927	Estradiol 3	194.779
GSM362928	Estradiol 4	212.397
GSM362929	Tamoxifen 1	347.062
GSM362930	Tamoxifen 2	241.809
GSM362931	Tamoxifen 3	238.437
GSM362932	Tamoxifen 4	363.108
GSM362933	Estradiol and Tamoxifen 1	255.806
GSM362934	Estradiol and Tamoxifen 2	272.225
GSM362935	Estradiol and Tamoxifen 3	264.55
GSM362936	Estradiol and Tamoxifen 4	459.833

Profile: *c-FOS* expression
GDS982 / 160901_at
Title
Uterine response to physiologic, plant-derived, and synthetic estrogen
Organism: Mus musculus

Estradiol increases the *c-FOS* expression

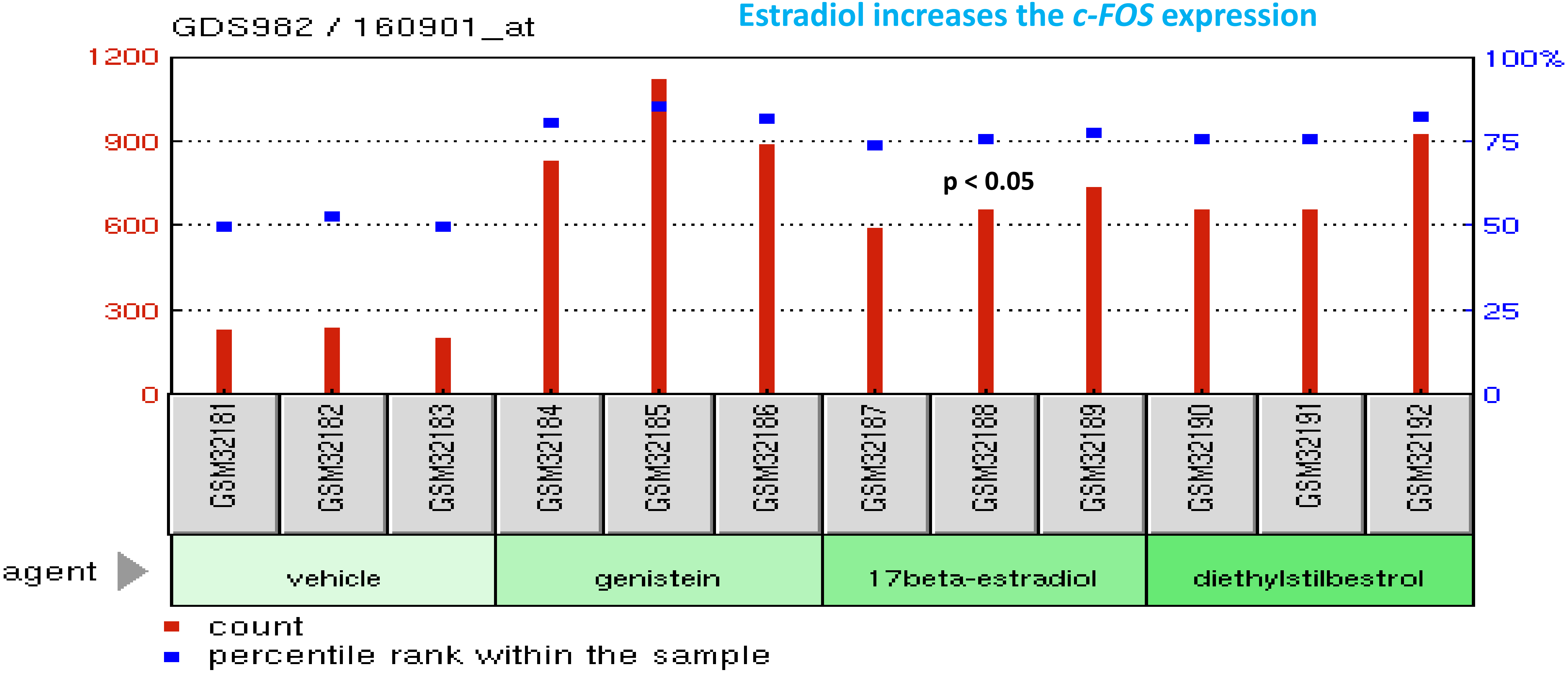

Profile: *c-FOS* expression
GDS982 / 160901_at
Title
Uterine response to physiologic, plant-derived, and synthetic estrogen
Organism
Mus musculus

Estradiol increases the *c-FOS* expression

Sample	Title	Value
GSM32181	AO C1	**236.8**
GSM32182	AO C2	**239.8**
GSM32183	AO C3	**208.6**
GSM32184	GEN G1	834.7
GSM32185	GEN G2	1122.6
GSM32186	GEN G3	892.9
GSM32187	E2 E1	599.6
GSM32188	E2 E2	660.6 **$p < 0.05$**
GSM32189	E2 E3	740.3
GSM32190	DES D1	659.8
GSM32191	DES D2	663.9
GSM32192	DES D3	928.8

Supplemental Figure S11. Testosterone effects on gene expression

An overview of the potential coronavirus-infection promoting activities

Profile: *ACE2* expression
GDS2920 / 222257_s_at
Title
Testosterone effect on skeletal muscles of HIV-infected males experiencing weight loss
Organism
Homo sapiens

Testosterone increases *ACE2* expression

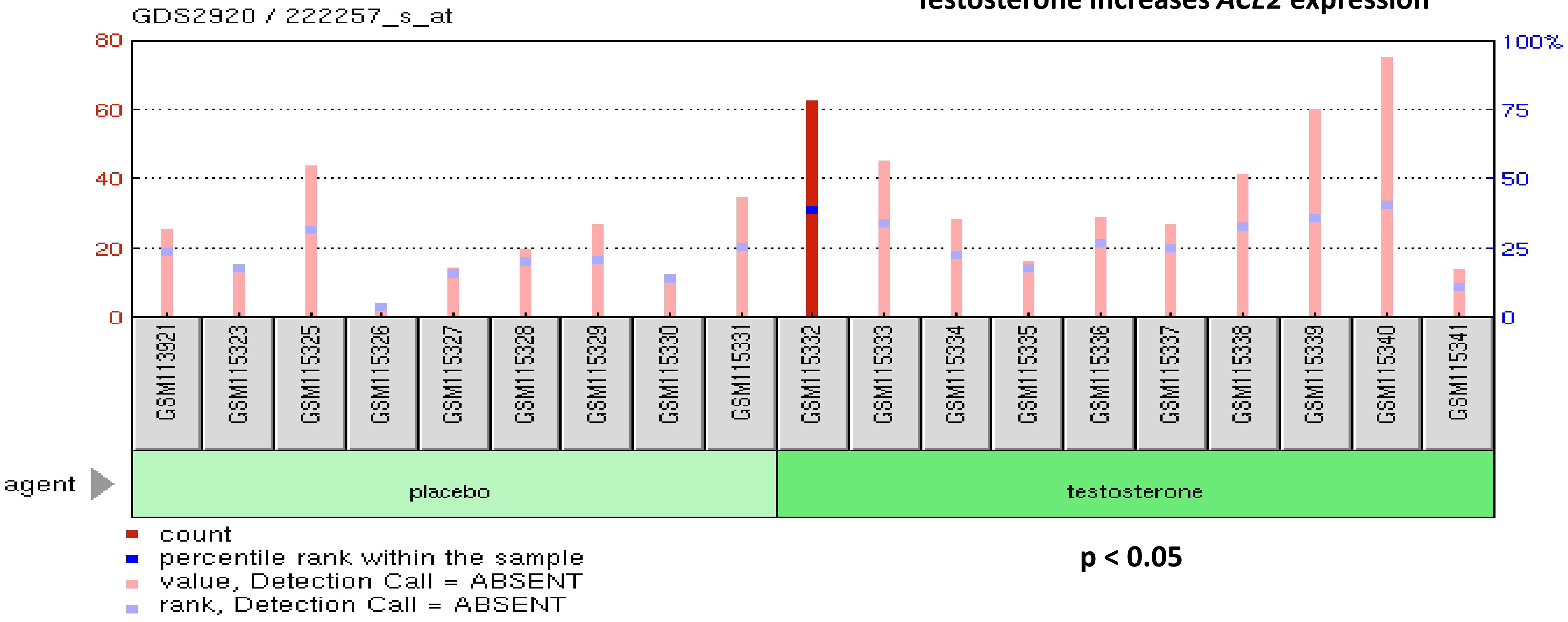

$p < 0.05$

Profile: *ACE2* expression
GDS2920 / 222257_s_at
Title
Testosterone effect on skeletal muscles of HIV-infected males experiencing weight loss
Organism
Homo sapiens

Testosterone increases *ACE2* expression

Sample	Title	Value	
GSM113921	Placebo treated, HIV-1 positive male, day 14 (1)	25.8	
GSM115323	Placebo treated, HIV-1 positive male, day 14 (2)	14.3	
GSM115325	Placebo treated, HIV-1 positive male, day 14 (3)	44.3	
GSM115326	Placebo treated, HIV-1 positive male, day 14 (4)	3.1	
GSM115327	Placebo treated, HIV-1 positive male, day 14 (5)	14.8	**$p < 0.05$**
GSM115328	Placebo treated, HIV-1 positive male, day 14 (6)	20.2	
GSM115329	Placebo treated, HIV-1 positive male, day 14 (7)	27	
GSM115330	Placebo treated, HIV-1 positive male, day 14 (8)	11.7	
GSM115331	Placebo treated, HIV-1 positive male, day 14 (9)	34.9	
GSM115332	Testosterone treated, HIV-1 positive male, day 14 (1)	**63.1**	
GSM115333	Testosterone treated, HIV-1 positive male, day 14 (2)	**45.6**	
GSM115334	Testosterone treated, HIV-1 positive male, day 14 (3)	**28.8**	
GSM115335	Testosterone treated, HIV-1 positive male, day 14 (4)	**16.8**	
GSM115336	Testosterone treated, HIV-1 positive male, day 14 (5)	**29.2**	
GSM115337	Testosterone treated, HIV-1 positive male, day 14 (6)	**27.3**	
GSM115338	Testosterone treated, HIV-1 positive male, day 14 (7)	**41.7**	
GSM115339	Testosterone treated, HIV-1 positive male, day 14 (8)	**60.3**	
GSM115340	Testosterone treated, HIV-1 positive male, day 14 (9)	**75.5**	
GSM115341	Testosterone treated, HIV-1 positive male, day 14 (10)	**14.1**	

Profile: *ACE2* expression
GDS1361 / 1598
Title
Testosterone effect on meibomian gland
Organism
Mus musculus

Testosterone increases *ACE2* expression

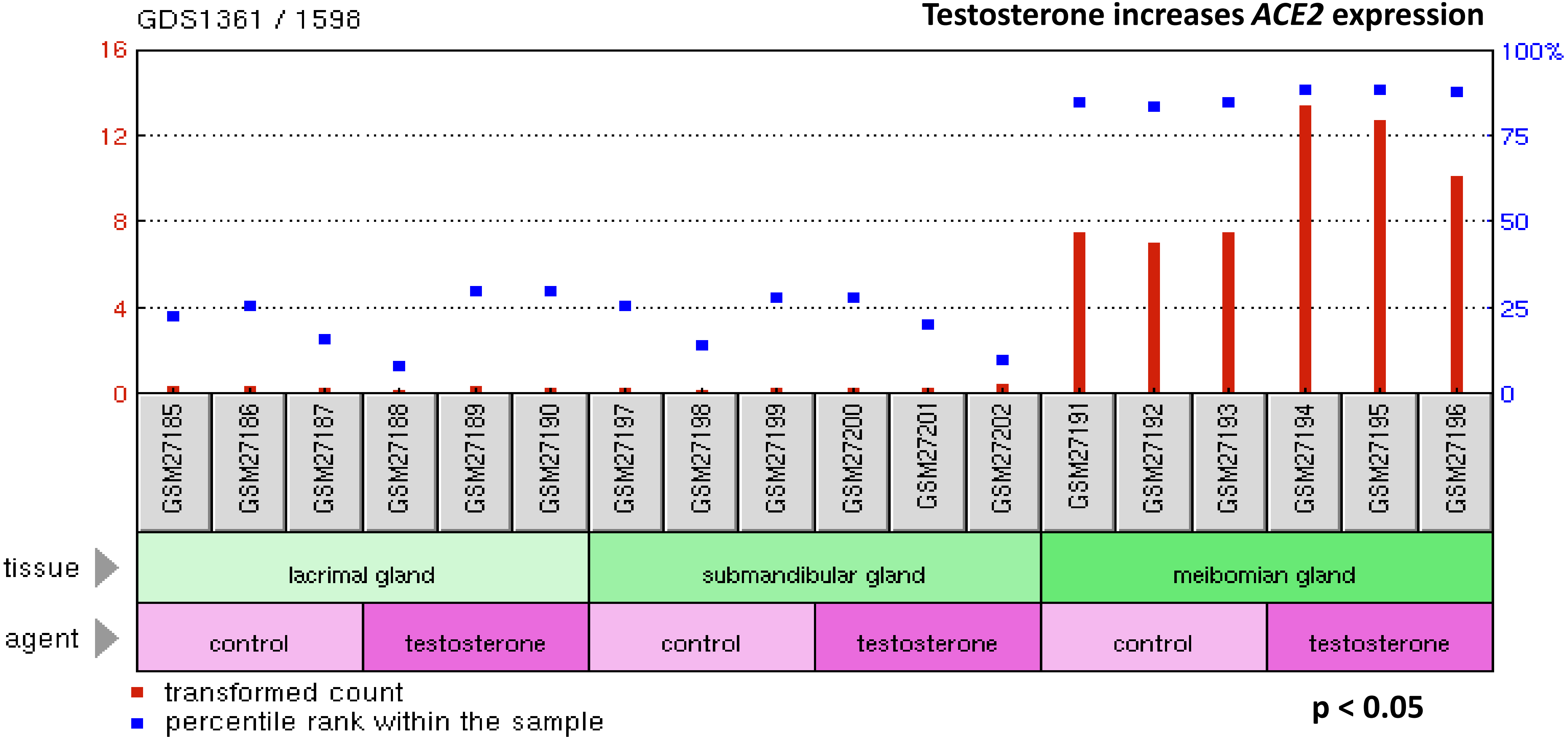

$p < 0.05$

Profile: *ACE2* expression
GDS1361 / 1598
Title
Testosterone effect on meibomian gland
Organism
Mus musculus

Testosterone increases *ACE2* expression

Sample	Title	Value		
GSM27185	Male Placebo-Treated Lacrimal Gland Sample A	0.432225		
GSM27186	Male Placebo-Treated Lacrimal Gland Sample B	0.457711		
GSM27187	Male Placeo-Treated Lacrimal Gland Sample C	0.359507		
GSM27188	Male Testosterone-Treated Lacrimal Gland Sample A	0.276599		
GSM27189	Male Testosterone-Treated Lacrimal Gland Sample B	0.425166		
GSM27190	Male Testosterone-Treated Lacrimal Gland Sample C	0.367615		
GSM27197	Male Placebo-Treated Submandibular Gland Sample A	0.343573		
GSM27198	Male Placebo-Treated Submandibular Gland Sample B	0.271271		
GSM27199	Male Placebo-Treated Submandibular Gland Sample C	0.384337		
GSM27200	Male Testosterone-Treated Submandibular Gland Sample A	0.365324		
GSM27201	Male Testosterone-Treated Submandibular Gland Sample B	0.308682		
GSM27202	Male Testosterone-Treated Submandibular Gland Sample C	0.205059		
GSM27191	Male Placebo-Treated Meibomian Gland Sample A	**7.61018**		
GSM27192	Male Placebo-Treated Meibomian Gland Sample B	**7.08807**	**$p < 0.05$**	
GSM27193	Male Placebo-Treated Meibomian Gland Sample C	**7.54525**		
GSM27194	Male Testosterone-Treated Meibomian Gland Sample A			**13.4706**
GSM27195	Male Testosterone-Treated Meibomian Gland Sample B			**12.7558**
GSM27196	Male Testosterone-Treated Meibomian Gland Sample C			**10.1925**

Profile: *ACE2* expression
GDS1832 / 1598
Title
Testosterone effect on female lacrimal, meibomian, and submandibular glands
Organism
Mus musculus

Testosterone increases *ACE2* expression

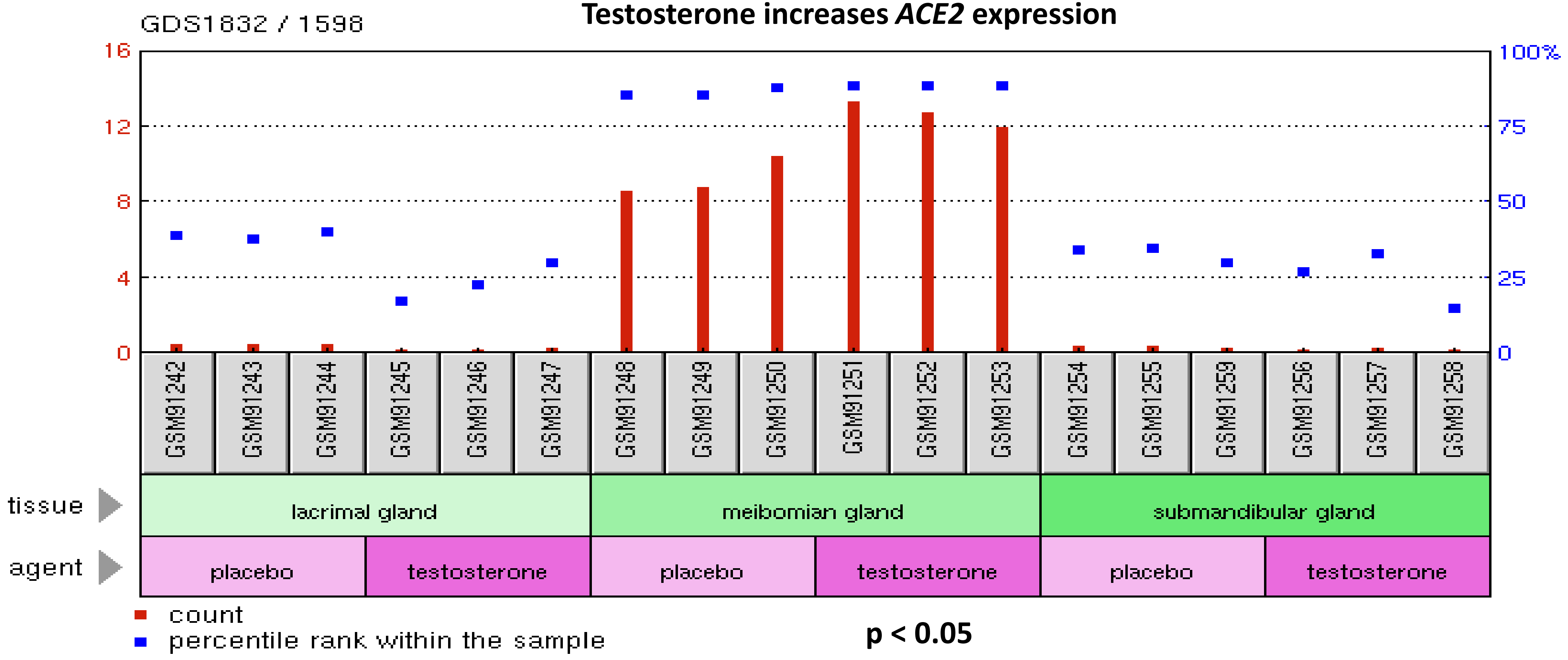

$p < 0.05$

Profile: *ACE2* expression

GDS1832 / 1598

Title

Testosterone effect on female lacrimal, meibomian, and submandibular glands

Organism

Mus musculus

Testosterone increases *ACE2* expression

Sample	Title	Value	
GSM91242	Female Placebo-Treated Lacrimal Gland Sample A	0.560279	
GSM91243	Female Placebo-Treated Lacrimal Gland Sample B	0.522894	**$p < 0.05$**
GSM91244	Female Placebo-Treated Lacrimal Gland Sample C	0.566438	
GSM91245	Female Testosterone-Treated Lacrimal Gland Sample A	**0.23071**	
GSM91246	Female Testosterone-Treated Lacrimal Gland Sample B	**0.275703**	
GSM91247	Female Testosterone-Treated Lacrimal Gland Sample C	**0.385156**	
GSM91248	Female Placebo-Treated Meibomian Gland Sample A	**8.58739**	
GSM91249	Female Placebo-Treated Meibomian Gland Sample B	**8.79653**	
GSM91250	Female Placebo-Treated Meibomian Gland Sample C	**10.4129**	
GSM91251	Female Testosterone-Treated Meibomian Gland Sample A	**13.3238**	
GSM91252	Female Testosterone-Treated Meibomian Gland Sample B	**12.7645**	**$p < 0.05$**
GSM91253	Female Testosterone-Treated Meibomian Gland Sample C	**11.962**	
GSM91254	Female Placebo-Treated Submandibular Gland Sample B	0.398068	
GSM91255	Female Placebo-Treated Submandibular Gland Sample C	0.403039	
GSM91259	Female Placebo-Treated Submandibular Gland Sample A	0.35605	
GSM91256	Female Testosterone-Treated Submandibular Gland Sample A	0.238247	
GSM91257	Female Testosterone-Treated Submandibular Gland Sample B	0.365064	$p = 0.130$
GSM91258	Female Testosterone-Treated Submandibular Gland Sample C	0.206147	

Profile: *FURIN* expression
GDS1833 / 100515_at
Title
Testosterone effect on the female submandibular gland
Organism
Mus musculus

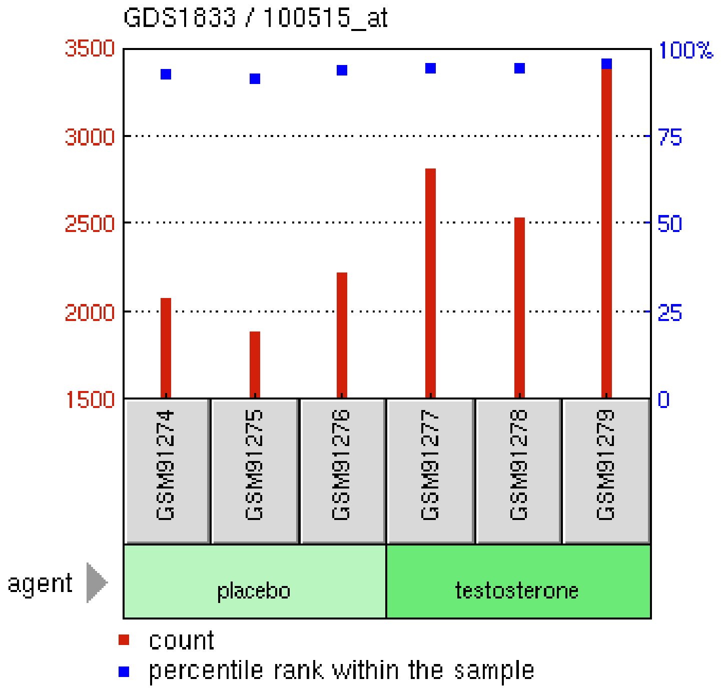

Testosterone increases *FURIN* expression

Sample	Title	Value	
GSM91274	Female Placebo-Treated Submandibular Gland Sample A Technical Replicate	2087.8	
GSM91275	Female Placebo-Treated Submandibular Gland Sample B Technical Replicate	1888.3	**$p < 0.05$**
GSM91276	Female Placebo-Treated Submandibular Gland Sample C Technical Replicate	2230.6	
GSM91277	Female Testosterone-Treated Submandibular Gland Sample A Technical Replicate	**2822.9**	
GSM91278	Female Testosterone-Treated Submandibular Gland Sample B Technical Replicate	**2536.3**	
GSM91279	Female Testosterone-Treated Submandibular Gland Sample C Technical Replicate	**3392.9**	

Profile: *FURIN* expression
GDS2562 / 100515_at
Title
Prostate response to castration and subsequent hormone replacement
Organism: Mus musculus

Castration decreases *FURIN* expression

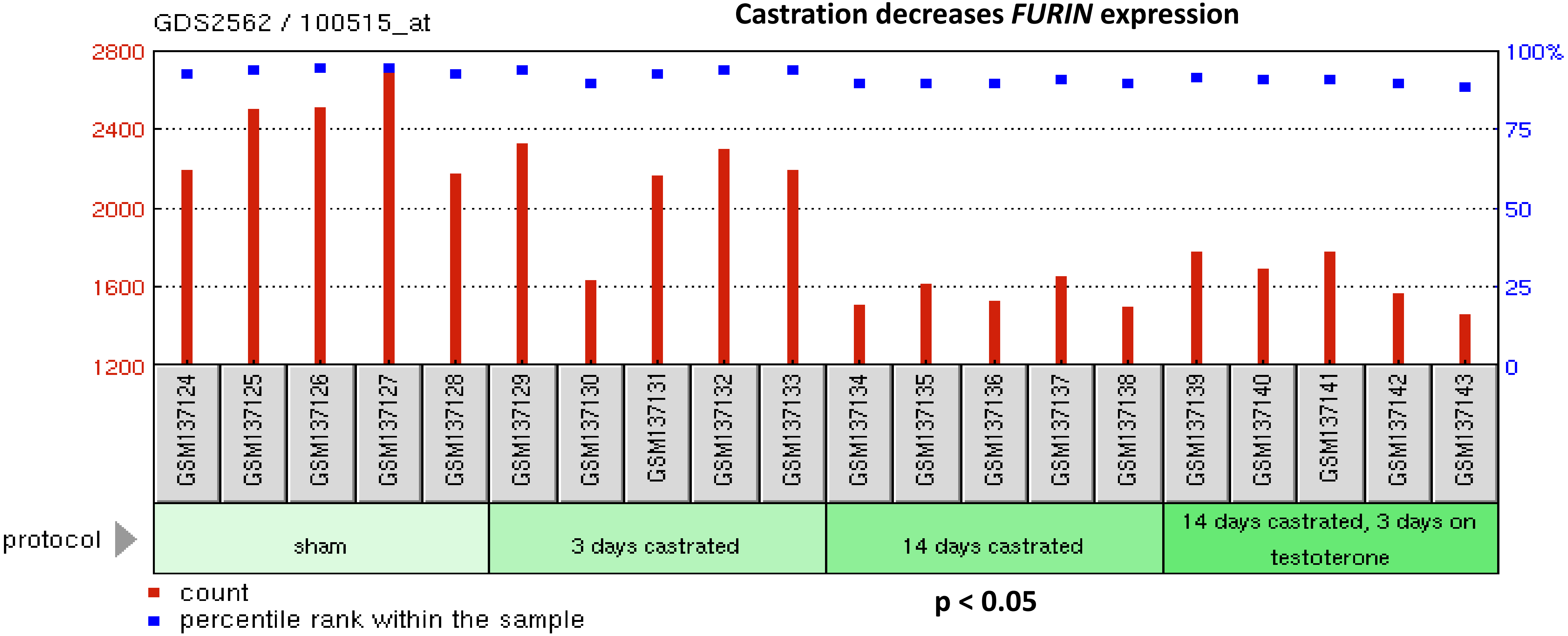

$p < 0.05$

Profile: *FURIN* expression
GDS2562 / 100515_at
Title
Prostate response to castration and subsequent hormone replacement
Organism: Mus musculus

Castration decreases *FURIN* expression

Sample	Title	Value
GSM137124	PRB3660 (MG-U74A)	2201.3
GSM137125	PRB3661 (MG-U74A)	2506.4
GSM137126	PRB3662 (MG-U74A)	2519.5
GSM137127	PRB3663 (MG-U74A)	2710.3
GSM137128	PRB3664 (MG-U74A)	2173.5
GSM137129	PRB3665 (MG-U74A)	**2332.6**
GSM137130	PRB3666 (MG-U74A)	**1636.1**
GSM137131	PRB3667 (MG-U74A)	**2167.8**
GSM137132	PRB3668 (MG-U74A)	**2305.2**
GSM137133	PRB3669 (MG-U74A)	**2193.6**
GSM137134	PRB3698 (MG-U74A)	**1510.4**
GSM137135	PRB3699 (MG-U74A)	**1619.7**
GSM137136	PRB3700 (MG-U74A)	**$p < 0.05$** **1532.1**
GSM137137	PRB3701 (MG-U74A)	**1654.5**
GSM137138	PRB3702 (MG-U74A)	**1502.6**
GSM137139	PRB3708 (MG-U74A)	1787.8
GSM137140	PRB3709 (MG-U74A)	1695.8
GSM137141	PRB3710 (MG-U74A)	1787.3
GSM137142	PRB3711 (MG-U74A)	1575.8
GSM137143	PRB3712 (MG-U74A)	1467.6

Profile: *FURIN* expression
GDS1361 / 5982
Title
Testosterone effect on meibomian gland
Organism
Mus musculus

Testosterone increases *FURIN* expression

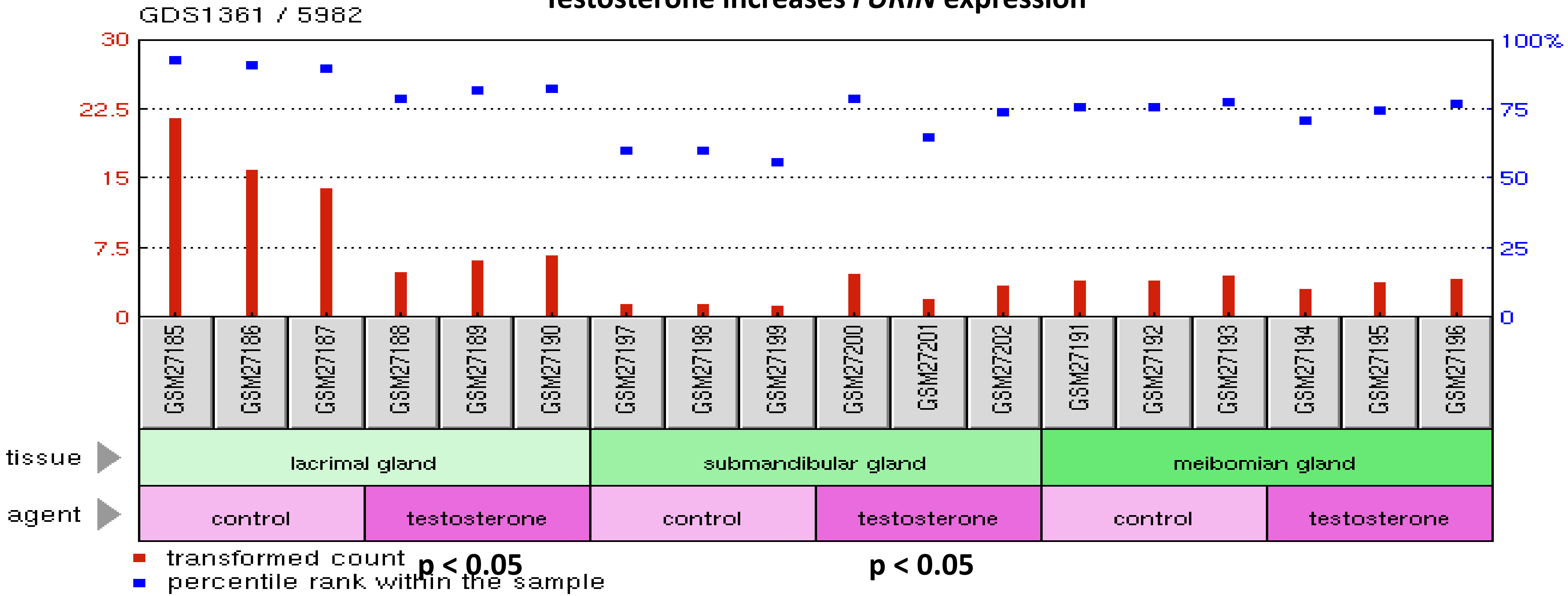

$p < 0.05$

$p < 0.05$

Profile: *FURIN* expression
GDS1361 / 5982
Title
Testosterone effect on meibomian gland
Organism
Mus musculus

Testosterone effects on *FURIN* expression

Sample	Title	Value	
GSM27185	Male Placebo-Treated Lacrimal Gland Sample A	21.6254	
GSM27186	Male Placebo-Treated Lacrimal Gland Sample B	15.916	**$p < 0.05$**
GSM27187	Male Placeo-Treated Lacrimal Gland Sample C	14.0492	
GSM27188	Male Testosterone-Treated Lacrimal Gland Sample A		**5.04977**
GSM27189	Male Testosterone-Treated Lacrimal Gland Sample B		**6.27436**
GSM27190	Male Testosterone-Treated Lacrimal Gland Sample C		**6.81647**
GSM27197	Male Placebo-Treated Submandibular Gland Sample A	**1.62319**	
GSM27198	Male Placebo-Treated Submandibular Gland Sample B	**1.54446**	**$p < 0.05$**
GSM27199	Male Placebo-Treated Submandibular Gland Sample C	**1.27973**	
GSM27200	Male Testosterone-Treated Submandibular Gland Sample A		**4.75181**
GSM27201	Male Testosterone-Treated Submandibular Gland Sample B		**2.12511**
GSM27202	Male Testosterone-Treated Submandibular Gland Sample C		**3.60242**
GSM27191	Male Placebo-Treated Meibomian Gland Sample A	4.08816	
GSM27192	Male Placebo-Treated Meibomian Gland Sample B	4.09734	
GSM27193	Male Placebo-Treated Meibomian Gland Sample C	4.58029	
GSM27194	Male Testosterone-Treated Meibomian Gland Sample A	3.08708	
GSM27195	Male Testosterone-Treated Meibomian Gland Sample B	3.88995	
GSM27196	Male Testosterone-Treated Meibomian Gland Sample C	4.22759	

Profile: *HMGA2* expression
GDS5301 / 1450780_PM_s_at
Title
Androgen deprivation effect on CD4 T-cells
Organism
Mus musculus

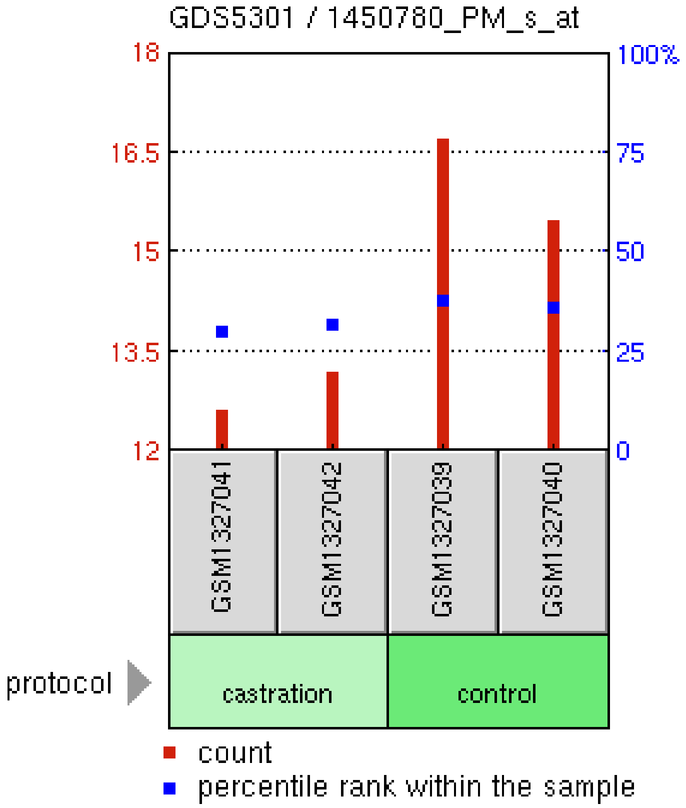

$p < 0.05$

Castration decreases *HMGA2* expression

Sample	Title	Value
GSM1327041	Cast_1	12.6312
GSM1327042	Cast_2	13.2155
GSM1327039	Sham_1	16.7133
GSM1327040	Sham_2	15.4878

Profile: *INSIG1* expression
GDS5301 / 1454671_PM_at
Title
Androgen deprivation effect on CD4 T-cells
Organism
Mus musculus

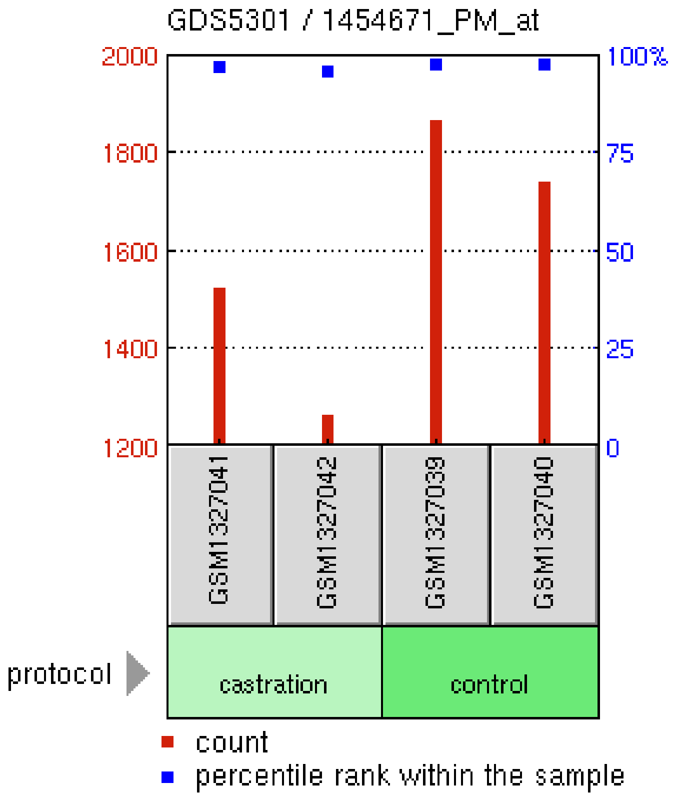

p = 0.053

Castration decreases *INSIG1* expression

Sample	Title	Value
GSM1327041	Cast_1	**1525.05**
GSM1327042	Cast_2	**1265.23**
GSM1327039	Sham_1	1866.04
GSM1327040	Sham_2	1741.1

Profile: *GATA5* expression
GDS1361 / 3834
Title
Testosterone effect on meibomian gland
Organism
Mus musculus

Testosterone effects on *GATA5* expression

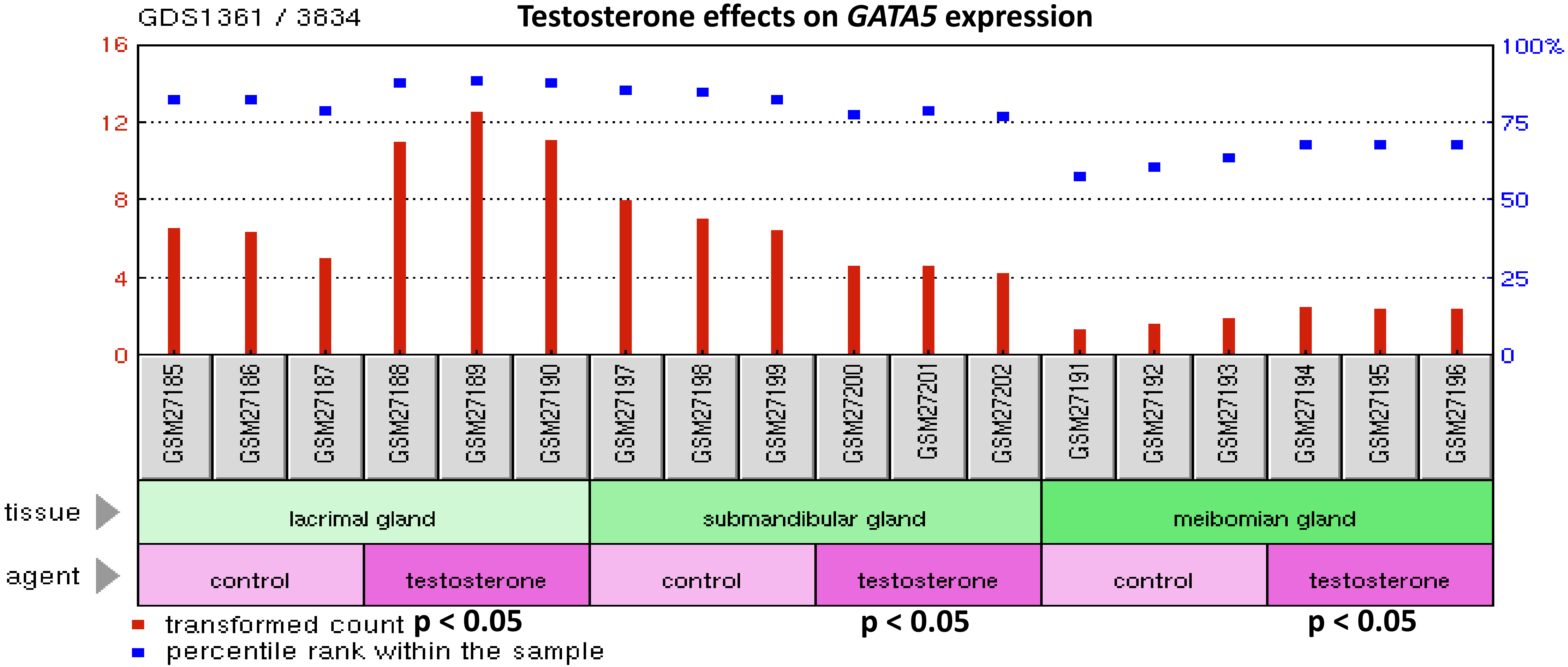

p < 0.05 **p < 0.05** **p < 0.05**

Profile: *GATA5* expression

GDS1361 / 3834

Title

Testosterone effect on meibomian gland

Organism

Mus musculus

Testosterone effects on *GATA5* expression

Sample	Title	Value	
GSM27185	Male Placebo-Treated Lacrimal Gland Sample A	6.64621	
GSM27186	Male Placebo-Treated Lacrimal Gland Sample B	6.45423	**$p < 0.05$**
GSM27187	Male Placeo-Treated Lacrimal Gland Sample C	5.10327	
GSM27188	Male Testosterone-Treated Lacrimal Gland Sample A	**10.9893**	
GSM27189	Male Testosterone-Treated Lacrimal Gland Sample B	**12.5373**	
GSM27190	Male Testosterone-Treated Lacrimal Gland Sample C	**11.1312**	
GSM27197	Male Placebo-Treated Submandibular Gland Sample A	**8.00934**	
GSM27198	Male Placebo-Treated Submandibular Gland Sample B	**7.04778**	**$p < 0.05$**
GSM27199	Male Placebo-Treated Submandibular Gland Sample C	**6.52222**	
GSM27200	Male Testosterone-Treated Submandibular Gland Sample A	**4.71727**	
GSM27201	Male Testosterone-Treated Submandibular Gland Sample B	**4.67808**	
GSM27202	Male Testosterone-Treated Submandibular Gland Sample C	**4.25649**	
GSM27191	Male Placebo-Treated Meibomian Gland Sample A	1.42448	
GSM27192	Male Placebo-Treated Meibomian Gland Sample B	1.65382	**$p < 0.05$**
GSM27193	Male Placebo-Treated Meibomian Gland Sample C	1.92874	
GSM27194	Male Testosterone-Treated Meibomian Gland Sample A	**2.54996**	
GSM27195	Male Testosterone-Treated Meibomian Gland Sample B	**2.49176**	
GSM27196	Male Testosterone-Treated Meibomian Gland Sample C	**2.45951**	

ACE2 and FURIN

Supplemental Figure S12. Potential mechanisms affecting gene expression inferred from

transgenic mouse models and observed in pathophysiologically & therapeutically relevant mouse and human cells:

A knowledge path toward potential therapy-enhancing interventions

Profile: ACE2 expression
GDS4584 / 1425103_at
Title
Alveolar type I cells deficient in sterol-response element-binding proteins inhibitor Insig1/2
Organism
Mus musculus

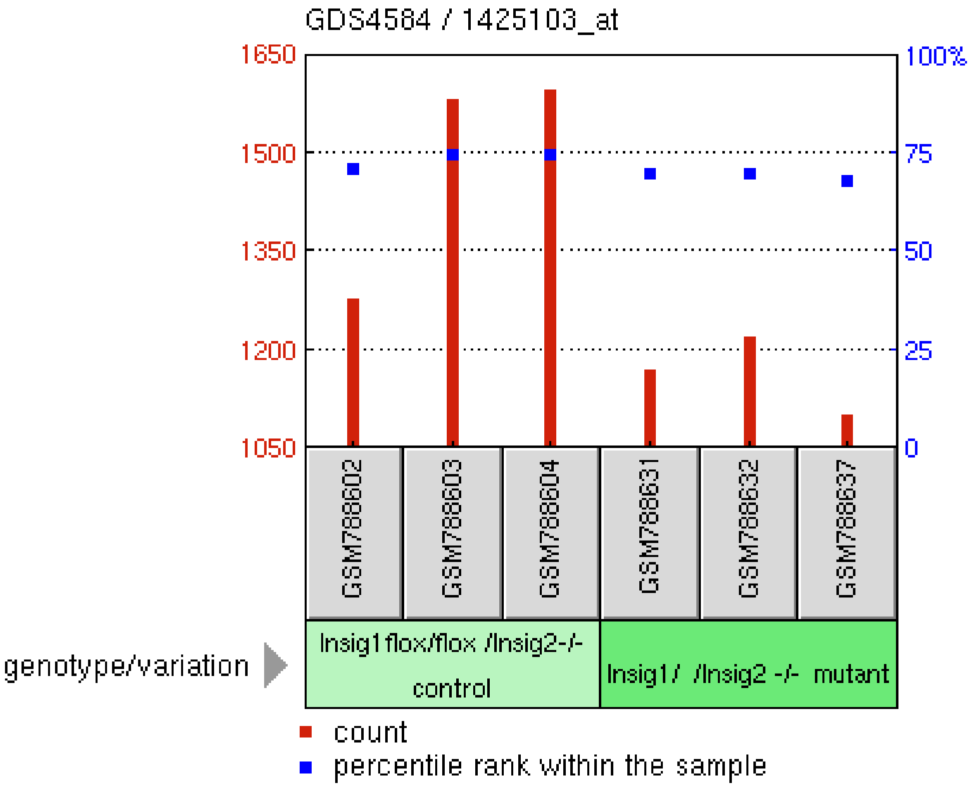

$p < 0.05$

Insig1: a candidate activator of the *ACE2* expression

Sample	Title	Value
GSM788602	Insig1 flox/flox /Insig2 -/- InsigKO_C1	1279.8
GSM788603	Insig1 flox/flox /Insig2 -/- InsigKO_C2	1584.9
GSM788604	Insig1 flox/flox /Insig2 -/- InsigKO_C3	1596
GSM788631	Insig1 Δ/Δ /Insig2 -/- InsigKO_E1	1169.8
GSM788632	Insig1 Δ/Δ /Insig2 -/- InsigKO_E2	1223
GSM788637	Insig1 Δ/Δ /Insig2 -/- InsigKO_E3	1102.8

Profile: ACE2 expression
GDS4584 / 1452138_a_at
Title
Alveolar type I cells deficient in sterol-response element-binding proteins inhibitor Insig1/2
Organism
Mus musculus

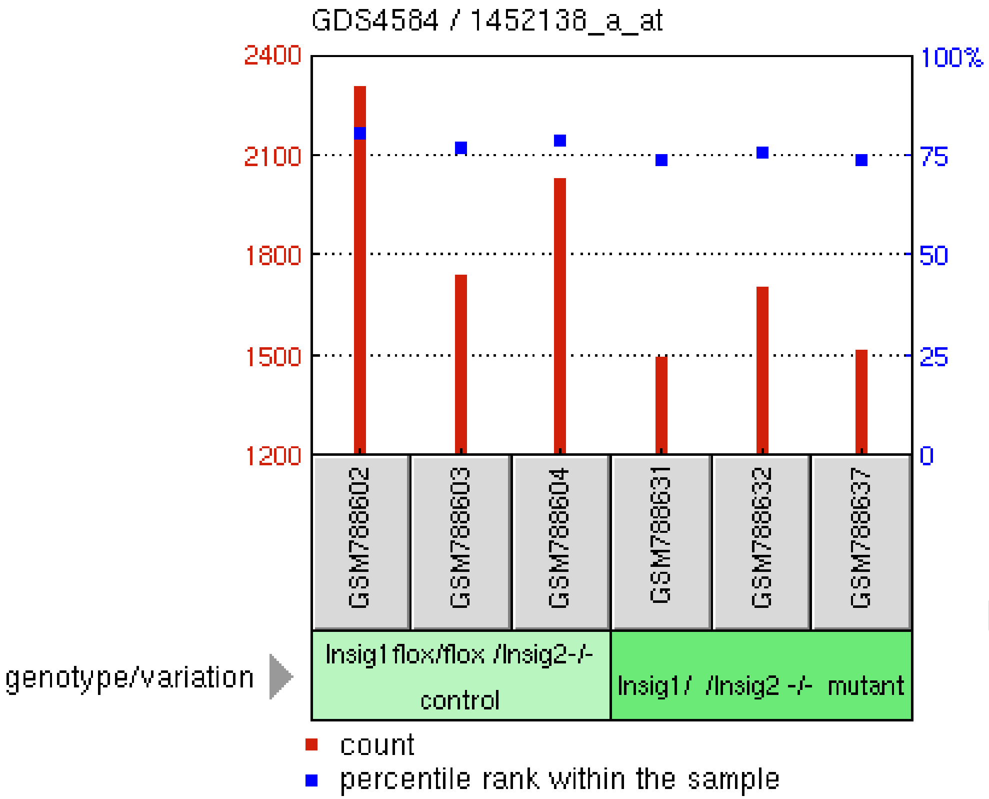

$p < 0.05$

Insig1: a candidate activator of the *ACE2* expression

Sample	Title	Value
GSM788602	Insig1 flox/flox /Insig2 -/- InsigKO_C1	2309.1
GSM788603	Insig1 flox/flox /Insig2 -/- InsigKO_C2	1742.5
GSM788604	Insig1 flox/flox /Insig2 -/- InsigKO_C3	2032.3
GSM788631	Insig1 Δ/Δ /Insig2 -/- InsigKO_E1	1496.5
GSM788632	Insig1 Δ/Δ /Insig2 -/- InsigKO_E2	1706.9
GSM788637	Insig1 Δ/Δ /Insig2 -/- InsigKO_E3	1518.6

Profile: *INSIG1* expression
GDS3676 / 201625_s_at
Title
Quercetin effect on CD14+ monocyte
Organism
Homo sapiens

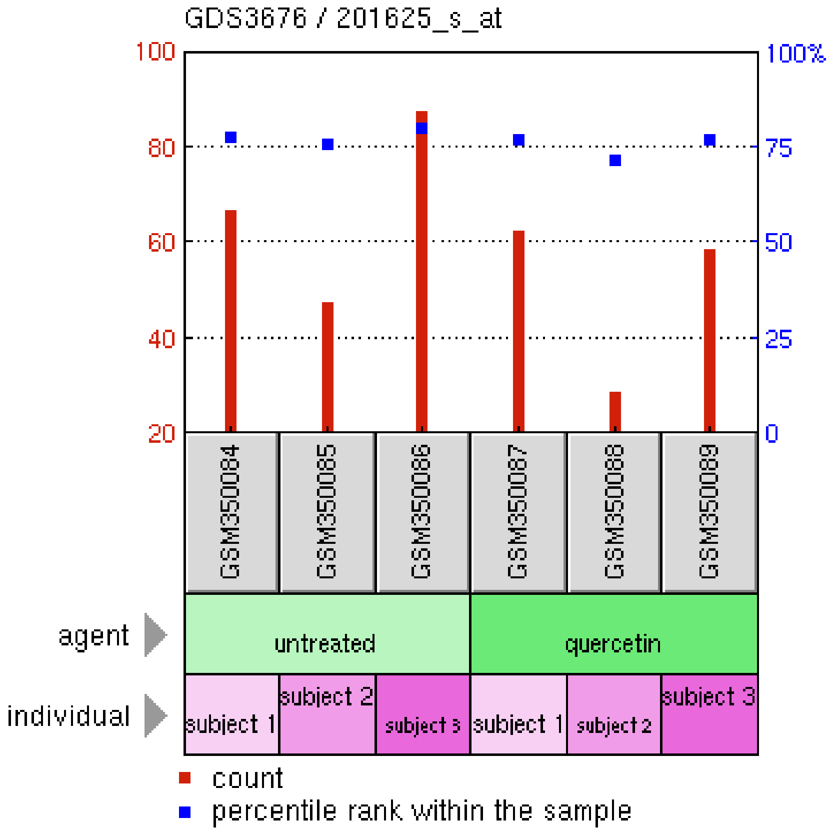

Quercetin inhibits the *INSIG1* expression

Sample	Title	Value	Percent changes
GSM350084	CD14+ at baseline, biological replicate 1	**66.9724**	
GSM350085	CD14+ at baseline, biological replicate 2	**47.9219**	
GSM350086	CD14+ at baseline, biological replicate 3	**87.5973**	
GSM350087	CD14+ after 2 wk quercetin supplementation, biological replicate 1	**62.5927**	**- 7%**
GSM350088	CD14+ after 2 wk quercetin supplementation, biological replicate 2	**28.8386**	**- 40%**
GSM350089	CD14+ after 2 wk quercetin supplementation, biological replicate 3	**58.8768**	**- 33%**

Profile: *HIF1a* expression
GDS4584 / 1427418_a_at
Title
Alveolar type I cells deficient in sterol-response element-binding proteins inhibitor Insig1/2
Organism
Mus musculus

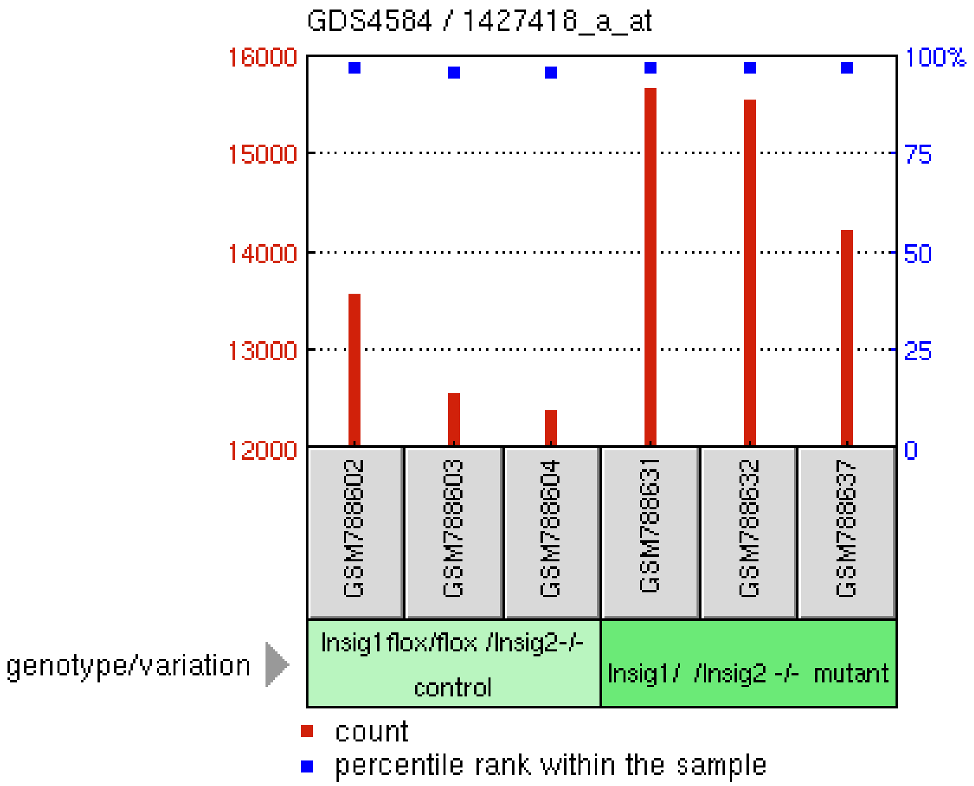

$p < 0.05$

Insig1: a candidate repressor of the *HIF1a* expression

Sample	Title	Value
GSM788602	Insig1 flox/flox /Insig2 -/- InsigKO_C1	13581.9
GSM788603	Insig1 flox/flox /Insig2 -/- InsigKO_C2	12568
GSM788604	Insig1 flox/flox /Insig2 -/- InsigKO_C3	12390.1
GSM788631	Insig1 Δ/Δ /Insig2 -/- InsigKO_E1	**15685.4**
GSM788632	Insig1 Δ/Δ /Insig2 -/- InsigKO_E2	**15544.2**
GSM788637	Insig1 Δ/Δ /Insig2 -/- InsigKO_E3	**14222.5**

Profile: *HIF1a* expression
GDS3676 / 200989_at
Title
Quercetin effect on CD14+ monocyte
Organism
Homo sapiens

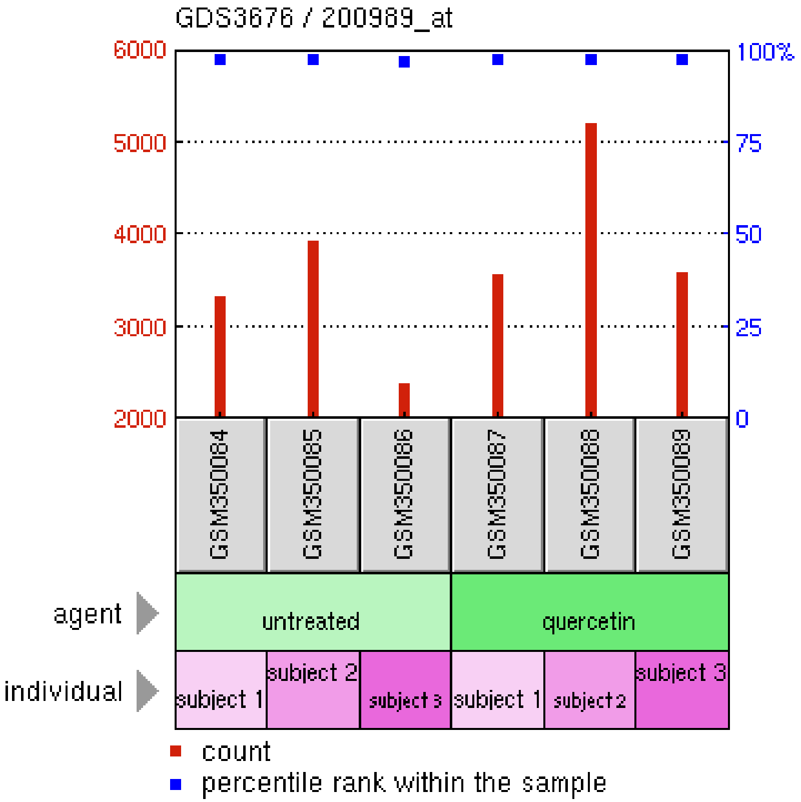

Quercetin activates the *HIF1a* expression

Sample	Title	Value
GSM350084	CD14+ at baseline, biological replicate 1	**3338.47**
GSM350085	CD14+ at baseline, biological replicate 2	**3929.88**
GSM350086	CD14+ at baseline, biological replicate 3	**2388.45**
GSM350087	CD14+ after 2 wk quercetin supplementation, biological replicate 1	**3577.94 (+ 7.2%)**
GSM350088	CD14+ after 2 wk quercetin supplementation, biological replicate 2	**5206.25 (+32.5%)**
GSM350089	CD14+ after 2 wk quercetin supplementation, biological replicate 3	**3604.21 (+50.9%)**

Profile: *HIF1A* expression

GDS3032 / 200989_at

Title

Quercetin effect on intestinal cell differentiation in vitro: time course

Organism

Homo sapiens

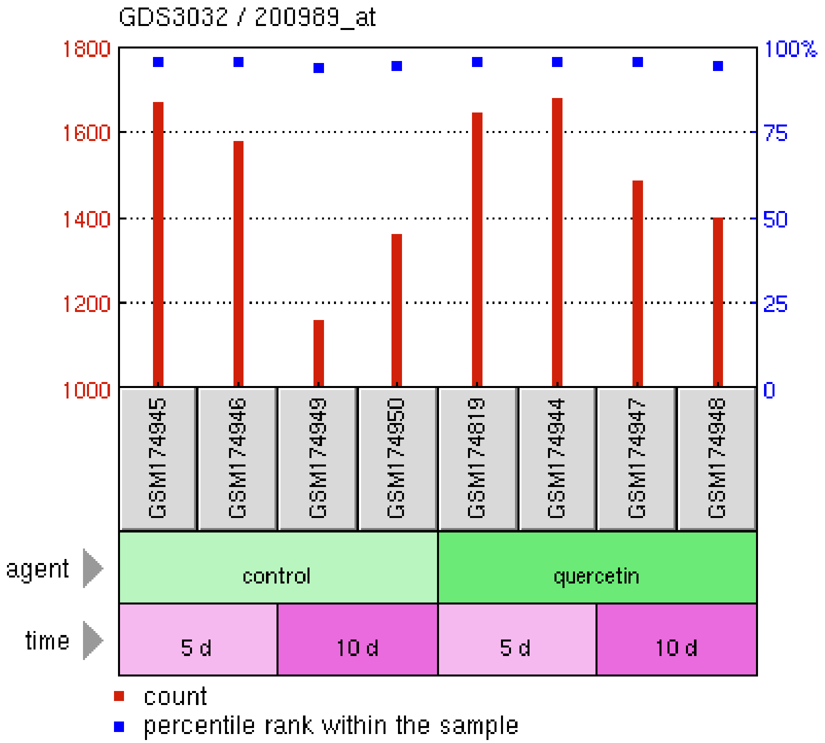

Quercetin ameliorates decline of the *HIF1a* expression

Sample	Title	Value	
GSM174945	Control day 05 sample 1	1673.78	**1628**
GSM174946	Control day 05 sample 2	1582.51	
GSM174949	Control day 10 sample 1	**1163.29**	**1264 (-22.4%)**
GSM174950	Control day 10 sample 2	**1364.98**	
GSM174819	Quercetin day 05 sample 1	1646.59	**1663**
GSM174944	Quercetin day 05 sample 2	1680.64	
GSM174947	Quercetin day 10 sample 1	**1491.23**	**1447 (-13.0%)**
GSM174948	Quercetin day 10 sample 2	**1403.56**	

Profile: ACE2

GDS4876 / 1425103_at

Title

Pulmonary surfactant protein-C deficiency effect on alveolar type II epithelial cell

Organism

Mus musculus

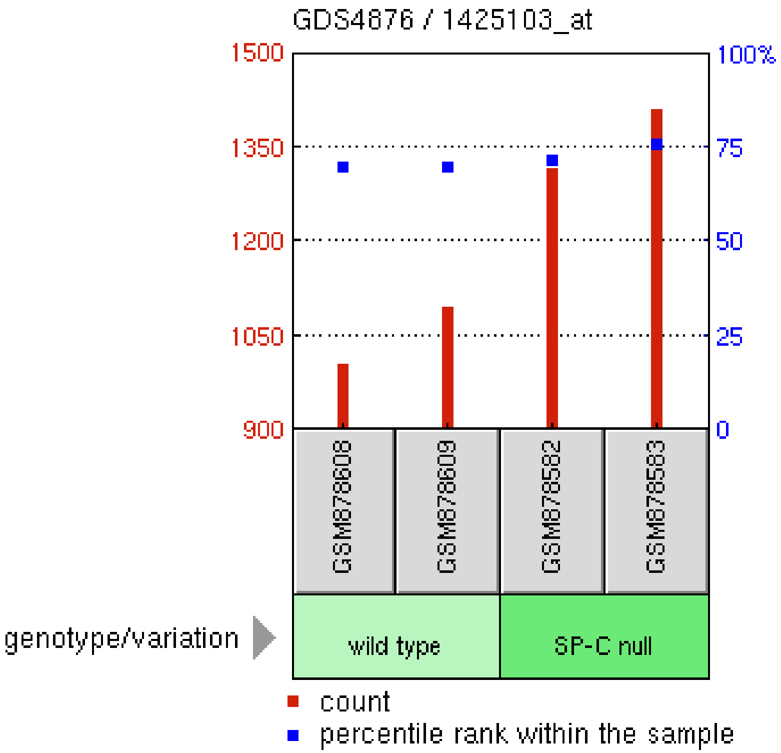

$p < 0.05$

SP-C (SFTPC): a candidate inhibitor of the *ACE2* expression

Sample	Title	Value
GSM878608	Wild-type Type II 1_8_9_04	1006
GSM878609	Wild-type Type II 9_27_04	1097.9
GSM878582	KO SPC Type II 9_27_04	1317.1
GSM878583	KO SPC Type II 2_8_9_04	1413.2

Profile: FURIN expression
GDS4876 / 1418518_at
Title
Pulmonary surfactant protein-C deficiency effect on alveolar type II epithelial cell
Organism
Mus musculus

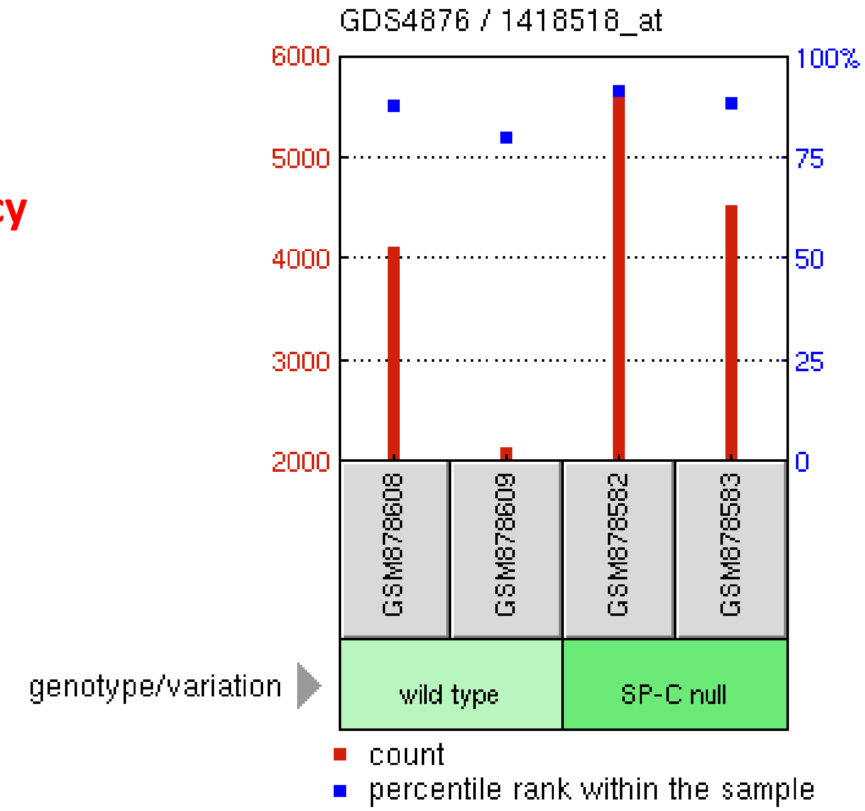

p = 0.114

SP-C (SFTPC): a candidate inhibitor of the *FURIN* expression

Sample	Title	Value
GSM878608	**Wild-type Type II 1_8_9_04**	**4141.2**
GSM878609	**Wild-type Type II 9_27_04**	**2156.1**
GSM878582	**KO SPC Type II 9_27_04**	**5733**
GSM878583	**KO SPC Type II 2_8_9_04**	**4537.4**

Profile: SP-C (SFTPC) expression
GDS3032 / 214387_x_at
Title
Quercetin effect on intestinal cell differentiation in vitro: time course
Organism
Homo sapiens

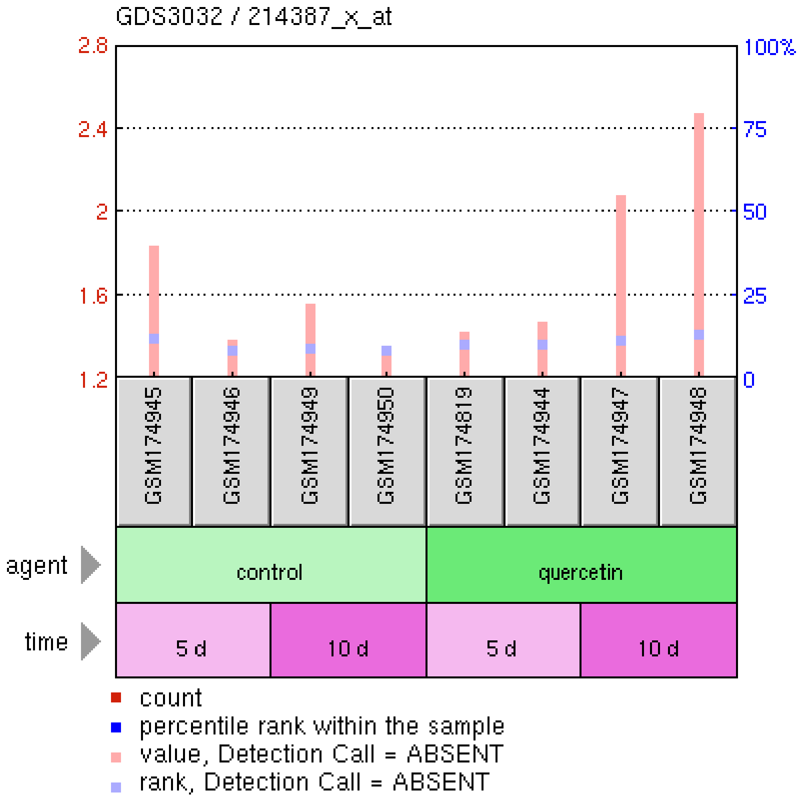

10 days
$p < 0.05$

Quercetin enhances the *SP-1 (SFTPC)* expression

Sample	Title	Value
GSM174945	Control day 05 sample 1	1.84127
GSM174946	Control day 05 sample 2	1.38638
GSM174949	Control day 10 sample 1	**1.56441**
GSM174950	Control day 10 sample 2	**1.35256**
GSM174819	Quercetin day 05 sample 1	1.42897
GSM174944	Quercetin day 05 sample 2	1.47099
GSM174947	Quercetin day 10 sample 1	**2.08499**
GSM174948	Quercetin day 10 sample 2	**2.47973**

Profile: SP-C (SFTPC) expression
GDS3676 / 38691_s_at
Title
Quercetin effect on CD14+ monocyte
Organism
Homo sapiens

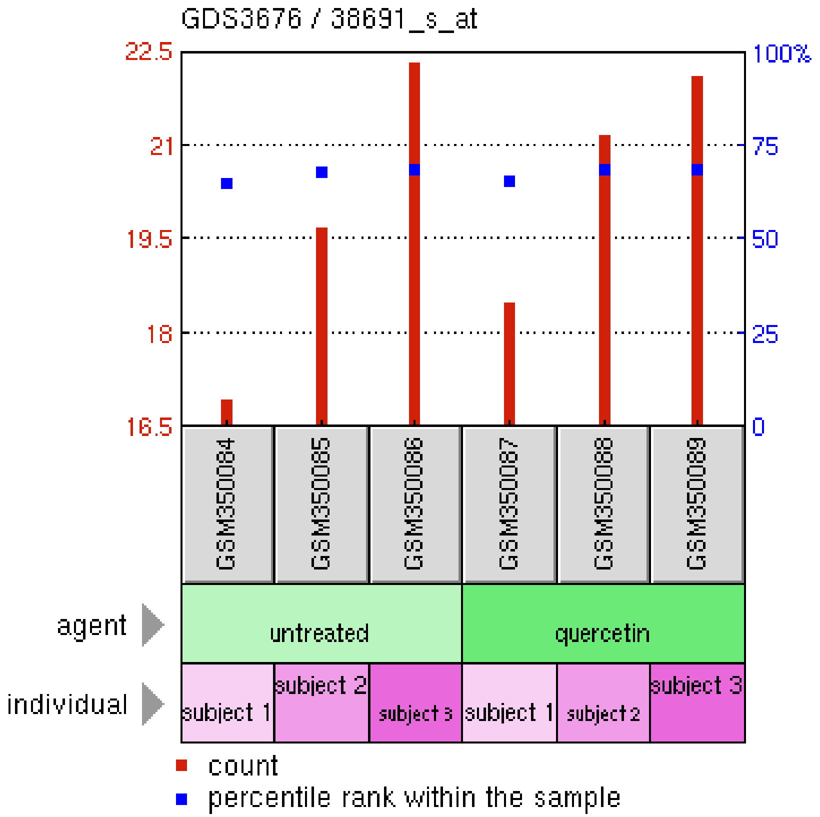

Quercetin enhances the *SP-1 (SFTPC)* expression

Sample	Title	Value
GSM350084	CD14+ at baseline, biological replicate 1	**16.9619**
GSM350085	CD14+ at baseline, biological replicate 2	**19.6833**
GSM350086	CD14+ at baseline, biological replicate 3	**22.3292**
GSM350087	CD14+ after 2 wk quercetin supplementation, biological replicate 1	**18.5089**
GSM350088	CD14+ after 2 wk quercetin supplementation, biological replicate 2	**21.1853**
GSM350089	CD14+ after 2 wk quercetin supplementation, biological replicate 3	**22.11**

Profile: ACE2 expression
GDS5048 / 10603066
Title
High Mobility Group AT-hook Protein 2 deficiency effect on the embryonic lung
Organism
Mus musculus

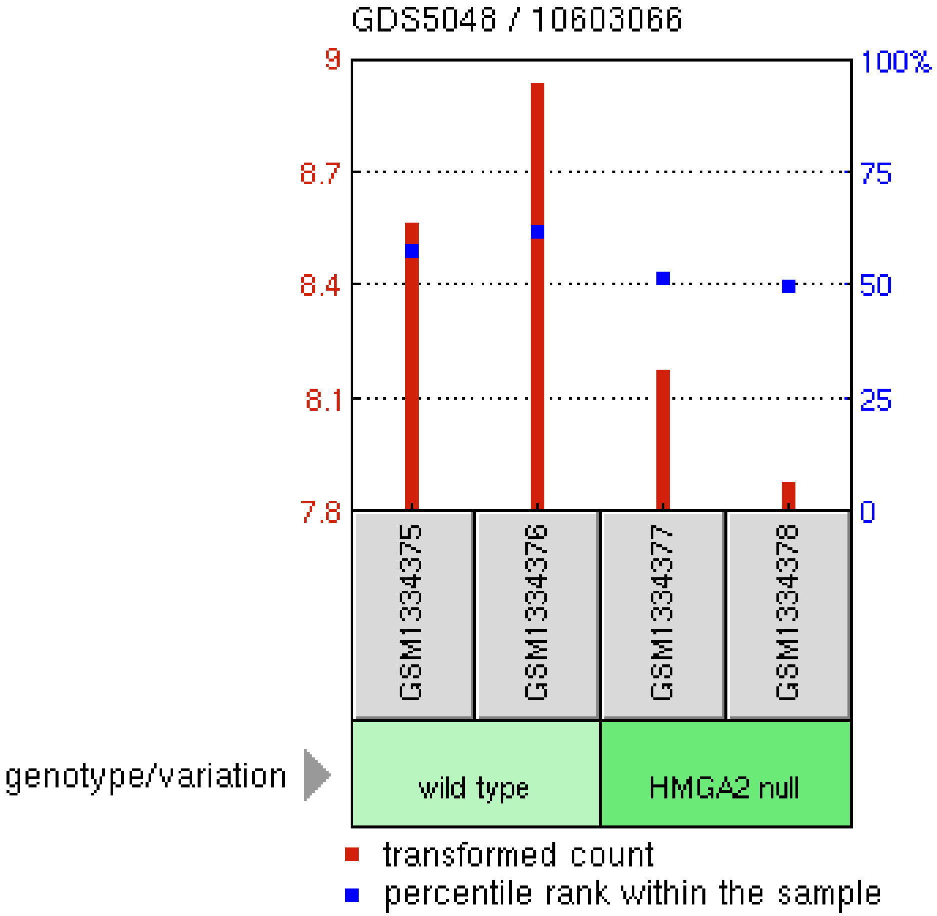

$p < 0.05$

***HMGA2*: a candidate activator of the *ACE2* expression**

Sample	Title	Value
GSM1334375	E18.5 lung WT1, biological rep1	8.57131
GSM1334376	E18.5 lung WT1, biological rep2	8.93831
GSM1334377	E18.5 lung KO1, biological rep1	8.18208
GSM1334378	E18.5 lung KO2, biological rep2	7.87966

Profile: *INSIG1* expression
GDS5048 / 10520362
Title
High Mobility Group AT-hook Protein 2 deficiency effect on the embryonic lung
Organism
Mus musculus

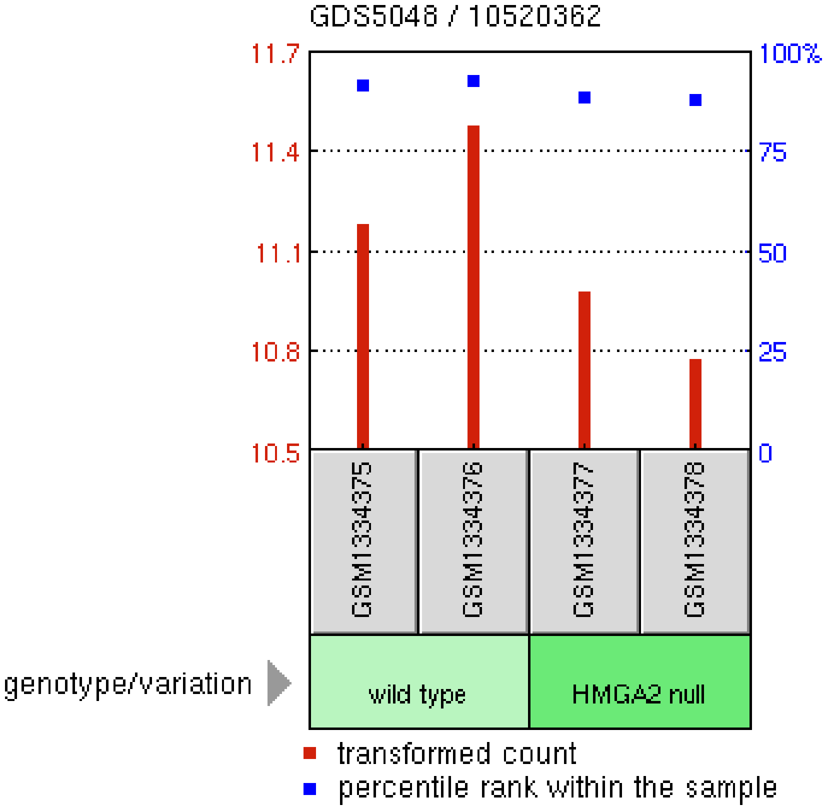

p = 0.062

HMGA2: a candidate activator of the *INSIG1* expression

Sample	Title	Value
GSM1334375	E18.5 lung WT1, biological rep1	11.1863
GSM1334376	E18.5 lung WT1, biological rep2	11.4769
GSM1334377	E18.5 lung KO1, biological rep1	10.9789
GSM1334378	E18.5 lung KO2, biological rep2	10.7801

Profile: *SFTPC* expression
GDS5048 / 10421387
Title
High Mobility Group AT-hook Protein 2 deficiency effect on the embryonic lung
Organism
Mus musculus

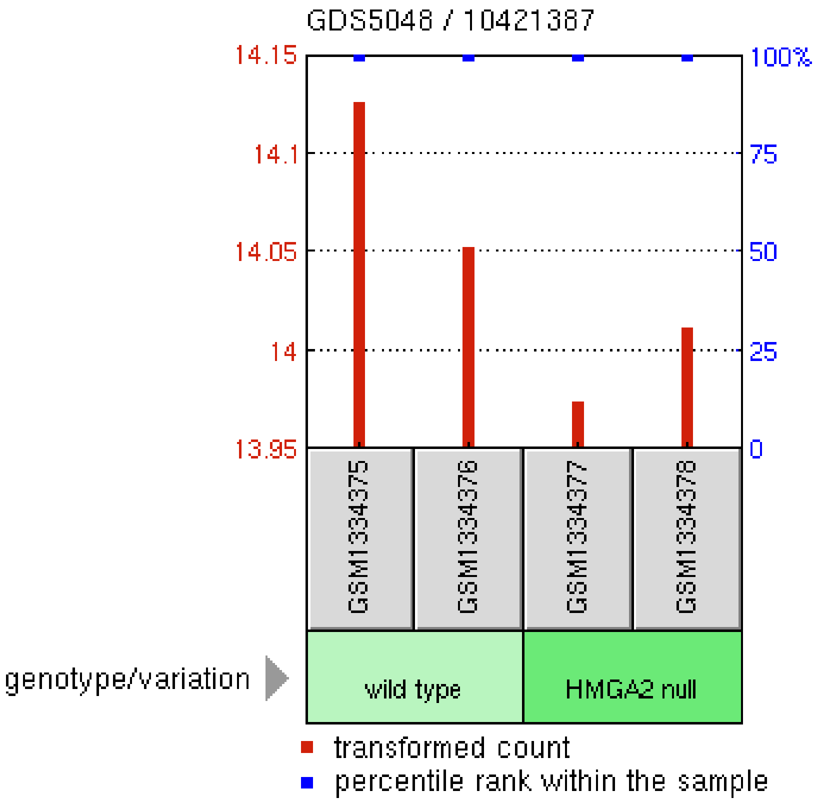

p = 0.073

HMGA2: a candidate activator of the *SFTPC* expression

Sample	Title	Value
GSM1334375	E18.5 lung WT1, biological rep1	14.1269
GSM1334376	E18.5 lung WT1, biological rep2	14.0528
GSM1334377	E18.5 lung KO1, biological rep1	13.975
GSM1334378	E18.5 lung KO2, biological rep2	14.0121

Profile: HMGA2 expression
GDS3676 / 1561633_at
Title
Quercetin effect on CD14+ monocyte
Organism
Homo sapiens

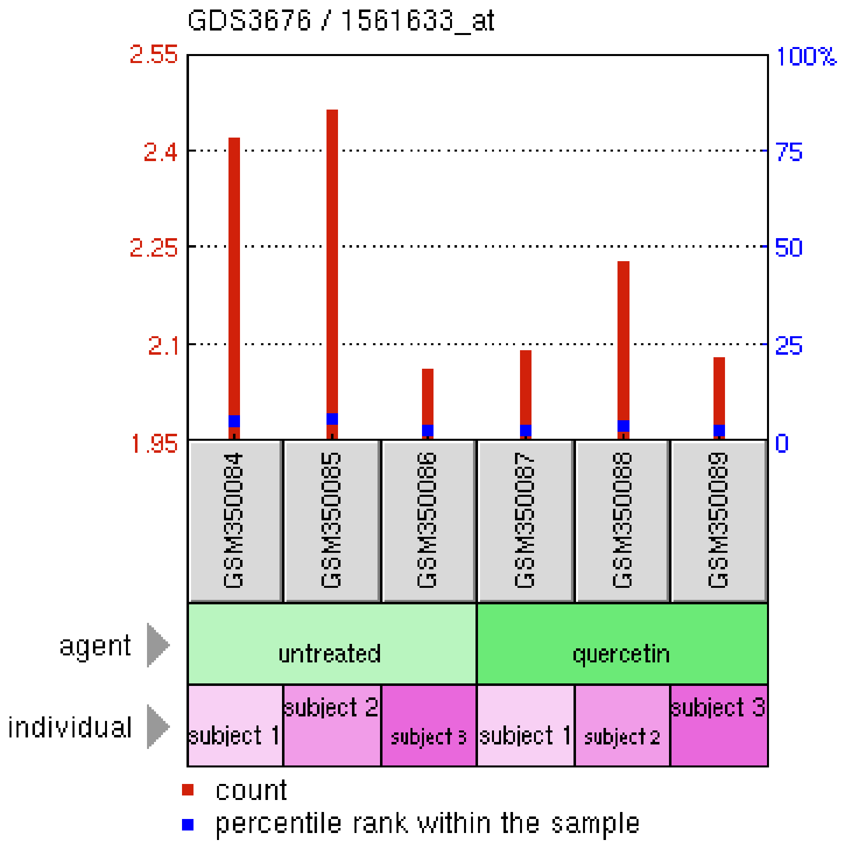

Quercetin inhibits the *HMGA2* expression

Sample	Title	Value
GSM350084	CD14+ at baseline, biological replicate 1	**2.4219**
GSM350085	CD14+ at baseline, biological replicate 2	**2.46504**
GSM350086	CD14+ at baseline, biological replicate 3	**2.06307**
GSM350087	CD14+ after 2 wk quercetin supplementation, biological replicate 1	**2.09436**
GSM350088	CD14+ after 2 wk quercetin supplementation, biological replicate 2	**2.22984**
GSM350089	CD14+ after 2 wk quercetin supplementation, biological replicate 3	**2.0814**

Profile: HMGA2 expression
GDS3032 / 208025_s_at
Title
Quercetin effect on intestinal cell differentiation in vitro: time course
Organism
Homo sapiens

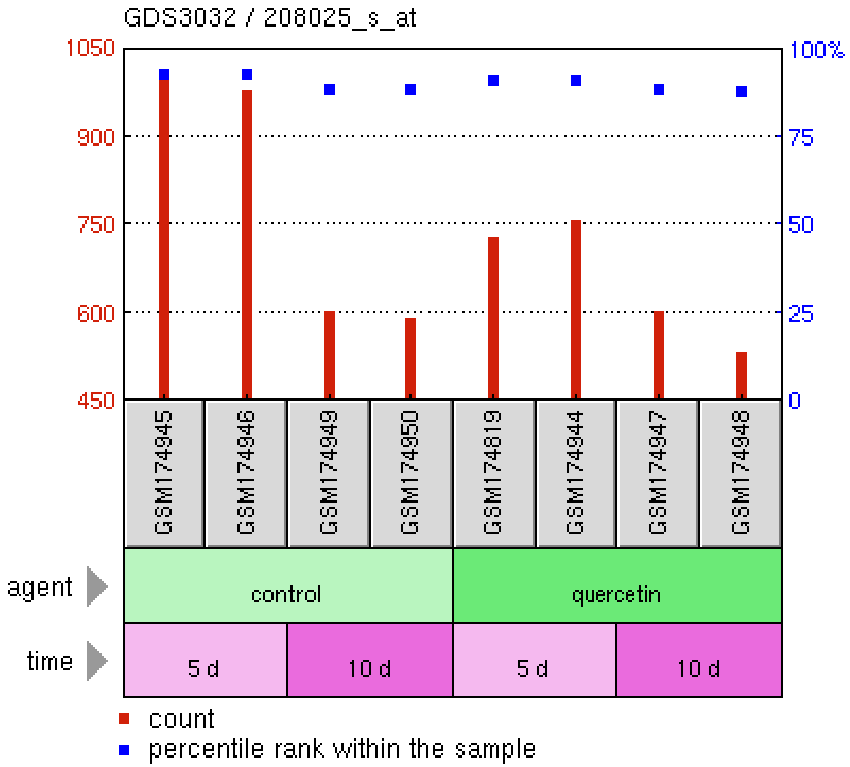

5 days
$p < 0.05$

Quercetin inhibits the *HMGA2* expression

Sample	Title	Value
GSM174945	Control day 05 sample 1	1015.28
GSM174946	Control day 05 sample 2	977.764
GSM174949	Control day 10 sample 1	601.884
GSM174950	Control day 10 sample 2	591.613
GSM174819	Quercetin day 05 sample 1	729.032
GSM174944	Quercetin day 05 sample 2	758.127
GSM174947	Quercetin day 10 sample 1	602.964
GSM174948	Quercetin day 10 sample 2	535.877

Profile: HMGA2 expression
GDS2708 / 14220
Title
Quercetin effect on cultured cardiomyocytes: time course
Organism
Rattus norvegicus

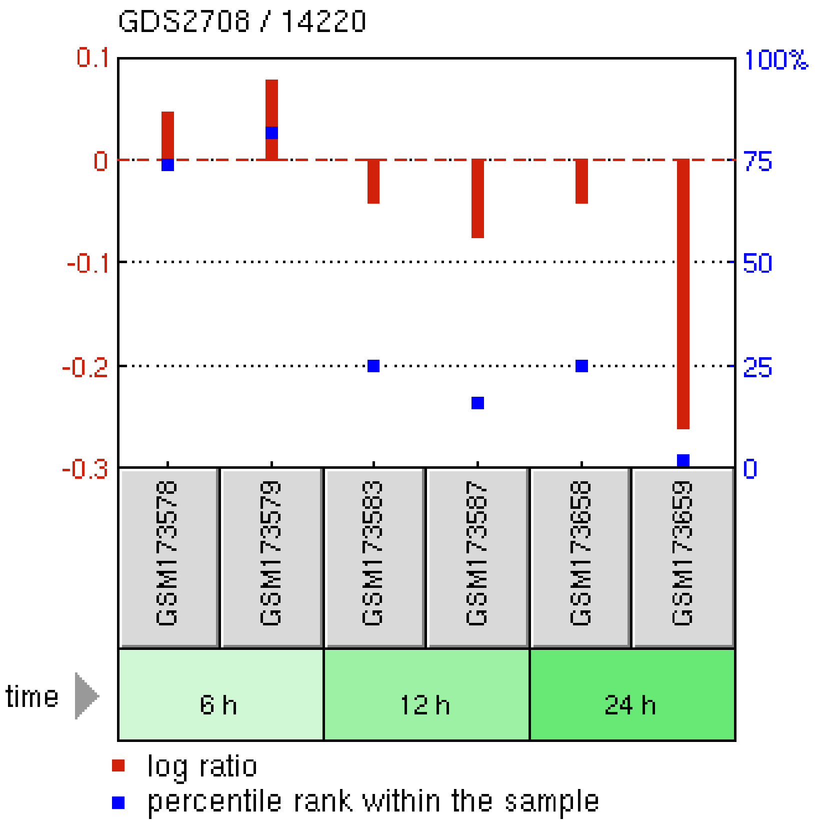

$p < 0.05$

Quercetin inhibits the *HMGA2* expression

Sample	Title	Value
GSM173578	Quercetin treated cardiomyocytes for 6 hours (Q6-A).	0.0475828
GSM173579	Quercetin treated cardiomyocytes for 6 hours (Q6-B).	0.0794489
GSM173583	Quercetin treated cardiomyocytes for 12 hours (Q12-A).	-0.0439453
GSM173587	Quercetin treated cardiomyocytes for 12 hours (Q12-B).	-0.078307
GSM173658	Quercetin treated cardiomyocytes for 24 hours (Q24-A).	-0.0439453
GSM173659	Quercetin treated cardiomyocytes for 24 hours (Q24-B).	-0.262591

Profile: *HMGA2* expression
GDS2628 / 1559891_at
Title
Vitamin D effect on bronchial smooth muscle cells
Organism
Homo sapiens

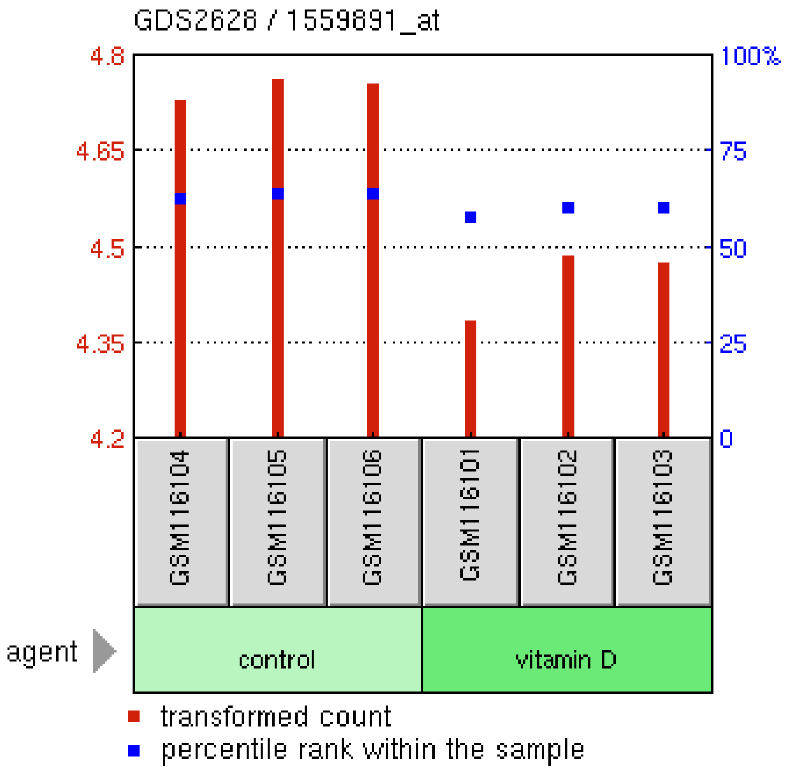

$p < 0.05$

Vitamin D inhibits the *HMGA2* expression

Sample	Title	Value
GSM116104	hBSMC_control_rep1	4.73008
GSM116105	hBSMC_control_rep2	4.7629
GSM116106	hBSMC_control_rep3	4.75495
GSM116101	hBSMC_vitamin D_rep1	4.38522
GSM116102	hBSMC_vitamin D_rep2	4.48749
GSM116103	hBSMC_vitamin D_rep3	4.47664

Profile: *HMGA2* expression
GDS2628 / 208025_s_at
Title
Vitamin D effect on bronchial smooth muscle cells
Organism
Homo sapiens

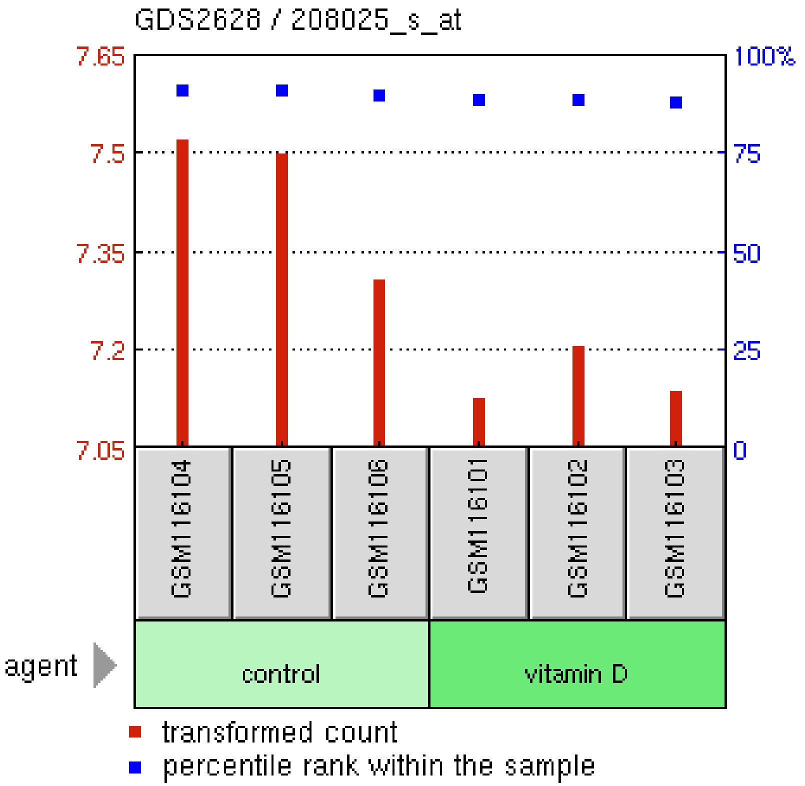

$p < 0.05$

Vitamin D inhibits the *HMGA2* expression

Sample	Title	Value
GSM116104	hBSMC_control_rep1	7.52243
GSM116105	hBSMC_control_rep2	7.50027
GSM116106	hBSMC_control_rep3	7.3098
GSM116101	hBSMC_vitamin D_rep1	7.12949
GSM116102	hBSMC_vitamin D_rep2	7.20614
GSM116103	hBSMC_vitamin D_rep3	7.13995

Profile: ACE2 expression
GDS4809 / 1425103_at
Title
Gata5 deficiency effect on the lung
Organism
Mus musculus

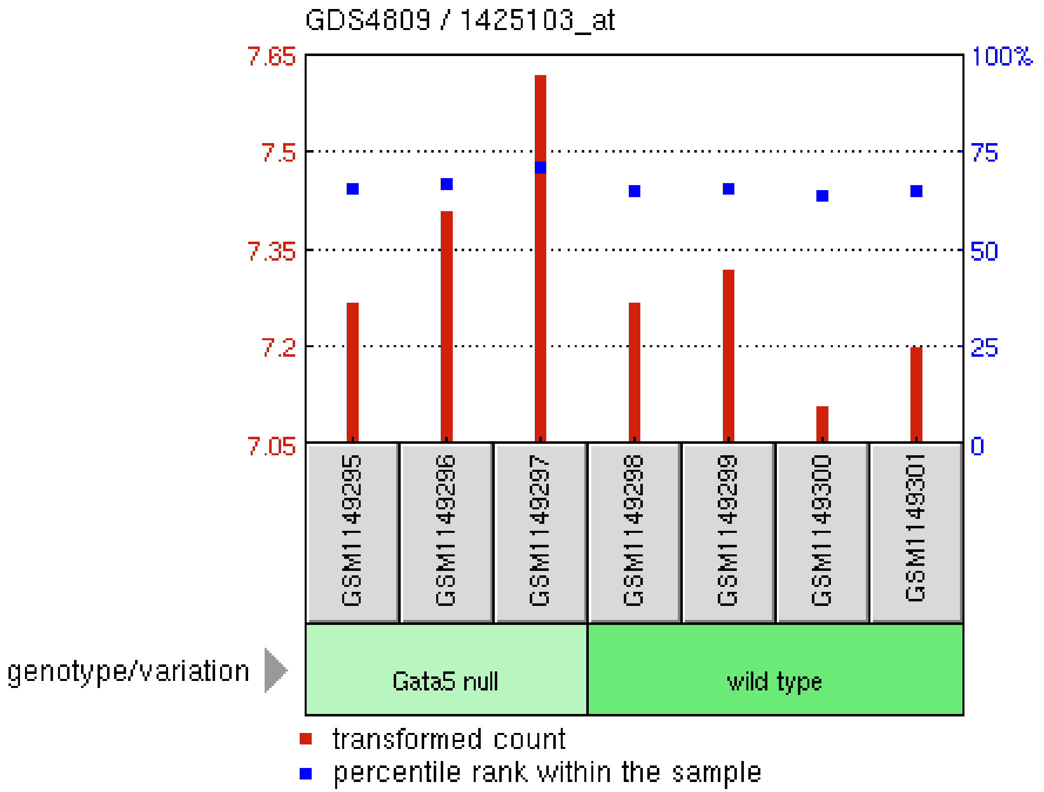

$p < 0.05$

GATA5: a candidate inhibitor of the *ACE2* expression

Sample	Title	Value
GSM1149295	KO618	7.27
GSM1149296	KO621	7.41
GSM1149297	KO624	7.62
GSM1149298	WT619	7.27
GSM1149299	WT623	7.32
GSM1149300	WT648	7.11
GSM1149301	WT659	7.2

Profile: *VDR* expression
GDS4809 / 1418176_at
Title
Gata5 deficiency effect on the lung
Organism
Mus musculus

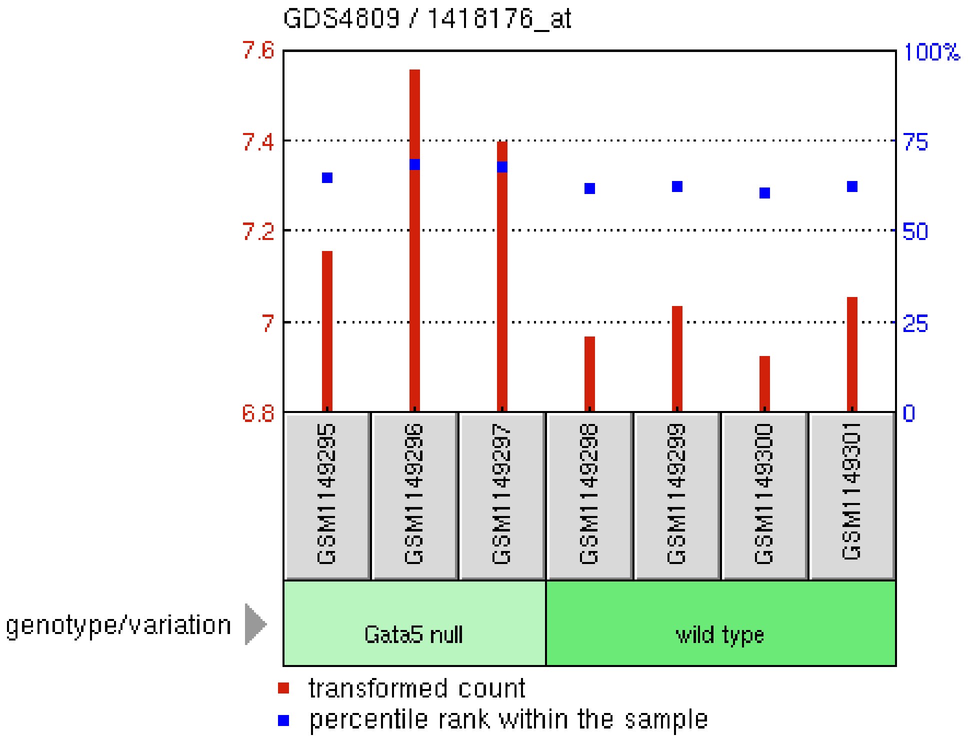

$p < 0.05$

GATA5: a candidate inhibitor of the *VDR* expression

Sample	Title	Value
GSM1149295	KO618	7.16
GSM1149296	KO621	7.56
GSM1149297	KO624	7.4
GSM1149298	WT619	6.97
GSM1149299	WT623	7.04
GSM1149300	WT648	6.93
GSM1149301	WT659	7.06

Profile: *SFTPC* expression
GDS4809 / 1418639_at
Title
Gata5 deficiency effect on the lung
Organism
Mus musculus

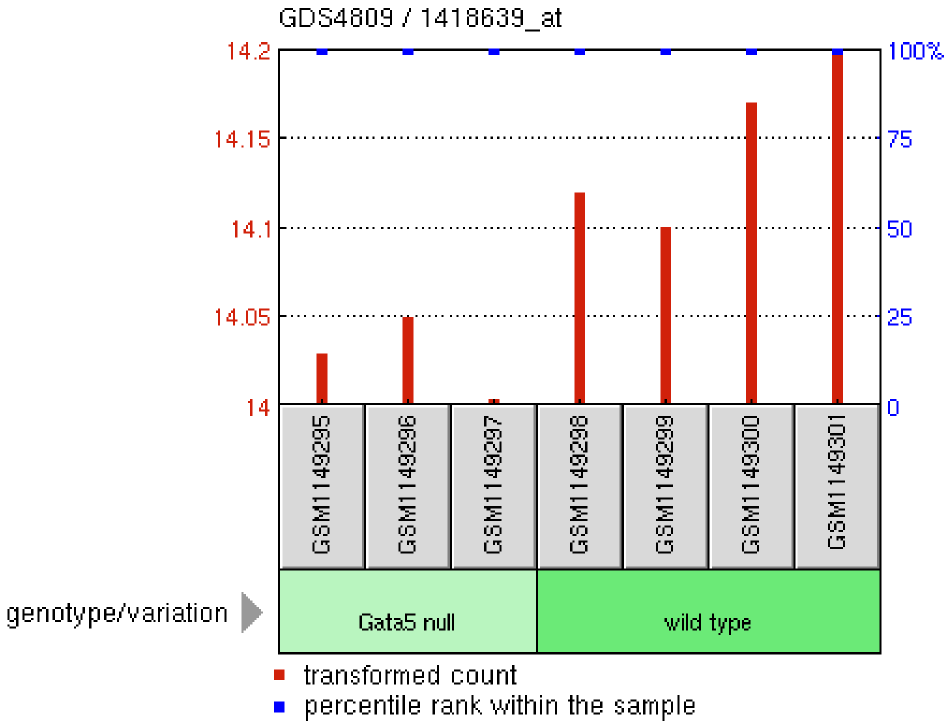

$p < 0.05$

GATA5: a candidate activator of the *SFTPC* expression

Sample	Title	Value
GSM1149295	KO618	14.03
GSM1149296	KO621	14.05
GSM1149297	KO624	14
GSM1149298	WT619	14.12
GSM1149299	WT623	14.1
GSM1149300	WT648	14.17
GSM1149301	WT659	14.2

Profile: GATA5 expression
GDS2708 / 15347
Title
Quercetin effect on cultured cardiomyocytes: time course
Organism
Rattus norvegicus

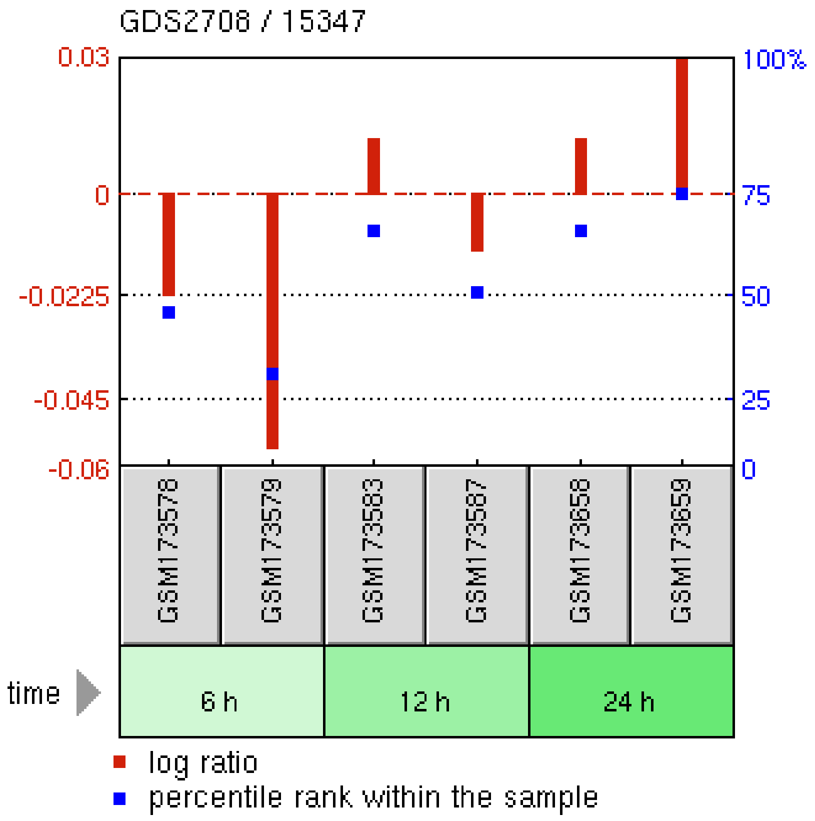

$p < 0.05$

Quercetin enhances the *GATA5* expression

Sample	Title	Value
GSM173578	Quercetin treated cardiomyocytes for 6 hours (Q6-A).	-0.0227201
GSM173579	Quercetin treated cardiomyocytes for 6 hours (Q6-B).	-0.0564461
GSM173583	Quercetin treated cardiomyocytes for 12 hours (Q12-A).	0.0121804
GSM173587	Quercetin treated cardiomyocytes for 12 hours (Q12-B).	-0.0128478
GSM173658	Quercetin treated cardiomyocytes for 24 hours (Q24-A).	0.0121804
GSM173659	Quercetin treated cardiomyocytes for 24 hours (Q24-B).	0.0297642

Profile: *GATA5* expression
GDS2628 / 238095_at
Title
Vitamin D effect on bronchial smooth muscle cells
Organism
Homo sapiens

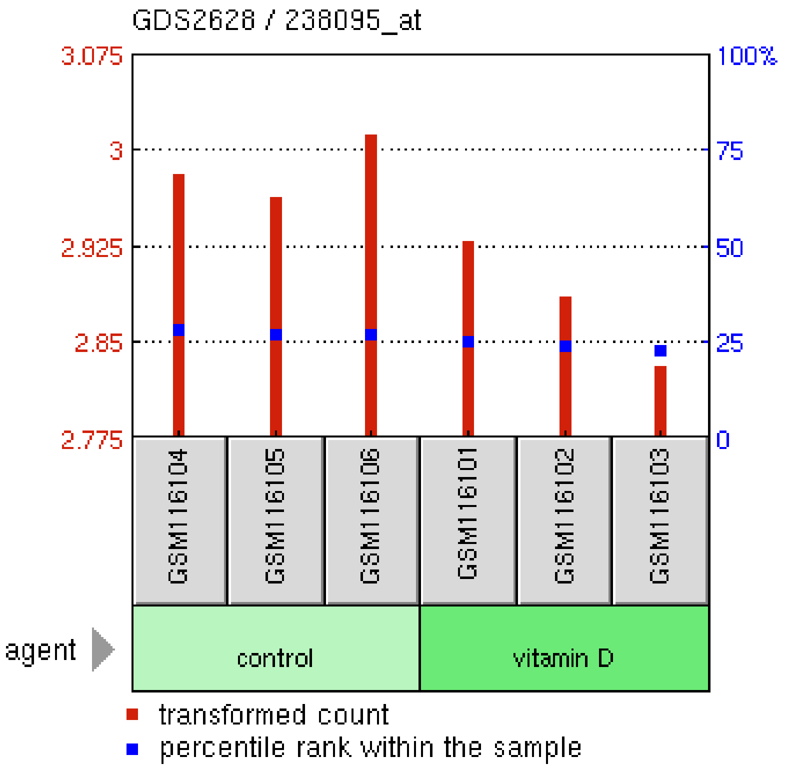

$p < 0.05$

Vitamin D inhibits the *GATA5* expression

Sample	Title	Value
GSM116104	hBSMC_control_rep1	2.98202
GSM116105	hBSMC_control_rep2	2.96376
GSM116106	hBSMC_control_rep3	3.01345
GSM116101	hBSMC_vitamin D_rep1	2.92934
GSM116102	hBSMC_vitamin D_rep2	2.88574
GSM116103	hBSMC_vitamin D_rep3	2.83171

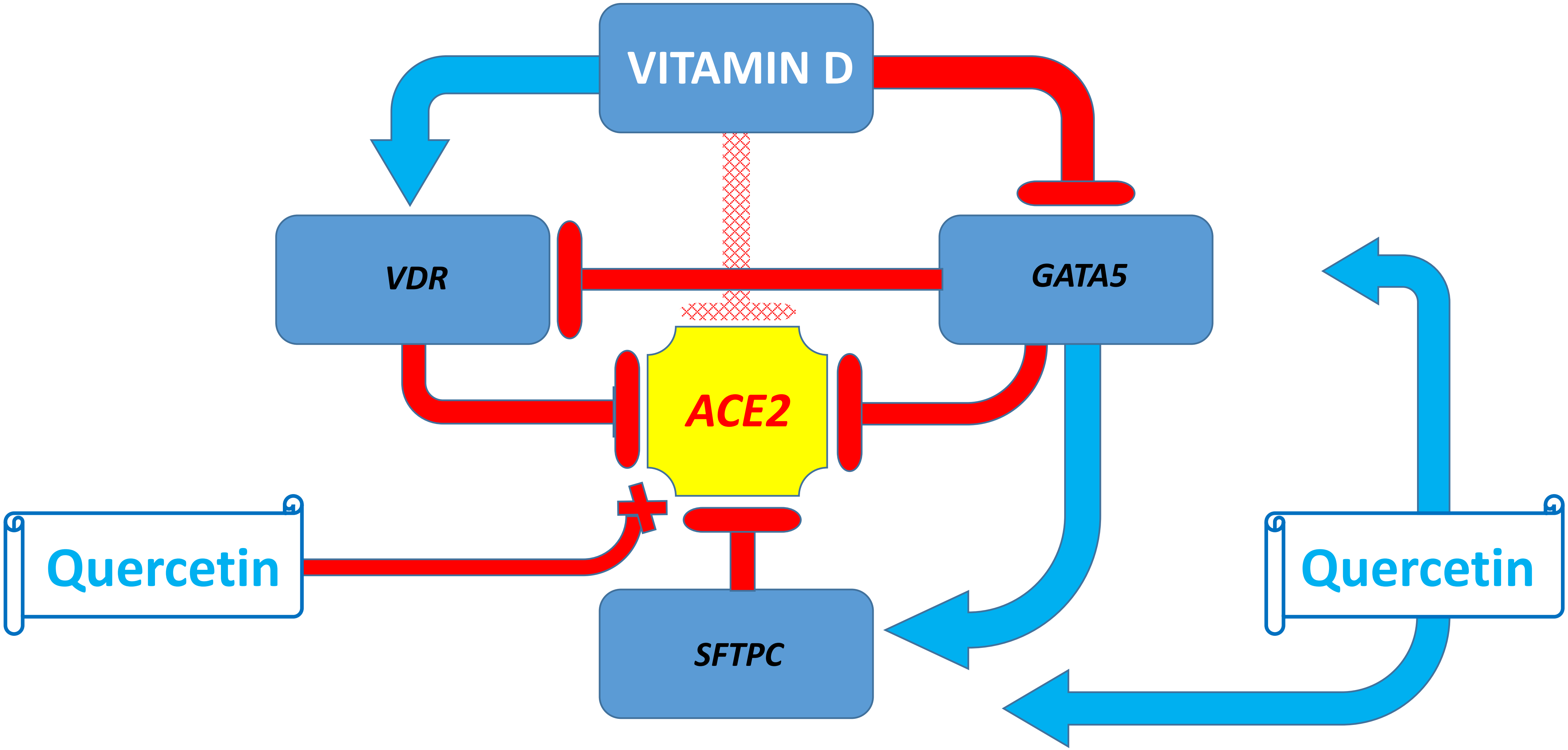
VITAMIN D
VDR
GATA5
ACE2
Quercetin
SFTPC
Quercetin

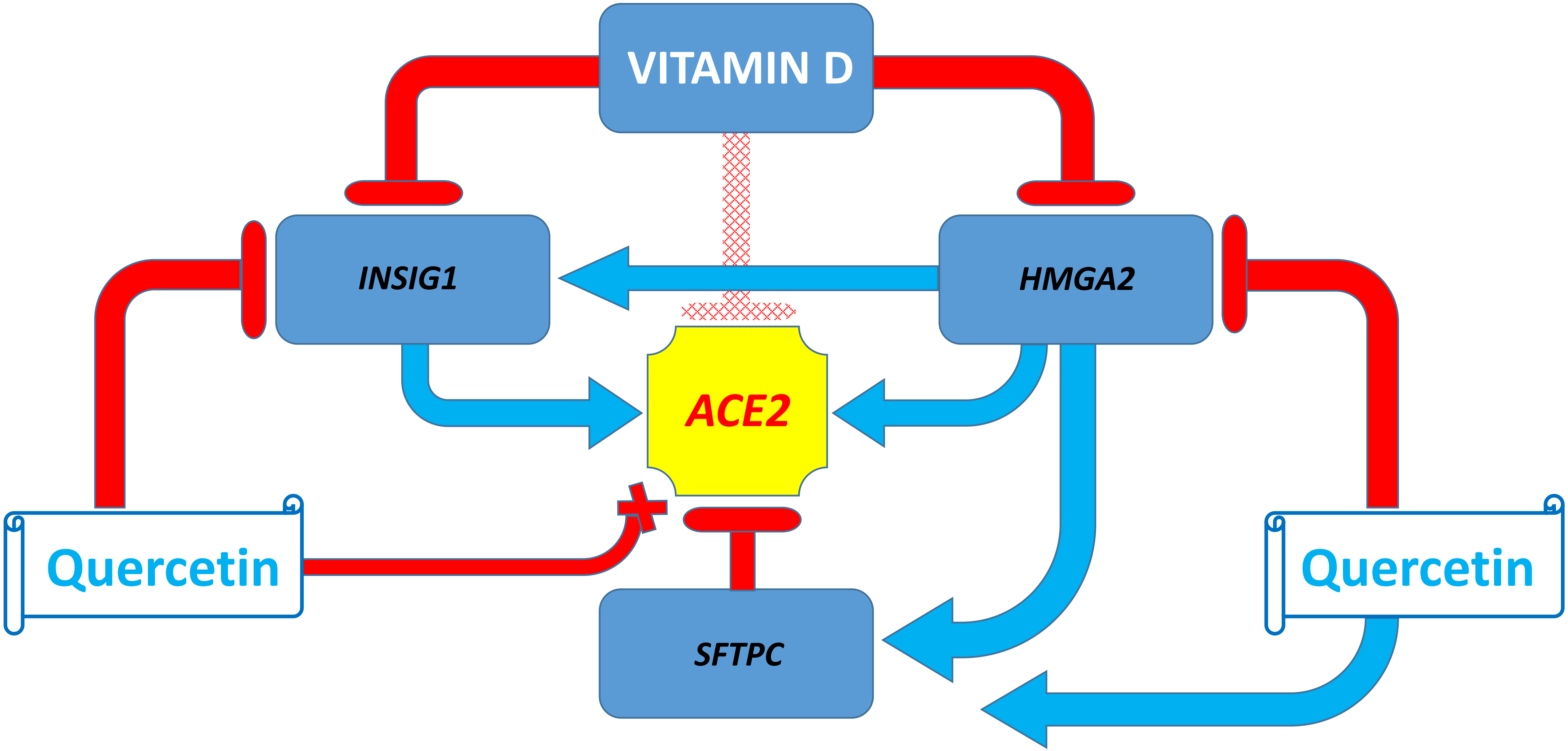
VITAMIN D
INSIG1
HMGA2
ACE2
SFTPC
Quercetin
Quercetin

Profile: *SFTPC* expression
GDS4809 / 1438290_x_at
Title
Gata5 deficiency effect on the lung
Organism
Mus musculus

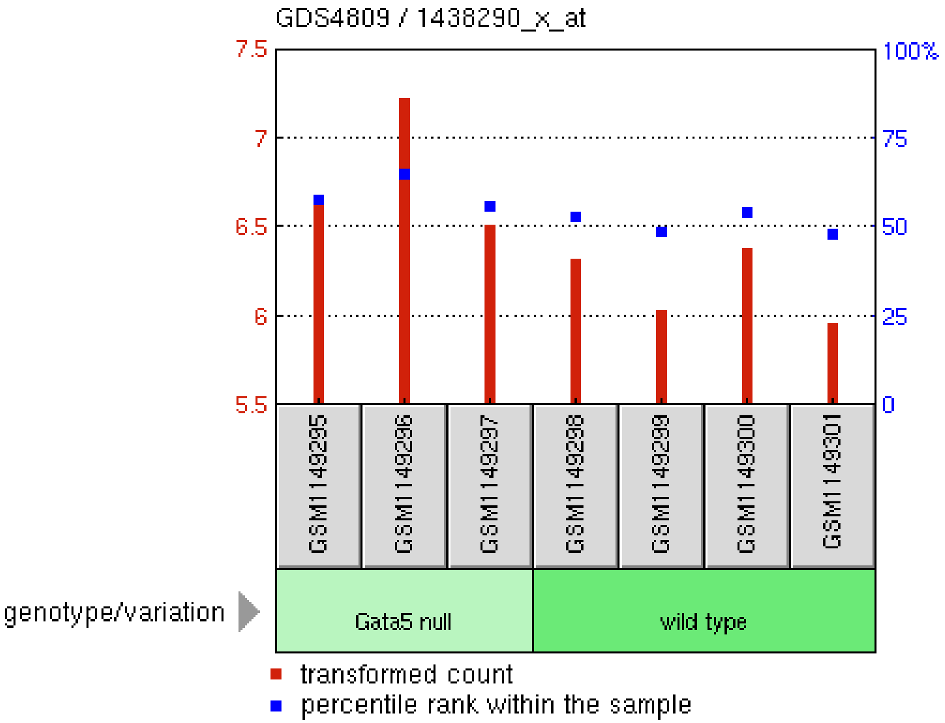

$p < 0.05$

GATA5: a candidate inhibitor of the *SFTPC* expression

Sample	Title	Value
GSM1149295	KO618	6.65
GSM1149296	KO621	7.23
GSM1149297	KO624	6.52
GSM1149298	WT619	6.32
GSM1149299	WT623	6.04
GSM1149300	WT648	6.39
GSM1149301	WT659	5.96

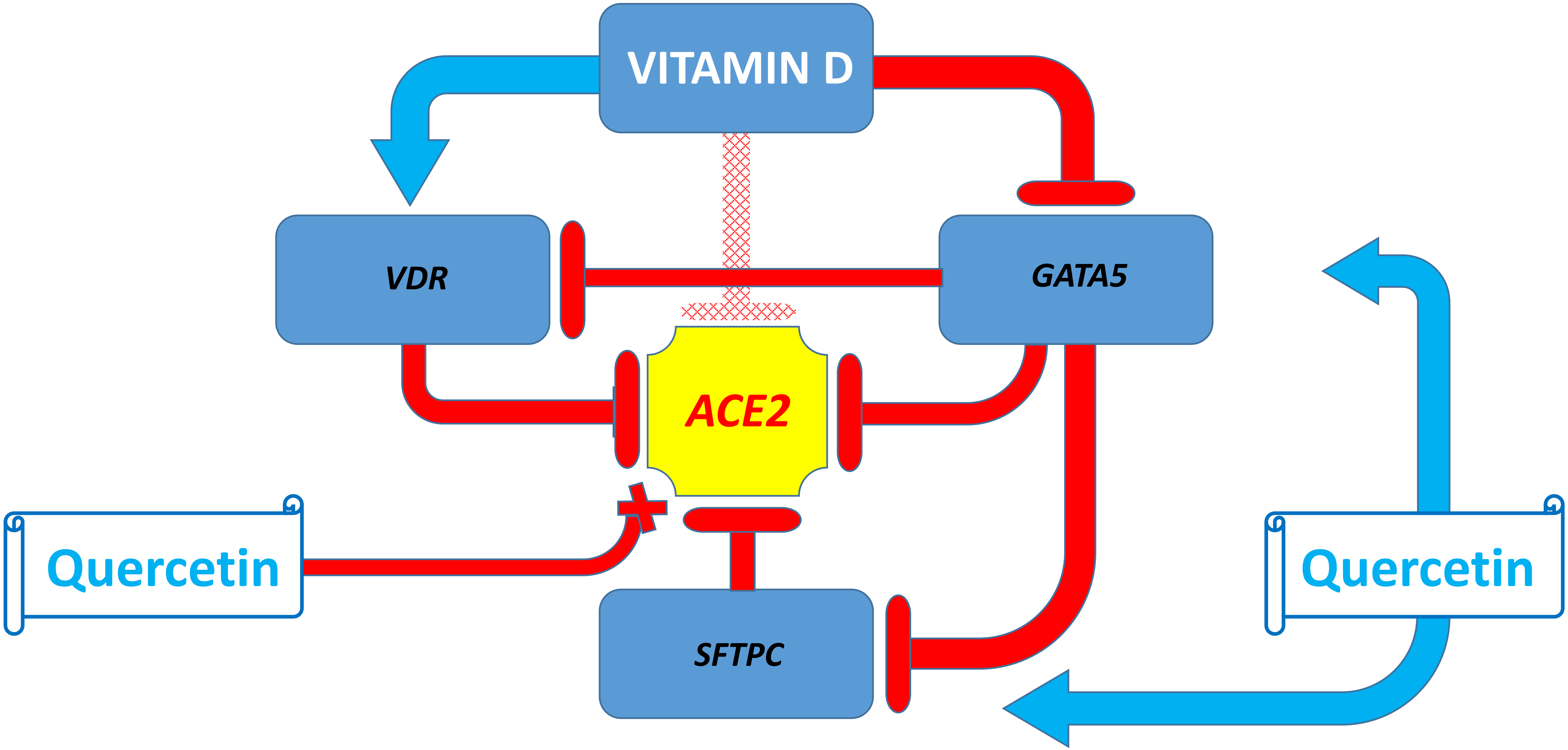
VITAMIN D
VDR
GATA5
ACE2
SFTPC
Quercetin
Quercetin

Examples of the potential negative effects of drugs on the ACE2 expression

Drug Perturbations from GEO up

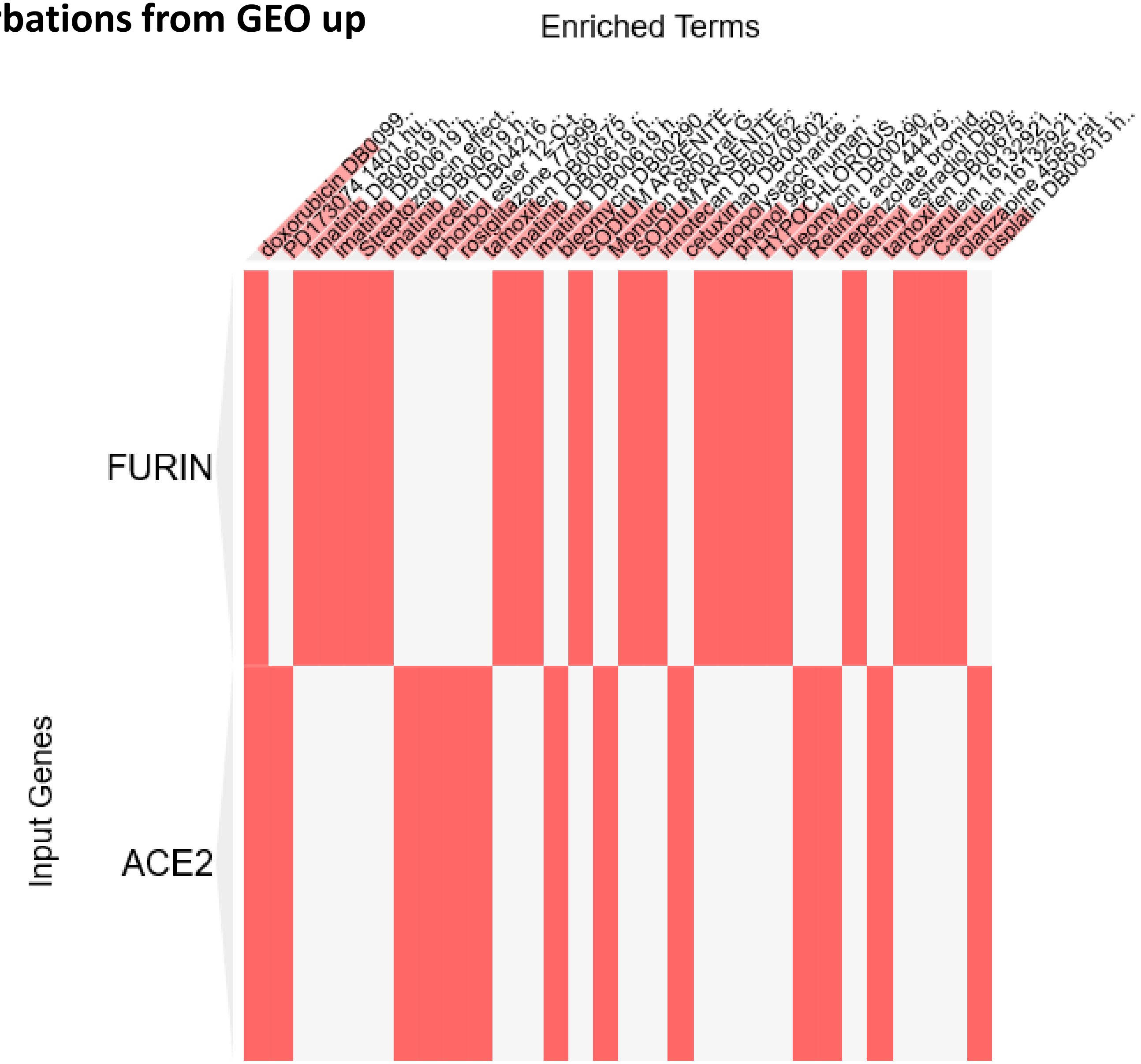

Profile *INSIG1* expression
GDS3032 / 201625_s_at
Title
Quercetin effect on intestinal cell differentiation in vitro: time course
Organism
Homo sapiens

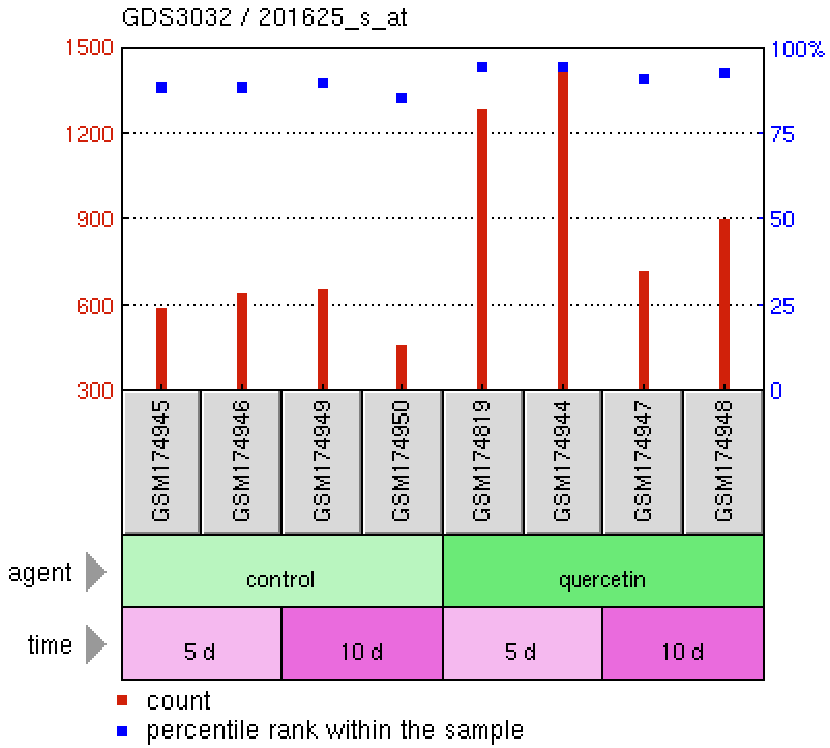

Quercetin enhances the *INSIG1* expression

Sample	Title	Value
GSM174945	Control day 05 sample 1	593.419
GSM174946	Control day 05 sample 2	640.636
GSM174949	Control day 10 sample 1	**654.401**
GSM174950	Control day 10 sample 2	**465.967**
GSM174819	Quercetin day 05 sample 1	1286.16
GSM174944	Quercetin day 05 sample 2	1420.46
GSM174947	Quercetin day 10 sample 1	**722.644**
GSM174948	Quercetin day 10 sample 2	**905.527**

Profile: *INSIG1* expression
GDS2628 / 201627_s_at
Title
Vitamin D effect on bronchial smooth muscle cells
Organism
Homo sapiens

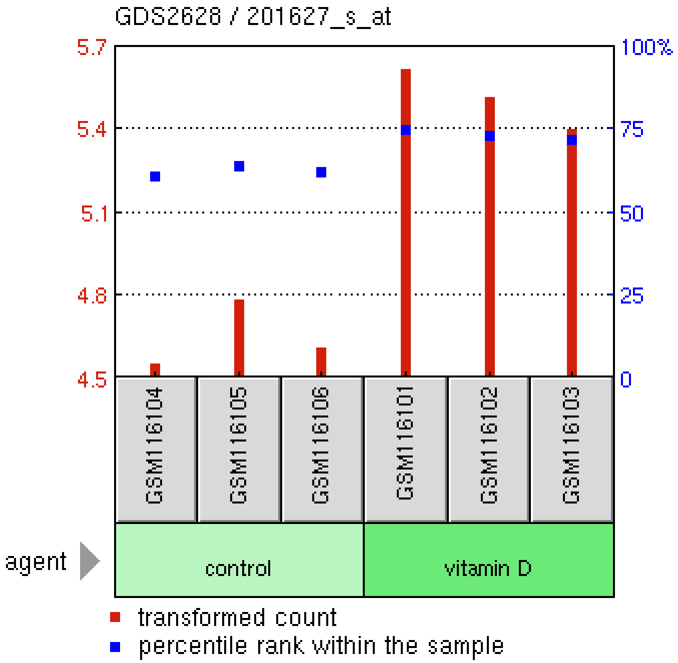

$p < 0.05$

Vitamin D enhances the *INSIG1* expression

Sample	Title	Value
GSM116104	hBSMC_control_rep1	4.5524
GSM116105	hBSMC_control_rep2	4.78368
GSM116106	hBSMC_control_rep3	4.6123
GSM116101	hBSMC_vitamin D_rep1	5.61848
GSM116102	hBSMC_vitamin D_rep2	5.51657
GSM116103	hBSMC_vitamin D_rep3	5.40349

Profile: *INSIG1* expression
GDS2628 / 201625_s_at
Title
Vitamin D effect on bronchial smooth muscle cells
Organism
Homo sapiens

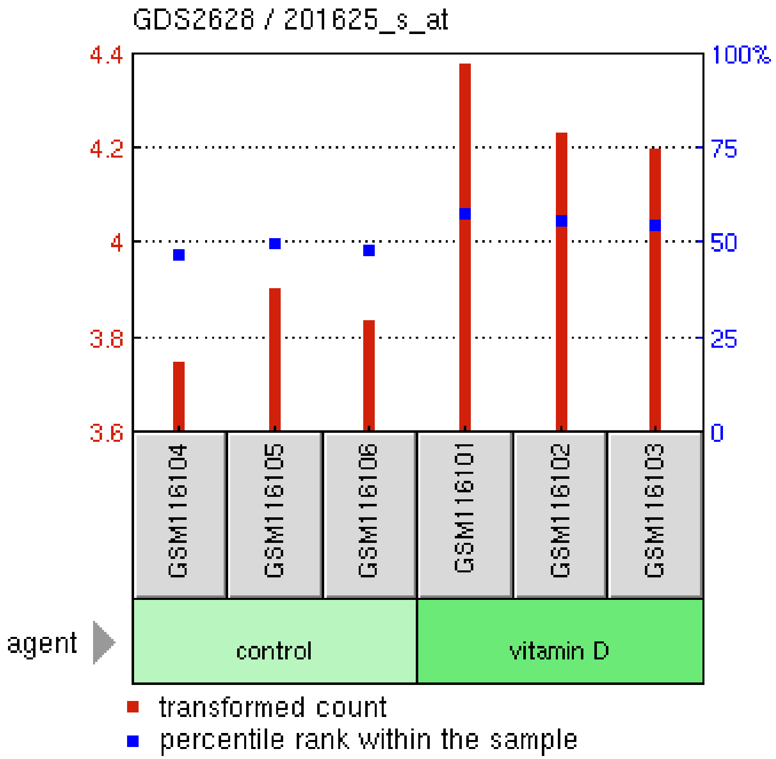

$p < 0.05$

Vitamin D enhances the *INSIG1* expression

Sample	Title	Value
GSM116104	hBSMC_control_rep1	3.75107
GSM116105	hBSMC_control_rep2	3.90763
GSM116106	hBSMC_control_rep3	3.83897
GSM116101	hBSMC_vitamin D_rep1	4.37812
GSM116102	hBSMC_vitamin D_rep2	4.2358
GSM116103	hBSMC_vitamin D_rep3	4.19935

Profile: *INSIG1* expression
GDS2628 / 201626_at
Title
Vitamin D effect on bronchial smooth muscle cells
Organism
Homo sapiens

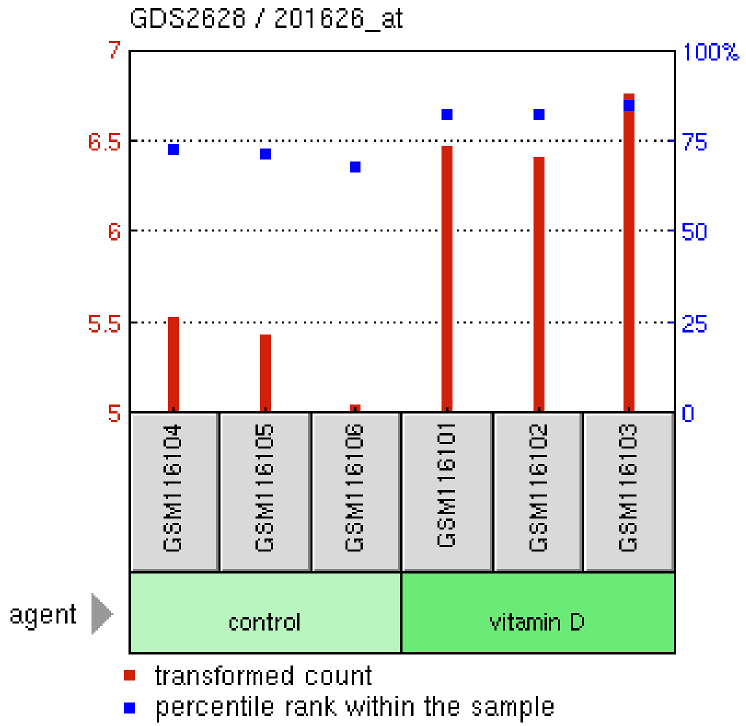

$p < 0.05$

Vitamin D enhances the *INSIG1* expression

Sample	Title	Value
GSM116104	hBSMC_control_rep1	5.53893
GSM116105	hBSMC_control_rep2	5.43847
GSM116106	hBSMC_control_rep3	5.01659
GSM116101	hBSMC_vitamin D_rep1	6.47195
GSM116102	hBSMC_vitamin D_rep2	6.42131
GSM116103	hBSMC_vitamin D_rep3	6.76932

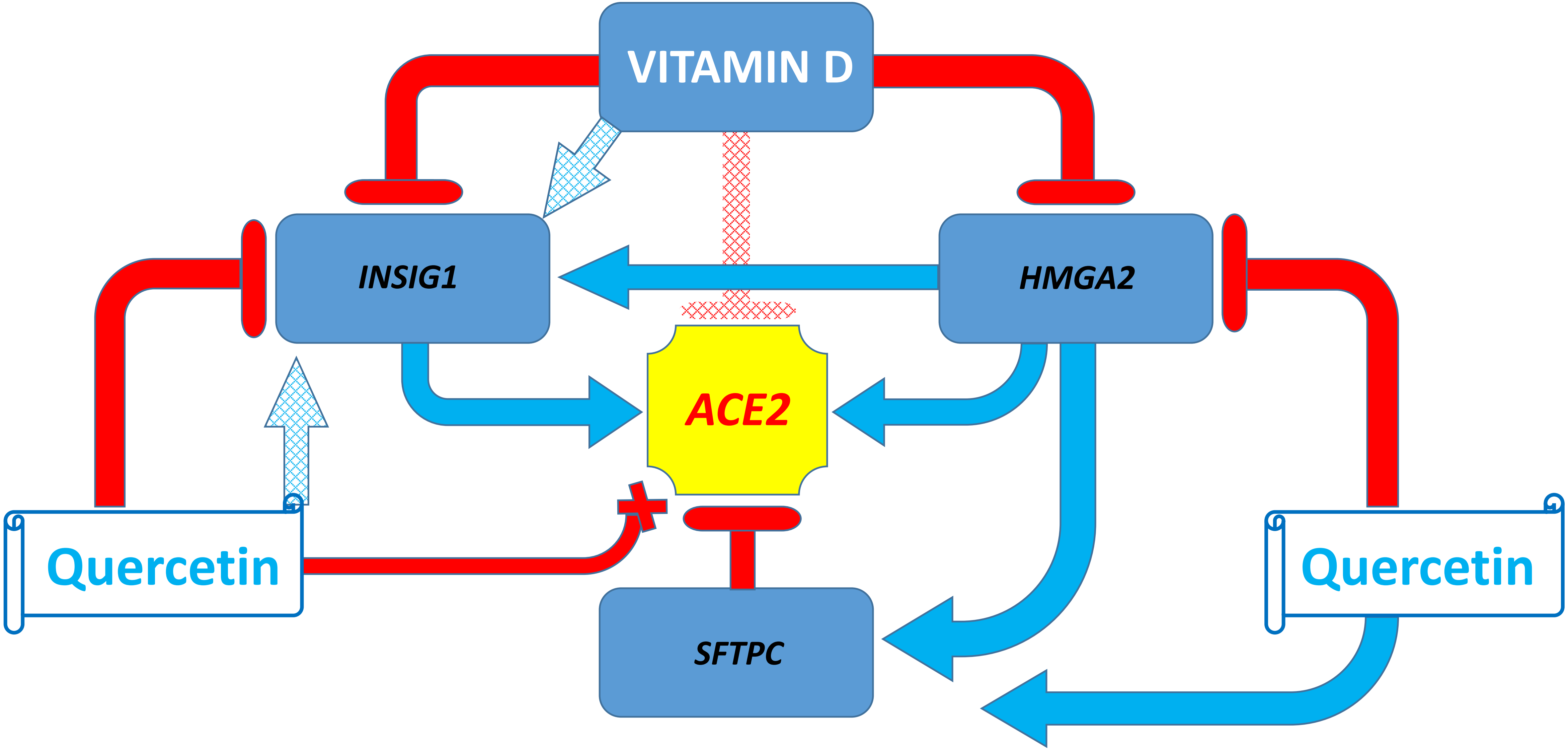
VITAMIN D
INSIG1
HMGA2
ACE2
SFTPC
Quercetin
Quercetin

Profile: ACE2 expression
GDS2314 / 1425102_a_at
Title
Antenatal steroid effect on the placenta
Organism
Mus musculus

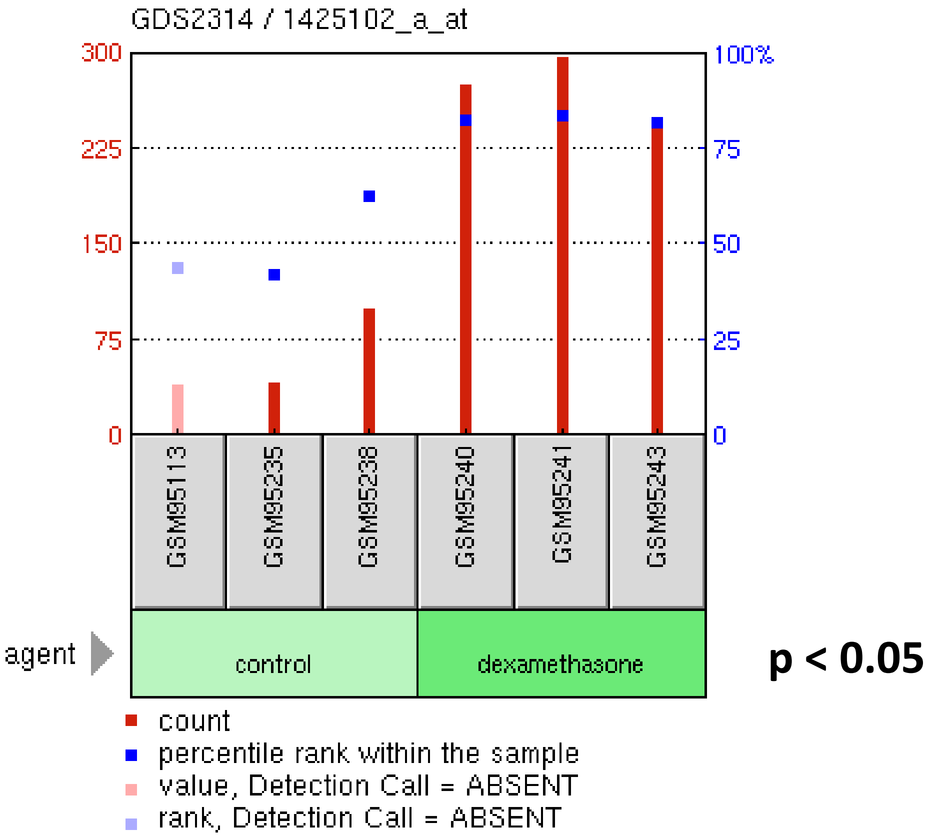

$p < 0.05$

Dexamethasone enhances the *ACE2* expression

Sample	Title	Value
GSM95113	Control 1	40.6
GSM95235	Control 2	42
GSM95238	Control 3	100.9
GSM95240	Dexamethasone 1	275.7
GSM95241	Dexamethasone 2	298
GSM95243	Dexamethasone 3	249.5

Profile: ACE2 expression
GDS2314 / 1425103_at
Title
Antenatal steroid effect on the placenta
Organism
Mus musculus

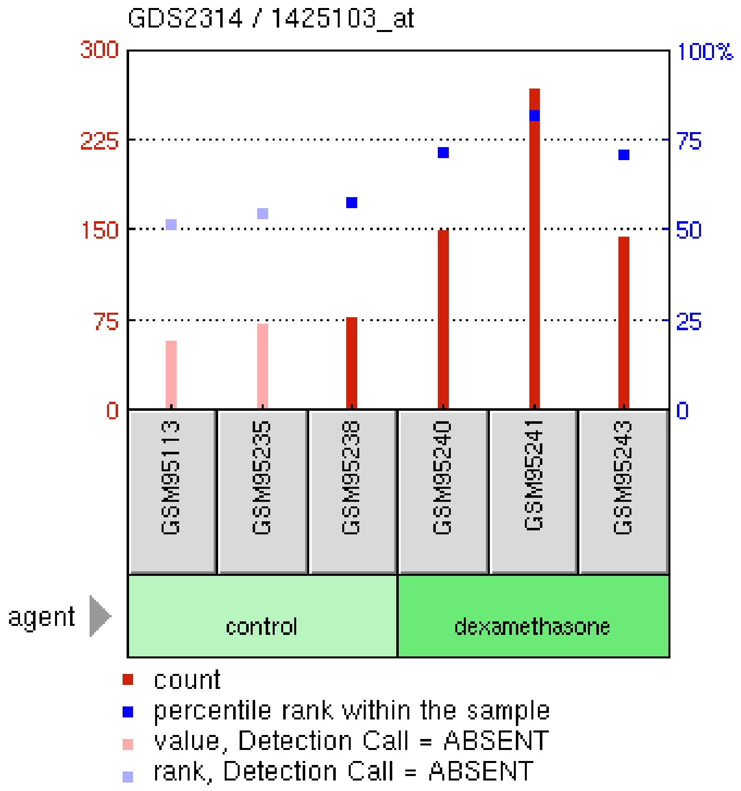

$p < 0.05$

Dexamethasone enhances the *ACE2* expression

Sample	Title	Value
GSM95113	Control 1	58
GSM95235	Control 2	73.3
GSM95238	Control 3	79.1
GSM95240	Dexamethasone 1	151
GSM95241	Dexamethasone 2	268.3
GSM95243	Dexamethasone 3	145.8

Profile: ACE2 expression
GDS2314 / 1452138_a_at
Title
Antenatal steroid effect on the placenta
Organism
Mus musculus

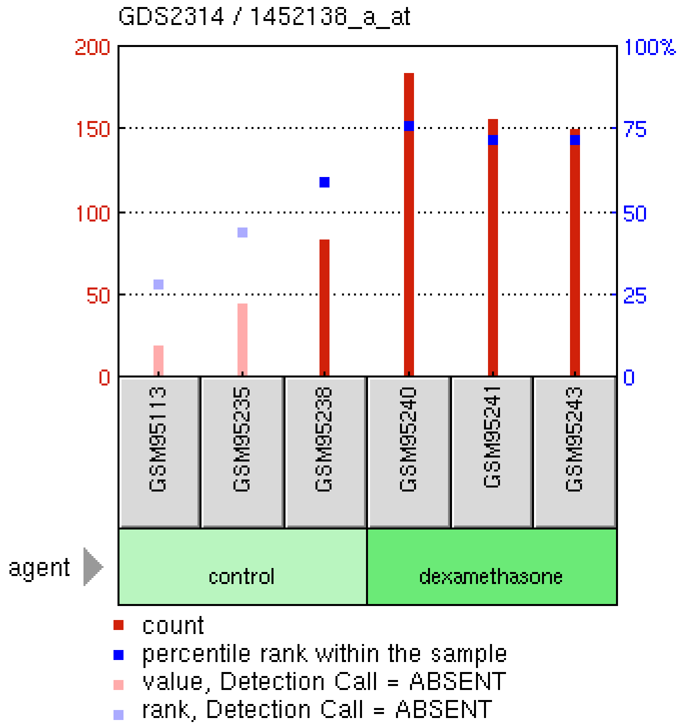

$p < 0.05$

Dexamethasone enhances the *ACE2* expression

Sample	Title	Value
GSM95113	Control 1	19.6
GSM95235	Control 2	45.5
GSM95238	Control 3	83.4
GSM95240	Dexamethasone 1	183.3
GSM95241	Dexamethasone 2	155.8
GSM95243	Dexamethasone 3	150

Supplemental Table S13. Additional examples of the potential activators and repressors of the *ACE2* expression identified using transgenic mouse models

Additional examples of the potential activators of the *ACE2* expression identified using transgenic mouse models

Profile: ACE2 expression
GDS4384 / 222257_s_at
Title
p53 mutations in microsatellite-stable, stage III colorectal cancer
Organism
Homo sapiens

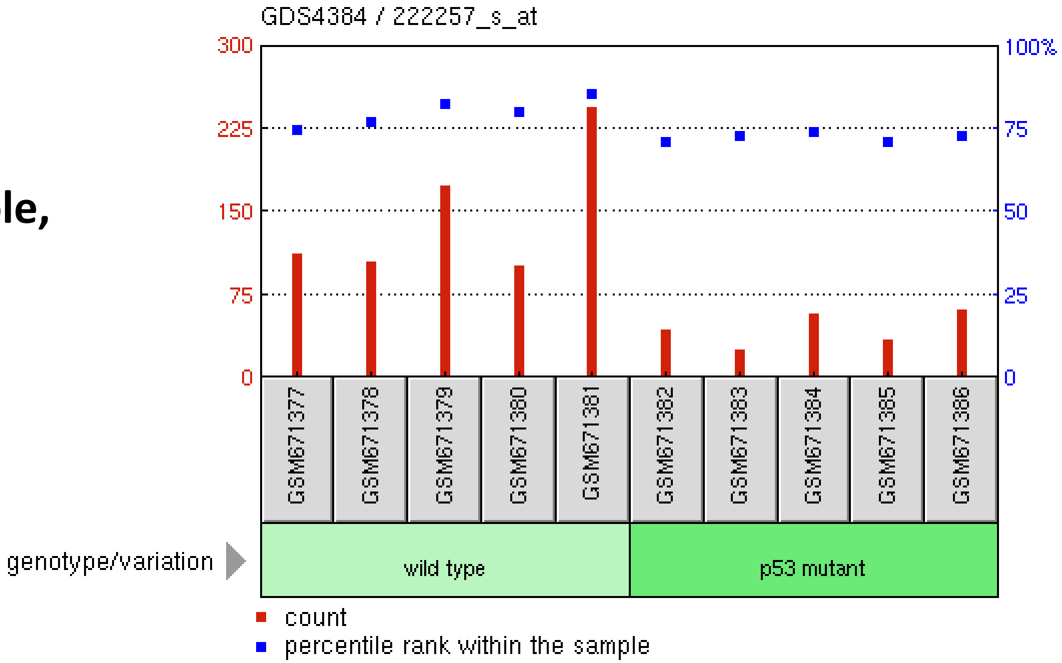

Sample	Title	Value
GSM671377	UM13_Wt_rep1	112.441
GSM671378	UM23_Wt_rep2	105.683
GSM671379	UM30_Wt_rep3	173.535
GSM671380	UM9_Wt_rep4	101.705
GSM671381	UM3_Wt_rep5	244.407
GSM671382	UM1_Mut_rep1	44.3901
GSM671383	UM15_Mtu_rep2	26.5371
GSM671384	UM17_Mtu_rep3	59.3536
GSM671385	UM2_Mtu_rep4	34.4729
GSM671386	UM25_Mtu_rep5	62.1878

Profile: ACE2 expression
GDS1434 / 160986_r_at
Title
Transcription factor p63 null mutation effect on skin (MG-U74A)
Organism
Mus musculus

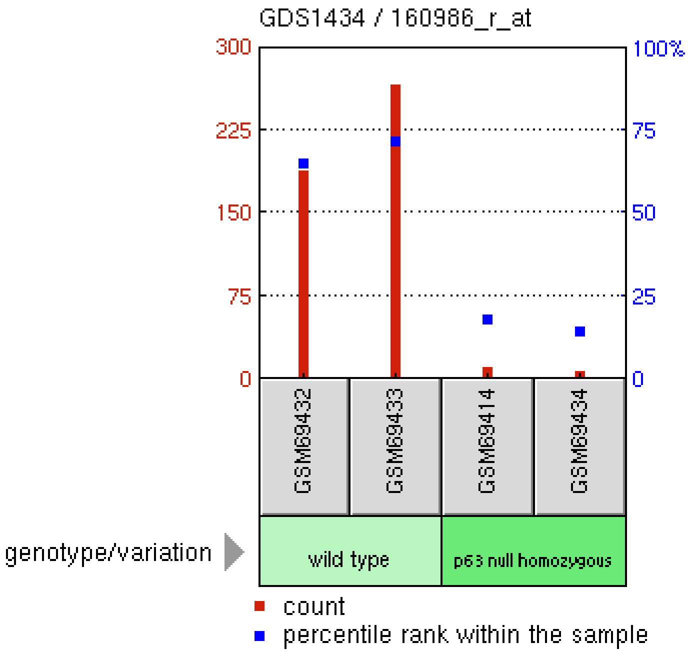

Sample	Title	Value
GSM69432	E18.5 p63 wt skin 1 A-chip	189.3
GSM69433	E18.5 p63 wt skin 2 A-chip	267.1
GSM69414	E18.5 p63 null skin 1 A-chip	11.5
GSM69434	E18.5 p63 null skin 2 A-chip	8.4

Profile: ACE2 expression
GDS1435 / 115071_at
Title
Transcription factor p63 null mutation effect on skin (MG-U74B)
Organism
Mus musculus

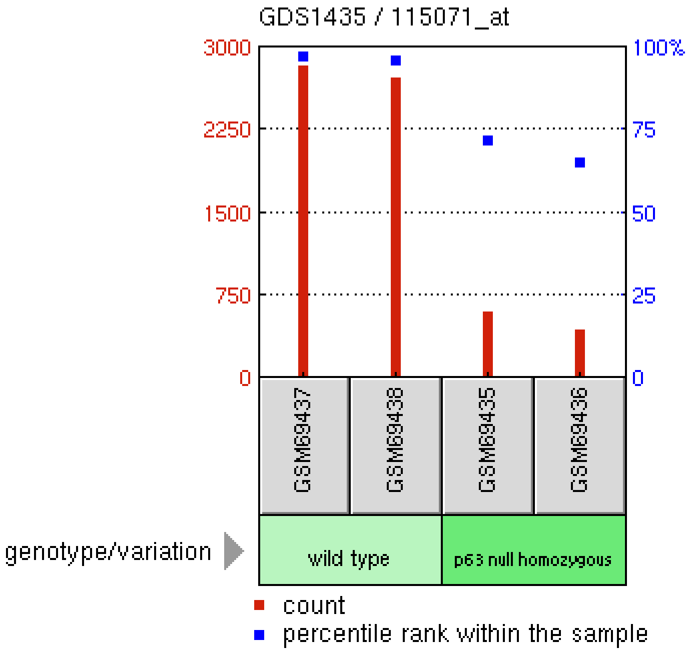

Sample	Title	Value
GSM69437	E18.5 wt skin 1 B-chip	2821.9
GSM69438	E18.5 wt skin 2 B-chip	2718.1
GSM69435	E18.5 p63 null skin 1 B-chip	602.2
GSM69436	E18.5 p63 null skin 2 B-chip	449.1

Profile: ACE2 expression
GDS4599 / 8171449
Title
ZNF750 silencing effect on differentiated keratinocytes
Organism
Homo sapiens

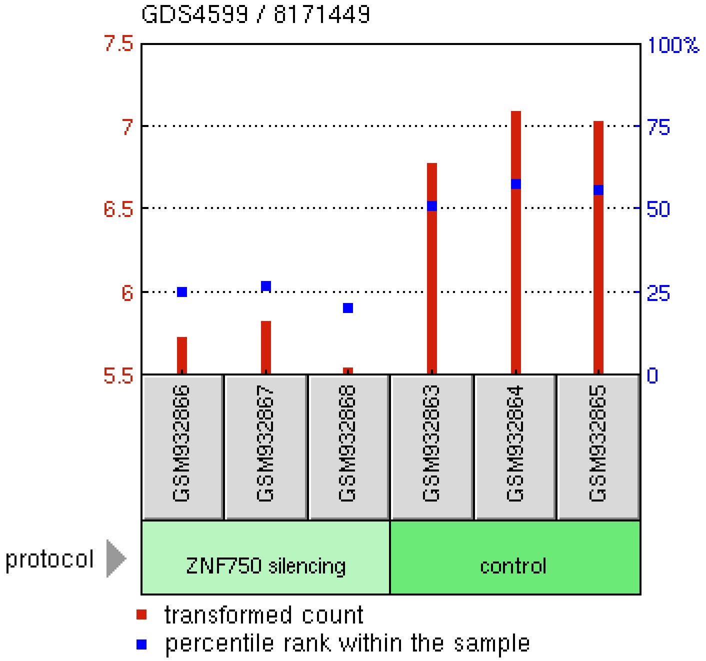

Sample	Title	Value
GSM932866	HaCaT_ZNF750i_ biological rep1	5.74045
GSM932867	HaCaT_ZNF750i_ biological rep2	5.82679
GSM932868	HaCaT_ZNF750i_ biological rep3	5.52392
GSM932863	HaCaT_Control_ biological rep1	6.78372
GSM932864	HaCaT_Control_ biological rep2	7.09307
GSM932865	HaCaT_Control_ biological rep3	7.03868

Profile: ACE2 expression
GDS4475 / 219962_at
Title
Extracellular matrix protein cysteine rich 61 (CCN1) effect on LN229 glioma cells
Organism
Homo sapiens

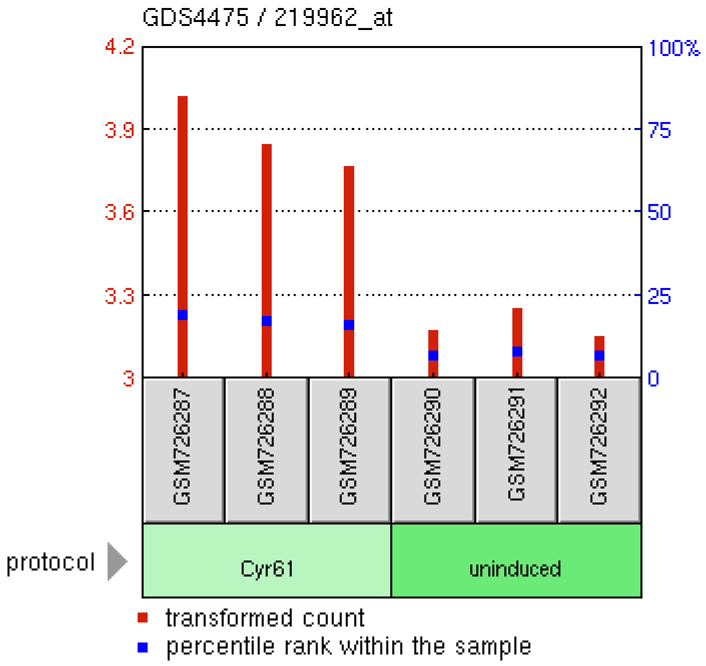

Sample	Title	Value
GSM726287	Tetracycline-inducible glioma cells dox 1	4.02176
GSM726288	Tetracycline-inducible glioma cells dox 2	3.85144
GSM726289	Tetracycline-inducible glioma cells dox 3	3.77096
GSM726290	Tetracycline-inducible glioma cells no dox 1	3.17831
GSM726291	Tetracycline-inducible glioma cells no dox 2	3.25989
GSM726292	Tetracycline-inducible glioma cells no dox 3	3.15658

Profile: ACE2 expression
GDS4061 / 222257_s_at
Title
Estrogen receptor alpha-silenced MCF7 breast cancer cells
Organism
Homo sapiens

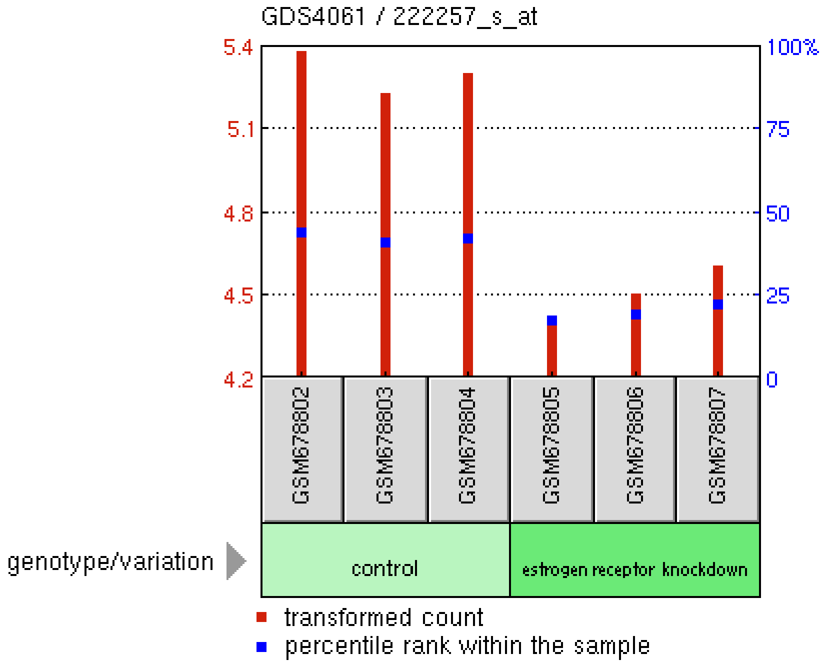

Sample	Title	Value
GSM678802	MCF7, biological rep1	5.37948
GSM678803	MCF7, biological rep2	5.23028
GSM678804	MCF7, biological rep3	5.30543
GSM678805	MCF7 silenced Estrogen receptor, biological rep1	4.40684
GSM678806	MCF7 silenced Estrogen receptor, biological rep2	4.50939
GSM678807	MCF7 silenced Estrogen receptor, biological rep3	4.609

Profile: ACE2 expression
GDS4061 / 219962_at
Title
Estrogen receptor alpha-silenced MCF7 breast cancer cells
Organism
Homo sapiens

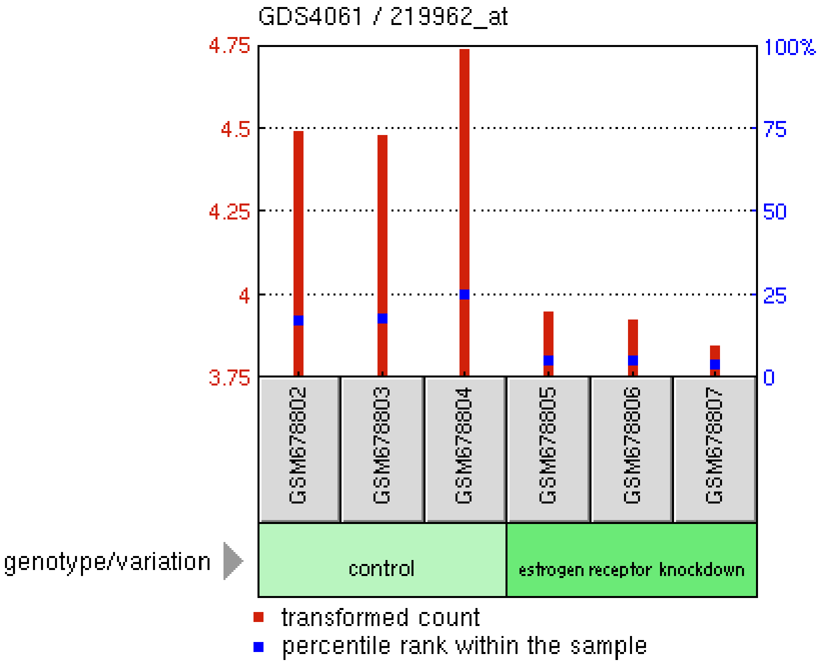

Sample	Title	Value
GSM678802	MCF7, biological rep1	4.49106
GSM678803	MCF7, biological rep2	4.48349
GSM678804	MCF7, biological rep3	4.73862
GSM678805	MCF7 silenced Estrogen receptor, biological rep1	3.94941
GSM678806	MCF7 silenced Estrogen receptor, biological rep2	3.9283
GSM678807	MCF7 silenced Estrogen receptor, biological rep3	3.84918

Profile: ACE2 expression
GDS4061 / 219962_at
Title
Estrogen receptor alpha-silenced MCF7 breast cancer cells
Organism
Homo sapiens

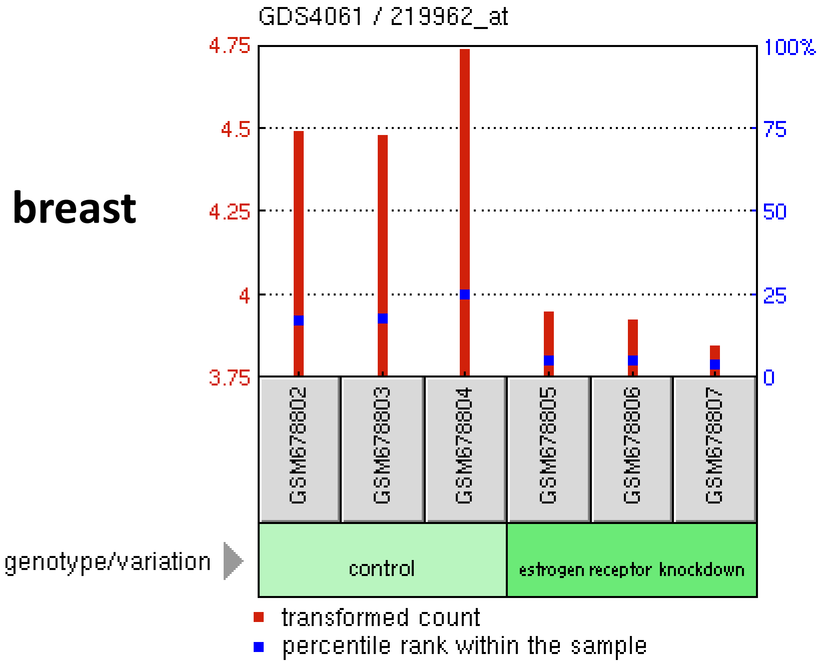

Sample	Title	Value
GSM678802	MCF7, biological rep1	4.49106
GSM678803	MCF7, biological rep2	4.48349
GSM678804	MCF7, biological rep3	4.73862
GSM678805	MCF7 silenced Estrogen receptor, biological rep1	3.94941
GSM678806	MCF7 silenced Estrogen receptor, biological rep2	3.9283
GSM678807	MCF7 silenced Estrogen receptor, biological rep3	3.84918

Profile: ACE2 expression
GDS1644 / 10205_at
Title
VTS1 deletion mutant
Organism
Saccharomyces cerevisiae

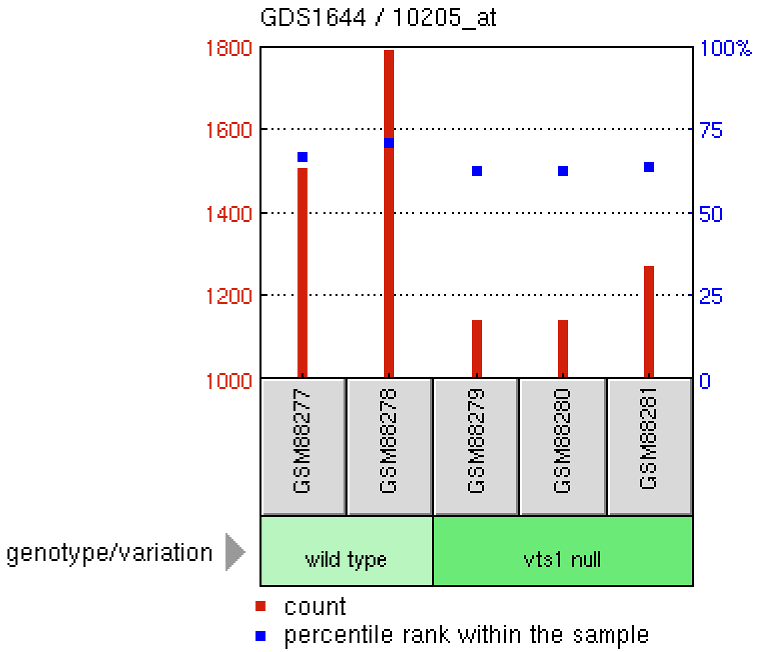

Sample	Title	Value
GSM88277	WT1	1506.8
GSM88278	WT2	1791
GSM88279	Exp Pt 1(dvts1-1 VTS1)	1141.9
GSM88280	Exp Pt 2(dvts1-2 VTS1)	1143.6
GSM88281	Exp Pt 3(VTS1)	1271.5

Profile: ACE2 expression
GDS3294 / 1425103_at
Title
CTCF depletion effect in oocytes
Organism
Mus musculus

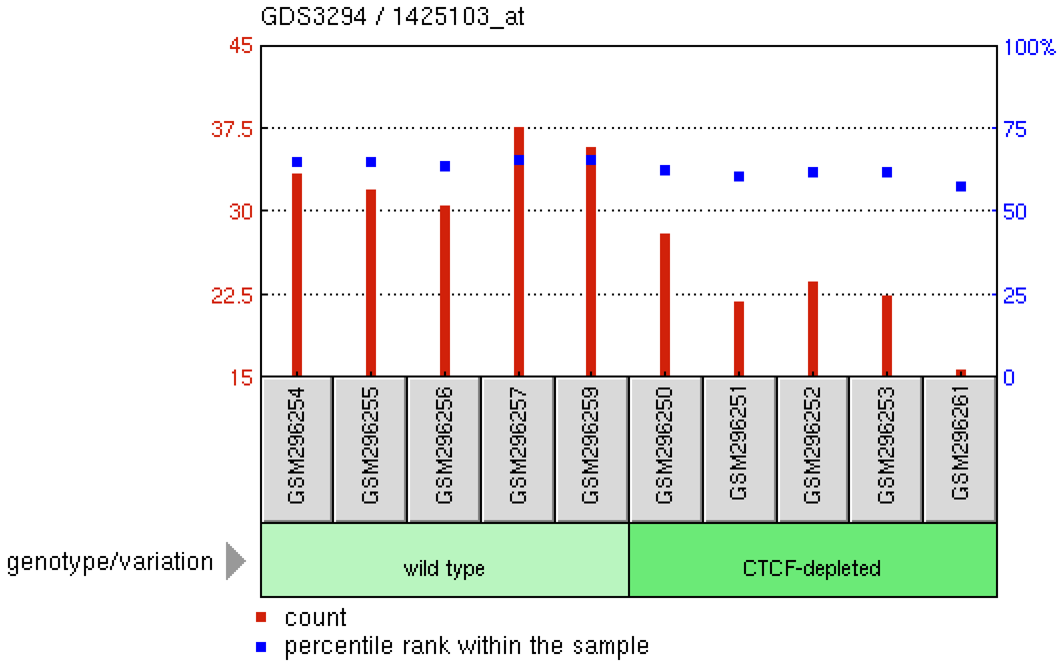

Sample	Title	Value
GSM296254	gene expression of wild type oocyte, replicate 1	33.5935
GSM296255	gene expression of wild type oocyte, replicate 2	32.1628
GSM296256	gene expression of wild type oocyte, replicate 3	30.5551
GSM296257	gene expression of wild type oocyte, replicate 4	37.7155
GSM296259	gene expression of wild type oocyte, replicate 5	35.8225
GSM296250	gene expression of CTCF-depleted oocyte, replicate 1	28.1471
GSM296251	gene expression of CTCF-depleted oocyte, replicate 3	21.9578
GSM296252	gene expression of CTCF-depleted oocyte, replicate 4	23.7345
GSM296253	gene expression of CTCF-depleted oocyte, replicate 5	22.4628
GSM296261	gene expression of CTCF-depleted oocyte, replicate 2	15.8604

Profile: ACE2 expression
GDS1555 / 1425103_at
Title
Glycerol kinase knockout effect on liver
Organism
Mus musculus

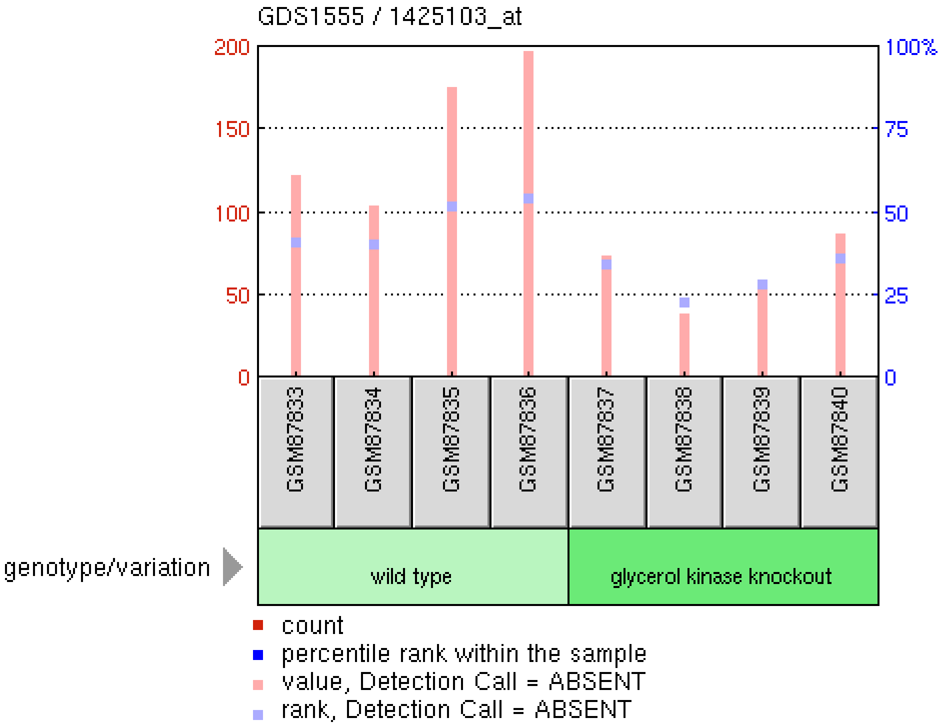

Sample	Title	Value
GSM87833	Liver_WildType_1	122.1
GSM87834	Liver_WildType_2	104.3
GSM87835	Liver_WildType_3	174.9
GSM87836	Liver_WildType_4	197.4
GSM87837	Liver_KnockOut_1	74
GSM87838	Liver_KnockOut_2	39.3
GSM87839	Liver_KnockOut_3	59.1
GSM87840	Liver_KnockOut_4	87.3

Profile: ACE2 expression
GDS4895 / 1425102_a_at
Title
SIRT1 deficiency effect on the brain
Organism
Mus musculus

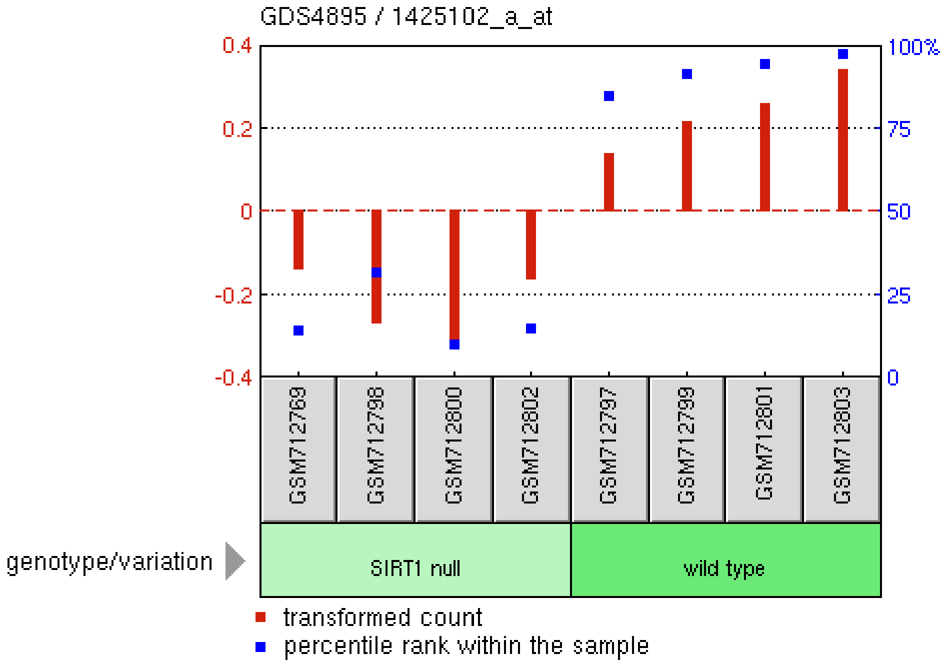

Sample	Title	Value
GSM712769	BSKO 1	-0.140546
GSM712798	BSKO 2	-0.27286
GSM712800	BSKO 3	-0.312008
GSM712802	BSKO 4	-0.167778
GSM712797	WT 1	0.140546
GSM712799	WT 2	0.221
GSM712801	WT 3	0.261022
GSM712803	WT 4	0.344256

Additional examples of the potential repressors of the *ACE2* expression identified using transgenic mouse models

Profile: ACE2 expression
GDS3361 / 160986_r_at
Title
Fibroblast growth factor-23 transgenic model
Organism
Mus musculus

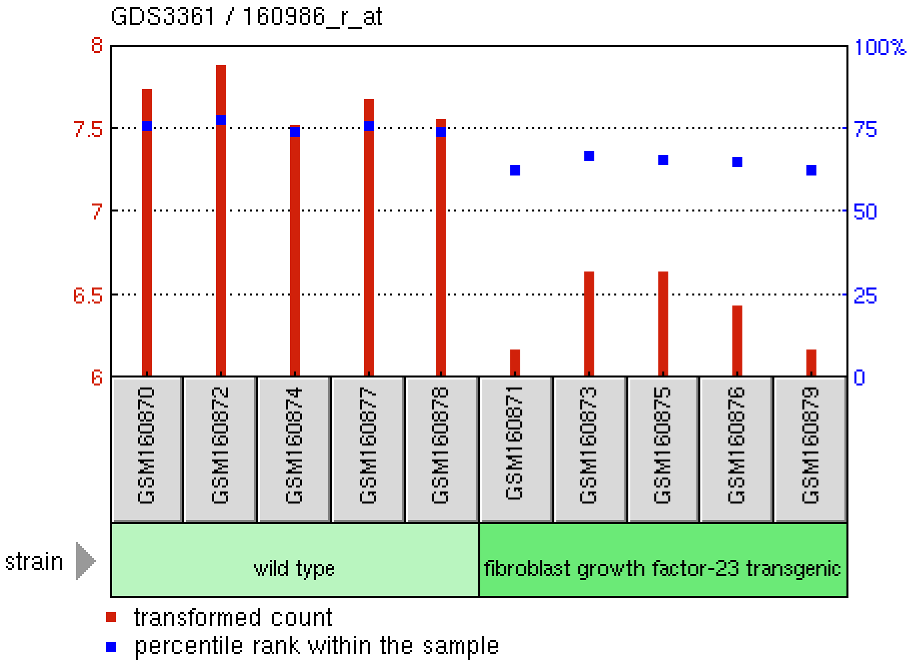

Sample	Title	Value
GSM160870	Wild Type 612	7.73651
GSM160872	Wild type 618	7.89066
GSM160874	Wild Type 650	7.5183
GSM160877	Wild Type nr 658	7.67757
GSM160878	Wild Type nr 663	7.55491
GSM160871	Transgenic nr 614	6.17722
GSM160873	Transgenic nr 619	6.64023
GSM160875	Transgenic nr 652	6.64915
GSM160876	Transgenic nr 657	6.44524
GSM160879	Transgenic nr 661	6.17914

Profile: ACE2 expression
GDS2359 / 1452138_a_at
Title
Interferon regulatory factor 6 null mutation effect on the skin
Organism
Mus musculus

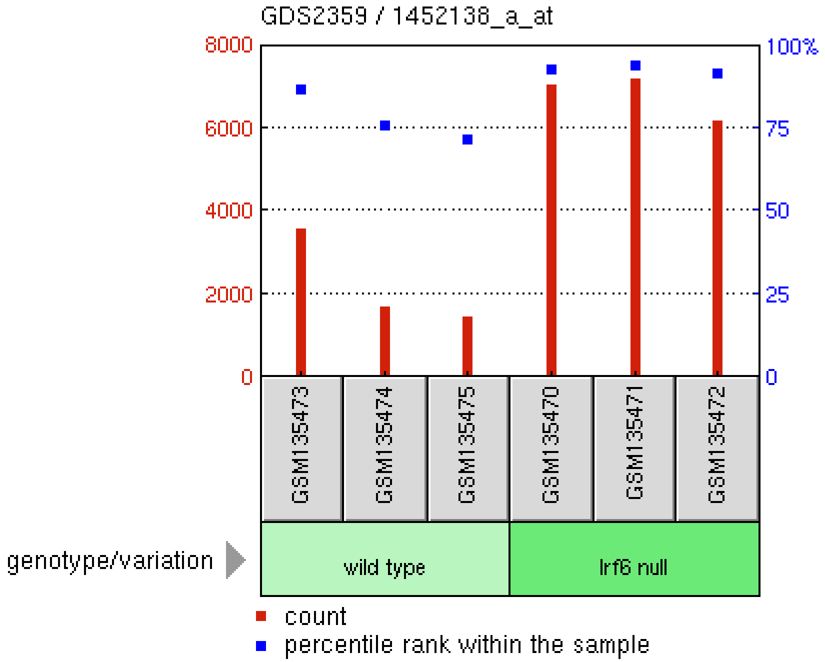

Sample	Title	Value
GSM135473	IRF6 wt, biological rep1	3607.2
GSM135474	IRF6 wt, biological rep2	1727.4
GSM135475	IRF6 wt, biological rep3	1461.4
GSM135470	IRF6 knockout, biological rep1	7077
GSM135471	IRF6 knockout, biological rep2	7212.6
GSM135472	IRF6 knockout, biological rep3	6184.5

Profile: ACE2 expression
GDS2359 / 1425103_at
Title
Interferon regulatory factor 6 null mutation effect on the skin
Organism
Mus musculus

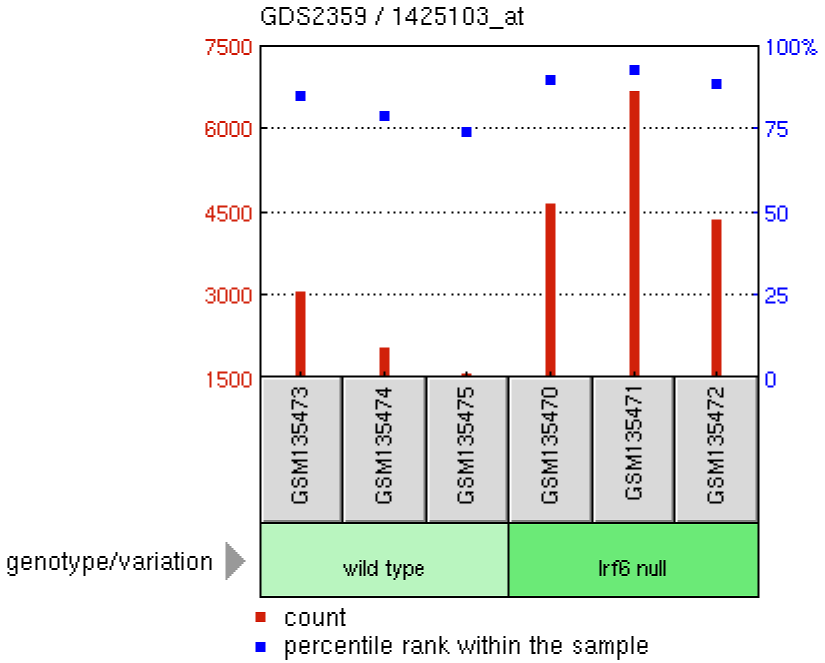

Sample	Title	Value
GSM135473	IRF6 wt, biological rep1	3067.9
GSM135474	IRF6 wt, biological rep2	2065.9
GSM135475	IRF6 wt, biological rep3	1589.4
GSM135470	IRF6 knockout, biological rep1	4678.8
GSM135471	IRF6 knockout, biological rep2	6695.8
GSM135472	IRF6 knockout, biological rep3	4381.7

Profile: ACE2 expression
GDS4498 / 1425102_a_at
Title
Transcription factor Nrf2 gain-of-function model: postnatal day 2.5 whole skin
Organism
Mus musculus

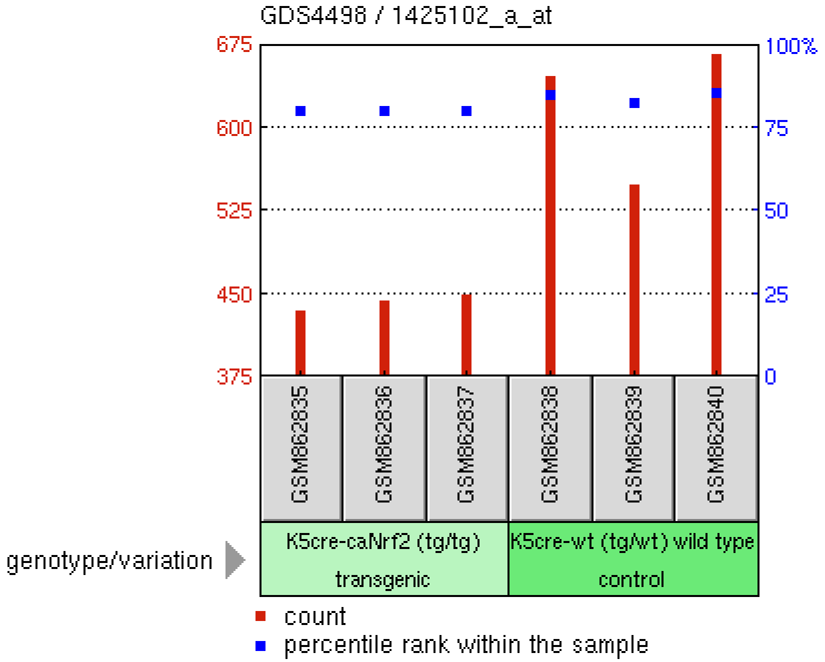

Sample	Title	Value
GSM862835	transgene strong 1	435.03
GSM862836	transgene strong 2	444.737
GSM862837	transgene strong 3	449.601
GSM862838	wildtype strong 1	647.134
GSM862839	wildtype strong 2	548.634
GSM862840	wildtype strong 3	667.753

Profile: ACE2 expression
GDS3397 / 1425102_a_at
Title
Dent disease model: renal proximal tubules
Organism
Mus musculus

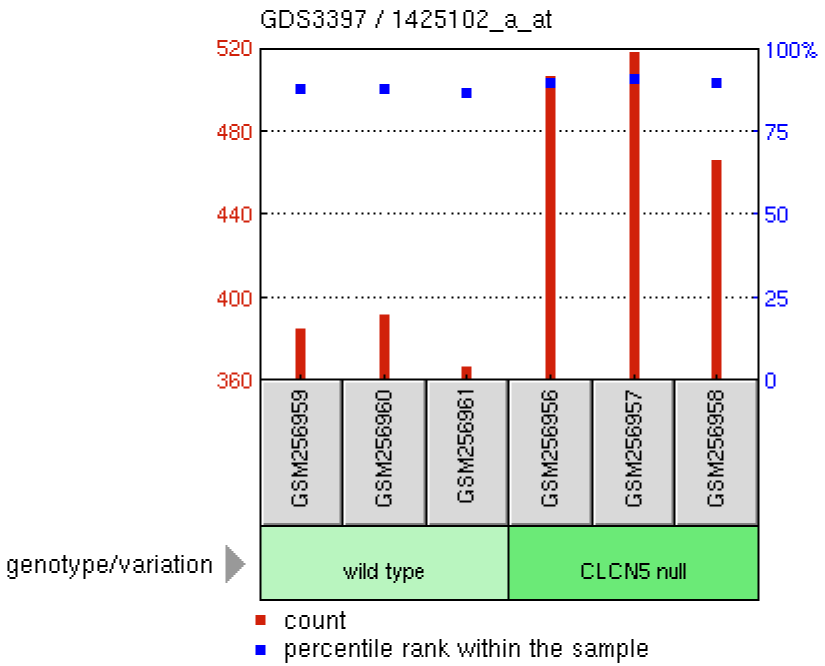

Sample	Title	Value
GSM256959	Wild type rep 1	385.878
GSM256960	Wild type rep 2	392.178
GSM256961	Wild type rep 3	367.379
GSM256956	CLC5 ko rep 1	507.455
GSM256957	CLC5 ko rep 2	518.388
GSM256958	CLC5 ko rep 3	466.324

Profile: ACE2 expression
GDS3323 / 1425103_at
Title
Na+/H+ exchanger 3 deficiency effect on the colon
Organism
Mus musculus

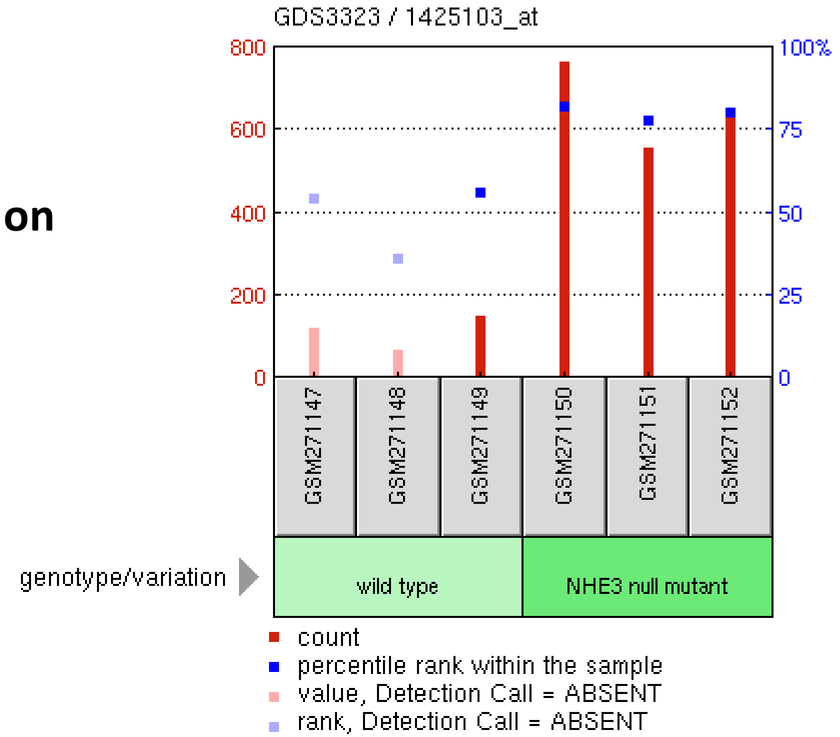

Sample	Title	Value
GSM271147	NHE3+/+ replicate 1	122.3
GSM271148	NHE3+/+ replicate 2	68.2
GSM271149	NHE3+/+ replicate 3	150.2
GSM271150	NHE3-/- replicate 1	762.3
GSM271151	NHE3-/- replicate 2	554.9
GSM271152	NHE3-/- replicate 3	634.3

Profile: ACE2 expression
GDS4910 / 1425103_at
Title
Stearoyl-CoA desaturase-1 deficiency effect on the skin
Organism
Mus musculus

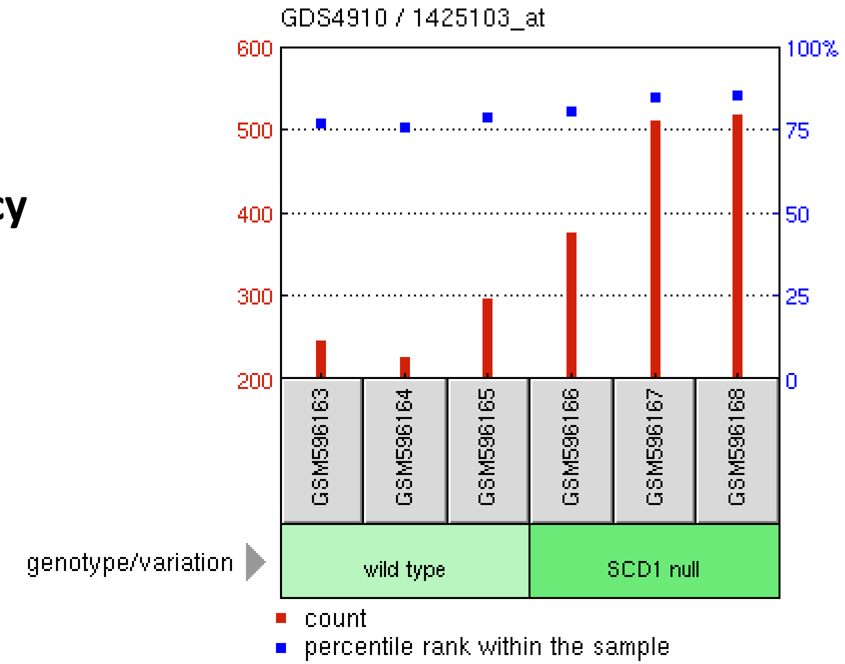

Sample	Title	Value
GSM596163	Skin_Lox_Control_Rep1	246.595
GSM596164	Skin_Lox_Control_Rep2	227.702
GSM596165	Skin_Lox_Control_Rep3	297.552
GSM596166	Skin_SKO_Rep1	377.151
GSM596167	Skin_SKO_Rep2	512
GSM596168	Skin_SKO_Rep3	519.147

Profile: ACE2 expression
GDS4910 / 1452138_a_at
Title
Stearoyl-CoA desaturase-1 deficiency effect on the skin
Organism
Mus musculus

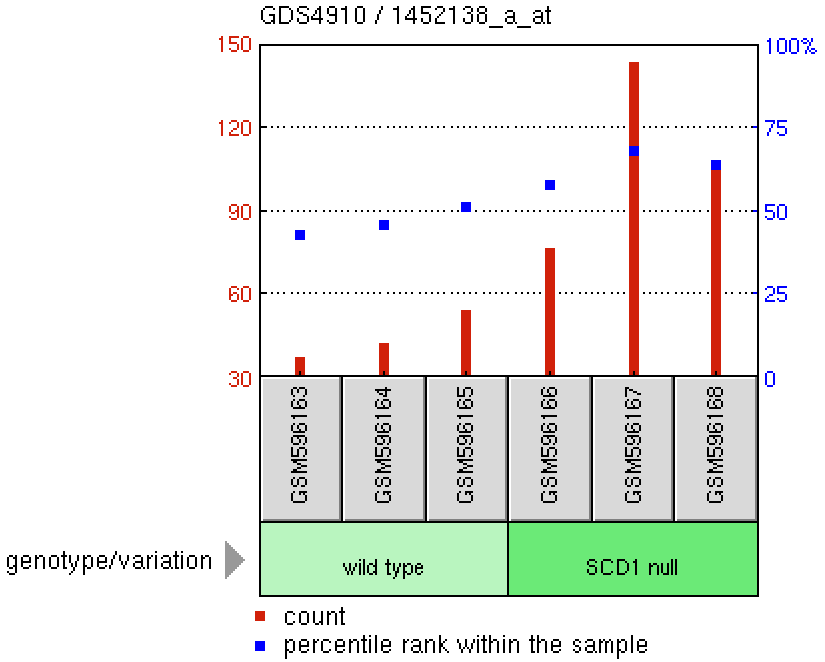

Sample	Title	Value
GSM596163	Skin_Lox_Control_Rep1	37.453
GSM596164	Skin_Lox_Control_Rep2	42.992
GSM596165	Skin_Lox_Control_Rep3	53.967
GSM596166	Skin_SKO_Rep1	76.692
GSM596167	Skin_SKO_Rep2	143.908
GSM596168	Skin_SKO_Rep3	105.639

Profile: ACE2 expression
GDS4910 / 1425102_a_at
Title
Stearoyl-CoA desaturase-1 deficiency effect on the skin
Organism
Mus musculus

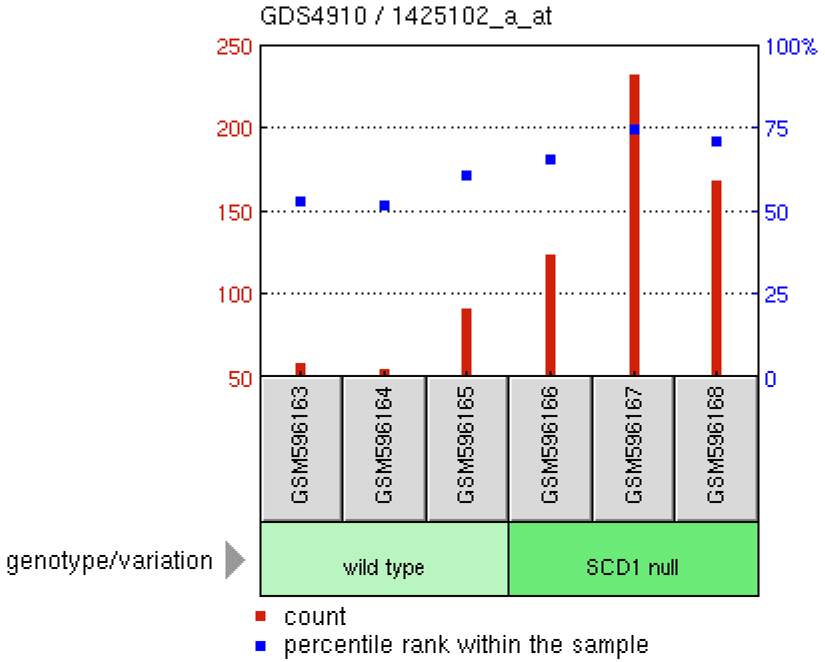

Sample	Title	Value
GSM596163	Skin_Lox_Control_Rep1	59.631
GSM596164	Skin_Lox_Control_Rep2	55.139
GSM596165	Skin_Lox_Control_Rep3	91.71
GSM596166	Skin_SKO_Rep1	123.983
GSM596167	Skin_SKO_Rep2	233.131
GSM596168	Skin_SKO_Rep3	168.429

Profile: ACE2 expression
GDS5087 / 1425102_a_at
Title
Transcriptional regulator steroid receptor coactivator-2 deficiency effect on the heart
Organism
Mus musculus

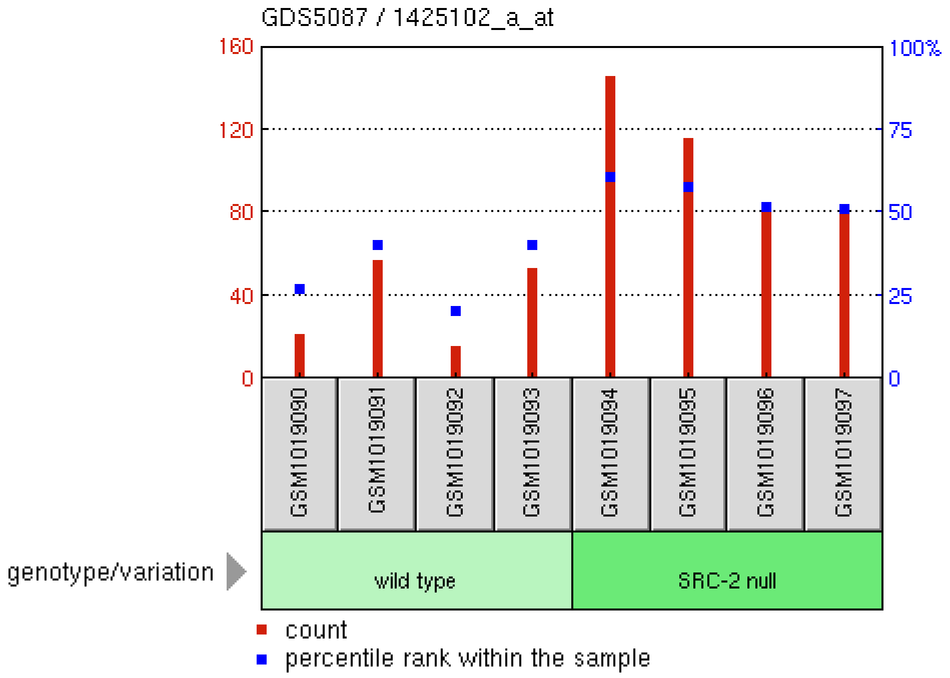

Sample	Title	Value
GSM1019090	Heart_WT_1	22.0184
GSM1019091	Heart_WT_2	57.5679
GSM1019092	Heart_WT_3	15.678
GSM1019093	Heart_WT_4	53.6421
GSM1019094	Heart_KO_1	145.776
GSM1019095	Heart_KO_2	116.278
GSM1019096	Heart_KO_3	85.0996
GSM1019097	Heart_KO_4	84.2244

Supplemental Figure S14. SARS-CoV-2 interactome: 332 genes encoding host protein preys of the coronavirus SARS-CoV-2 proteins

David E. Gordon, Gwendolyn M. Jang, Mehdi Bouhaddou, Jiewei Xu, Kirsten Obernier, Matthew J. O'Meara, Jeffrey Z. Guo, Danielle L. Swaney, Tia A. Tummino, Ruth Huettenhain, Robyn M. Kaake, Alicia L. Richards, Beril Tutuncuoglu, Helene Foussard, Jyoti Batra, N. J. K. A SARS-CoV-2-Human Protein-Protein Interaction Map Reveals Drug Targets and Potential Drug Repurposing. (2020). **doi:** https://doi.org/10.1101/2020.03.22.002386

ABSTRACT

An outbreak of the novel coronavirus SARS-CoV-2, the causative agent of COVID-19 respiratory disease, has infected over 290,000 people since the end of 2019, killed over 12,000, and caused worldwide social and economic disruption1,2. There are currently no antiviral drugs with proven efficacy nor are there vaccines for its prevention. Unfortunately, the scientific community has little knowledge of the molecular details of SARS-CoV-2 infection. To illuminate this, we cloned, tagged and expressed 26 of the 29 viral proteins in human cells and identified the human proteins physically associated with each using affinity-purification mass spectrometry (AP-MS), which identified 332 high confidence SARS-CoV-2-human protein-protein interactions (PPIs). Among these, we identify 67 druggable human proteins or host factors targeted by 69 existing FDA-approved drugs, drugs in clinical trials and/or preclinical compounds, that we are currently evaluating for efficacy in live SARS-CoV-2 infection assays. The identification of host dependency factors mediating virus infection may provide key insights into effective molecular targets for developing broadly acting antiviral therapeutics against SARS-CoV-2 and other deadly coronavirus strains.

Gordon et a. A SARS-CoV-2-Human Protein-Protein Interaction Map Reveals Drug Targets and Potential Drug Repurposing. (2020). doi: https://doi.org/10.1101/2020.03.22.002386

GSEA of the 332 genes encoding human prey proteins for 27 SARS-CoV-2 viral proteins.

Ligand Perturbations from GEO down

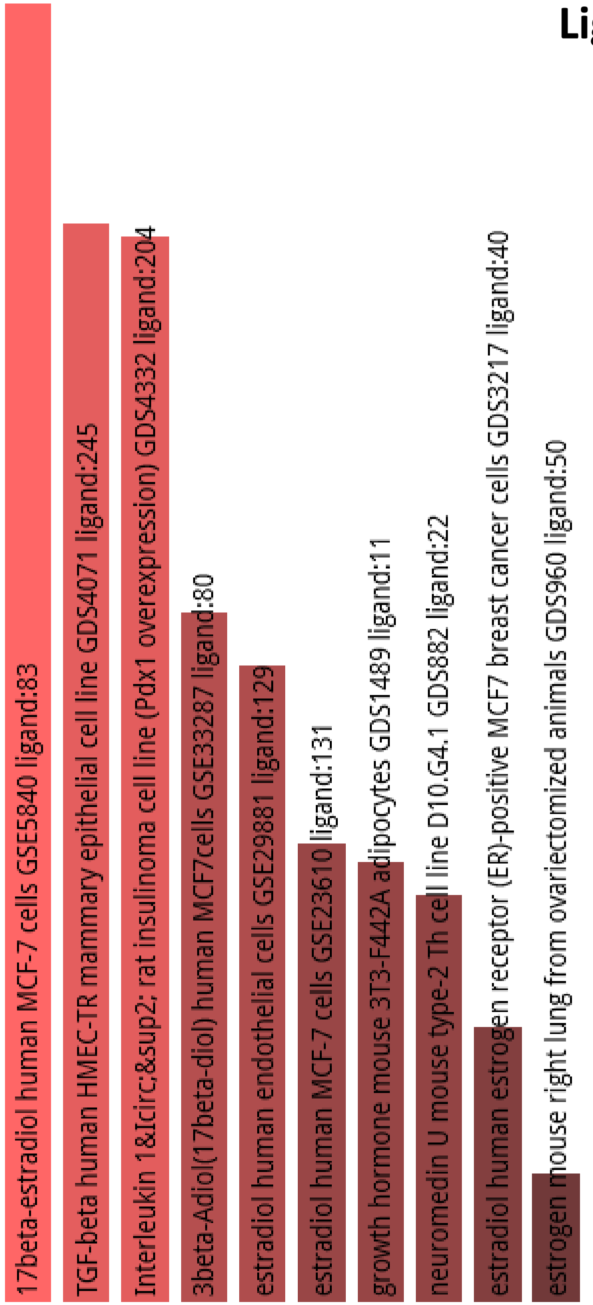

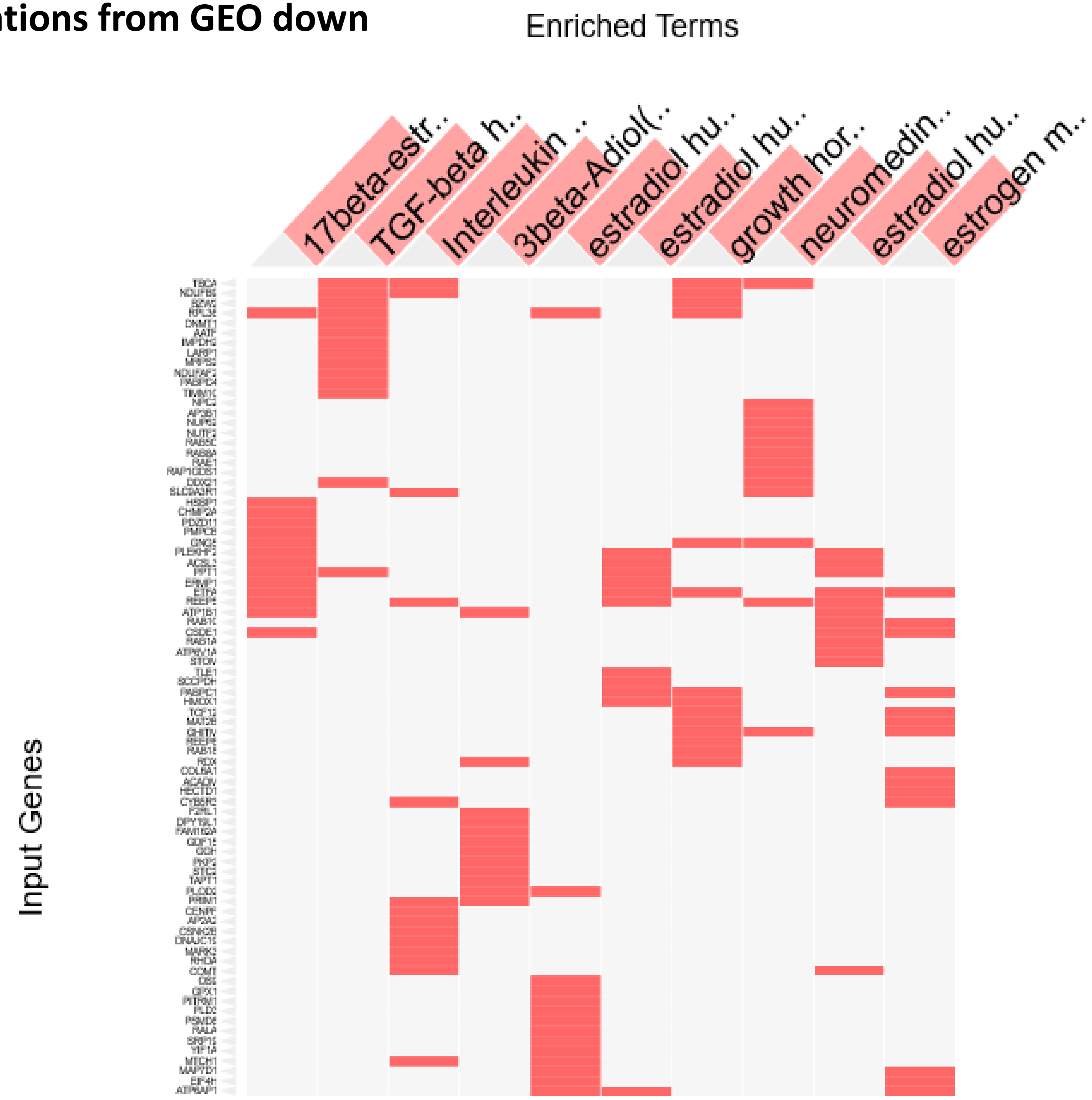

Ligand Perturbations from GEO up

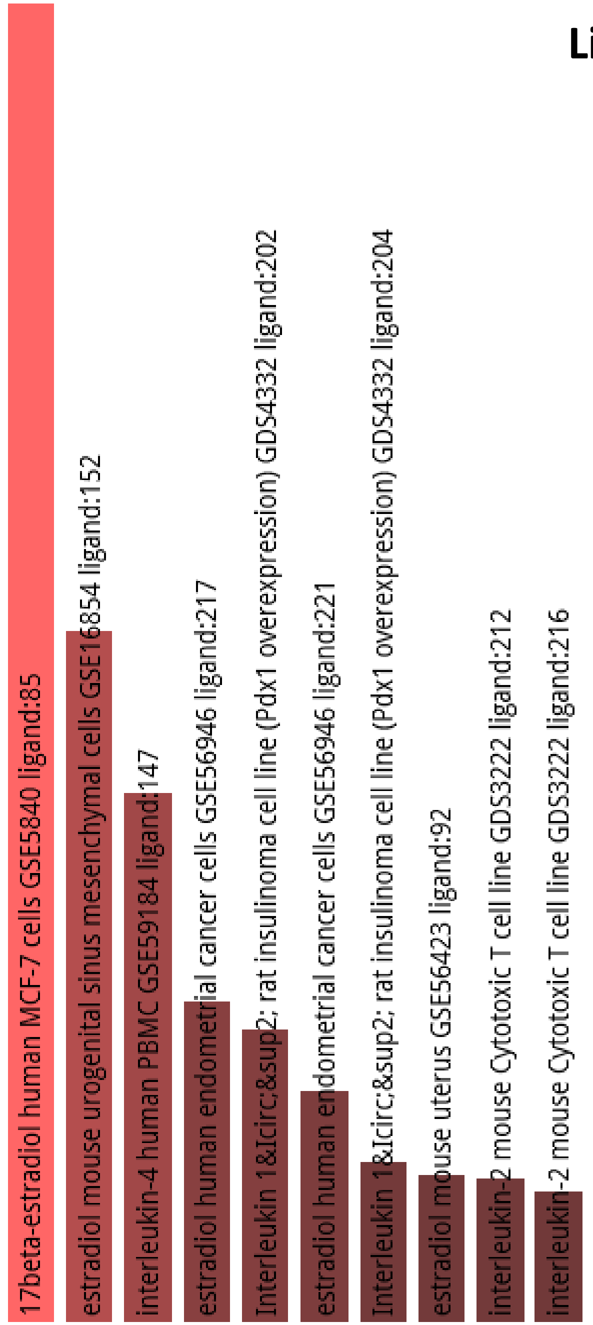

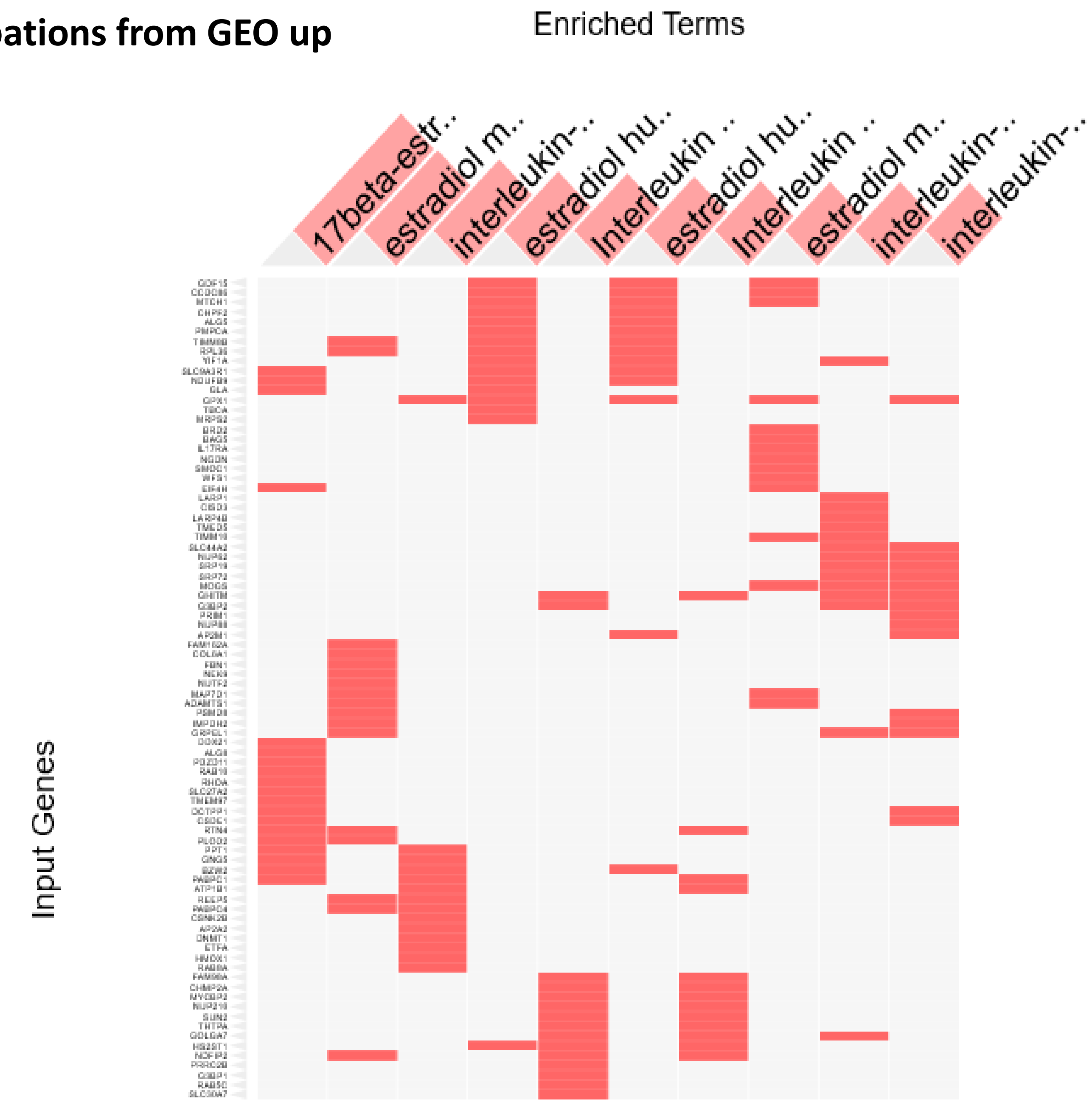

Virus Perturbations from GEO up

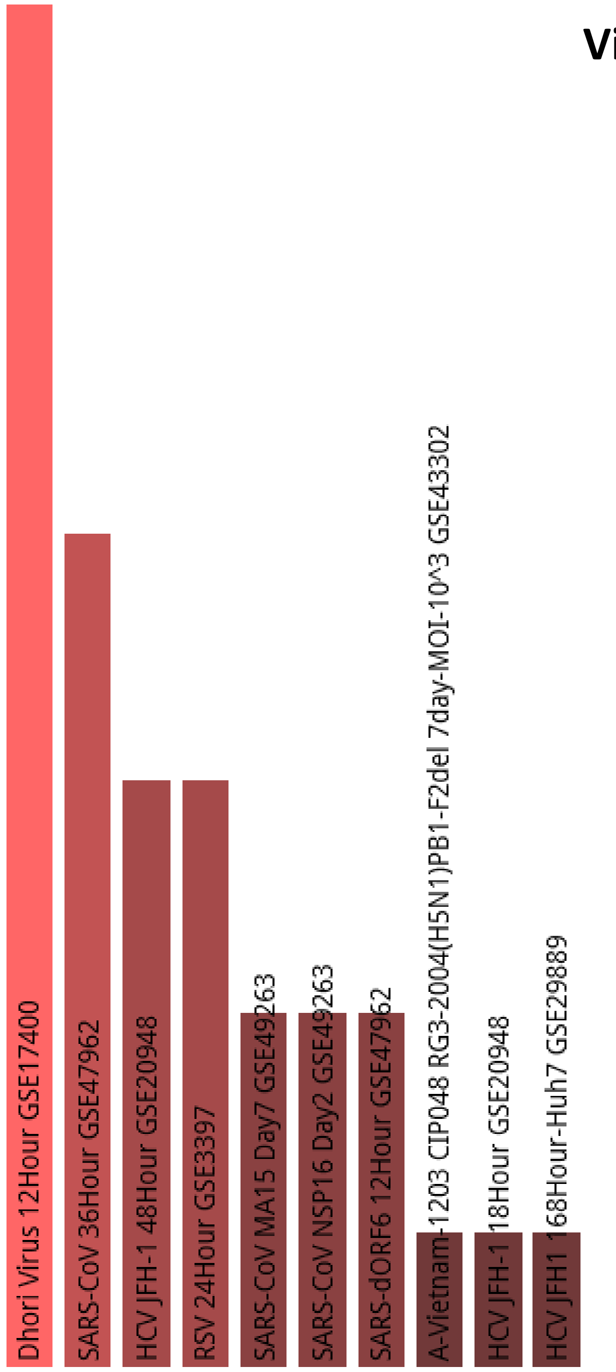

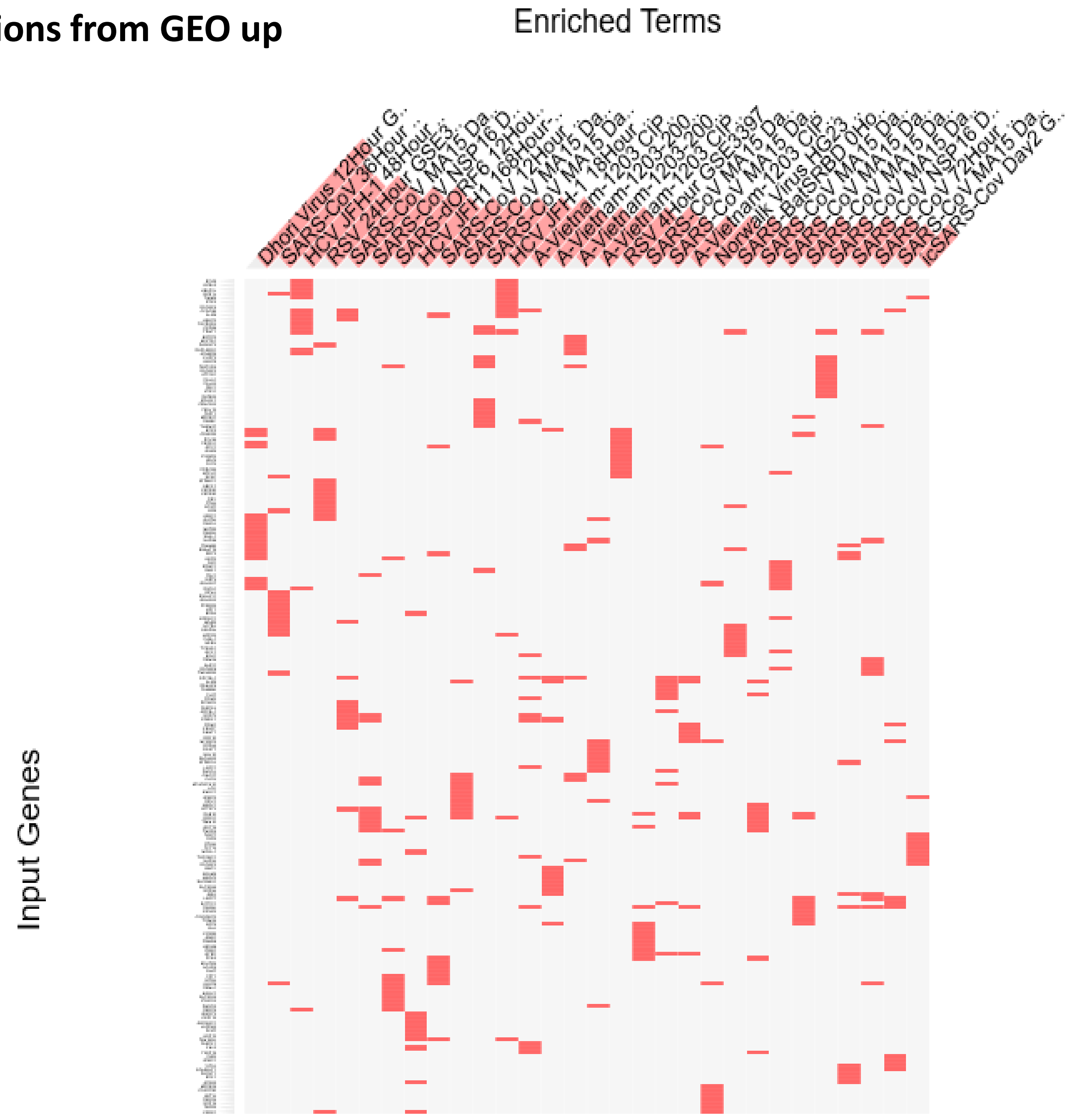

Virus Perturbations from GEO down

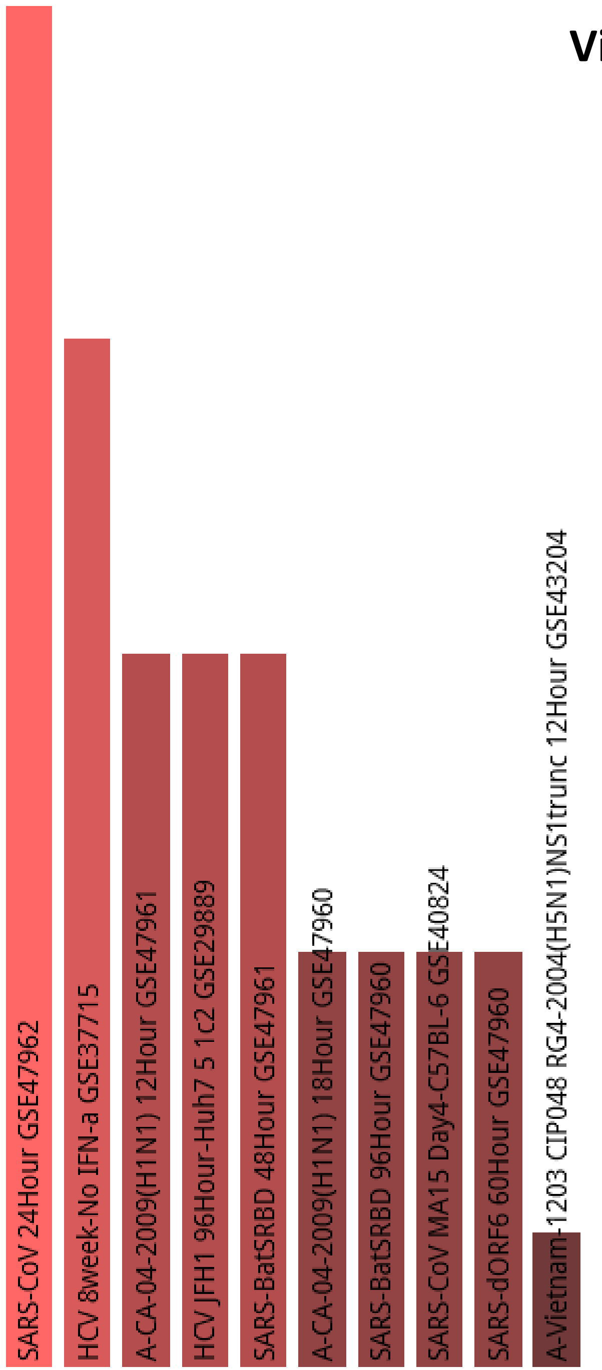

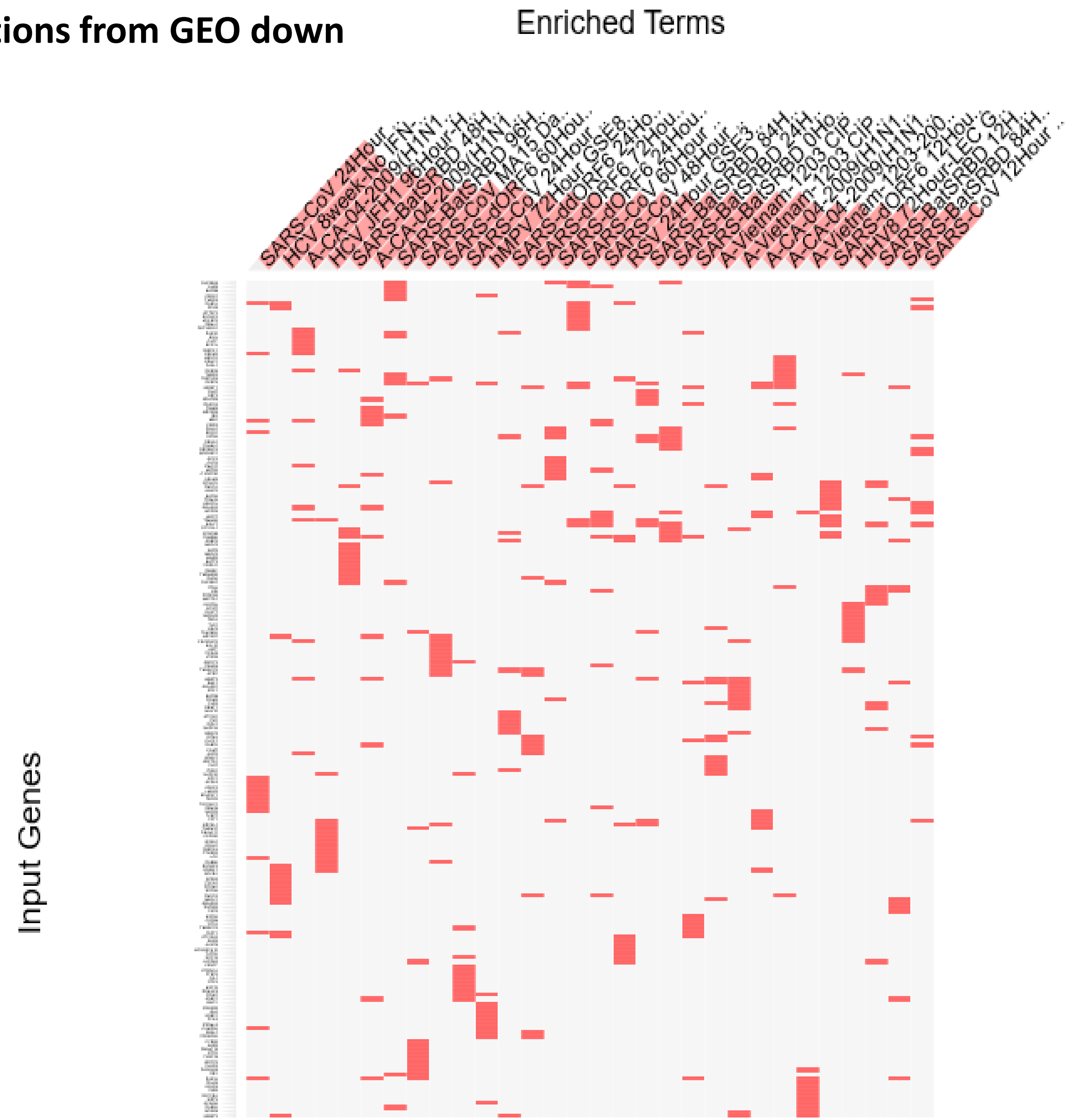

GO Biological Process 2018

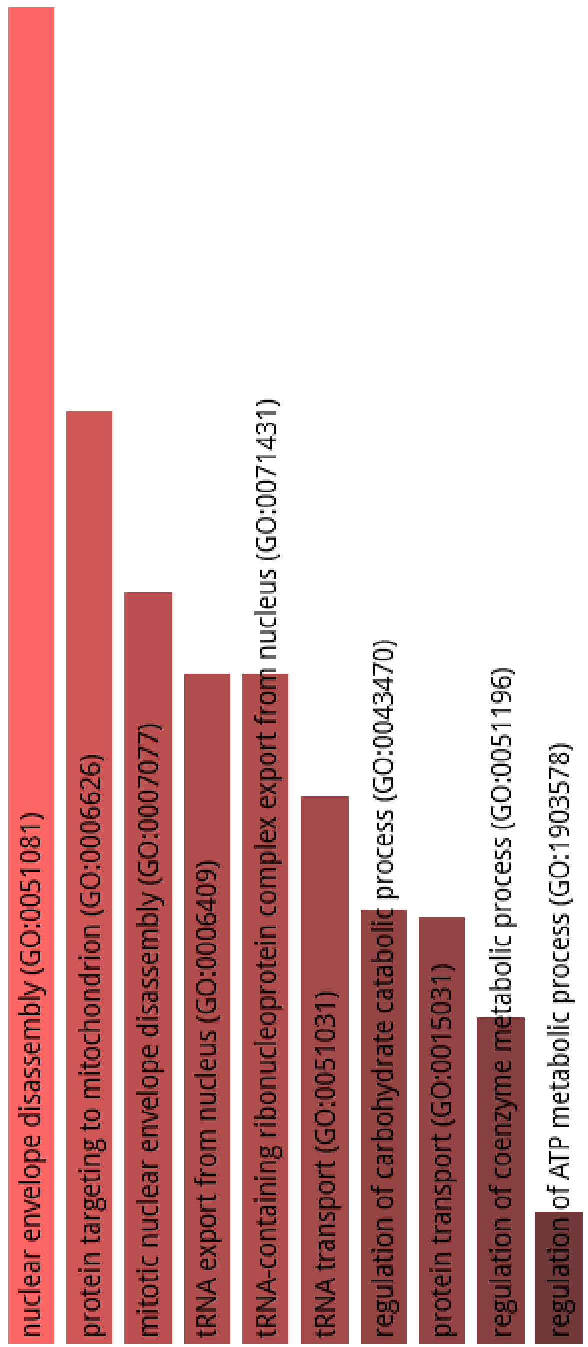

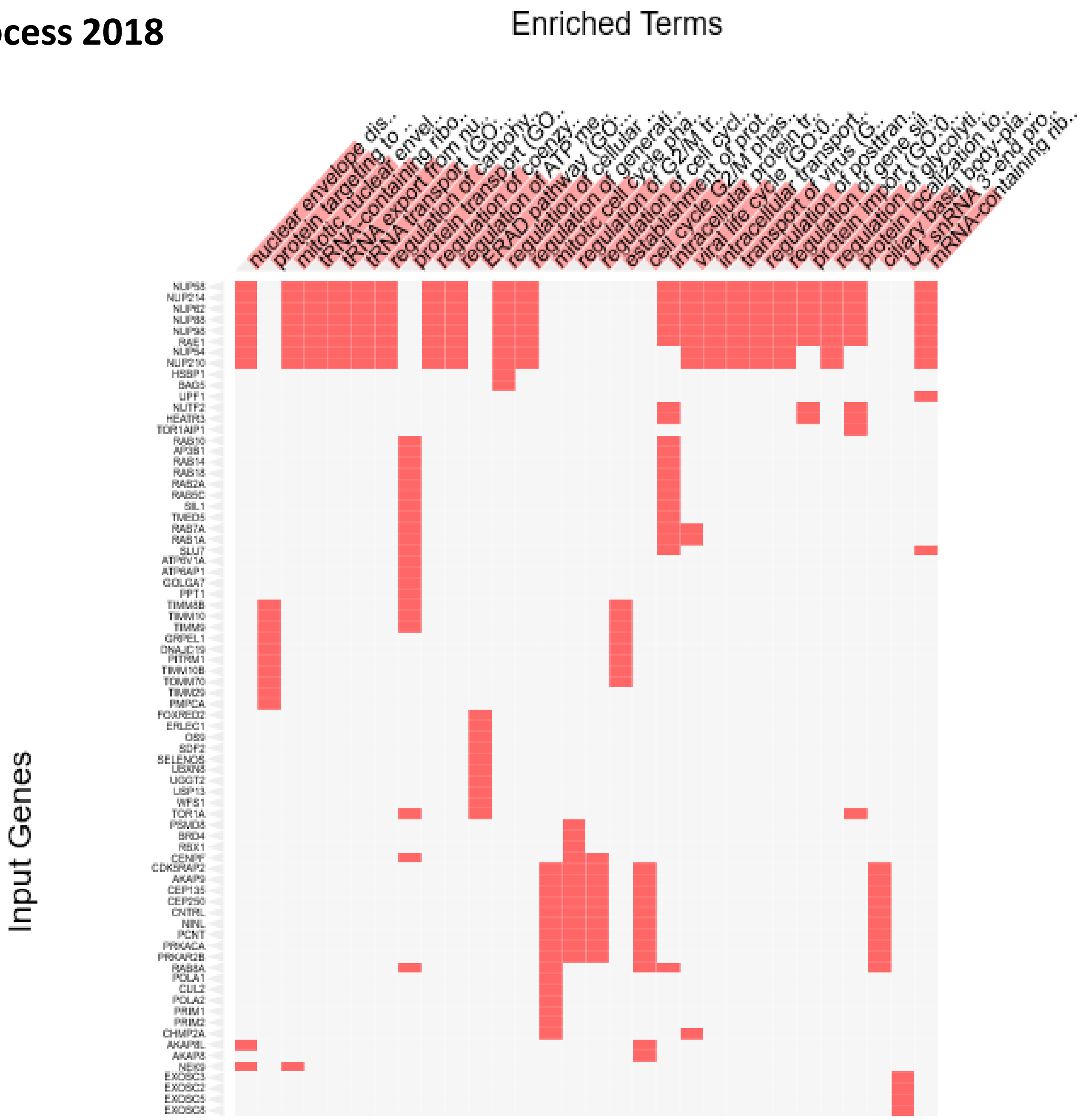

GO Biological Process 2018

Bait	PreyGene
**SARS-CoV2 nsp9**	***NUP54***
**SARS-CoV2 nsp9**	***NUP214***
**SARS-CoV2 nsp9**	***NUP58***
**SARS-CoV2 nsp9**	***NUP62***
**SARS-CoV2 nsp9**	***NUP88***
**SARS-CoV2 orf6**	***NUP98***
**SARS-CoV2 orf6**	***RAE1***
**SARS-CoV2 nsp4**	***NUP210***

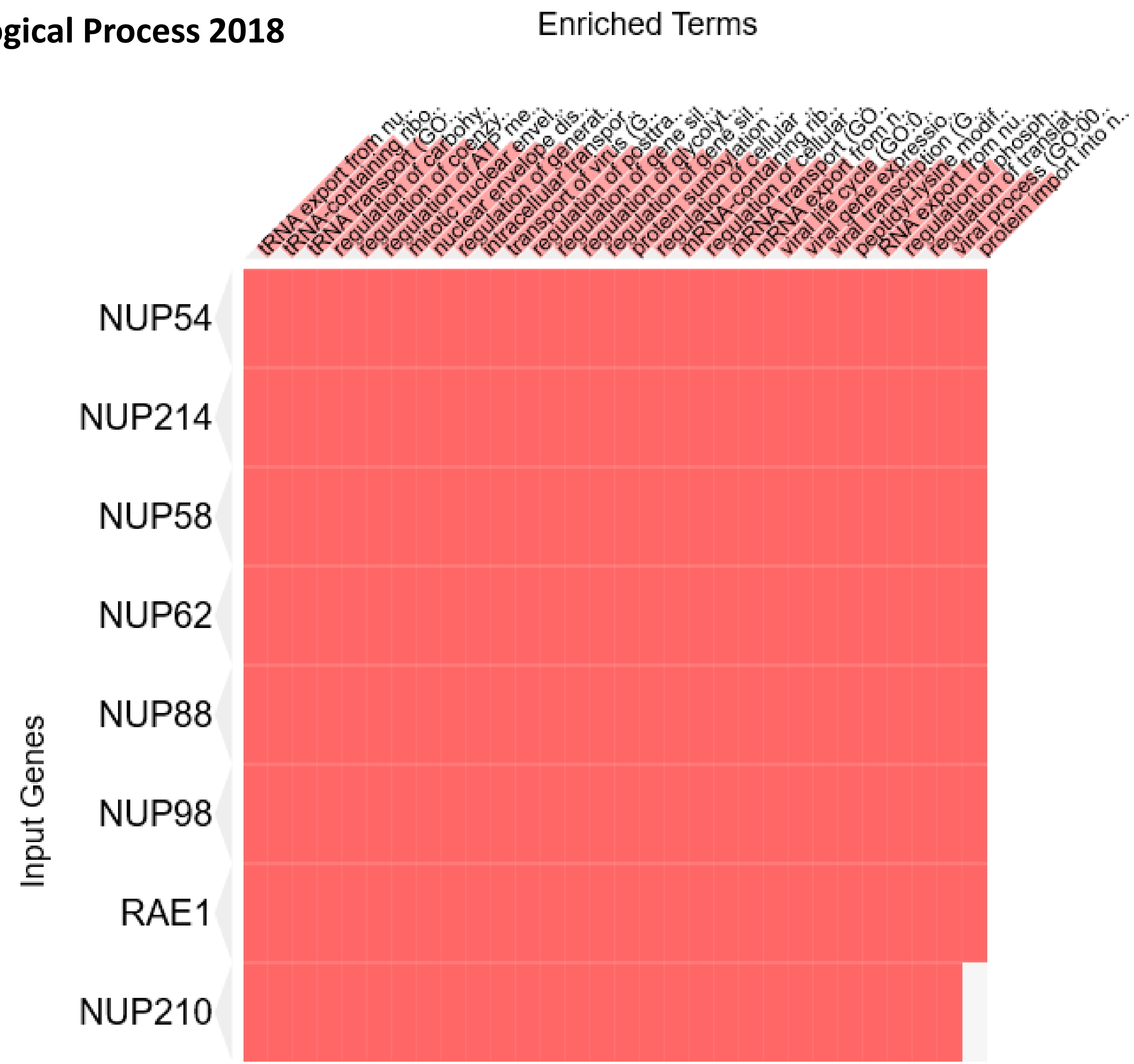

Virus Perturbations from GEO up

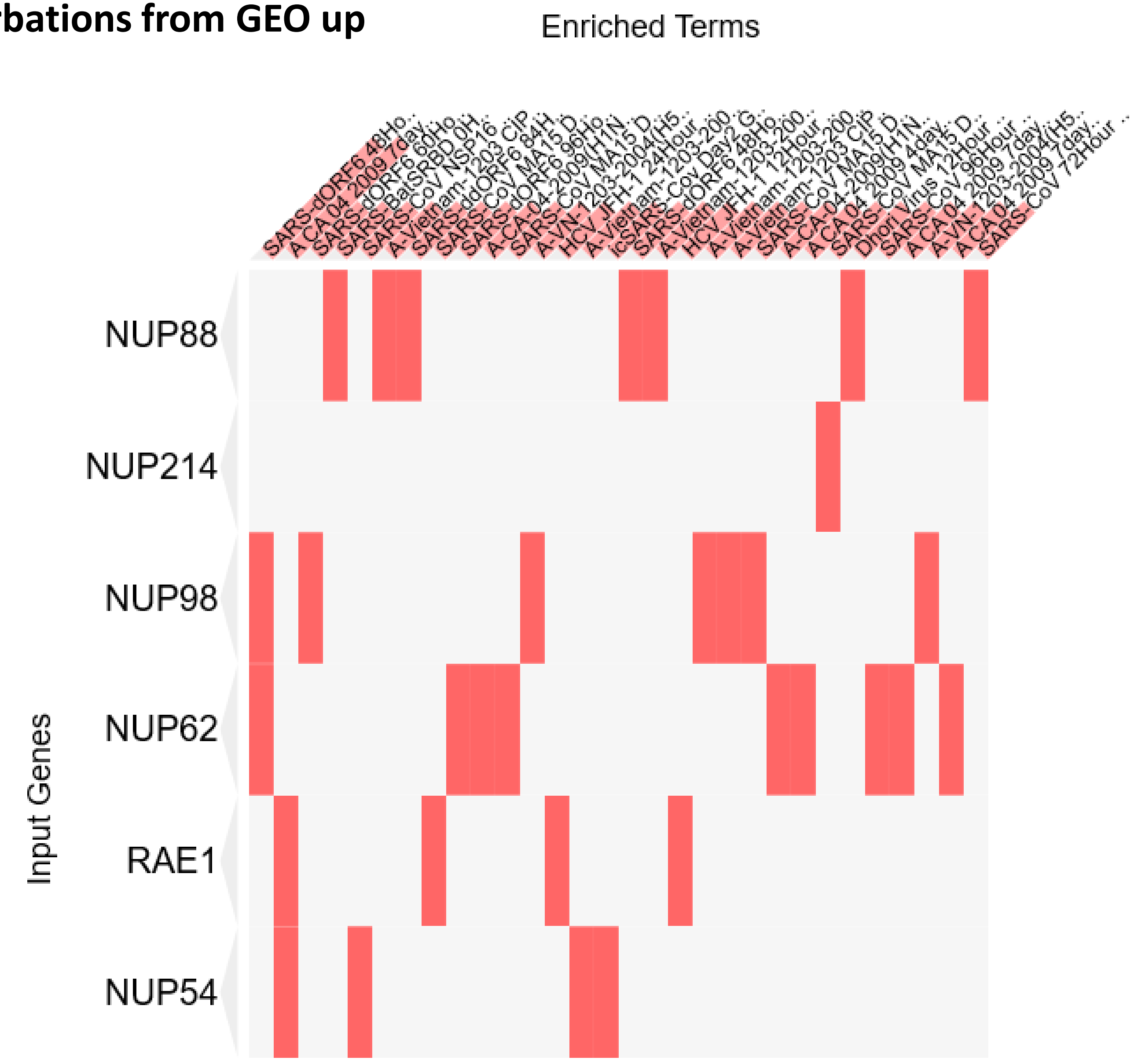

Virus Perturbations from GEO down

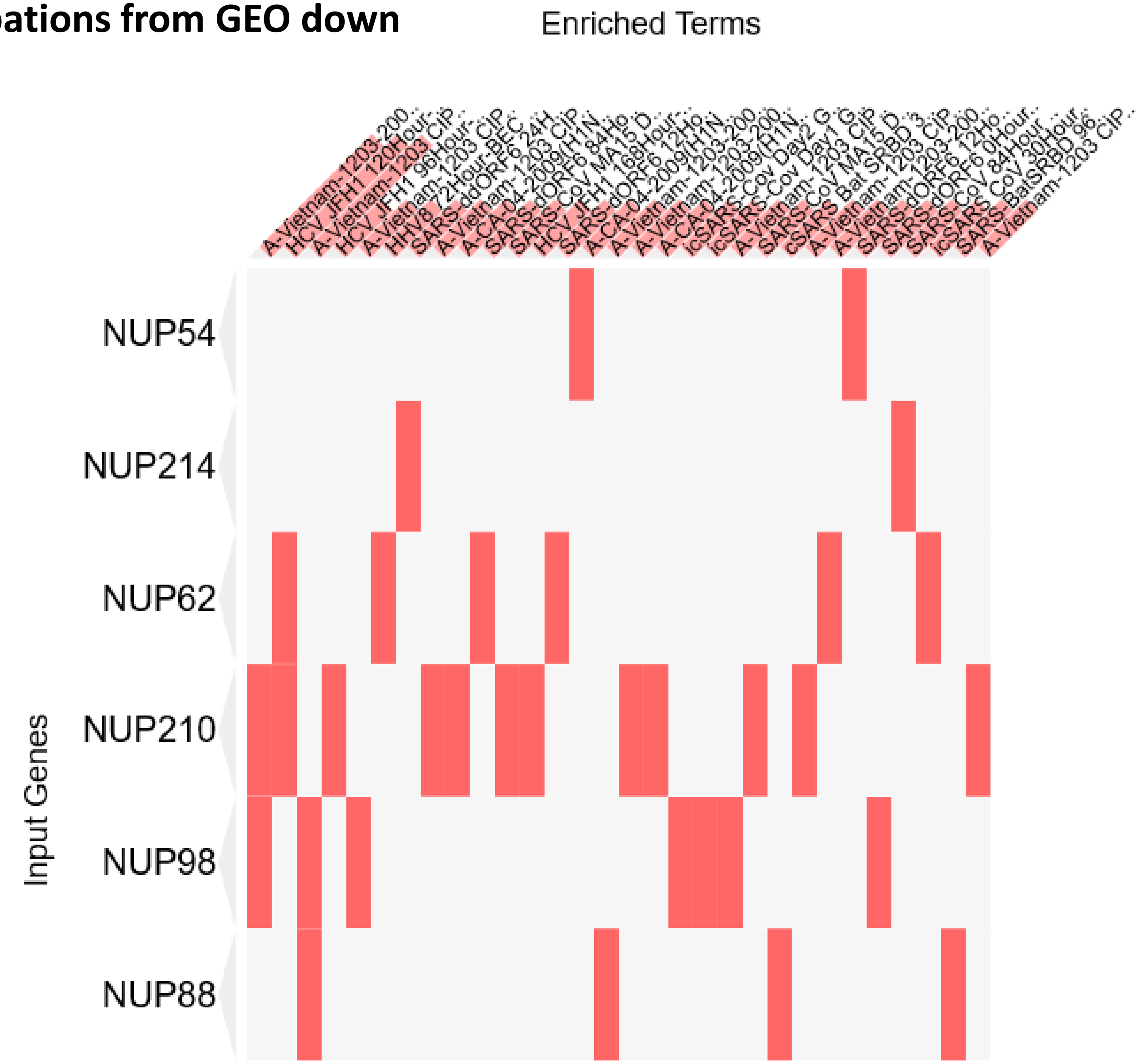

GO Molecular Function 2018

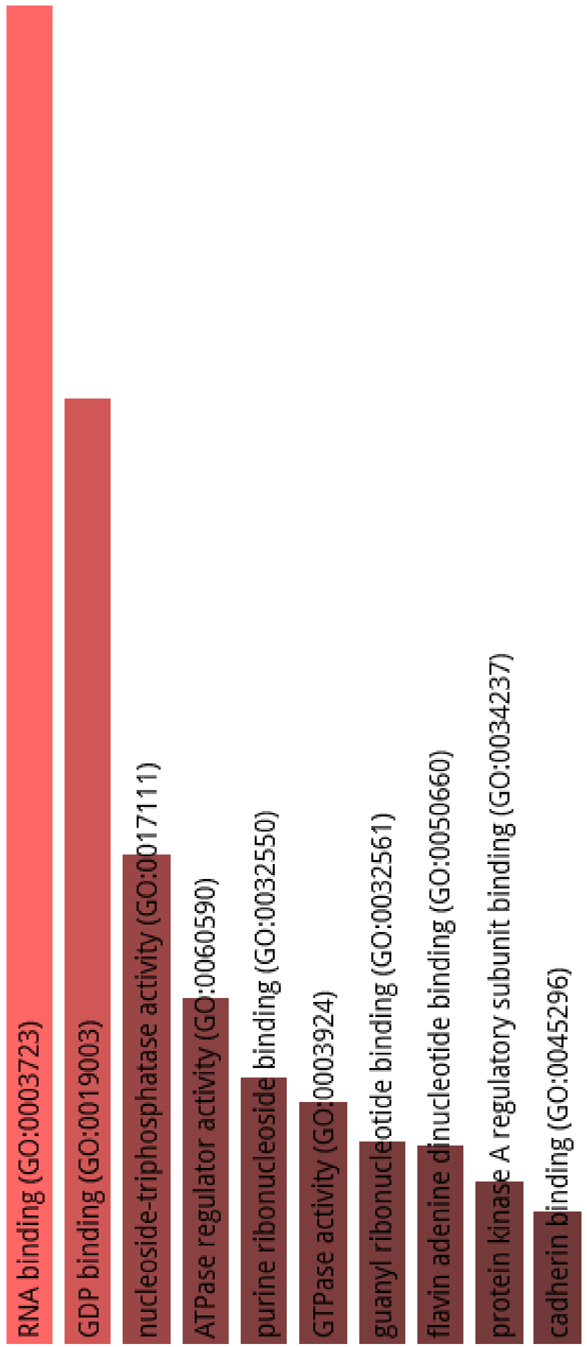

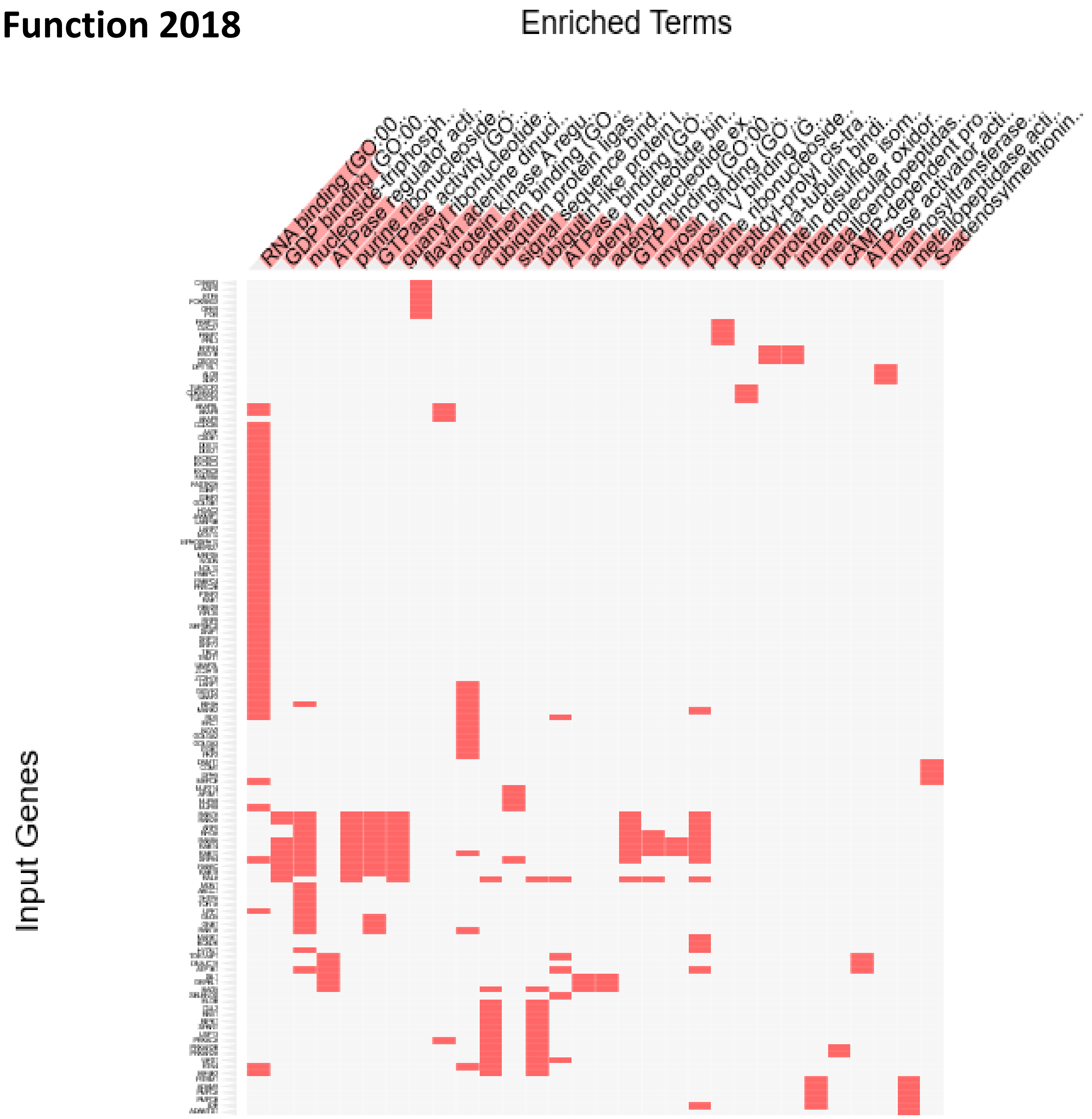

GO Cellular Component 2018

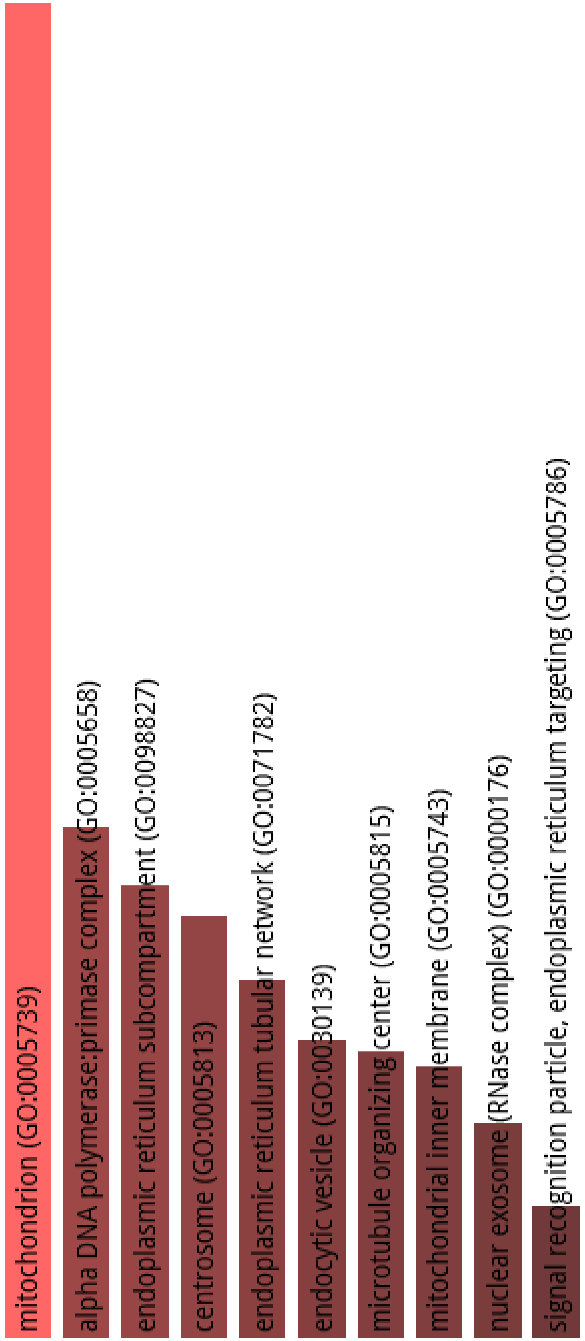

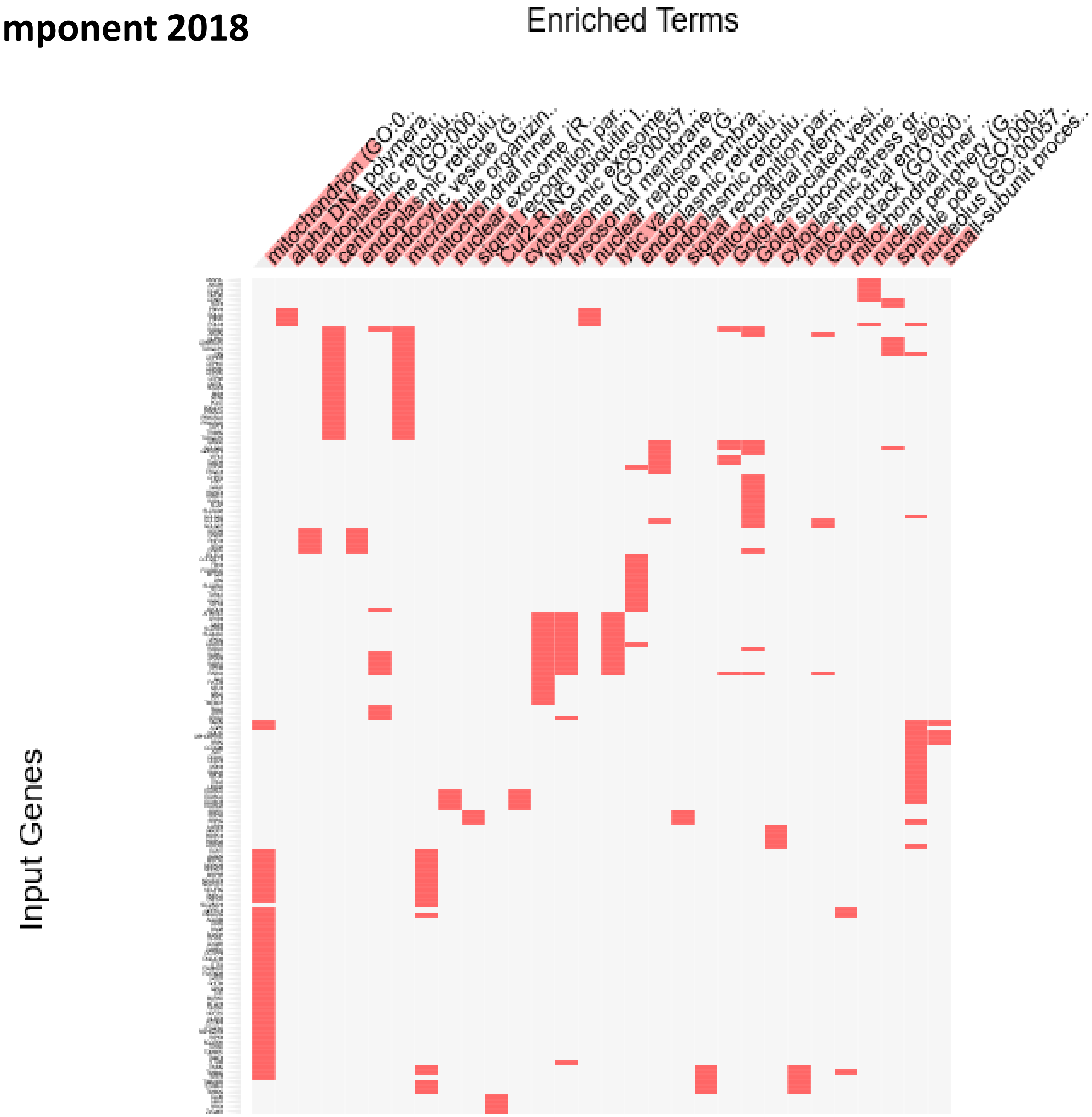

SARS-CoV-2 interactome: 332 genes encoding host protein preys of the coronavirus SARS-CoV-2 proteins

Quercetin alters expression of 98 of 332 (30%) genes encoding human protein preys for 23 of 27 (85%) SARS-CoV-2 proteins

A

Quercetin alters expression of 98 of 332 (30%) genes encoding human protein preys for 23 of 27 (85%) SARS-CoV-2 proteins

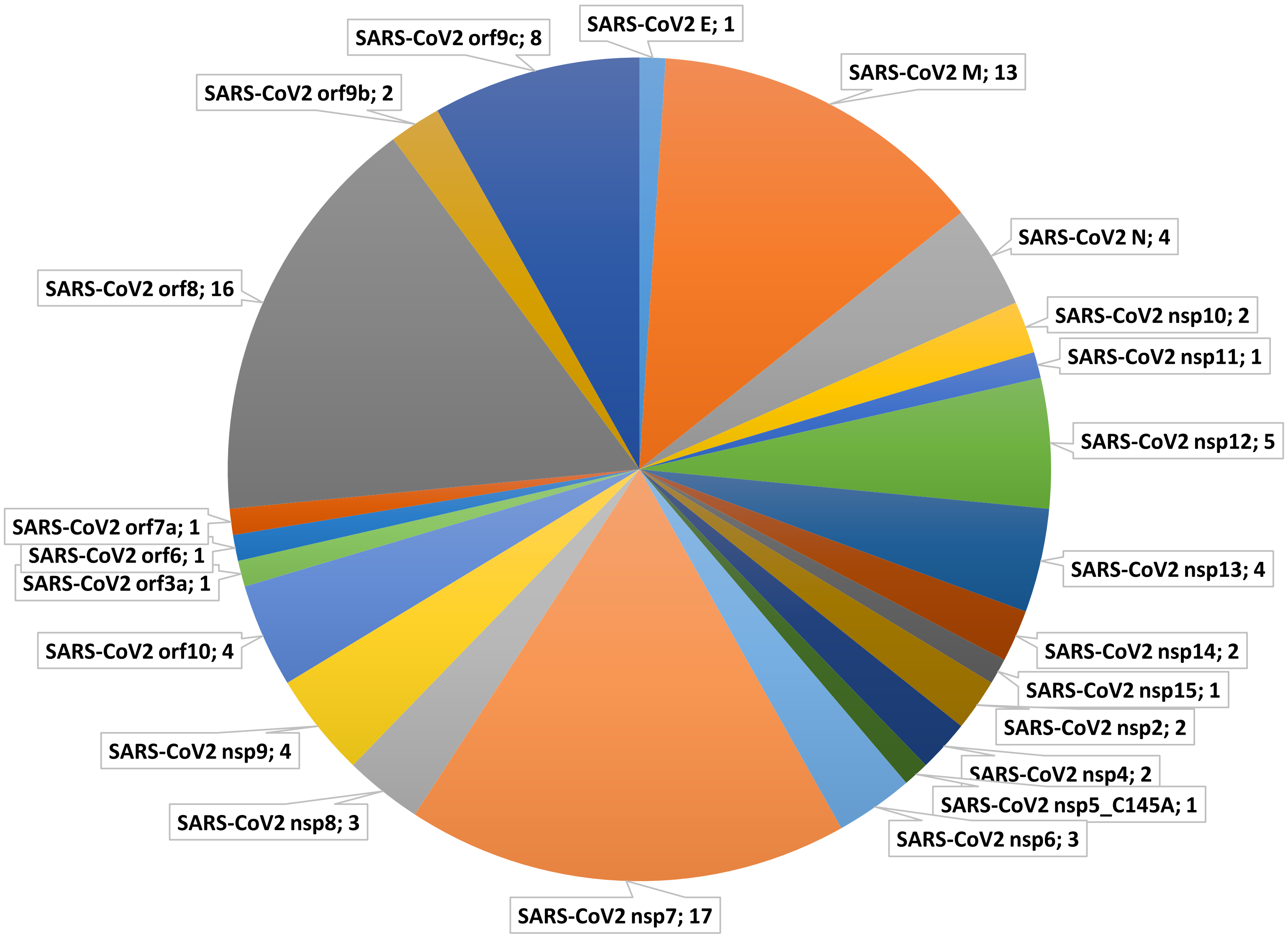

SARS-CoV-2 interactome:
332 genes encoding host protein preys of the coronavirus SARS-CoV-2 proteins

Vitamin D alters expression of 84 of 332 (25%) genes encoding human protein preys for 19 of 27 (70%) SARS-CoV-2 proteins

B

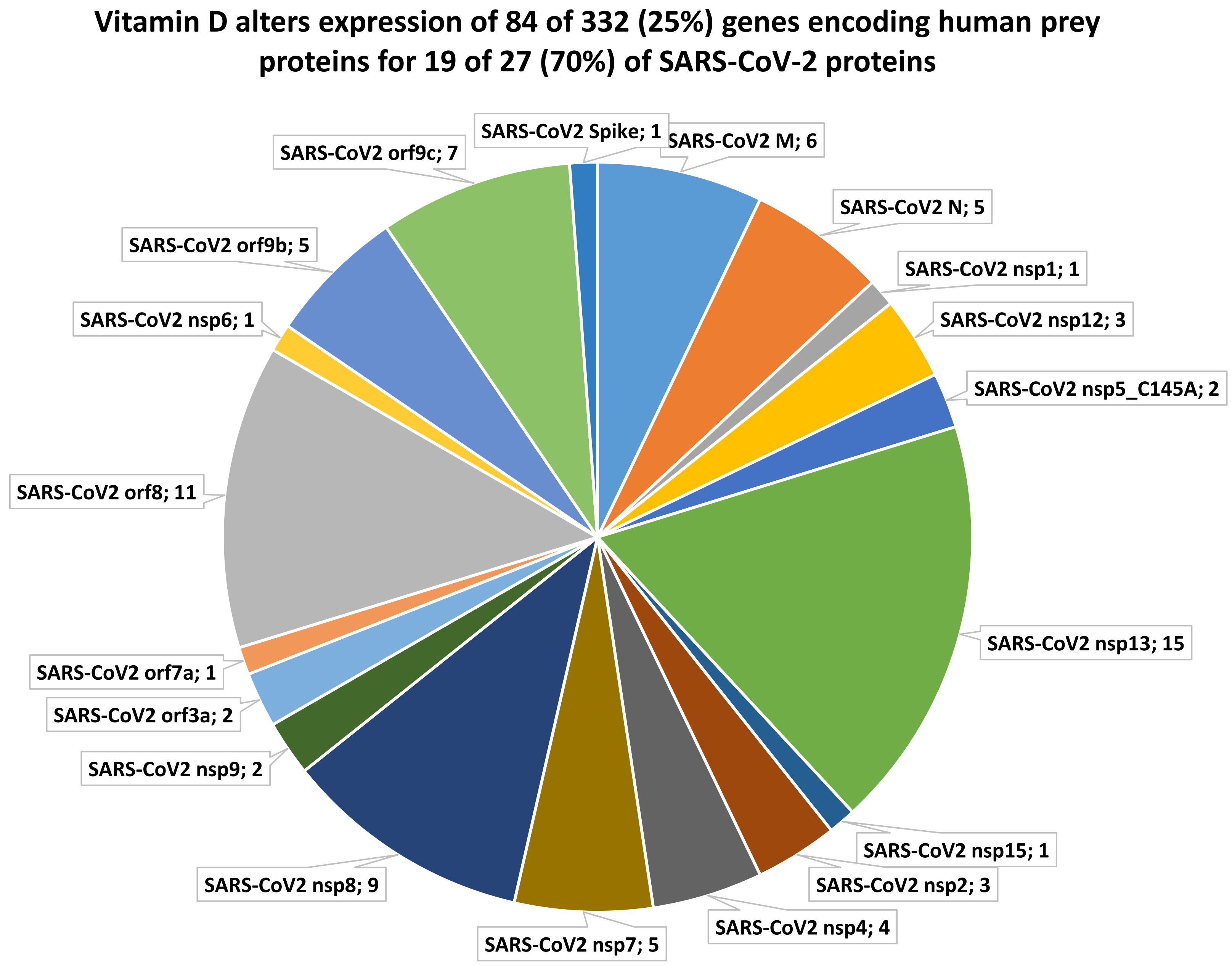
Vitamin D alters expression of 84 of 332 (25%) genes encoding human prey proteins for 19 of 27 (70%) of SARS-CoV-2 proteins
SARS-CoV2 Spike; 1
SARS-CoV2 M; 6
SARS-CoV2 N; 5
SARS-CoV2 nsp1; 1
SARS-CoV2 nsp12; 3
SARS-CoV2 nsp5_C145A; 2
SARS-CoV2 nsp13; 15
SARS-CoV2 nsp15; 1
SARS-CoV2 nsp2; 3
SARS-CoV2 nsp4; 4
SARS-CoV2 nsp7; 5
SARS-CoV2 nsp8; 9
SARS-CoV2 nsp9; 2
SARS-CoV2 orf3a; 2
SARS-CoV2 orf7a; 1
SARS-CoV2 orf8; 11
SARS-CoV2 nsp6; 1
SARS-CoV2 orf9b; 5
SARS-CoV2 orf9c; 7

Effects of Estradiol on expression of genes encoding human prey proteins of the SARS-CoV-2 coronavirus.

Estradiol alters expression of 203 of 332 (61%) of genes encoding human prey proteins for 26 of 27 (96%) of SARS-CoV-2 proteins.

A

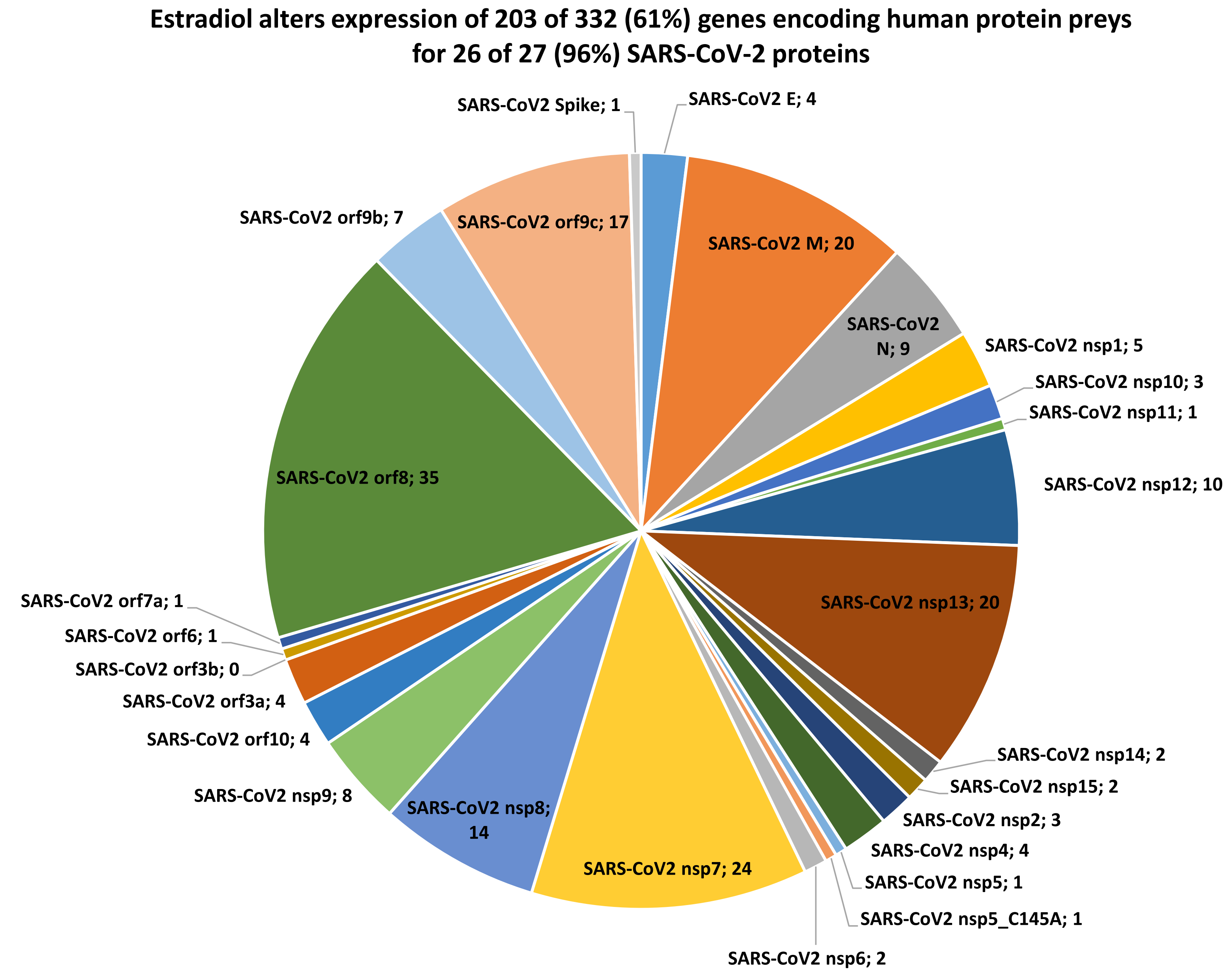
Estradiol alters expression of 203 of 332 (61%) genes encoding human protein preys
for 26 of 27 (96%) SARS-CoV-2 proteins
SARS-CoV2 Spike; 1
SARS-CoV2 E; 4
SARS-CoV2 orf9b; 7
SARS-CoV2 orf9c; 17
SARS-CoV2 M; 20
SARS-CoV2 N; 9
SARS-CoV2 nsp1; 5
SARS-CoV2 nsp10; 3
SARS-CoV2 nsp11; 1
SARS-CoV2 orf8; 35
SARS-CoV2 nsp12; 10
SARS-CoV2 nsp13; 20
SARS-CoV2 orf7a; 1
SARS-CoV2 orf6; 1
SARS-CoV2 orf3b; 0
SARS-CoV2 orf3a; 4
SARS-CoV2 orf10; 4
SARS-CoV2 nsp14; 2
SARS-CoV2 nsp15; 2
SARS-CoV2 nsp9; 8
SARS-CoV2 nsp8; 14
SARS-CoV2 nsp2; 3
SARS-CoV2 nsp4; 4
SARS-CoV2 nsp5; 1
SARS-CoV2 nsp7; 24
SARS-CoV2 nsp5_C145A; 1
SARS-CoV2 nsp6; 2
